# Synthesis and migrastatic activity of cytochalasin analogues lacking a macrocyclic moiety†

**DOI:** 10.1039/d3md00535f

**Published:** 2023-11-28

**Authors:** Bedřich Formánek, Dorian Dupommier, Tereza Volfová, Silvie Rimpelová, Aneta Škarková, Jana Herciková, Daniel Rösel, Jan Brábek, Pavla Perlíková

**Affiliations:** a Department of Organic Chemistry, Faculty of Chemical Technology, University of Chemistry and Technology, Prague Technická 5 166 28 Prague Czech Republic perlikop@vscht.cz; b Department of Cell Biology, BIOCEV, Faculty of Science, Charles University Průmyslová 595, 252 50 Vestec Prague West Czech Republic; c Department of Biochemistry and Microbiology, Faculty of Food and Biochemical Technology, University of Chemistry and Technology Prague Technická 5 166 28 Prague The Czech Republic; d Institute of Organic Chemistry and Biochemistry, Czech Academy of Sciences Flemingovo nám. 2 160 00 Prague Czech Republic

## Abstract

Cytochalasans are known as inhibitors of actin polymerization and for their cytotoxic and migrastatic activity. In this study, we synthesized a series of cytochalasin derivatives that lack a macrocyclic moiety, a structural element traditionally considered essential for their biological activity. We focused on substituting the macrocycle with simple aryl-containing sidechains, and we have also synthesized compounds with different substitution patterns on the cytochalasin core. The cytochalasin analogues were screened for their migrastatic and cytotoxic activity. Compound 24 which shares the substitution pattern with natural cytochalasins B and D exhibited not only significant *in vitro* migrastatic activity towards BLM cells but also demonstrated inhibition of actin polymerization, with no cytotoxic effect observed at 50 μM concentration. Our results demonstrate that even compounds lacking the macrocyclic moiety can exhibit biological activities, albeit less pronounced than those of natural cytochalasins. However, our findings emphasize the pivotal role of substituting the core structure in switching between migrastatic activity and cytotoxicity. These findings hold significant promise for further development of easily accessible cytochalasan analogues as novel migrastatic agents.

## Abstract 2

Macrocyclic moiety is not essential for the biological activity of cytochalasan analogues.

## Graphical abstract

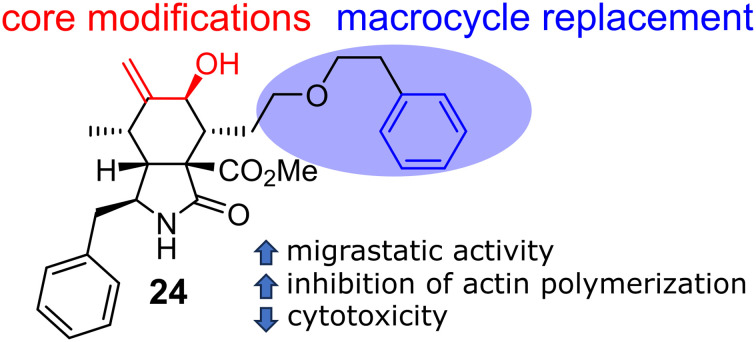

## Introduction

Significant strides have been achieved in the treatment of solid tumours. However, cancer remains a serious disease owing to the metastatic dissemination of cancer cells that is responsible for 90% of cancer-related fatalities.^1,2^ Despite this reality, current cancer therapeutics do not target metastasis. In recent years, an emphasis has been placed on re-evaluating the paradigm of novel cancer drug development. This paradigm shift encompasses not only a focus on compounds exhibiting antiproliferative/cytotoxic effects (termed ‘cytostatics’), but also a consideration of compounds impeding cancer cell invasion and migration, dubbed ‘migrastatics’.^3–5^ The integration of migrastatics into existing cancer treatment stands as a promising avenue to alleviate the potentially lethal consequences associated with cancer.

Actin microfilament dynamics, *i.e.* its polymerization and depolymerization, play a pivotal role in cell motility across all modes of cancer cell migration.^6^ Hence, agents that interfere with actin microfilament dynamics, such as cytochalasans, emerge as promising migrastatics.

**Fig. 1:**
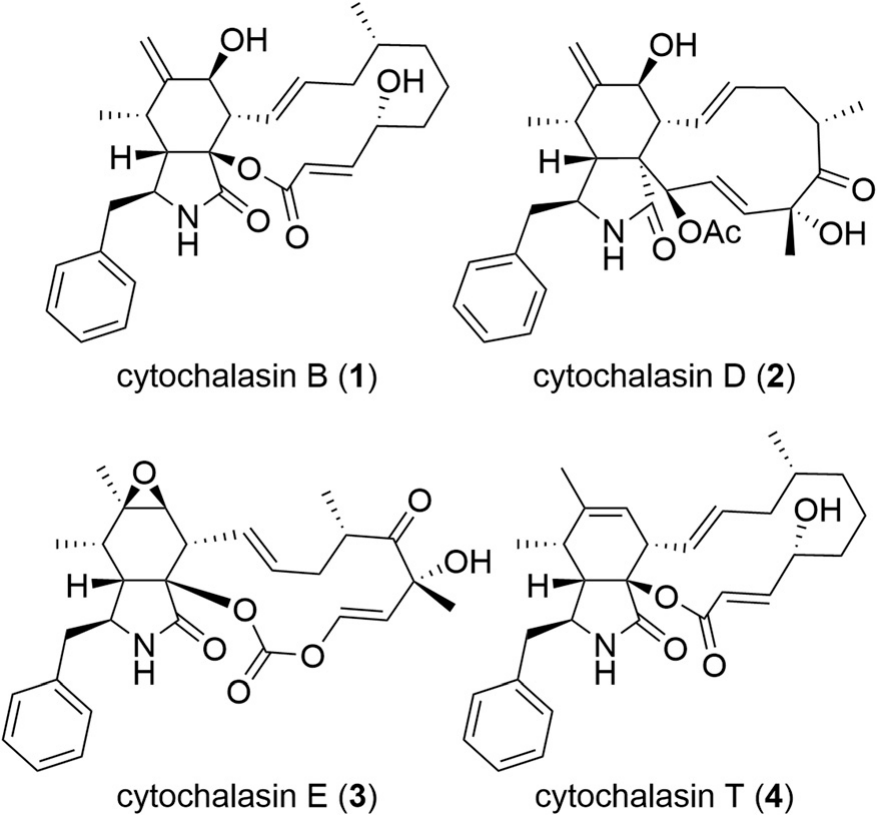
Structures of natural cytochalasins.

Cytochalasans constitute a vast category of natural compounds synthesized by fungi (Fig. 1).^7,8^ The most important characteristic of cytochalasans is their binding to the barbed end of actin microfilaments, resulting in the inhibition of actin polymerization.^9,10^ Among cytochalasans, cytochalasin B (CytB, 1)^11–14^ and cytochalasin D (CytD, 2)^15–17^ have been subjected to extensive investigation of their mechanism of action and antitumor activity. Besides direct cytotoxicity, both CytB (1) and CytD (2) showed antimetastatic activity.^18–21^ Notably, the cytotoxic and migrastatic effects of cytochalasans manifest at significantly different concentration levels.^22^ Hence, cytochalasans warrant consideration as plausible migrastatics.

Cytochalasans represent polyketide–amino acid hybrids sharing a perhydroisoindolone core which serves as the structural framework for an attached macrocycle (Fig. 1). Their complex structure is given by the presence of the macrocyclic moiety and remains a challenge for total synthesis even five decades after their discovery.^23^

Although over 400 cytochalasans were isolated from natural sources^24^ and the X-ray crystal structure of a complex of CytD (2) with actin is known,^25^ limited knowledge exists regarding their structure–activity relationship (SAR).^26–29^ In general, there are two important factors that contribute to the biological activity: 1) an intact macrocyclic ring that contains several stereogenic centres and double bonds;^30^ and 2) C-7 hydroxy group^30–32^ (such as in CytB, 1, and CytD, 2) or an epoxy group C-6 and C-7 (ref. 32) (such as in cytochalasin E, 3). In general, the oxidized cytochalasans usually show better activities than those lacking any oxygen substitution, such as cytochalasin T (4).^31^

This study outlines the synthesis of cytochalasin analogues featuring a streamlined structure, deliberately omitting the complex macrocyclic moiety. The macrocycle was substituted with simple phenyl groups connected by a 5-atom aliphatic linker, chosen for their anticipated compatibility with the lipophilic pocket of the actin binding site. This study primarily explores the migrastatic activity, cytotoxicity, and the structure–activity relationship of these deliberately simplified cytochalasin analogues.

## Results and discussion

### Chemistry

**Scheme 1:**
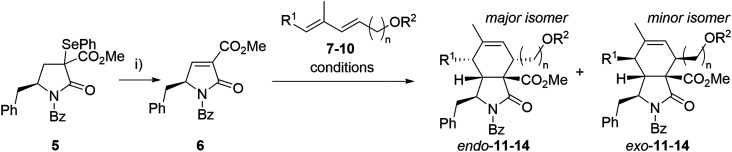
Optimization of the Diels–Alder reaction. Reagents and conditions: i) H_2_O_2_, DCM, 15 °C, 30 min. Reaction conditions for Diels–Alder reactions are given in Table 1.

Our synthesis began with the preparation of partners for the intended Diels–Alder reaction. Selenide 5 was synthesized from *N*-Boc-l-phenylalanine through an optimized seven-step synthesis (a total yield of 60%) following known procedures or analogous methodologies^33–37^ (see ESI† for details, Scheme S1). Benzoylated pyrrolidinone dienophiles are unstable. Therefore, dienophile 6 was synthesized just prior to the Diels–Alder reaction, starting from selenide 5*via* oxidative elimination, and then directly employed in the subsequent step (Scheme 1). As a result, the Diels–Alder reaction yields represent the combined overall yield over two steps: dienophile formation and the subsequent Diels–Alder reaction.

As a model diene, TBS-protected dienol 7 was used. This silylated dienol showed improved stability (from days to weeks) than its unprotected analogue 10. Initial Diels–Alder reactions between dienophile 6 and diene 7 were conducted under thermal conditions (neat, 50 °C, Scheme 1). Expected cycloaddition product 11 was obtained after 14 h in good yield as a mixture of *endo*- and *exo*-adduct (51% yield, *endo*/*exo* = 3.6/1, Table 1, entry 7). No other regioisomers were observed, and the medium yield was mainly attributed to the severe decomposition of 6. Taking into account the slow nature of this Diels–Alder reaction in thermal conditions causing instability of the dienophile, a set of Lewis acid catalysts was evaluated, but with little success (Table 1). The presence of copper catalysts (Cu(OTf)_2_, (CuOTf)_2_·PhMe; entry 1, 2) led to complex reaction mixtures with only small proportions of desired products. Zinc chloride caused almost complete disruption of *endo*/*exo* selectivity (entry 3, 5), while BF_3_·Et_2_O failed to promote the reaction (entry 4). We have observed improved diastereoselectivity (3.6/1 to 5.5/1) with dienol 8 carrying an additional methyl group. After the optimization steps, unprotected dienol 9 was employed in view of the possible shortage of the total synthesis. To our delight, reaction with no catalyst afforded the Diels–Alder product 13 with even better *endo* selectivity and yield (68%, *endo*/*exo* = 6.0/1). These conditions with extended dienol 10 furnished the corresponding cycloadducts 14 in good yield and high selectivity (Scheme 1). Due to the difficult separation of diastereomers, the mixtures of the *endo*- and *exo*-isomers of 13 and 14 were used in the next step.

**Table 1:** Optimization of the Diels–Alder reaction

Entry	Dienol	*n*	R^1^	R^2^	Conditions	Product	Yield^a^ (%)	*Endo*/*exo*^b^
1	7	2	H	TBS	4 Å mol. sieves, DCM/toluene, 0 °C, Cu(OTf)_2_	11	13	1.5/1
2	7	2	H	TBS	4 Å mol. sieves, DCM/toluene, 0 °C, (CuOTf)_2_·PhMe	11	11	1.6/1
3	7	2	H	TBS	4 Å mol. sieves, DCM, 0 °C, ZnCl_2_	11	68	1.3/1
4	7	2	H	TBS	4 Å mol. sieves, DCM/toluene, 0 °C, BF_3_·OEt_2_	11	0	—
5	7	2	H	TBS	DCM, 0 °C, ZnCl_2_	11	35	1.5/1
6	7	2	H	TBS	DCM, 50 °C^c^	11	53	3.4/1
7	7	2	H	TBS	Solvent-free, 50 °C	11	51	3.6/1
8	8	2	Me	TBS	Solvent-free, 50 °C	12	63	5.5/1
9	9	2	Me	H	Solvent-free, 50 °C	13	68	6.0/1
10	10	3	Me	H	Solvent-free, 50 °C	14	61	6.0/1

a Overall isolated yield of all diastereomers over two steps.

b Determined by ^1^H NMR analysis of a crude mixture.

c In a sealed vial.

**Scheme 2:**
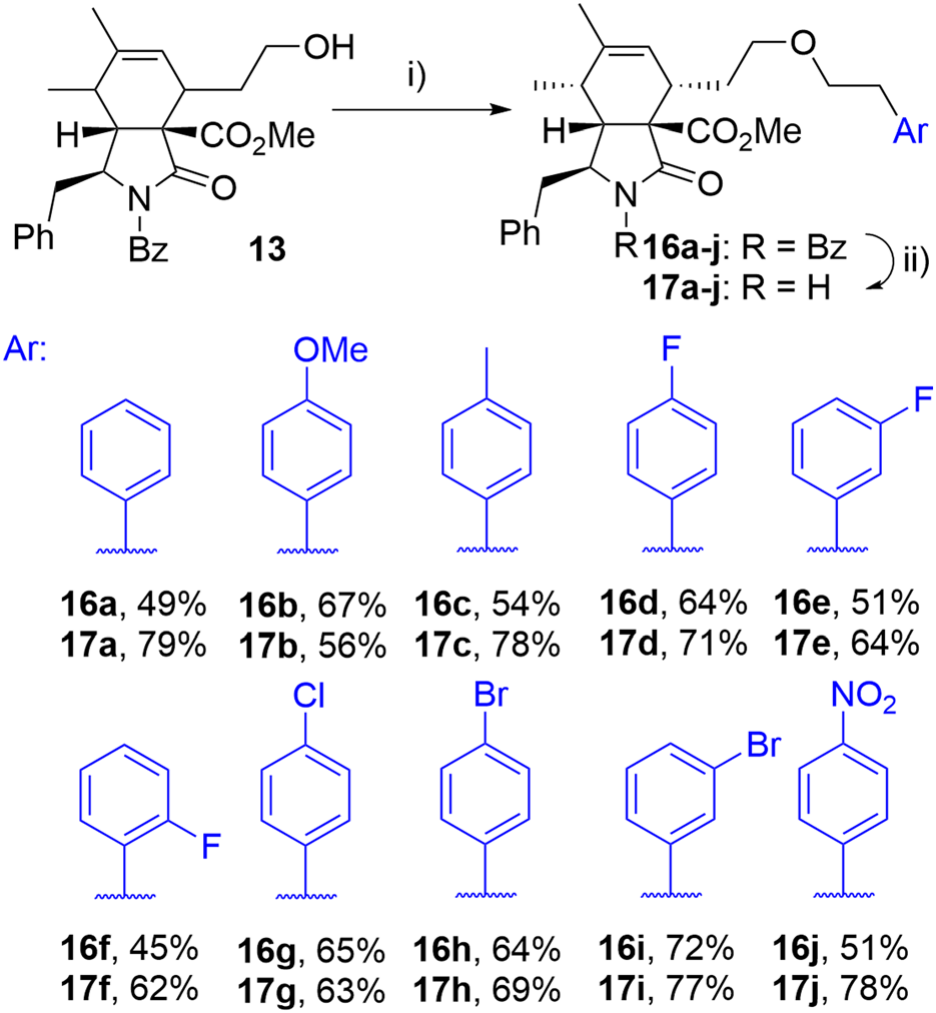
Reagents and conditions: i) ArCH_2_CH_2_I (15a–j), 2,6-di-*t*Bu-pyridine, AgOTf, DCM, 0 °C-RT, 24 h; ii) KOH, toluene/MeOH/H_2_O, RT, 14 h.

The initial attempts to incorporate the phenethyl moiety into the side-chain of cytochalasin analogues were primarily focused on the formation of tosylates or mesylates, followed by subsequent reaction with phenethyl alcohol. These reactions did not yield the desired transformation; in most instances, eliminations or other reactions were observed (data not presented). The successful etherification was achieved by reversing the roles of nucleophile and electrophile. Employing the readily available phenethyl iodide 15a,^38^ which produced the corresponding triflate *in situ* through a displacement reaction with a silver salt,^39^ the corresponding ether derivative 16a was obtained from alcohol 13 in a high yield as a single diastereomer after column chromatography (Scheme 2). This procedure enabled us to prepare a series of derivatives 16a–j with ether-linked aryls substituted by electron-withdrawing and electron-donating groups in different positions. By selective deprotection protocol,^40^ we were able to cleave the benzoyl group getting products 17a–j (Scheme 2).

**Scheme 3:**
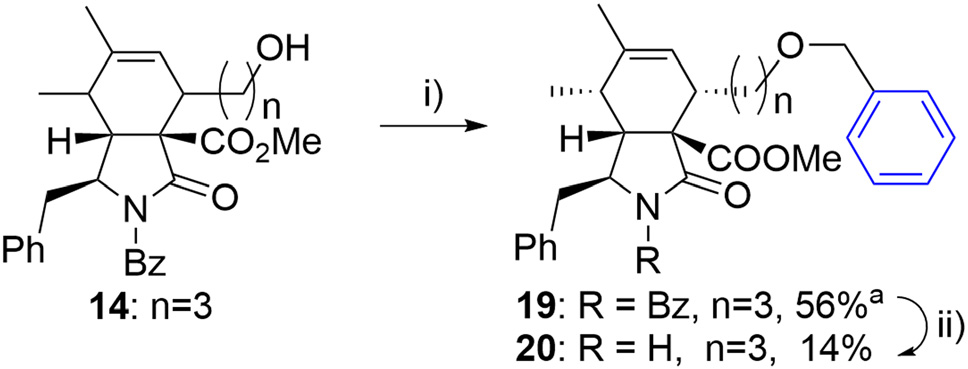
Reagents and conditions: i) PhCH_2_I (18), 2,6-di-*t*Bu-pyridine, AgOTf, DCM, 0 °C-RT, 24 h; ii) KOH, toluene/MeOH/H_2_O, RT, 14 h. ^a^Isolated as a mixture of diastereomers (6/1).

The same approach was employed for the synthesis of an analogue with an oxygen atom at a different position within the linker. However, the reaction of alcohol 14 with benzyl iodide (18) suffered from poor separation of *endo*/*exo* diastereomers. Therefore, the final unprotected derivate 20 was obtained as a single diastereomer in low yield after laborious separation (Scheme 3).

**Scheme 4:**
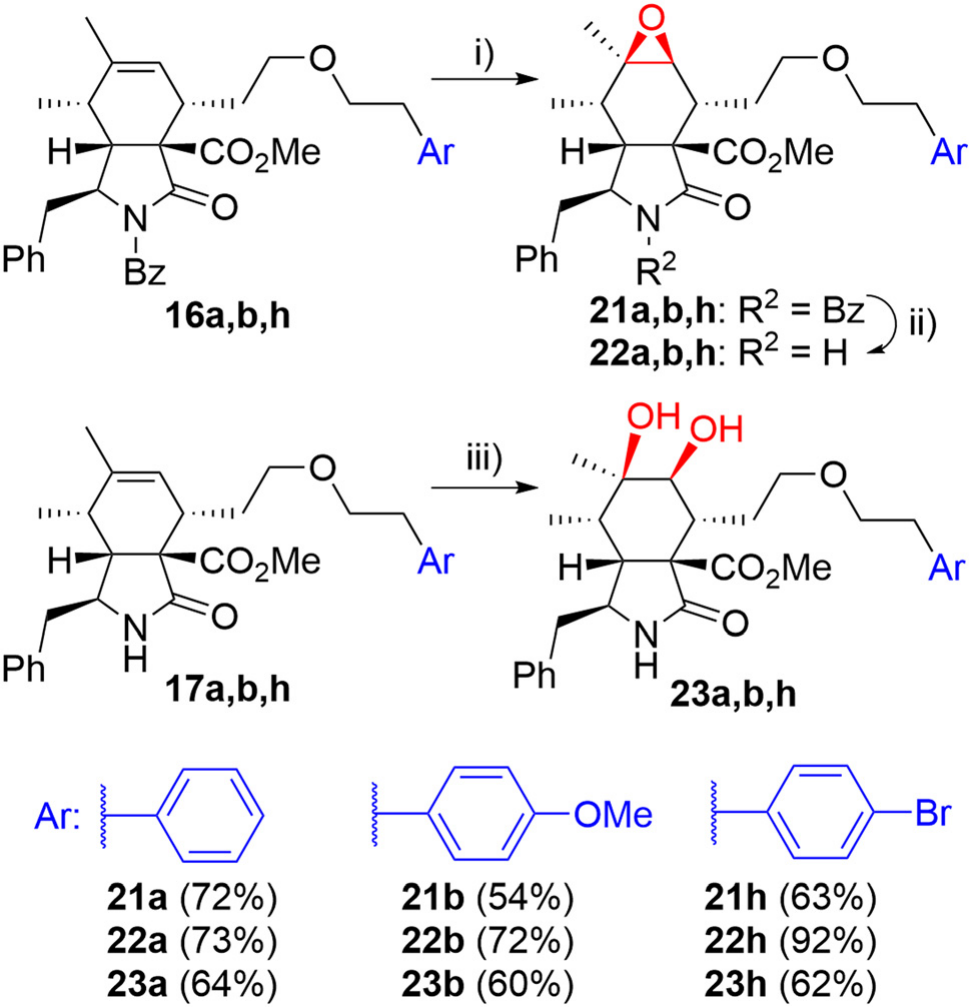
Reagents and conditions: i) mCPBA, DCM, 0 °C-RT, 3 h; ii) KOH, toluene/MeOH/H_2_O, RT, 14 h; iii) K_2_OsO_2_(OH)_4_ (5 mol%), NMO, acetone/H_2_O, RT, 14 h.

Then, we turned our attention to modifications of the cytochalasin core. We focused on the synthesis of epoxides 22 and diols 23 and we were particularly interested in achieving the same substitution pattern of the core as in CytB (1) and CytD (2). Epoxides 22 were prepared by epoxidation of *N*-benzoyl-protected intermediates 16 followed by deprotection (Scheme 4). The epoxidation of 16a using the combination of *t*BuOOH and Mo(CO)_6_ (ref. 41) furnished 22a with hardly separable impurities. Transformation with dimethyldioxirane (DMDO)^42^ provided spot to spot reaction. However, we preferred epoxidation with *m*CPBA^43^ due to an easy experimental setting. A series of three unprotected epoxides 22a,b,h was prepared in acceptable yields (Scheme 4). Corresponding *syn*-diols 23a,b,h were prepared by dihydroxylation of the double bond in unprotected derivatives 17a,b,h by K_2_OsO_2_(OH)_4_ – *N*-methylmorpholine-*N*-oxide (NMO) system in good yields (Scheme 4).

**Scheme 5:**
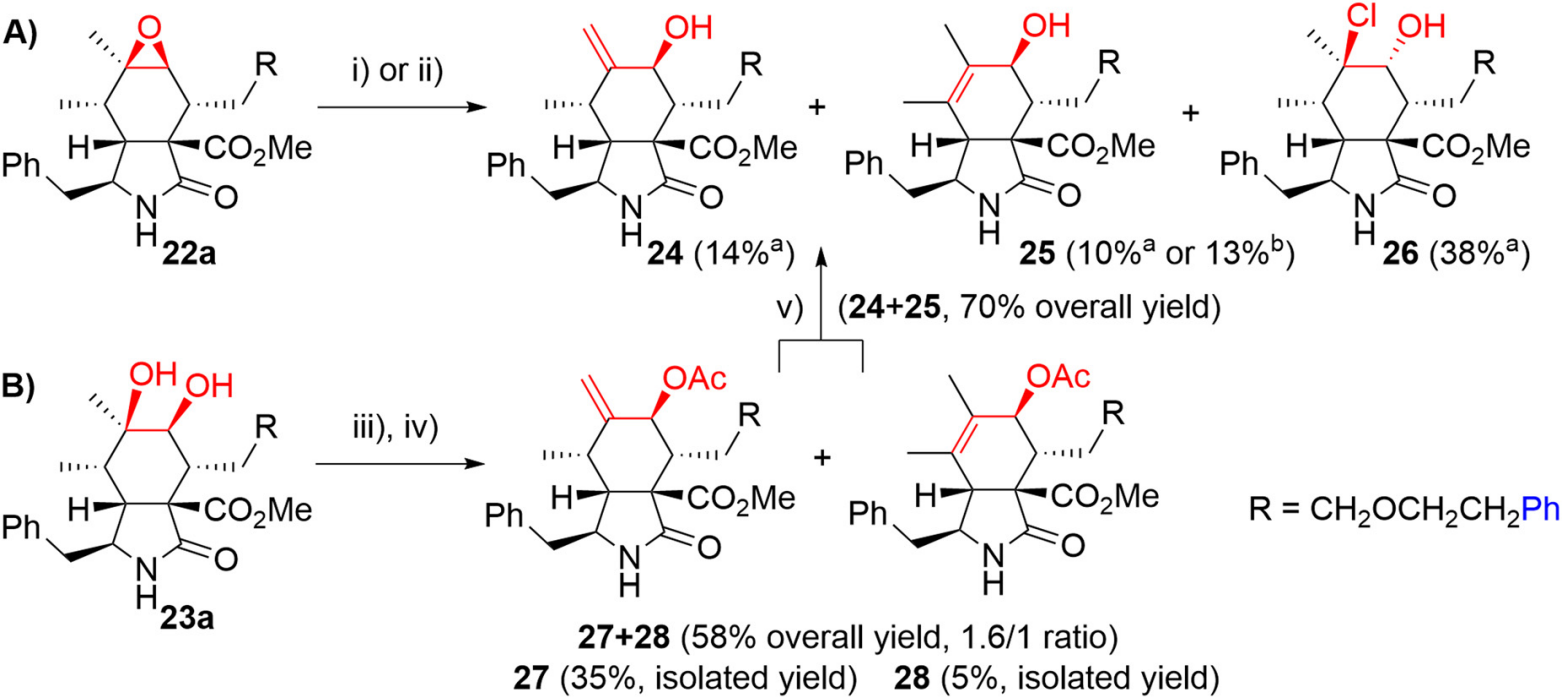
Two synthetic routes to allylic alcohols. Reagents and conditions: i) HCl, Et_2_O, 0 °C, 3 h; ii) DOWEX 50 W H^+^, Et_2_O, RT, 2 days; iii) Ac_2_O, py, RT, 14 h; iv) SOCl_2_, DMAP, py, 0 °C, 1 h; v) KCN, MeOH, RT, 2 days. ^a^Isolated yield, conditions i); ^b^isolated yield, conditions ii).

We explored two synthetic routes to achieve the substitution pattern of CytB (1) and CytD (2), *i.e.* an allylic alcohol with an exocyclic double bond between C6 and C12, and C7-hydroxy group (Scheme 5). First, we focused on rearrangement of the epoxide group to allyl alcohol (route A). Second, a *syn*-diol was subjected to a protection/elimination/deprotection sequence (route B).^44^

In route A, the rearrangement of epoxide 16a with aluminium isopropoxide, which is typically used for this transformation,^41,43^ failed probably due to side reactions with an ester group or an ether bond on the linker. Wet MgSO_4_, previously employed by Myers,^42^ did not even promote any reaction. Subjecting the epoxide to LDA did not lead to the desired reaction. On the other hand, acid-catalyzed reactions seemed to be more reasonable (Scheme 5). Using HCl in Et_2_O, the desired rearrangement occurred furnishing allyl alcohol derivative 24 and its regioisomer 25 having an *endo*-double bond with very similar *R*_f_. Unfortunately, less polar chlorohydrin side-product 26 was also formed. The configuration of compound 26 was determined by employing an ROESY NMR experiment. The proposed mechanism of its formation is not straightforward and includes dehydration/rehydration steps (Scheme S3†). Careful normal-phase HPLC separation afforded chlorohydrin 26 (38%), desired allylic alcohol 24 (14%) and its slightly impure regioisomer 25 (10%). Alternatively, the isomerization of epoxide 22a in the presence of Dowex 50 W in the H^+^-cycle led to the formation of a mixture of regioisomers 24 and 25 (1/3.0 ratio). Pure regioisomer 25 (13%) was obtained by reversed-phase HPLC focused on the isolation of the compound. In order to increase the efficacy of the reaction, several non-nucleophilic acids were screened (see details in ESI,† Scheme S2), unfortunately, with no success.

Because of the poor regioselectivity and the difficult separation of the products, we moved our attention to route B (Scheme 5). Protection of the secondary hydroxyl group in *syn*-diol 23a by the TBS group was attempted without any success. Fortunately, alternative acetylation by Ac_2_O in pyridine proceeded quantitatively. The following dehydration with a mixture of SOCl_2_, DMAP, and pyridine at 0 °C provided the best results (see ESI,† Scheme S4). We got regioisomers 27 and 28 as a mixture (ratio 27/28 = 1.6/1) in 58% overall yield. The mixture was then separated further using HPLC. Major *exo*-isomer 27 was obtained in 35% yield, whereas pure *endo*-derivative 28 only in 5% yield due to problematic separation. Deprotection of acetyl groups by diluted KOH affected also the methyl ester group (partial saponification) and in Zemplén's conditions (cat. EtONa/EtOH), a complex mixture of products difficult to identify was obtained. To our delight, dried KCN in MeOH^45^ provided very mild conditions for smooth cleavage of the acetyl group affording allyl alcohols 24 and 25 (70% combined yield).

**Scheme 6:**
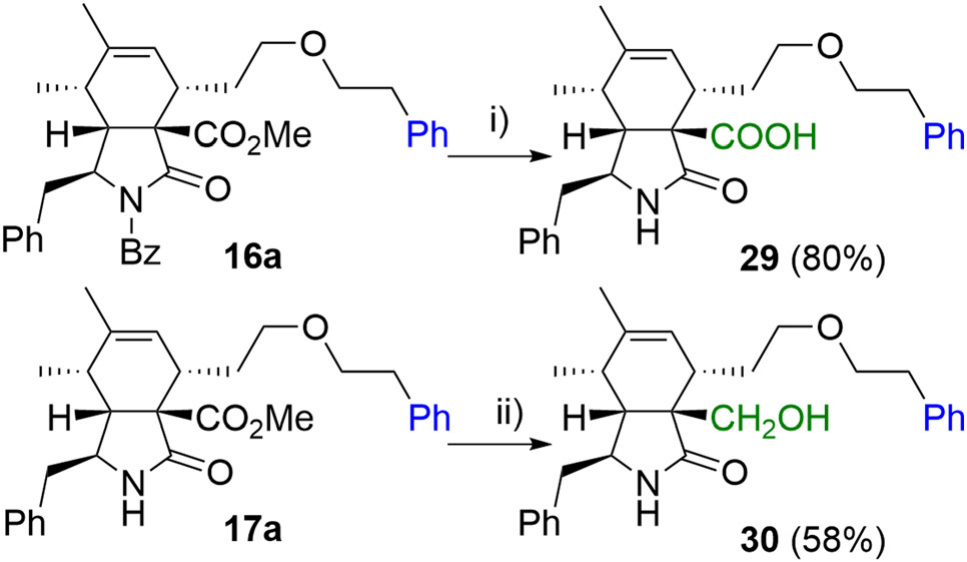
Reagents and conditions: i) NaOH, MeOH/H_2_O, 60 °C, 6 h; ii) LiBH_4_, THF, RT, 2 days.

In the last step, we focused on transformations of the ester group in position 9 (Scheme 6). Standard saponification conditions led to simultaneous cleavage of the base labile *N*-benzoyl group furnishing carboxylic acid derivative 29 in a good yield. Additionally, selective reduction of the ester group was Achieved in the presence of LiBH_4_ giving primary alcohol 30.

### Biological profiling

**Fig. 2:**
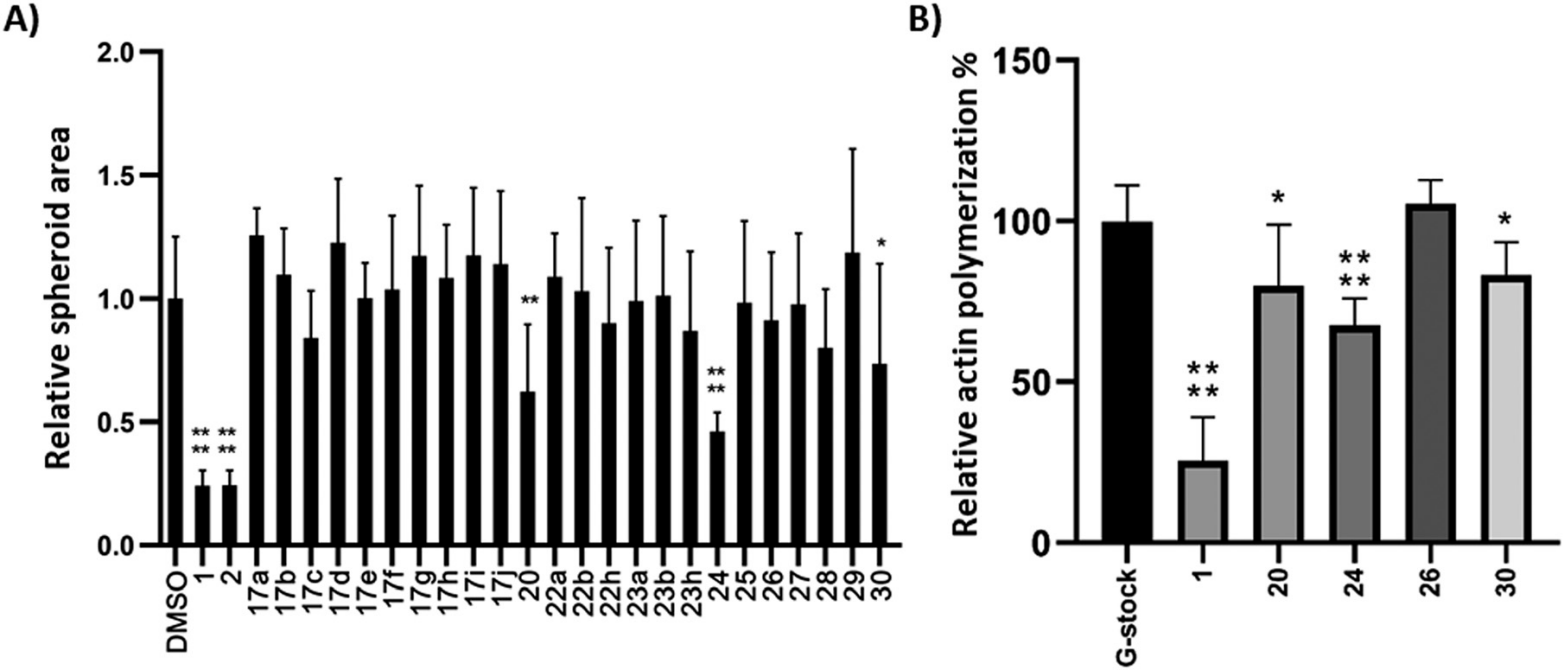
Spheroid invasion assay with BLM cell line (A) at 10 μM concentration, 24 h; actin polymerization assay (B) at 10 μM concentration. Error bars represent standard deviations. *p ≤ 0.05; **p ≤ 0.01; ****p ≤ 0.0001.

Compounds 17, 20, and 22–30 were screened for their migrastatic activity using a spheroid invasion assay with the human melanoma cell line BLM.^46^ Spheroids were formed and subsequently treated with cytochalasin analogues at a concentration of 10 μM for 24 h. CytB (1) and CytD (2) served as reference compounds for comparison. There was no significant increase in cell migration observed for any of the compounds tested. However, a significant decrease in cell migration, as evidenced by a reduction in the relative spheroid area, was observed exclusively with compounds 20, 24, and 30, as depicted in Fig. 2A. However, the effect was less profound than that of the reference compounds 1 and 2.

To further ascertain the compounds' interference with actin polymerization, the active compounds, along with CytB (1) as a positive control and compound 26 as a negative control, were subjected to an *in vitro* actin polymerization assay (Fig. 2B). The inhibition of actin polymerization was evident across all compounds exhibiting migrastatic activity. This correlation between actin inhibitory activity and migrastatic effect underscores the pivotal role of actin polymerization inhibition in mediating the observed migrastatic activity.

**Fig. 3:**
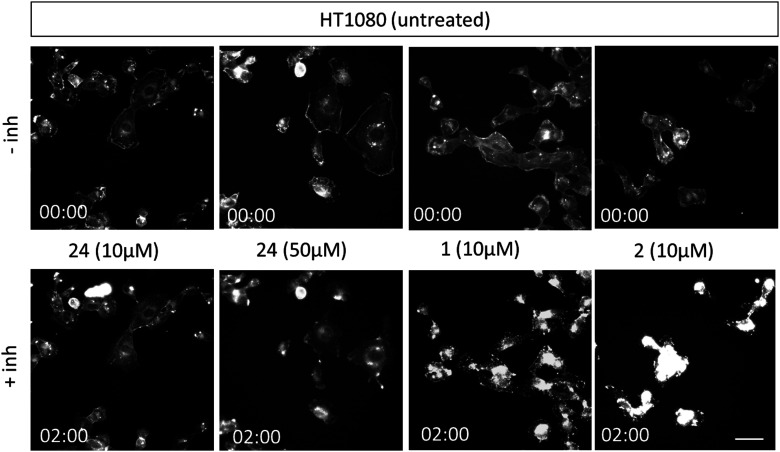
Representative fluorescent images from time/lapse imaging of actin cytoskeleton in untreated (control) and treated cells. Images of HT1080 cells stained with SPY650-FastAct™ were obtained from video recordings (see ESI†) at 0 and 2 min. HT1080 cells were imaged in 12-well glass-bottom plate using Leica TCS SP2 microscope (40×/0.6 dry objective). The scale bar is 150 μm.

Next, we conducted experiments to assess the impact of compound 24, CytB (1) and CytD (2) on the actin cytoskeleton using fluorescence microscopy in HT1080 fibrosarcoma cells (Fig. 3). Both cytochalasins 1 and 2 showed a profound effect on the actin cytoskeleton within 2 min of their addition. In contrast, no effect was observed in cells treated with 10 μM of compound 24 during the initial 2 min period. Nevertheless, observable alterations became apparent after an extended 5 min duration (see ESI† video). Therefore, we increased the concentration up to 50 μM. When using higher concentration, a visible change in cell morphology was observed after 2 min. All of these findings are consistent with the prior discovery, suggesting that compound 24 exerts an effect on the actin cytoskeleton, which is comparable or closely related, albeit less pronounced when compared with the effects induced by cytochalasins 1 and 2.

In addition, the cytotoxic activity of compounds 17, 20, and 22–30 was assessed by the WST-1 viability assay using BLM (human melanoma) and MRC-5 (noncancerous human fibroblasts) cells growing in a monolayer (2D cultures, Table 2) after 72 h of treatment. In general, the cytochalasin analogues exhibited either similar or lower cytotoxicity towards the BLM cell line when compared to the effects of CytB (1) and CytD (2). Notably, diol 23a displayed an IC_50_ of 9.41 μM, signifying an exception to this trend.

**Table 2:** Cytotoxic activity of cytochalasin analogues in human melanoma cells (BLM) and human fibroblasts (MRC-5) with 72 h treatment (measured by WST-1 assay) and selectivity index (SI). The IC_50_ value represents half maximal inhibitory concentration

Compound	IC_50_ [μM]		SI
	BLM	MRC-5	
CytB (1)	26.20 ± 0.55	0.27 ± 0.01	0.01
CytD (2)	13.71 ± 1.20	2.36 ± 0.07	0.17
17a	27.07 ± 1.24	30.50 ± 0.38	1.1
17b	35.34 ± 4.75	40.54 ± 4.65	1.1
17c	30.26 ± 4.87	21.54 ± 2.78	0.71
17d	27.66 ± 2.45	33.53 ± 4.98	1.2
17e	25.91 ± 2.97	31.93 ± 4.02	1.2
17f	21.03 ± 4.32	34.46 ± 4.69	1.6
17g	19.47 ± 1.23	22.17 ± 12.20	1.1
17h	>50	>50	—
17i	24.26 ± 1.82	31.19 ± 6.95	1.3
17j	35.18 ± 2.51	34.70 ± 6.18	1.0
20	28.36 ± 0.59	28.60 ± 1.86	1.0
22a	>50	>50	—
22b	20.62 ± 6.43	>50	>2.4
22h	>50	>50	—
23a	9.41 ± 2.52	20.89 ± 2.38	2.2
23b	>50	>50	—
23h	22.99 ± 2.17	>50	>2.2
24	>50	>50	—
25	>50	>50	—
26	>50	>50	—
27	>50	>50	—
28	>50	>50	—
29	>50	>50	—
30	>50	>50	—

Conversely, the cytochalasin analogues demonstrated significantly reduced cytotoxicity compared to their natural counterparts when tested in non-malignant fibroblasts MRC-5. These analogues generally exhibited only moderate cytotoxicities, with IC_50_ values ranging from 20–40 μM; some of them did not reach the IC_50_ values up to the highest concentration tested (50 μM). While natural cytochalasins 1 and 2 displayed selective cytotoxicity towards the fibroblast cell line, among the cytochalasan analogues, the IC_50_ values were generally similar for both tested cell lines. However, in some cases, a slight selectivity towards BLM cells was observed, except for compound 17c.

The substitution pattern within the cytochalasin core has been recognized as a pivotal structural motif affecting the biological properties of cytochalasin analogues. Unsubstituted derivatives that share the same substitution pattern as cytochalasin T (4), compounds 17, 20, 29, and 30, exhibited moderate cytotoxicities. Among them, only compounds 20 and 30 displayed modest migrastatic activity.

In contrast, epoxides 22 and diols 23, which share the substitution pattern with cytochalasin E (3) and other members of the cytochalasin family,^47^ did not exhibit migrastatic effects. However, some of these compounds displayed cytotoxicity. Conversely, compound 24, which shares the same substitution pattern with CytB (1) and CytD (2), exhibited the most potent migrastatic activity, albeit less pronounced than that of the parent compounds. Notably, it exhibited no cytotoxicity even at the highest concentration tested (IC_50_ > 50 μM). The migrastatic activity of compound 24 underscores the significance of the 7-OH group for this activity, as its acetylated counterpart, compound 27, was inactive. However, it is essential to note that the presence of the 7-OH group alone does not guarantee activity, as is evident from the inactivity of compound 25, the regioisomer of the active compound 24, and diols 23.

These findings suggest that migrastatic activity and cytotoxicity are not necessarily closely linked within the cytochalasin family. It can be speculated that other molecular targets, aside from actin polymerization, may contribute to their cytotoxicity. Conversely, the results indicate that migrastatic and cytotoxic activities can be uncoupled, offering promising prospects for the further development of cytochalasin analogues as migrastatics.

## Experimental section

### General remarks

Chemicals and solvents were used as purchased from commercial suppliers or purified by standard techniques. Dienols 7–10 (ref. 48–51) and iodides 17a–j, and 18 were synthesized using previously published protocols.^52–61^ For thin-layer chromatography (TLC), silica gel plates Merck 60 F_254_ were used. Flash chromatography was performed by using silica gel Silicycle – Siliaflash® P 60 (particle size 40–63 μm, pore diameter 60 Å).

^1^H, ^13^C, ^19^F NMR spectra were measured on FT-NMR spectrometers Bruker Avance III™ HD 400 MHz, or Agilent 400-MR DDR2 or JEOL-ECZL400G (400.0 MHz for ^1^H, 100.6 MHz for ^13^C, 376.0 MHz for ^19^F). The complete assignment of all NMR signals was performed using a combination of H,H-COSY, H,H-ROESY, H,C-HSQC, and H,C-HMBC experiments. Cytochalasin atom numbering was used for the assignment of the NMR signals.^62^ The spectra were recorded in CDCl_3_. It served as an internal standard (*δ*_CDCl_3__ = 7.26 ppm) for ^1^H NMR and (*δ*_CDCl_3__ = 77.0 ppm) for ^13^C NMR, trifluoroacetic acid was used as an external standard for ^19^F NMR. Low- and high-resolution mass spectroscopic data were obtained on LTQ Orbitrap XL (Thermo Fisher Scientific) using ESI at the Laboratory of Mass spectrometry, IOCB Prague. Optical rotations were measured at 25 °C, and [*α*]_D_ values are given in 10^−1^ deg cm^2^ g^−1^. Flash chromatography (FC) was performed using the Büchi Pure C-815 Flash system on CHROMABOND Flash empty cartridges filled with silica gel Silicycle – Siliaflash® P 60 (particle size 40–63 μm, pore diameter 60 Å) or reverse phase (C18) RediSep Rf Gold columns. Purification of some compounds was performed using HPLC Büchi Pure C-850 FlashPrep system on a column packed with 5 μm normal phase (ProntoSIL 60-5-Si 150 × 20 mm, BISCHOFF Chromatography) or a column packed with 5 μm C18 reversed phase (XBridge C18 130 Å 100 × 19 mm, Waters). The purity of all final compounds was determined by clean NMR spectra and by HPLC.

#### General procedure for Diels–Alder reactions

Hydrogen peroxide (0.44 mL, 30% aq.) was added dropwise to a stirred solution of selenide 5 (0.13 mmol) in DCM (0.7 mL) at 15 °C. Then, the reaction mixture was vigorously stirred at 15 °C until full conversion (20–30 min, TLC). Next, a solution of NaHCO_3_ (10 mL) was added and resulting mixture was extracted with DCM (3 × 5 mL). The organics were combined, washed with water (5 mL) and brine (5 mL), dried using MgSO_4_ and concentrated under reduced pressure. The dienophile 6 was used immediately in the next step without analysis due to its instability.

A catalyst (0.015 mmol, 15 mol%) together with 4 Å molecular sieves (15 mg, crushed rods) were placed into a flask (if indicated in reaction conditions), followed by the addition of dienophile 6 in anhydrous solvent (0.6 mL) under argon atmosphere. Then a corresponding diene 7–10 (0.10 mmol) was added and, in case of solvent-free conditions, the reaction mixture was concentrated under reduced pressure to dryness, before being stirred until full conversion (TLC) at an indicated temperature under argon atmosphere. Column chromatography of the residue on silica gel furnished corresponding Diels–Alder products.

##### Methyl (1*S*,3*aR*,7*aR*)-2-benzoyl-1-benzyl-4-(2-((*tert*-butyldimethylsilyl)oxy)ethyl)-6-methyl-3-oxo-1,2,3,4,7,7*a*-hexahydro-3*aH*-isoindole-3*a*-carboxylate (11)

By following the general procedure, the Diels–Alder reaction was carried out with 5 (64 mg, 0.13 mmol) and 7 (23 mg, 0.10 mmol) at 50 °C under solvent-free conditions without molecular sieves or a catalyst. FC (5–7% EtOAc in hexanes) furnished 11 (29 mg, 51%) as a 3.6/1 (*endo*/*exo*) diastereomeric mixture, transparent thick oil. ^1^H NMR (400 MHz, CDCl_3_) *major*: *δ* 7.68 (m, 2H *overlapped*, 2× *o*-Bz), 7.54 (m, 1H, *p*-Bz), 7.44–7.25 (m, 5H, 2× *m*-Bn, *p*-Bn, 2× *m*-Bz), 7.20 (m, 2H *overlapped*, 2× *o*-Bn), 5.46 (m, 1H, H-7), 4.16 (dt, *J*_1_ = 9.0 Hz, *J*_2_ = *J*_3_ = 3.7 Hz, 1H, H-3), 3.76 (s, 3H, COOCH_3_), 3.66–3.53 (m, 2H *overlapped*, H-14a,b), 3.26 (dd, *J*_gem_ = 13.6 Hz, *J*_10b,3_ = 3.2 Hz, 1H, H-10b), 2.84 (m, 1H *overlapped*, H-4), 2.80 (m, 1H, H-10a), 2.67 (m, 1H, H-8), 2.17 (m, 1H, H-5b), 2.01–1.91 (m, 1H *overlapped*, H-13b), 1.75–1.65 (m, 1H *overlapped*, H-13a), 1.72 (m, 3H *overlapped*, CH_3_-12), 1.68 (m, 1H, H-5a), 0.85 (s, 9H *overlapped*, SiC(CH_3_)_3_), −0.02 (m, 6H, Si(CH_3_)_2_) ppm; *minor*: *δ* 7.61 (m, 2H *overlapped*, 2× *o*-Bz), 7.54 (m, 1H *overlapped*, *p*-Bz), 7.44–7.25 (m, 5H *overlapped*, 2× *m*-Bn, *p*-Bn, 2× *m*-Bz), 7.19 (m, 2H *overlapped*, 2× *o*-Bn), 5.54 (m, 1H, H-7), 4.29 (dt, *J*_1_ = 10.0 Hz, *J*_2_ = *J*_3_ = 3.5 Hz, 1H, H-3), 3.81 (s, 3H, COOCH_3_), 3.72–3.60 (m, 2H *overlapped*, H-14a,b), 3.13 (m, 1H *overlapped*, H-10b), 2.95 (m, 1H *overlapped*, H-4), 2.77 (m, 1H, H-10a), 2.73 (m, 1H *overlapped*, H-8), 2.11 (m, 1H, H-5b), 1.89–1.82 (m, 1H *overlapped*, H-13b), 1.79–1.69 (m, 1H *overlapped*, H-13a), 1.73 (m, 1H, H-5a), 1.66 (m, 3H *overlapped*, CH_3_-12), 0.85 (s, 9H *overlapped*, SiC(CH_3_)_3_), −0.01 (m, 6H, Si(CH_3_)_2_) ppm. ^13^C NMR (101 MHz, CDCl_3_) *major*: *δ* 172.76 (*C*OOCH_3_), 172.37 (C-1), 170.61 (C=O–Bz), 136.38 (C–*i*-Bn), 135.62 (C-6), 134.47 (C–*i*-Bz), 132.34 (CH–*p*-Bz), 129.79 (2× CH–*o*-Bn), 129.13 (2× CH–*o*-Bz), 128.62 (2× CH–*m*-Bn), 127.96 (2× CH–*m*-Bz), 126.92 (CH–*p*-Bn), 125.69 (CH-7), 63.37 (CH-3), 61.55 (CH_2_-14), 61.31 (C-9), 52.91 (COO*C*H_3_), 40.98 (CH-4), 38.94 (CH_2_-10), 36.97 (CH-8), 33.64 (CH_2_-5), 33.02 (CH_2_-13), 25.85 (C(*C*H_3_)_3_), 23.57 (CH_3_-12), 18.21 (*C*(CH_3_)_3_), −5.40 (Si(CH_3_)_2_) ppm; *minor*: *δ* 172.25 (C-1), 171.05 (*C*OOCH_3_), 170.76 (C=O–Bz), 136.70 (C–*i*-Bn), 134.43 (C–*i*-Bz), 134.05 (C-6), 132.28 (CH–*p*-Bz), 129.69 (2× CH–*o*-Bn), 129.01 (2× CH–*o*-Bz), 128.66 (2× CH–*m*-Bn), 127.93 (2× CH–*m*-Bz), 126.85 (CH–*p*-Bn), 123.56 (CH-7), 64.04 (CH-3), 61.46 (CH_2_-14), 60.92 (C-9), 52.81 (COO*C*H_3_), 38.06 (CH_2_-10), 37.42 (CH-4), 35.78 (CH-8), 33.11 (CH_2_-5), 32.82 (CH_2_-13), 25.88 (C(*C*H_3_)_3_), 23.36 (CH_3_-12), 17.96 (*C*(CH_3_)_3_), −5.42 (Si(CH_3_)_2_) ppm. HRMS (ESI) *m*/*z* calcd for C_33_H_44_O_5_NSi^+^ [M + H]^+^ 562.2983; found 562.2986.

##### Methyl (1*S*,3*aR*,7*aR*)-2-benzoyl-1-benzyl-4-(2-((*tert*-butyldimethylsilyl)oxy)ethyl)-6,7-dimethyl-3-oxo-1,2,3,4,7,7*a*-hexahydro-3*aH*-isoindole-3*a*-carboxylate (12)

By following the general procedure, the Diels–Alder reaction was carried out with 5 (64 mg, 0.13 mmol) and 8 (24 mg, 0.10 mmol) at 50 °C under solvent-free conditions without molecular sieves or a catalyst. FC (0–10% EtOAc in hexanes) furnished compound 12 (36 mg, 63%) as a 5.5/1 (*endo*/*exo*) diastereomeric mixture, transparent thick oil. ^1^H NMR (400 MHz, CDCl_3_) *major*: *δ* 7.64 (m, 2H *overlapped*, 2× *o*-Bz), 7.52 (m, 1H, *p*-Bz), 7.34–7.25 (m, 5H *overlapped*, 2× *m*-Bn, *p*-Bn, 2× *m*-Bz), 7.15 (m, 2H *overlapped*, 2× *o*-Bn), 5.48 (m, 1H, H-7), 4.30 (dt, *J*_3,10a_ = 8.0 Hz, *J*_3,10b_ = *J*_3,4_ = 3.2 Hz, 1H, H-3), 3.67 (s, 3H, COOCH_3_), 3.66–3.50 (m, 2H *overlapped*, H-14a,b), 3.10 (dd, *J*_gem_ = 13.4 Hz, *J*_10b,3_ = 3.2 Hz, 1H, H-10b), 2.94 (dd, *J*_gem_ = 13.4 Hz, *J*_10a,3_ = 8.0 Hz, 1H, H-10a), 2.74 (dd, *J*_4,5_ = 5.7 Hz, *J*_4,3_ = 3.2 Hz, 1H, H-4), 2.65 (m, 1H, H-8), 2.43 (m, 1H, H-5), 2.06 (m, 1H *overlapped*, H-13b), 1.73 (m, 3H, CH_3_-12), 1.68 (m, 1H *overlapped*, H-13a), 0.86 (d, *J*_11,5_ = 6.9 Hz, 3H, CH_3_-11), 0.85 (s, 9H *overlapped*, SiC(CH_3_)_3_), −0.03 (m, 6H, Si(CH_3_)_2_) ppm; *minor*: *δ* 7.64 (m, 2H *overlapped*, 2× *o*-Bz), 7.52 (m, 1H, *p*-Bz), 7.34–7.25 (m, 5H *overlapped*, 2× *m*-Bn, *p*-Bn, 2× *m*-Bz), 7.15 (m, 2H *overlapped*, 2× *o*-Bn), 5.53 (m, 1H, H-7), 4.35 (dt, *J*_3,10a_ = 8.0 Hz, *J*_3,10b_ = *J*_3,4_ = 3.3 Hz, 1H, H-3), 3.85 (s, 3H, COOCH_3_), 3.66–3.50 (m, 2H *overlapped*, H-14a,b), 3.20 (dd, *J*_gem_ = 13.0 Hz, *J*_10b,3_ = 3.3 Hz, 1H, H-10b), 2.88 (dd, *J*_gem_ = 13.0 Hz, *J*_10a,3_ = 8.0 Hz, 1H, H-10a), 2.75 (m, 1H *overlapped*, H-8), 2.47 (m, 1H, H-4), 2.31 (m, 1H *overlapped*, H-13b), 1.82 (m, 1H *overlapped*, H-13a), 1.80 (m, 1H *overlapped*, H-5), 1.62 (m, 3H, CH_3_-12), 0.85 (s, 9H *overlapped*, SiC(CH_3_)_3_), 0.72 (d, *J*_11,5_ = 7.1 Hz, 3H, CH_3_-11), −0.02 (m, 6H, Si(CH_3_)_2_) ppm. ^13^C NMR (101 MHz, CDCl_3_) *major*: *δ* 172.51 (COOCH_3_), 172.49 (C-1), 170.33 (C=O–Bz), 139.84 (C-6), 136.12 (C–*i*-Bn), 134.49 (C–*i*-Bz), 132.15 (CH–*p*-Bz), 130.46 (2× CH–*o*-Bn), 128.99 (2× CH–*o*-Bz), 128.44 (2× CH–*m*-Bn *overlapped*), 127.91 (2× CH–*m*-Bz *overlapped*), 126.99 (CH), 126.92 (CH), 61.82 (CH_2_-14), 61.39 (C-9), 57.29 (CH-3), 52.80 (COOCH_3_), 47.17 (CH-4), 39.94 (CH_2_-10), 37.86 (CH-8), 34.21 (CH-5), 32.82 (CH_2_-13), 25.85 (C(*C*H_3_)_3_), 19.69 (CH_3_-12), 18.19 (*C*(CH_3_)_3_), 12.99 (CH_3_-11), −5.42 (Si(CH_3_)_2_) ppm; *minor*: *δ* 172.68 (COOCH_3_), 172.44 (C-1), 170.27 (C=O–Bz), 140.64 (C-6), 136.02 (C–*i*-Bn), 134.43 (C–*i*-Bz), 132.22 (CH–*p*-Bz), 130.53 (2× CH–*o*-Bn), 128.97 (2× CH–*o*-Bz), 128.44 (2× CH–*m*-Bn *overlapped*), 127.91 (2× CH–*m*-Bz *overlapped*), 126.75 (CH), 126.51 (CH), 61.50 (CH_2_-14), 60.75 (C-9), 57.59 (CH-3), 52.77 (COOCH_3_), 44.27 (CH-4), 39.84 (CH_2_-10), 36.90 (CH-8), 36.47 (CH-5), 33.40 (CH_2_-13), 25.90 (C(*C*H_3_)_3_), 20.91 (CH_3_-12), 17.95 (*C*(CH_3_)_3_), 16.67 (CH_3_-11), −5.35 (Si(CH_3_)_2_) ppm. HRMS (ESI) *m*/*z* calcd for C_34_H_45_O_5_NNaSi^+^ [M + Na]^+^ 598.2959; found 598.2956.

##### Methyl (1*S*,3*aR*,7*aR*)-2-benzoyl-1-benzyl-4-(2-hydroxyethyl)-6,7-dimethyl-3-oxo-1,2,3,4,7,7*a*-hexahydro-3*aH*-isoindole-3*a*-carboxylate (13)

By following the general procedure, the Diels–Alder reaction was carried out with selenide 5 (64 mg, 0.13 mmol) and dienol 9 (13 mg, 0.10 mmol) at 50 °C under solvent-free conditions without molecular sieves or a catalyst. FC (10–60% EtOAc in hexanes) furnished compound 13 (31 mg, 68%) as a 6.0/1 (*endo*/*exo*) diastereomeric mixture, transparent density oil. A small quantity of pure *endo*-product was isolated from the mixture for measurement of optical activity. [*α*]_D_ = +33.5° (c 0.26; CHCl_3_). ^1^H NMR (400 MHz, CDCl_3_) *major*: *δ* 7.62 (m, 2H, 2× *o*-Bz), 7.53 (m, 1H, *p*-Bz), 7.42 (m, 2H, 2× *m*-Bz), 7.34–7.24 (m, 3H *overlapped*, 2× *m*-Bn, *p*-Bn), 7.15 (m, 2H, 2× *o*-Bn), 5.45 (m, 1H *overlapped*, H-7), 4.10 (dt, *J*_3,10a_ = 7.8 Hz, *J*_3,4_ = *J*_3,10b_ = 3.4 Hz, 1H, H-3), 3.66 (s, 3H, COOCH_3_), 3.69–3.53 (m, 2H *overlapped*, H-14a,b), 3.12 (dd, *J*_gem_ = 13.4 Hz, *J*_10b,3_ = 3.4 Hz, 1H, H-10b), 2.99 (dd, *J*_gem_ = 13.4 Hz, *J*_10a,3_ = 7.8 Hz, 1H, H-10a), 2.72 (dd, *J*_4,5_ = 5.5 Hz, *J*_4,3_ = 3.4 Hz, 1H, H-4), 2.75–2.71 (m, 1H *overlapped*, H-8), 2.46 (m, 1H, H-5), 2.09–2.00 (m, 1H *overlapped*, H-13b), 1.86–1.78 (m, 1H, H-13a), 1.75 (m, 3H, CH_3_-12), 0.86 (d, *J*_11,5_ = 7.3 Hz, 3H, CH_3_-11) ppm (*H from OH is missing*); *minor*: *δ* 7.68 (m, 2H, 2× *o*-Bz), 7.54 (m, 1H *overlapped*, *p*-Bz), 7.44 (m, 2H *overlapped*, 2× *m*-Bz), 7.35–7.25 (m, 5H *overlapped*, Bn), 5.45 (m, 1H *overlapped*, H-7), 4.46 (ddd, *J*_3,10a_ = 10.1 Hz, *J*_3,10b_ = 3.5 Hz, *J*_3,4_ = 2.5 Hz, 1H, H-3), 3.84 (s, 3H, COOCH_3_), 3.69–3.53 (m, 2H *overlapped*, H-14a,b), 3.20 (dd, *J*_gem_ = 13.1 Hz, *J*_10b,3_ = 3.5 Hz, 1H, H-10b), 2.71 (m, 1H *overlapped*, H-10a), 2.62 (m, 1H, H-5), 2.37 (dd, *J*_4,5_ = 7.8 Hz, *J*_4,3_ = 2.5 Hz, 1H, H-4), 1.96–1.88 (m, 1H, H-13b), 1.82 (m, 1H, H-8), 1.64 (m, 3H, CH_3_-12), 1.62–1.55 (m, 1H, H-13a), 0.71 (d, *J*_11,5_ = 7.1 Hz, 3H, CH_3_-11) ppm (*H from OH is missing*). ^13^C NMR (101 MHz, CDCl_3_) *major*: *δ* 172.68 (*C*OOCH_3_), 172.49 (C-1), 170.27 (C=O–Bz), 140.64 (C-6), 136.01 (C–*i*-Bn), 134.42 (C–*i*-Bz), 132.24 (CH–*p*-Bz), 130.55 (2× CH–*o*-Bn), 128.97 (2× CH–*o*-Bz), 128.45 (2× CH–*m*-Bn), 127.93 (2× CH–*m*-Bz), 126.98 (CH–*p*-Bn), 126.51 (CH-7), 61.52 (CH_2_-14), 60.80 (C-9), 57.58 (CH-3), 53.00 (COO*C*H_3_), 47.34 (CH-4), 39.81 (CH_2_-10), 37.39 (CH-8), 34.29 (CH-5), 33.18 (CH_2_-13), 19.84 (CH_3_-12), 13.08 (CH_3_-11) ppm; *minor*: *δ* 173.07 (C-1), 171.81 (*C*OOCH_3_), 170.56 (C=O–Bz), 138.17 (C-6), 136.56 (C–*i*-Bn), 134.35 (C–*i*-Bz), 132.43 (CH–*p*-Bz), 129.84 (2× CH–*o*-Bn), 129.01 (2× CH–*o*-Bz), 128.58 (2× CH–*m*-Bn), 128.01 (2× CH–*m*-Bz), 126.96 (CH–*p*-Bn), 124.04 (CH-7), 62.55 (CH_2_-14), 62.52 (CH-3), 60.55 (C-9), 52.79 (COO*C*H_3_), 45.94 (CH-4), 38.41 (CH_2_-10), 36.48 (CH-8), 34.96 (CH_2_-13), 34.17 (CH-5), 20.93 (CH_3_-12), 16.67 (CH_3_-11) ppm. HRMS (ESI) *m*/*z* calcd for C_28_H_32_O_5_N^+^ [M + H]^+^ 462.2275; found 462.2274.

##### Methyl (1*S*,3*aR*,7*aR*)-2-benzoyl-1-benzyl-4-(3-hydroxypropyl)-6,7-dimethyl-3-oxo-1,2,3,4,7,7*a*-hexahydro-3*aH*-isoindole-3*a*-carboxylate (14)

Aqueous hydrogen peroxide (30%, 246 μL, 2.41 mmol) was added dropwise to a stirred solution of 5 (600 mg, 1.21 mmol) in DCM (10 mL) at 0 °C. Then, the reaction mixture was vigorously stirred at 0 °C for 30 min and then at RT until full conversion was observed (30 min, TLC). Next, a solution of NaHCO_3_ (10 mL) was added and the resulting mixture was extracted with DCM (3 × 5 mL). The organics were combined, dried over Na_2_SO_4_ and concentrated under reduced pressure to approx. 1 mL. Diene 10 (135 mg, 0.96 mmol) was added and residual DCM was evaporated off. The mixture was stirred at 50 °C for 21 h under argon atmosphere. Column chromatography of the mixture on silica gel (80 g) furnished 14 (277 mg, 61%) as a 6.0/1 (*endo*/*exo*) diastereomeric mixture, white amorphous solid. ^1^H NMR (400 MHz, CDCl_3_) *δ* 7.64–7.58 (m, 2H, 2× *o*-Bz), 7.55–7.49 (m, 1H, *p*-Bz), 7.47–7.39 (m, 2H, 2× *m*-Bz), 7.35–7.22 (m, 3H, 2× *m*-Bn, *p*-Bn), 7.17–7.11 (m, 2H, 2× *o*-Bn), 5.45 (bs, 1H, H-7), 4.28 (dt, *J*_3,10a_ = 8.0 Hz, *J*_3,4_ = *J*_3,10b_ = 3.4 Hz, 1H, H-3), 3.66 (s, 3H, COOCH_3_), 3.58 (t, *J*_15,14_ = 6.4 Hz, 2H, H-15), 3.10 (dd, *J*_gem_ = 13.4 Hz, *J*_10b,3_ = 3.3 Hz, 1H, H-10b), 2.97 (dd, *J*_gem_ = 13.5 Hz, *J*_10a,3_ = 8.0 Hz, 1H, H-10a), 2.71 (dd, *J*_4,5_ = 5.6 Hz, *J*_4,3_ = 3.4 Hz, 1H, H-4), 2.55–2.47 (m, 1H, H-8), 2.47–2.41 (m, 1H, H-5), 1.93–1.81 (m, 1H, H-13b), 1.74 (s, 3H, CH_3_-12), 1.72–1.59 (m, 1H *overlapped*, H-14b), 1.57–1.50 (m, 1H, H-13a), 1.49–1.39 (m, 1H, H-14a), 0.86 (d, *J*_11,5_ = 7.3 Hz, 3H, CH_3_-11) ppm. ^13^C NMR (101 MHz, CDCl_3_) *δ* 172.76 (C-1), 172.42 (*C*OOCH_3_), 170.29 (C=O–Bz), 140.49 (C-6), 136.01 (C–*i*-Bn), 134.40 (C–*i*-Bz), 132.23 (CH–*p*-Bz), 130.52 (2× CH–*o*-Bn), 128.98 (2× CH–*o*-Bz), 128.44 (2× CH–*m*-Bn), 127.93 (2× CH–*m*-Bz), 126.96 (CH–*p*-Bn), 126.68 (CH-7), 63.00 (C-9), 62.47 (CH_2_-15), 57.43 (CH-3), 52.99 (COO*C*H_3_), 47.24 (CH-4), 40.68 (CH-8), 39.76 (CH_2_-10), 34.25 (CH-5), 31.85 (CH_2_-14), 25.83 (CH_2_-13), 19.80 (CH_3_-12), 13.03 (CH_3_-11) ppm. NMR spectra of the minor isomer were not assigned due to overlapping signals. HRMS (ESI) *m*/*z*: [M + H]^+^ calcd for C_29_H_34_O_5_N^+^ 476.2432; found 476.2429. MS (ESI) *m*/*z* (%): 476 (22) [M + H], 498 (100) [M + Na], 973 (17) [2M + Na].

#### General procedure for etherifications

Following a slightly modified procedure,^39^ AgOTf (193 mg, 0.75 mmol) was placed to a flask and dried under high vacuum by means of a heatgun (150–200 °C). Then, 2,6-di-*tert*-butylpyridine (168 mg, 0.88 mmol) followed by a solution of compounds 13 (115 mg, 0.25 mmol) in dry DCM (0.5 mL) were added at 0 °C under argon atmosphere. The corresponding iodide 15 (0.80 mmol) was added and the resulting yellow suspension was stirred at room temperature until full conversion (4–20 h, TLC). The reaction mixture was concentrated under reduced pressure, then diluted with Et_2_O (10 mL) and filtered through a small pad of celite. The organic layer was washed with 1 M HCl (5 mL), a solution of NaHCO_3_ (5 mL), brine (5 mL), dried (Na_2_SO_4_) and concentrated under reduced pressure. Column chromatography of the residue on silica gel (15% EtOAc in hexanes) furnished compounds 16 as single diastereomers. In several cases 2-arylethanols originating from corresponding iodides were present as impurities.

##### Methyl (1*S*,3*aR*,4*S*,7*S*,7*aR*)-2-benzoyl-1-benzyl-6,7-dimethyl-3-oxo-4-(2-phenethoxyethyl)-1,2,3,4,7,7*a*-hexahydro-3*aH*-isoindole-3*a*-carboxylate (16a)

By following the general procedure for etherifications, the reaction was carried out with iodide 15a (186 mg, 0.80 mmol). FC furnished compound 16a (69 mg, 49%), transparent oil, and a small amount of phenethyl alcohol as an impurity. [*α*]_D_ = +120.6° (c 0.22; CHCl_3_). ^1^H NMR (400 MHz, CDCl_3_) *δ* 7.63 (m, 2H, 2× *o*-Bz), 7.53 (m, 1H, *p*-Bz), 7.43 (m, 2H, 2× *m*-Bz), 7.35–7.13 (m, 10H, Ph, Bn), 5.46 (m, 1H, H-7), 4.29 (dt, *J*_3,10a_ = 8.1 Hz, *J*_3,4_ = *J*_3,10b_ = 3.3 Hz, 1H, H-3), 3.68 (s, 3H, COOCH_3_), 3.61 (dt, *J*_gem_ = 9.2 Hz, *J*_15b,16a_ = *J*_15b,16b_ = 7.1 Hz, 1H, H-15b), 3.51 (dt, *J*_gem_ = 9.2 Hz, *J*_15a,16a_ = *J*_15a,16b_ = 7.1 Hz, 1H, H-15a), 3.49–3.37 (m, 2H, H-14a,b), 3.13 (dd, *J*_gem_ = 13.4 Hz, *J*_10b,3_ = 3.3 Hz, 1H, H-10b), 2.95 (dd, *J*_gem_ = 13.4 Hz, *J*_10a,3_ = 8.1 Hz, 1H, H-10a), 2.86–2.79 (m, 2H, H-16a,b), 2.73 (dd, *J*_4,5_ = 5.8 Hz, *J*_4,3_ = 3.3 Hz, 1H, H-4), 2.65 (m, 1H, H-8), 2.42 (m, 1H, H-5), 2.15–2.04 (m, 1H, H-13b), 1.85–1.74 (m, 1H, H-13a), 1.73 (m, 3H, CH_3_-12), 0.85 (d, *J*_11,5_ = 7.3 Hz, 3H, CH_3_-11) ppm. ^13^C NMR (101 MHz, CDCl_3_) *δ* 172.45 (2C *overlapped*, *C*OOCH_3_, C-1), 170.25 (C=O–Bz), 140.09 (C-6), 139.14 (C–*i*-Ph), 136.04 (C–*i*-Bn), 134.41 (C–*i*-Bz), 132.13 (CH–*p*-Bz), 130.43 (2× CH–*o*-Bn), 128.92 (2× CH–*o*-Bz), 128.87 (2× CH–*o*-Ph), 128.40 (2× CH–*m*-Bn), 128.13 (2× CH–*m*-Ph), 127.88 (2× CH–*m*-Bz), 126.91 (CH–*p*-Bn), 126.53 (CH-7), 125.94 (CH–*p*-Ph), 71.31 (CH_2_-15), 69.39 (CH_2_-14), 62.87 (C-9), 57.31 (CH-3), 52.84 (COO*C*H_3_), 47.13 (CH-4), 39.83 (CH_2_-10), 37.91 (CH-8), 36.23 (CH_2_-16), 34.13 (CH-5), 29.48 (CH_2_-13), 19.76 (CH_3_-12), 12.92 (CH_3_-11) ppm. HRMS (ESI) *m*/*z* calcd for C_36_H_40_O_5_N^+^ [M + H]^+^ 566.2901, found = 566.2900.

##### Methyl (1*S*,3*aR*,4*S*,7*S*,7*aR*)-2-benzoyl-1-benzyl-4-(2-(4-methoxyphenethoxy)ethyl)-6,7-dimethyl-3-oxo-1,2,3,4,7,7*a*-hexahydro-3*aH*-isoindole-3*a*-carboxylate (16b)

By following the general procedure for etherifications, the reaction was carried out with iodide 15b (210 mg, 0.80 mmol). FC furnished compound 16b (95 mg, 64%), transparent oil; [*α*]_D_ = +76.9° (c 0.29; CHCl_3_). ^1^H NMR (400 MHz, CDCl_3_) *δ* 7.62 (m, 2H, 2× *o*-Bz), 7.52 (m, 1H, *p*-Bz), 7.42 (m, 2H, 2× *m*-Bz), 7.35–7.23 (m, 3H, 2× *m*-Bn, *p*-Bn), 7.15 (m, 2H, 2× *o*-Bn), 7.10 (m, 2H, 2× *o*-Ph), 6.79 (m, 2H, 2× *m*-Ph), 5.45 (m, 1H, H-7), 4.29 (dt, *J*_3,10a_ = 8.1 Hz, *J*_3,4_ = *J*_3,10b_ = 3.3 Hz, 1H, H-3), 3.76 (s, 3H, OCH_3_), 3.68 (s, 3H, COOCH_3_), 3.56 (dt, *J*_gem_ = 9.2 Hz, *J*_15b,16a_ = *J*_15b,16b_ = 7.0 Hz, 1H, H-15b), 3.46 (dt, *J*_gem_ = 9.2 Hz, *J*_15a,16a_ = *J*_15a,16b_ = 7.0 Hz, 1H, H-15a), 3.46–3.36 (m, 2H, H-14a,b), 3.12 (dd, *J*_gem_ = 13.4 Hz, *J*_10b,3_ = 3.3 Hz, 1H, H-10b), 2.94 (dd, *J*_gem_ = 13.4 Hz, *J*_10a,3_ = 8.1 Hz, 1H, H-10a), 2.79–2.73 (m, 2H, H-16a,b), 2.72 (dd, *J*_4,5_ = 5.6 Hz, *J*_4,3_ = 3.3 Hz, 1H, H-4), 2.64 (m, 1H, H-8), 2.42 (m, 1H, H-5), 2.14–2.02 (m, 1H, H-13b), 1.84–1.73 (m, 1H, H-13a), 1.73 (m, 3H, CH_3_-12), 0.84 (d, *J*_11,5_ = 7.3 Hz, 3H, CH_3_-11) ppm. ^13^C NMR (101 MHz, CDCl_3_) *δ* 172.52 (*C*OOCH_3_), 172.45 (C-1), 170.30 (C=O–Bz), 157.93 (C–*p*-Ph), 140.12 (C-6), 136.15 (C–*i*-Bn), 134.51 (C–*i*-Bz), 132.15 (CH–*p*-Bz), 131.30 (C–*i*-Ph), 130.46 (2× CH–*o*-Bn), 129.83 (2× CH–*o*-Ph), 128.95 (2× CH–*o*-Bz), 128.46 (2× CH–*m*-Bn), 127.92 (2× CH–*m*-Bz), 126.95 (CH–*p*-Bn), 126.64 (CH-7), 113.62 (2× CH–*m*-Ph), 71.62 (CH_2_-15), 69.45 (CH_2_-14), 62.95 (C-9), 57.37 (CH-3), 55.19 (OCH_3_), 52.86 (COO*C*H_3_), 47.24 (CH-4), 39.97 (CH_2_-10), 38.01 (CH-8), 35.37 (CH_2_-16), 34.19 (CH-5), 29.56 (CH_2_-13), 19.76 (CH_3_-12), 12.96 (CH_3_-11) ppm. HRMS (ESI) *m*/*z* calcd for C_37_H_42_O_6_N^+^ [M + H]^+^ 596.3007, found 596.3007.

##### Methyl (1*S*,3*aR*,4*S*,7*S*,7*aR*)-2-benzoyl-1-benzyl-6,7-dimethyl-4-(2-(4-methylphenethoxy)ethyl)-3-oxo-1,2,3,4,7,7*a*-hexahydro-3*aH*-isoindole-3*a*-carboxylate (16c)

By following the general procedure for etherifications, the reaction was carried out with iodide 15c (197 mg, 0.80 mmol). FC furnished compound 16c (78 mg, 54%), transparent oil; [*α*]_D_ = +80.7° (c 0.22; CHCl_3_). ^1^H NMR (400 MHz, CDCl_3_) *δ* 7.63 (m, 2H, 2× *o*-Bz), 7.52 (m, 1H, *p*-Bz), 7.42 (m, 2H, 2× *m*-Bz), 7.35–7.24 (m, 3H, 2× *m*-Bn, *p*-Bn), 7.16 (m, 2H, 2× *o*-Bn), 7.10–7.03 (m, 4H, 2× *o*-Ph, 2× *m*-Ph), 5.46 (m, 1H, H-7), 4.30 (dt, *J*_3,10a_ = 8.2 Hz, *J*_3,4_ = *J*_3,10b_ = 3.3 Hz, 1H, H-3), 3.69 (s, 3H, COOCH_3_), 3.57 (dt, *J*_gem_ = 9.2 Hz, *J*_15b,16a_ = *J*_15b,16b_ = 7.1 Hz, 1H, H-15b), 3.47 (dt, *J*_gem_ = 9.2 Hz, *J*_15a,16a_ = *J*_15a,16b_ = 7.1 Hz, 1H, H-15a), 3.46–3.37 (m, 2H, H-14a,b), 3.13 (dd, *J*_gem_ = 13.4 Hz, *J*_10b,3_ = 3.3 Hz, 1H, H-10b), 2.93 (dd, *J*_gem_ = 13.4 Hz, *J*_10a,3_ = 8.2 Hz, 1H, H-10a), 2.81–2.75 (m, 2H, H-16a,b), 2.73 (dd, *J*_4,5_ = 5.6 Hz, *J*_4,3_ = 3.3 Hz, 1H, H-4), 2.64 (m, 1H, H-8), 2.42 (m, 1H, H-5), 2.30 (s, 3H, PhCH_3_), 2.15–2.03 (m, 1H, H-13b), 1.84–1.74 (m, 1H, H-13a), 1.73 (m, 3H, CH_3_-12), 0.84 (d, *J*_11,5_ = 7.3 Hz, 3H, CH_3_-11) ppm. ^13^C NMR (101 MHz, CDCl_3_) *δ* 172.52 (*C*OOCH_3_), 172.45 (C-1), 170.30 (C=O–Bz), 140.11 (C-6), 136.15 (C–*i*-Bn), 136.07 (C–*i*-Ph), 135.43 (C–*p*-Ph), 134.51 (C–*i*-Bz), 132.14 (CH–*p*-Bz), 130.46 (2× CH–*o*-Bn), 128.95 (2× CH–Ar), 128.88 (2× CH–*o*-Ph), 128.76 (2× CH–Ar), 128.46 (2× CH–*m*-Bn), 127.92 (2× CH–*m*-Bz), 126.95 (CH–*p*-Bn), 126.64 (CH-7), 71.55 (CH_2_-15), 69.44 (CH_2_-14), 62.96 (C-9), 57.38 (CH-3), 52.85 (COO*C*H_3_), 47.23 (CH-4), 39.98 (CH_2_-10), 38.01 (CH-8), 35.84 (CH_2_-16), 34.19 (CH-5), 29.56 (CH_2_-13), 20.98 (Ph*C*H_3_), 19.75 (CH_3_-12), 12.97 (CH_3_-11) ppm. HRMS (ESI) *m*/*z* calcd for C_37_H_42_O_5_N^+^ [M + H]^+^ 580.3058, found 580.3056.

##### Methyl (1*S*,3*aR*,4*S*,7*S*,7*aR*)-2-benzoyl-1-benzyl-4-(2-(4-fluorophenethoxy)ethyl)-6,7-dimethyl-3-oxo-1,2,3,4,7,7*a*-hexahydro-3*aH*-isoindole-3*a*-carboxylate (16d)

By following the general procedure for etherifications, the reaction was carried out with iodide 15d (200 mg, 0.80 mmol). FC furnished compound 16d (93 mg, 64%), transparent oil; [*α*]_D_ = +85.2° (c 0.28; CHCl_3_) and small amount of 2-(4-fluorophenyl)ethan-1-ol as an impurity. ^1^H NMR (400 MHz, CDCl_3_) *δ* 7.62 (m, 2H, 2× *o*-Bz), 7.52 (m, 1H, *p*-Bz), 7.42 (m, 2H, 2× *m*-Bz), 7.35–7.24 (m, 3H, 2× *m*-Bn, *p*-Bn), 7.18–7.10 (m, 4H, 2× *o*-Bn, 2× *o*-Ph), 6.92 (m, 2H, 2× *m*-Ph), 5.43 (m, 1H, H-7), 4.29 (dt, *J*_3,10a_ = 8.1 Hz, *J*_3,4_ = *J*_3,10b_ = 3.3 Hz, 1H, H-3), 3.67 (s, 3H, COOCH_3_), 3.57 (dt, *J*_gem_ = 9.3 Hz, *J*_15b,16a_ = *J*_15b,16b_ = 6.8 Hz, 1H, H-15b), 3.47 (dt, *J*_gem_ = 9.3 Hz, *J*_15a,16a_ = *J*_15a,16b_ = 6.8 Hz, 1H, H-15a), 3.45–3.35 (m, 2H, H-14a,b), 3.12 (dd, *J*_gem_ = 13.4 Hz, *J*_10b,3_ = 3.3 Hz, 1H, H-10b), 2.94 (dd, *J*_gem_ = 13.4 Hz, *J*_10a,3_ = 8.1 Hz, 1H, H-10a), 2.81–2.74 (m, 2H, H-16a,b), 2.72 (dd, *J*_4,5_ = 5.7 Hz, *J*_4,3_ = 3.3 Hz, 1H, H-4), 2.61 (m, 1H, H-8), 2.39 (m, 1H, H-5), 2.12–2.00 (m, 1H, H-13b), 1.83–1.73 (m, 1H, H-13a), 1.72 (m, 3H, CH_3_-12), 0.85 (d, *J*_11,5_ = 7.3 Hz, 3H, CH_3_-11) ppm. ^13^C NMR (101 MHz, CDCl_3_) *δ* 172.46 (2C *overlapped*, C-1, *C*OOCH_3_), 170.25 (C=O–Bz), 161.22 (d, *J*_C,F_ = 244 Hz, C–*p*-Ph), 140.17 (C-6), 136.12 (C–*i*-Bn), 134.98 (d, *J*_C,F_ = 3.2 Hz, C–*i*-Ph), 134.64 (C–*i*-Bz), 132.18 (CH–*p*-Bz), 130.49 (2× CH–*o*-Bn), 130.30 (d, *J*_C,F_ = 8.1 Hz, 2× CH–*o*-Ph), 128.98 (2× CH–*o*-Bz), 128.47 (2× CH–*m*-Bn), 127.93 (2× CH–*m*-Bz), 126.97 (CH–*p*-Bn), 126.56 (CH-7), 114.88 (d, *J*_C,F_ = 21.2 Hz, 2× CH–*m*-Ph), 71.24 (CH_2_-15), 69.50 (CH_2_-14), 62.93 (C-9), 57.39 (CH-3), 52.87 (COO*C*H_3_), 47.26 (CH-4), 39.95 (CH_2_-10), 37.99 (CH-8), 35.46 (CH_2_-16), 34.23 (CH-5), 29.55 (CH_2_-13), 19.77 (CH_3_-12), 12.96 (CH_3_-11) ppm. ^19^F NMR (376 MHz, CDCl_3_) −144.71 (s, 1F). HRMS (ESI) *m*/*z* calcd for C_36_H_39_O_5_NF^+^ [M + H]^+^ 584.2807, found 584.2807.

##### Methyl (1*S*,3*aR*,4*S*,7*S*,7*aR*)-2-benzoyl-1-benzyl-4-(2-(3-fluorophenethoxy)ethyl)-6,7-dimethyl-3-oxo-1,2,3,4,7,7*a*-hexahydro-3*aH*-isoindole-3*a*-carboxylate (16e)

By following the general procedure for etherifications, the reaction was carried out with iodide 15e (200 mg, 0.80 mmol). FC furnished compound 16e (74 mg, 51%), transparent oil; [*α*]_D_ = +77.7° (c 0.25; CHCl_3_) and small amount of 2-(3-fluorophenyl)ethan-1-ol as an impurity. ^1^H NMR (400 MHz, CDCl_3_) *δ* 7.63 (m, 2H, 2× *o*-Bz), 7.52 (m, 1H, *p*-Bz), 7.42 (m, 2H, 2× *m*-Bz), 7.35–7.23 (m, 3H, 2× *m*-Bn, *p*-Bn), 7.20 (m, 1H, H-5′), 7.15 (m, 2H, 2× *o*-Bn), 6.95 (m, 1H, H-6′), 6.91 (m, 1H, H-2′), 6.86 (m, 1H, H-4′), 5.44 (m, 1H, H-7), 4.29 (dt, *J*_3,10a_ = 8.2 Hz, *J*_3,4_ = *J*_3,10b_ = 3.3 Hz, 1H, H-3), 3.68 (s, 3H, COOCH_3_), 3.60 (dt, *J*_gem_ = 9.4 Hz, *J*_15b,16a_ = *J*_15b,16b_ = 6.7 Hz, 1H, H-15b), 3.50 (dt, *J*_gem_ = 9.4 Hz, *J*_15a,16a_ = *J*_15a,16b_ = 6.7 Hz, 1H, H-15a), 3.46–3.36 (m, 2H, H-14a,b), 3.12 (dd, *J*_gem_ = 13.4 Hz, *J*_10b,3_ = 3.3 Hz, 1H, H-10b), 2.94 (dd, *J*_gem_ = 13.4 Hz, *J*_10a,3_ = 8.2 Hz, 1H, H-10a), 2.84–2.76 (m, 2H, H-16a,b), 2.72 (dd, *J*_4,5_ = 5.7 Hz, *J*_4,3_ = 3.3 Hz, 1H, H-4), 2.64 (m, 1H, H-8), 2.40 (m, 1H, H-5), 2.13–2.01 (m, 1H, H-13b), 1.84–1.73 (m, 1H, H-13a), 1.72 (m, 3H, CH_3_-12), 0.84 (d, *J*_11,5_ = 7.3 Hz, 3H, CH_3_-11) ppm. ^13^C NMR (101 MHz, CDCl_3_) *δ* 172.50 (2C *overlapped*, *C*OOCH_3_, C-1), 170.30 (C=O–Bz), 162.75 (d, *J*_C,F_ = 245 Hz, C-3′), 142.00 (d, *J*_C,F_ = 7.1 Hz, C–*i*-Ph), 140.24 (C-6), 136.12 (C–*i*-Bn), 134.50 (C–*i*-Bz), 132.16 (CH–*p*-Bz), 130.47 (2× CH–*o*-Bn), 129.48 (d, *J*_C,F_ = 8.1 Hz, CH-5′), 128.96 (2× CH–*o*-Bz), 128.45 (2× CH–*m*-Bn), 127.92 (2× CH–*m*-Bz), 126.95 (CH–*p*-Bn), 126.47 (CH-7), 124.55 (d, *J*_C,F_ = 2.0 Hz, CH-6′), 115.76 (d, *J*_C,F_ = 21 Hz, CH-2′), 112.82 (d, *J*_C,F_ = 20 Hz, CH-4′), 70.81 (CH_2_-15), 69.44 (CH_2_-14), 62.95 (C-9), 57.40 (CH-3), 52.85 (COO*C*H_3_), 47.23 (CH-4), 39.95 (CH_2_-10), 37.93 (CH-8), 35.97 (d, *J*_C,F_ = 2.0 Hz, CH_2_-16), 34.20 (CH-5), 29.53 (CH_2_-13), 19.74 (CH_3_-12), 12.93 (CH_3_-11) ppm. ^19^F NMR (376 MHz, CDCl_3_) −114.55 (s, 1F). HRMS (ESI) *m*/*z* calcd for C_36_H_39_O_5_NF^+^ [M + H]^+^ 584.2807, found 584.2802.

##### Methyl (1*S*,3*aR*,4*S*,7*S*,7*aR*)-2-benzoyl-1-benzyl-4-(2-(2-fluorophenethoxy)ethyl)-6,7-dimethyl-3-oxo-1,2,3,4,7,7*a*-hexahydro-3*aH*-isoindole-3*a*-carboxylate (16f)

By following the general procedure for etherifications, the reaction was carried out with iodide 15f (200 mg, 0.80 mmol). FC furnished compound 16f (66 mg, 45%), transparent oil; [*α*]_D_ = +88.7° (c 0.22; CHCl_3_). ^1^H NMR (400 MHz, CDCl_3_) *δ* 7.65 (m, 2H, 2× *o*-Bz), 7.52 (m, 1H, *p*-Bz), 7.42 (m, 2H, 2× *m*-Bz), 7.35–7.23 (m, 3H, 2× *m*-Bn, *p*-Bn), 7.20 (m, 1H, H-6′), 7.18–7.21 (m, 3H, 2× *o*-Bn, H-4′), 7.01 (m, 1H, H-5′), 6.97 (m, 1H, H-3′), 5.45 (m, 1H, H-7), 4.29 (dt, *J*_3,10a_ = 8.2 Hz, *J*_3,4_ = *J*_3,10b_ = 3.3 Hz, 1H, H-3), 3.68 (s, 3H, COOCH_3_), 3.60 (dt, *J*_gem_ = 9.3 Hz, *J*_15b,16a_ = *J*_15b,16b_ = 6.9 Hz, 1H, H-15b), 3.51 (dt, *J*_gem_ = 9.3 Hz, *J*_15a,16a_ = *J*_15a,16b_ = 6.9 Hz, 1H, H-15a), 3.47–3.37 (m, 2H, H-14a,b), 3.12 (dd, *J*_gem_ = 13.4 Hz, *J*_10b,3_ = 3.3 Hz, 1H, H-10b), 2.93 (dd, *J*_gem_ = 13.4 Hz, *J*_10a,3_ = 8.2 Hz, 1H, H-10a), 2.89–2.82 (m, 2H, H-16a,b), 2.72 (dd, *J*_4,5_ = 5.7 Hz, *J*_4,3_ = 3.3 Hz, 1H, H-4), 2.63 (m, 1H, H-8), 2.40 (m, 1H, H-5), 2.13–2.02 (m, 1H, H-13b), 1.83–1.73 (m, 1H, H-13a), 1.72 (m, 3H, CH_3_-12), 0.84 (d, *J*_11,5_ = 7.3 Hz, 3H, CH_3_-11) ppm. ^13^C NMR (101 MHz, CDCl_3_) *δ* 172.52 (*C*OOCH_3_), 172.45 (C-1), 170.30 (C=O–Bz), 162.40 (d, *J*_C,F_ = 245 Hz, C-2′), 140.11 (C-6), 136.15 (C–*i*-Bn), 134.51 (C–*i*-Bz), 132.15 (CH–*p*-Bz), 131.33 (d, *J*_C,F_ = 5.1 Hz, CH-6′), 130.46 (2× CH–*o*-Bn), 128.96 (2× CH–*o*-Bz), 128.46 (2× CH–*m*-Bn), 127.92 (2× CH–*m*-Bz), 127.72 (d, *J*_C,F_ = 8.1 Hz, CH-4′), 126.95 (CH–*p*-Bn), 126.60 (CH-7), 125.98 (d, *J*_C,F_ = 16 Hz, C–*i*-Ph), 123.75 (d, *J*_C,F_ = 3.0 Hz, CH-5′), 115.03 (d, *J*_C,F_ = 22 Hz, C-3′), 69.89 (d, *J*_C,F_ = 1.0 Hz, CH_2_-15), 69.46 (CH_2_-14), 62.95 (C-9), 57.38 (CH-3), 52.85 (COO*C*H_3_), 47.23 (CH-4), 39.98 (CH_2_-10), 38.02 (CH-8), 34.17 (CH-5), 29.56 (CH_2_-13), 29.37 (d, *J*_C,F_ = 3.0 Hz, CH_2_-16), 19.76 (CH_3_-12), 12.95 (CH_3_-11) ppm. ^19^F NMR (376 MHz, CDCl_3_) *δ* −118.69 (s, 1F). HRMS (ESI) *m*/*z* calcd for C_36_H_39_O_5_NF^+^ [M + H]^+^ 584.2807, found 584.2808.

##### Methyl (1*S*,3*aR*,4*S*,7*S*,7*aR*)-2-benzoyl-1-benzyl-4-(2-(4-chlorophenethoxy)ethyl)-6,7-dimethyl-3-oxo-1,2,3,4,7,7*a*-hexahydro-3*aH*-isoindole-3*a*-carboxylate (16g)

By following the general procedure for etherifications, the reaction was carried out with iodide 15g (231 mg, 0.80 mmol). FC furnished compound 16g (98 mg, 65%), transparent oil; [*α*]_D_ = +78.6° (c 0.20; CHCl_3_). ^1^H NMR (400 MHz, CDCl_3_) *δ* 7.62 (m, 2H, 2× *o*-Bz), 7.52 (m, 1H, *p*-Bz), 7.42 (m, 2H, 2× *m*-Bz), 7.35–7.23 (m, 3H, 2× *m*-Bn, *p*-Bn), 7.20 (m, 2H, 2× *m*-Ph), 7.15 (m, 2H, 2× *o*-Bn), 7.11 (m, 2H, 2× *o*-Ph), 5.42 (m, 1H, H-7), 4.29 (dt, *J*_3,10a_ = 8.1 Hz, *J*_3,4_ = *J*_3,10b_ = 3.3 Hz, 1H, H-3), 3.67 (s, 3H, COOCH_3_), 3.58 (dt, *J*_gem_ = 9.3 Hz, *J*_15b,16a_ = *J*_15b,16b_ = 6.7 Hz, 1H, H-15b), 3.47 (dt, *J*_gem_ = 9.3 Hz, *J*_15a,16a_ = *J*_15a,16b_ = 6.7 Hz, 1H, H-15a), 3.46–3.34 (m, 2H, H-14a,b), 3.12 (dd, *J*_gem_ = 13.4 Hz, *J*_10b,3_ = 3.3 Hz, 1H, H-10b), 2.94 (dd, *J*_gem_ = 13.4 Hz, *J*_10a,3_ = 8.1 Hz, 1H, H-10a), 2.81–2.74 (m, 2H, H-16a,b), 2.72 (dd, *J*_4,5_ = 5.7 Hz, *J*_4,3_ = 3.3 Hz, 1H, H-4), 2.61 (m, 1H, H-8), 2.38 (m, 1H, H-5), 2.12–2.00 (m, 1H, H-13b), 1.83–1.73 (m, 1H, H-13a), 1.72 (m, 3H, CH_3_-12), 0.85 (d, *J*_11,5_ = 7.3 Hz, 3H, CH_3_-11) ppm. ^13^C NMR (101 MHz, CDCl_3_) *δ* 172.49 (*C*OOCH_3_), 172.47 (C-1), 170.29 (C=O–Bz), 140.18 (C-6), 137.85 (C–*i*-Ph), 136.10 (C–*i*-Bn), 134.49 (C–*i*-Bz), 132.18 (CH–*p*-Bz), 131.73 (C–*p*-Ph), 130.48 (2× CH–*o*-Bn), 130.30 (2× CH–Ar), 128.96 (2× CH–Ar), 128.46 (2× CH–Ar), 128.21 (2× CH–Ar), 127.92 (2× CH–*m*-Ph), 126.96 (CH–*p*-Bn), 126.48 (CH-7), 70.94 (CH_2_-15), 69.45 (CH_2_-14), 62.91 (C-9), 57.38 (CH-3), 52.86 (COO*C*H_3_), 47.24 (CH-4), 39.92 (CH_2_-10), 37.93 (CH-8), 35.60 (CH_2_-16), 34.23 (CH-5), 29.52 (CH_2_-13), 19.76 (CH_3_-12), 12.98 (CH_3_-11) ppm. HRMS (ESI) *m*/*z* calcd for C_36_H_39_O_5_NCl^+^ [M + H]^+^ 600.2511, found 600.2509.

##### Methyl (1*S*,3*aR*,4*S*,7*S*,7*aR*)-2-benzoyl-1-benzyl-4-(2-(4-bromophenethoxy)ethyl)-6,7-dimethyl-3-oxo-1,2,3,4,7,7*a*-hexahydro-3*aH*-isoindole-3*a*-carboxylate (16h)

By following the general procedure for etherifications, the reaction was carried out with iodide 15h (249 mg, 0.80 mmol). FC furnished compound 16h (103 mg, 64%), transparent oil; [*α*]_D_ = +69.3° (c 0.33; CHCl_3_). ^1^H NMR (400 MHz, CDCl_3_) *δ* 7.62 (m, 2H, 2× *o*-Bz), 7.52 (m, 1H, *p*-Bz), 7.42 (m, 2H, 2× *m*-Bz), 7.35 (m, 2H, 2× *m*-Ph), 7.34–7.24 (m, 3H, 2× *m*-Bn, *p*-Bn), 7.15 (m, 2H, 2× *o*-Bn), 7.06 (m, 2H, 2× *o*-Ph), 5.42 (m, 1H, H-7), 4.29 (dt, *J*_3,10a_ = 8.1 Hz, *J*_3,4_ = *J*_3,10b_ = 3.3 Hz, 1H, H-3), 3.67 (s, 3H, COOCH_3_), 3.58 (dt, *J*_gem_ = 9.3 Hz, *J*_15b,16a_ = *J*_15b,16b_ = 6.6 Hz, 1H, H-15b), 3.47 (dt, *J*_gem_ = 9.3 Hz, *J*_15a,16a_ = *J*_15a,16b_ = 6.6 Hz, 1H, H-15a), 3.45–3.34 (m, 2H, H-14a,b), 3.12 (dd, *J*_gem_ = 13.4 Hz, *J*_10b,3_ = 3.3 Hz, 1H, H-10b), 2.94 (dd, *J*_gem_ = 13.4 Hz, *J*_10a,3_ = 8.1 Hz, 1H, H-10a), 2.80–2.72 (m, 2H, H-16a,b), 2.72 (dd, *J*_4,5_ = 5.7 Hz, *J*_4,3_ = 3.3 Hz, 1H, H-4), 2.60 (m, 1H, H-8), 2.38 (m, 1H, H-5), 2.12–2.00 (m, 1H, H-13b), 1.82–1.73 (m, 1H, H-13a), 1.72 (m, 3H, CH_3_-12), 0.86 (d, *J*_11,5_ = 7.3 Hz, 3H, CH_3_-11) ppm. ^13^C NMR (101 MHz, CDCl_3_) *δ* 172.48 (2C *overlapped*, *C*OOCH_3_, C-1), 170.28 (C=O–Bz), 140.18 (C-6), 138.39 (C–*i*-Ph), 136.11 (C–*i*-Bn), 134.50 (C–*i*-Bz), 132.17 (CH–*p*-Bz), 131.17 (2× CH–*m*-Ph), 130.72 (2× CH–*o*-Ph), 130.48 (2× CH–*o*-Bn), 128.97 (2× CH–*o*-Bz), 128.47 (2× CH–*m*-Bn), 127.92 (2× CH–*m*-Bz), 126.96 (CH–*p*-Bn), 126.50 (CH-7), 119.79 (C–*p*-Ph), 70.85 (CH_2_-15), 69.45 (CH_2_-14), 62.92 (C-9), 57.38 (CH-3), 52.86 (COO*C*H_3_), 47.24 (CH-4), 39.93 (CH_2_-10), 37.93 (CH-8), 35.67 (CH_2_-16), 34.23 (CH-5), 29.53 (CH_2_-13), 19.78 (CH_3_-12), 13.01 (CH_3_-11) ppm. HRMS (ESI) *m*/*z* calcd for C_36_H_39_O_5_NBr^+^ [M + H]^+^ 644.2006, found 644.2007.

##### Methyl (1*S*,3*aR*,4*S*,7*S*,7*aR*)-2-benzoyl-1-benzyl-4-(2-(3-bromophenethoxy)ethyl)-6,7-dimethyl-3-oxo-1,2,3,4,7,7*a*-hexahydro-3*aH*-isoindole-3*a*-carboxylate (16i)

By following the general procedure for etherifications, the reaction was carried out with iodide 15i (249 mg, 0.80 mmol). FC furnished compound 16i (116 mg, 72%), transparent oil; [*α*]_D_ = +76.1° (c 0.23; CHCl_3_). ^1^H NMR (400 MHz, CDCl_3_) *δ* 7.63 (m, 2H, 2× *o*-Bz), 7.51 (m, 1H, *p*-Bz), 7.42 (m, 2H, 2× *m*-Bz), 7.37 (m, 1H, H-2′), 7.35–7.23 (m, 4H, 1× Ph, 2× *m*-Bn, *p*-Bn), 7.16 (m, 2H, 2× *o*-Bn), 7.13–7.06 (m, 2H, 1× Ph), 5.43 (m, 1H, H-7), 4.29 (dt, *J*_3,10a_ = 8.2 Hz, *J*_3,4_ = *J*_3,10b_ = 3.3 Hz, 1H, H-3), 3.69 (s, 3H, COOCH_3_), 3.60 (dt, *J*_gem_ = 9.3 Hz, *J*_15b,16a_ = *J*_15b,16b_ = 6.6 Hz, 1H, H-15b), 3.48 (dt, *J*_gem_ = 9.3 Hz, *J*_15a,16a_ = *J*_15a,16b_ = 6.6 Hz, 1H, H-15a), 3.46–3.36 (m, 2H, H-14a,b), 3.13 (dd, *J*_gem_ = 13.3 Hz, *J*_10b,3_ = 3.3 Hz, 1H, H-10b), 2.93 (dd, *J*_gem_ = 13.3 Hz, *J*_10a,3_ = 8.2 Hz, 1H, H-10a), 2.82–2.74 (m, 2H, H-16a,b), 2.73 (dd, *J*_4,5_ = 5.8 Hz, *J*_4,3_ = 3.3 Hz, 1H, H-4), 2.63 (m, 1H, H-8), 2.39 (m, 1H, H-5), 2.12–2.00 (m, 1H, H-13b), 1.84–1.74 (m, 1H, H-13a), 1.72 (m, 3H, CH_3_-12), 0.83 (d, *J*_11,5_ = 7.3 Hz, 3H, CH_3_-11) ppm. ^13^C NMR (101 MHz, CDCl_3_) *δ* 172.50 (2C *overlapped*, *C*OOCH_3_, C-1), 170.29 (C=O–Bz), 141.83 (C–*i*-Ph), 140.23 (C-6), 136.16 (C–*i*-Bn), 134.51 (C–*i*-Bz), 132.16 (CH–*p*-Bz), 131.91 (CH-2′), 130.47 (2× CH–*o*-Bn), 129.69 (CH–Ph), 129.10 (CH–Ph), 128.96 (2× CH–*o*-Bz), 128.46 (2× CH–*m*-Bn), 127.92 (2× CH–*m*-Bz), 127.67 (CH–Ph), 126.96 (CH–*p*-Bn), 126.48 (CH-7), 122.23 (C-3′), 70.70 (CH_2_-15), 69.38 (CH_2_-14), 62.97 (C-9), 57.41 (CH-3), 52.86 (COO*C*H_3_), 47.21 (CH-4), 39.97 (CH_2_-10), 37.82 (CH-8), 35.93 (CH_2_-16), 34.19 (CH-5), 29.52 (CH_2_-13), 19.78 (CH_3_-12), 12.94 (CH_3_-11) ppm. HRMS (ESI) *m*/*z* calcd for C_36_H_39_O_5_NBr^+^ [M + H]^+^ 644.2006, found 644.2007.

##### Methyl (1*S*,3*aR*,4*S*,7*S*,7*aR*)-2-benzoyl-1-benzyl-6,7-dimethyl-4-(2-(4-nitrophenethoxy)ethyl)-3-oxo-1,2,3,4,7,7*a*-hexahydro-3*aH*-isoindole-3*a*-carboxylate (16j)

By following the general procedure for etherifications, the reaction was carried out with iodide 15j (222 mg, 0.80 mmol). FC furnished compound 16j (78 mg, 51%), yellowish oil; [*α*]_D_ = +77.5° (c 0.29; CHCl_3_). ^1^H NMR (400 MHz, CDCl_3_) *δ* 8.10 (m, 2H, 2× *m*-Ph), 7.62 (m, 2H, 2× *o*-Bz), 7.52 (m, 1H, *p*-Bz), 7.41 (m, 2H, 2× *m*-Bz), 7.35 (m, 2H, 2× *o*-Ph), 7.34–7.24 (m, 3H, 2× *m*-Bn, *p*-Bn), 7.15 (m, 2H, 2× *o*-Bn), 5.41 (m, 1H, H-7), 4.28 (dt, *J*_3,10a_ = 7.9 Hz, *J*_3,4_ = *J*_3,10b_ = 3.3 Hz, 1H, H-3), 3.66 (s, 3H, COOCH_3_), 3.63 (dt, *J*_gem_ = 9.3 Hz, *J*_15b,16a_ = *J*_15b,16b_ = 6.4 Hz, 1H, H-15b), 3.53 (dt, *J*_gem_ = 9.3 Hz, *J*_15a,16a_ = *J*_15a,16b_ = 6.4 Hz, 1H, H-15a), 3.45–3.34 (m, 2H, H-14a,b), 3.09 (dd, *J*_gem_ = 13.4 Hz, *J*_10b,3_ = 3.3 Hz, 1H, H-10b), 2.96 (dd, *J*_gem_ = 13.4 Hz, *J*_10a,3_ = 7.9 Hz, 1H, H-10a), 2.94–2.87 (m, 2H, H-16a,b), 2.71 (dd, *J*_4,5_ = 5.7 Hz, *J*_4,3_ = 3.3 Hz, 1H, H-4), 2.59 (m, 1H, H-8), 2.35 (m, 1H, H-5), 2.11–2.00 (m, 1H, H-13b), 1.82–1.72 (m, 1H, H-13a), 1.72 (m, 3H, CH_3_-12), 0.85 (d, *J*_11,5_ = 7.3 Hz, 3H, CH_3_-11) ppm. ^13^C NMR (101 MHz, CDCl_3_) *δ* 172.47 (*C*OOCH_3_), 172.44 (C-1), 170.25 (C=O–Bz), 147.52 (C–*i*-Ph), 146.47 (C–*p*-Ph), 140.30 (C-6), 136.01 (C–*i*-Bn), 134.45 (C–*i*-Bz), 132.22 (CH–*p*-Bz), 130.50 (2× CH–*o*-Bn), 129.82 (2× CH–*o*-Ph), 128.99 (2× CH–*o*-Bz), 128.47 (2× CH–*m*-Bn), 127.92 (2× CH–*m*-Bz), 126.99 (CH–*p*-Bn), 126.38 (CH-7), 123.32 (2× CH–*m*-Ph), 70.12 (CH_2_-15), 69.54 (CH_2_-14), 62.85 (C-9), 57.37 (CH-3), 52.89 (COO*C*H_3_), 47.24 (CH-4), 39.83 (CH_2_-10), 37.84 (CH-8), 36.13 (CH_2_-16), 34.30 (CH-5), 29.52 (CH_2_-13), 19.78 (CH_3_-12), 12.94 (CH_3_-11) ppm. HRMS (ESI) *m*/*z* calcd for C_36_H_39_O_7_N_2_^+^ [M + H]^+^ 611.2752, found 611.2748.

#### General procedure for debenzoylations

A solution of KOH (46 mg, 0.80 mmol) in H_2_O (0.2 mL) was added dropwise to a stirred solution of benzoylated derivative 16 (0.10 mmol) in toluene (1.5 mL) and MeOH (0.5 mL) at 15 °C. The reaction mixture was vigorously stirred at room temperature until full conversion (6–12 h, TLC). Then, the reaction mixture was poured into a saturated aqueous NaHCO_3_ (10 mL), and the resulting solution was extracted with EtOAc (3 × 5 mL). The organic layers were combined, washed with brine (5 mL), dried (MgSO_4_) and concentrated under reduced pressure. Column chromatography of the residue on silica gel (20–70% EtOAc in hexanes) furnished corresponding unprotected products 17.

##### Methyl (1*S*,3*aR*,4*S*,7*S*,7*aR*)-1-benzyl-6,7-dimethyl-3-oxo-4-(2-phenethoxyethyl)-1,2,3,4,7,7*a*-hexahydro-3*aH*-isoindole-3*a*-carboxylate (17a)

By following the general procedure for debenzoylations, the reaction was carried out with 16a (57 mg, 0.10 mmol). FC furnished compound 17a (36 mg, 79%), transparent wax; [*α*]_D_ = −0.0° (c 0.24; CHCl_3_). ^1^H NMR (400 MHz, CDCl_3_) *δ* 7.36–7.15 (m, 10H, Ph, Bn), 5.82 (bs, 1H, NH), 5.49 (m, 1H, H-7), 3.76 (s, 3H, COOCH_3_), 3.67 (dt, *J*_gem_ = 9.4 Hz, *J*_15b,16a_ = *J*_15b,16b_ = 7.1 Hz, 1H, H-15b), 3.57 (dt, *J*_gem_ = 9.4 Hz, *J*_15a,16a_ = *J*_15a,16b_ = 7.2 Hz, 1H, H-15a), 3.47–3.57 (m, 2H, H-14a,b), 3.25 (dt, *J*_3,10a_ = 9.2 Hz, *J*_3,4_ = *J*_3,10b_ = 4.2 Hz, 1H, H-3), 2.89 (dd, *J*_gem_ = 13.5 Hz, *J*_10b,3_ = 4.0 Hz, 1H, H-10b), 2.79–2.94 (m, 2H, H-16a,b), 2.71 (m, 1H, H-8), 2.66 (dd, *J*_gem_ = 13.5 Hz, *J*_10a,3_ = 9.3 Hz, 1H, H-10a), 2.58 (t, *J*_4,3_ = *J*_4,5_ = 4.4 Hz, 1H, H-4), 2.48 (m, 1H, H-5), 2.11 (dtd, *J*_gem_ = 14.0 Hz, *J*_13b,14_ = 7.4 Hz, *J*_13b,8_ = 2.7 Hz, 1H, H-13b), 1.92 (dm, *J*_gem_ = 14.0 Hz, 1H, H-13a), 1.75 (m, 3H, CH_3_-12), 1.15 (d, *J*_11,5_ = 7.3 Hz, 3H, CH_3_-11) ppm. ^13^C NMR (101 MHz, CDCl_3_) *δ* 173.57 (C-1), 173.14 (*C*OOCH_3_), 139.27 (C–*i*-Ph), 138.97 (C-6), 137.34 (C–*i*-Bn), 129.15 (2× CH–*o*-Bn), 128.92 (2× CH–*o*-Ph), 128.83 (2× CH–*m*-Bn), 128.16 (2× CH–*m*-Ph), 126.95 (CH), 126.74 (CH-7), 125.93 (CH), 71.32 (CH_2_-15), 69.83 (CH_2_-14), 60.26 (C-9), 55.72 (CH-3), 53.72 (CH-4), 52.67 (COO*C*H_3_), 44.81 (CH_2_-10), 36.53 (CH-8), 36.31 (CH_2_-16), 34.22 (CH-5), 29.95 (CH_2_-13), 20.22 (CH_3_-12), 13.97 (CH_3_-11) ppm. HRMS (ESI) *m*/*z* calcd for C_29_H_36_O4N^+^ [M + H]^+^ 462.2639, found 462.2640.

##### Methyl (1*S*,3*aR*,4*S*,7*S*,7*aR*)-1-benzyl-4-(2-(4-methoxyphenethoxy)ethyl)-6,7-dimethyl-3-oxo-1,2,3,4,7,7*a*-hexahydro-3*aH*-isoindole-3*a*-carboxylate (17b)

By following the general procedure for debenzoylations, the reaction was carried out with 16b (60 mg, 0.10 mmol). FC furnished compound 17b (27 mg, 56%), transparent wax; [*α*]_D_ = −2.7° (c 0.30; CHCl_3_). ^1^H NMR (400 MHz, CDCl_3_) *δ* 7.32 (m, 2H, 2× *m*-Bn), 7.25 (m, 1H, *p*-Bn), 7.19–7.10 (m, 4H, 2× *o*-Bn, 2× *o*-Ph), 6.81 (m, 2H, 2× *m*-Ph), 5.87 (m, 1H, NH), 5.49 (m, 1H, H-7), 3.78 (s, 3H, OCH_3_), 3.76 (s, 3H, COOCH_3_), 3.63 (dt, *J*_gem_ = 9.3 Hz, *J*_15b,16a_ = *J*_15b,16b_ = 7.1 Hz, 1H, H-15b), 3.53 (dt, *J*_gem_ = 9.3 Hz, *J*_15a,16a_ = *J*_15a,16b_ = 7.1 Hz, 1H, H-15a), 3.56–3.48 (m, 2H, H-14a,b), 3.26 (dt, *J*_3,10a_ = 8.7 Hz, *J*_3,4_ = *J*_3,10b_ = 4.1 Hz, 1H, H-3), 2.89 (dd, *J*_gem_ = 13.4 Hz, *J*_10b,3_ = 4.0 Hz, 1H, H-10b), 2.84–2.76 (m, 2H, H-16a,b), 2.71 (m, 1H, H-8), 2.65 (dd, *J*_gem_ = 13.4 Hz, *J*_10a,3_ = 9.3 Hz, 1H, H-10a), 2.58 (t, *J*_4,3_ = *J*_4,5_ = 4.4 Hz, 1H, H-4), 2.48 (m, 1H, H-5), 2.14–2.04 (m, 1H, H-13b), 1.97–1.85 (m, 1H, H-13a), 1.75 (m, 3H, CH_3_-12), 1.15 (d, *J*_11,5_ = 7.3 Hz, 3H, CH_3_-11) ppm. ^13^C NMR (101 MHz, CDCl_3_) *δ* 173.80 (C-1), 172.97 (*C*OOCH_3_), 157.90 (C–*p*-Ph), 139.04 (C-6), 137.22 (C–*i*-Bn), 131.34 (C–*i*-Ph), 129.84 (2× CH–*o*-Ph), 129.17 (2× CH–*o*-Bn), 128.86 (2× CH–*m*-Bn), 127.00 (CH–*p*-Bn), 126.72 (CH-7), 113.63 (2× CH–*m*-Ph), 71.58 (CH_2_-15), 69.79 (CH_2_-14), 60.41 (C-9), 56.01 (CH-3), 55.18 (OCH_3_), 53.66 (CH-4), 52.72 (COO*C*H_3_), 44.70 (CH_2_-10), 36.59 (CH-8), 35.40 (CH_2_-16), 34.22 (CH-5), 29.95 (CH_2_-13), 20.22 (CH_3_-12), 13.95 (CH_3_-11) ppm. HRMS (ESI) *m*/*z* calcd for C_30_H_38_O_5_N^+^ [M + H]^+^ 492.2745, found 492.2744.

##### Methyl (1*S*,3*aR*,4*S*,7*S*,7*aR*)-1-benzyl-6,7-dimethyl-4-(2-(4-methylphenethoxy)ethyl)-3-oxo-1,2,3,4,7,7*a*-hexahydro-3*aH*-isoindole-3*a*-carboxylate (17c)

By following the general procedure for debenzoylations, the reaction was carried out with 16c (58 mg, 0.10 mmol). FC furnished compound 17c (37 mg, 78%), transparent wax; [*α*]_D_ = −4.7° (c 0.23; CHCl_3_). ^1^H NMR (400 MHz, CDCl_3_) 7.32 (m, 2H, 2× *m*-Bn), 7.25 (m, 1H, *p*-Bn), 7.15 (m, 2H, 2× *o*-Bn), 7.13–7.04 (m, 4H, 4× Ph), 5.79 (bs, 1H, NH), 5.49 (m, 1H, H-7), 3.77 (s, 3H, COOCH_3_), 3.64 (dt, *J*_gem_ = 9.2 Hz, *J*_15b,16a_ = *J*_15b,16b_ = 7.2 Hz, 1H, H-15b), 3.54 (dt, *J*_gem_ = 9.2 Hz, *J*_15a,16a_ = *J*_15a,16b_ = 7.2 Hz, 1H, H-15a), 3.54–3.46 (m, 2H, H-14a,b), 3.25 (dt, *J*_3,10a_ = 8.8 Hz, *J*_3,4_ = *J*_3,10b_ = 4.2 Hz, 1H, H-3), 2.89 (dd, *J*_gem_ = 13.4 Hz, *J*_10b,3_ = 4.0 Hz, 1H, H-10b), 2.85–2.79 (m, 2H, H-16a,b), 2.71 (m, 1H, H-8), 2.65 (dd, *J*_gem_ = 13.4 Hz, *J*_10a,3_ = 9.3 Hz, 1H, H-10a), 2.58 (t, *J*_4,3_ = *J*_4,5_ = 4.4 Hz, 1H, H-4), 2.48 (m, 1H, H-5), 2.31 (s, 3H, PhC*H*_3_), 2.15–2.06 (m, 1H, H-13b), 2.96–2.87 (m, 1H, H-13a), 1.75 (m, 3H, CH_3_-12), 1.16 (d, *J*_11,5_ = 7.3 Hz, 3H, CH_3_-11) ppm. ^13^C NMR (101 MHz, CDCl_3_) *δ* 173.65 (C-1), 173.07 (*C*OOCH_3_), 138.98 (C-6), 137.32 (C–*i*-Bn), 136.13 (C–*i*-Ph), 135.40 (C–*p*-Ph), 129.13 (2× CH–Bn), 128.89 (4× CH–Ar), 128.82 (2× CH–Ph), 127.02 (CH-7), 126.79 (CH–*p*-Bn), 71.53 (CH_2_-15), 69.83 (CH_2_-14), 60.33 (C-9), 55.89 (CH-3), 53.80 (CH-4), 52.72 (COO*C*H_3_), 44.84 (CH_2_-10), 36.59 (CH-8), 35.89 (CH_2_-16), 34.25 (CH-5), 29.97 (CH_2_-13), 20.99 (Ph*C*H_3_), 20.23 (CH_3_-12), 14.00 (CH_3_-11) ppm. HRMS (ESI) *m*/*z* calcd for C_30_H_38_O_4_N^+^ [M + H]^+^ 476.2795, found 476.2790.

##### Methyl (1*S*,3*aR*,4*S*,7*S*,7*aR*)-1-benzyl-4-(2-(4-fluorophenethoxy)ethyl)-6,7-dimethyl-3-oxo-1,2,3,4,7,7*a*-hexahydro-3*aH*-isoindole-3*a*-carboxylate (17d)

By following the general procedure for debenzoylations, the reaction was carried out with 16d (58 mg, 0.10 mmol). FC furnished compound 17d (34 mg, 71%), transparent wax; [*α*]_D_ = −4.1° (c 0.42; CHCl_3_). ^1^H NMR (400 MHz, CDCl_3_) *δ* 7.32 (m, 2H, 2× *m*-Bn), 7.24 (m, 1H, *p*-Bn), 7.21–7.11 (m, 4H, 2× *o*-Ph, 2× *o*-Bn), 6.93 (m, 2H, 2× *m*-Ph), 5.90 (m, 1H, NH), 5.47 (m, 1H, H-7), 3.75 (s, 3H, COOCH_3_), 3.64 (dt, *J*_gem_ = 9.2 Hz, *J*_15b,16a_ = *J*_15b,16b_ = 6.8 Hz, 1H, H-15b), 3.53 (dt, *J*_gem_ = 9.2 Hz, *J*_15a,16a_ = *J*_15a,16b_ = 6.8 Hz, 1H, H-15a), 3.53–3.47 (m, 2H, H-14a,b), 3.25 (dt, *J*_3,10a_ = 9.3 Hz, *J*_3,4_ = *J*_3,10b_ = 6.9 Hz, 1H, H-3), 2.89 (dd, *J*_gem_ = 13.5 Hz, *J*_10b,3_ = 4.0 Hz, 1H, H-10b), 2.85–2.78 (m, 2H, H-16a,b), 2.69 (m, 1H, H-8), 2.65 (dd, *J*_gem_ = 13.5 Hz, *J*_10a,3_ = 9.3 Hz, 1H, H-10a), 2.57 (t, *J*_4,3_ = *J*_4,5_ = 4.4 Hz, 1H, H-4), 2.45 (m, 1H, H-5), 2.14–2.03 (m, 1H, H-13b), 1.96–1.84 (m, 1H, H-13a), 1.74 (m, 3H, CH_3_-12), 1.14 (d, *J*_11,5_ = 7.3 Hz, 3H, CH_3_-11) ppm. ^13^C NMR (101 MHz, CDCl_3_) *δ* 173.76 (C-1), 172.99 (*C*OOCH_3_), 161.37 (d, *J*_C,F_ = 244 Hz, C–*p*-Ph), 139.06 (C-6), 137.21 (C–*i*-Bn), 135.03 (d, *J*_C,F_ = 3.0 Hz, C–*i*-Ph), 130.30 (d, *J*_C,F_ = 8.1 Hz, 2× CH–*o*-Ph), 129.18 (2× CH–*o*-Bn), 128.84 (2× CH–*m*-Bn), 126.99 (CH–*p*-Bn), 126.62 (CH-7), 114.80 (d, *J*_C,F_ = 21 Hz, 2× CH–*m*-Ph), 71.16 (CH_2_-15), 69.79 (CH_2_-14), 60.36 (C-9), 55.95 (CH-3), 53.64 (CH-4), 52.70 (COO*C*H_3_), 44.68 (CH_2_-10), 36.52 (CH-8), 35.47 (CH_2_-16), 34.23 (CH-5), 29.91 (CH_2_-13), 20.21 (CH_3_-12), 13.93 (CH_3_-11) ppm. ^19^F NMR (376 MHz, CDCl_3_) *δ* −117.64 (s, 1F). HRMS (ESI) *m*/*z* calcd for C_29_H_35_O_4_NF^+^ [M + H]^+^ 480.2545, found 480.2544.

##### Methyl (1*S*,3*aR*,4*S*,7*S*,7*aR*)-1-benzyl-4-(2-(3-fluorophenethoxy)ethyl)-6,7-dimethyl-3-oxo-1,2,3,4,7,7*a*-hexahydro-3*aH*-isoindole-3*a*-carboxylate (17e)

By following the general procedure for debenzoylations, the reaction was carried out with 16e (58 mg, 0.10 mmol). FC furnished compound 17e (31 mg, 64%), transparent wax; [*α*]_D_ = +0.0° (c 0.29; CHCl_3_). ^1^H NMR (400 MHz, CDCl_3_) *δ* 7.32 (m, 2H, 2× *m*-Bn), 7.25 (m, 1H, *p*-Bn), 7.20 (m, 1H, H-5′), 7.16 (m, 2H, 2× *o*-Bn), 6.99 (m, 1H, H-6′), 6.95 (m, 1H, H-2′), 6.88 (m, 1H, H-4′), 5.81 (m, 1H, NH), 5.48 (m, 1H, H-7), 3.76 (s, 3H, COOCH_3_), 3.67 (dt, *J*_gem_ = 9.2 Hz, *J*_15b,16a_ = *J*_15b,16b_ = 6.8 Hz, 1H, H-15b), 3.56 (dt, *J*_gem_ = 9.2 Hz, *J*_15a,16a_ = *J*_15a,16b_ = 6.8 Hz, 1H, H-15a), 3.55–3.48 (m, 2H, H-14a,b), 3.25 (dt, *J*_3,10a_ = 9.2 Hz, *J*_3,4_ = *J*_3,10b_ = 4.1 Hz, 1H, H-3), 2.89 (dd, *J*_gem_ = 13.5 Hz, *J*_10b,3_ = 4.4 Hz, 1H, H-10b), 2.88–2.82 (m, 2H, H-16a,b), 2.72 (m, 1H, H-8), 2.65 (dd, *J*_gem_ = 13.5 Hz, *J*_10a,3_ = 9.2 Hz, 1H, H-10a), 2.58 (t, *J*_4,3_ = *J*_4,5_ = 4.5 Hz, 1H, H-4), 2.47 (m, 1H, H-5), 2.14–2.04 (m, 1H, H-13b), 1.95–1.85 (m, 1H, H-13a), 1.74 (m, 3H, CH_3_-12), 1.15 (d, *J*_11,5_ = 7.3 Hz, 3H, CH_3_-11) ppm. ^13^C NMR (101 MHz, CDCl_3_) *δ* 173.68 (C-1), 173.05 (*C*OOCH_3_), 162.76 (d, *J*_C,F_ = 246 Hz, C-3′), 142.07 (d, *J*_C,F_ = 7.1 Hz, C–*i*-Ph), 139.11 (C-6), 137.32 (C–*i*-Bn), 129.48 (d, *J*_C,F_ = 8.1 Hz, CH-5′), 129.13 (2× CH–*o*-Bn), 128.88 (2× CH–*m*-Bn), 127.01 (CH–*p*-Bn), 126.61 (CH-7), 124.59 (d, *J*_C,F_ = 3.0 Hz, CH-6′), 115.79 (d, *J*_C,F_ = 21 Hz, C-2′), 112.79 (d, *J*_C,F_ = 21 Hz, C-4′), 70.75 (CH_2_-15), 69.78 (CH_2_-14), 60.33 (C-9), 55.89 (CH-3), 53.79 (CH-4), 52.72 (COO*C*H_3_), 44.82 (CH_2_-10), 36.49 (CH-8), 36.03 (CH_2_-16), 34.25 (CH-5), 29.90 (CH_2_-13), 20.21 (CH_3_-12), 13.96 (CH_3_-11) ppm. ^19^F NMR (376 MHz, CDCl_3_) *δ* −114.07 (s, 1F). HRMS (ESI) *m*/*z* calcd for C_29_H_35_O_4_NF^+^ [M + H]^+^ 480.2545, found 480.2541.

##### Methyl (1*S*,3*aR*,4*S*,7*S*,7*aR*)-1-benzyl-4-(2-(2-fluorophenethoxy)ethyl)-6,7-dimethyl-3-oxo-1,2,3,4,7,7*a*-hexahydro-3*aH*-isoindole-3*a*-carboxylate (17f)

By following the general procedure for debenzoylations, the reaction was carried out with 16f (58 mg, 0.10 mmol). FC furnished compound 17f (30 mg, 62%), transparent wax; [*α*]_D_ = −4.1° (c 0.27; CHCl_3_). ^1^H NMR (400 MHz, CDCl_3_) *δ* 7.32 (m, 2H, 2× *m*-Bn), 7.27–7.21 (m, 2H, *p*-Bn, H-6′), 7.19–7.11 (m, 3H, 2× *o*-Bn, H-4′), 7.02 (m, 1H, H-5′), 6.98 (m, 1H, H-3′), 5.84 (bs, 1H, NH), 5.48 (m, 1H, H-7), 3.76 (s, 3H, COOCH_3_), 3.66 (dt, *J*_gem_ = 9.4 Hz, *J*_15b,16a_ = *J*_15b,16b_ = 7.0 Hz, 1H, H-15b), 3.57 (dt, *J*_gem_ = 9.4 Hz, *J*_15a,16a_ = *J*_15a,16b_ = 7.0 Hz, 1H, H-15a), 3.54–3.48 (m, 2H, H-14a,b), 3.25 (dt, *J*_3,10a_ = 8.8 Hz, *J*_3,4_ = *J*_3,10b_ = 4.1 Hz, 1H, H-3), 2.94–2.87 (m, 2H, H-16a,b), 2.89 (dd, *J*_gem_ = 13.4 Hz, *J*_10b,3_ = 4.0 Hz, 1H, H-10b), 2.70 (m, 1H, H-8), 2.65 (dd, *J*_gem_ = 13.4 Hz, *J*_10a,3_ = 9.4 Hz, 1H, H-10a), 2.57 (t, *J*_4,3_ = *J*_4,5_ = 4.5 Hz, 1H, H-4), 2.47 (m, 1H, H-5), 2.13–2.02 (m, 1H, H-13b), 1.96–1.85 (m, 1H, H-13a), 1.74 (m, 3H, CH_3_-12), 1.15 (d, *J*_11,5_ = 7.3 Hz, 3H, CH_3_-11) ppm. ^13^C NMR (101 MHz, CDCl_3_) *δ* 173.83 (C-1), 172.95 (*C*OOCH_3_), 161.21 (d, *J*_C,F_ = 245 Hz, C-2′), 139.04 (C-6), 137.21 (C–*i*-Bn), 131.35 (d, *J*_C,F_ = 5.1 Hz, CH-6′), 129.17 (2× CH–*o*-Bn), 128.87 (2× CH–*m*-Bn), 127.68 (d, *J*_C,F_ = 8.1 Hz, CH-4′), 127.02 (CH–*p*-Bn), 126.69 (CH-7), 126.03 (d, *J*_C,F_ = 16 Hz, C–*i*-Ph), 123.77 (d, *J*_C,F_ = 3.0 Hz, CH-5′), 115.01 (d, *J*_C,F_ = 22 Hz, CH-3′), 69.84 (CH-15), 69.79 (CH-14), 60.43 (C-9), 56.06 (CH-3), 53.66 (CH-4), 52.73 (COO*C*H_3_), 44.70 (CH_2_-10), 36.61 (CH-8), 34.21 (CH-5), 29.96 (CH_2_-13), 29.38 (CH_2_-16), 20.22 (CH_3_-12), 13.95 (CH_3_-11) ppm. ^19^F NMR (376 MHz, CDCl_3_) *δ* −118.67 (s, 1H). HRMS (ESI) *m*/*z* calcd for C_29_H_35_O_4_NF^+^ [M + H]^+^ 480.2545, found 480.2544.

##### Methyl (1*S*,3*aR*,4*S*,7*S*,7*aR*)-1-benzyl-4-(2-(4-chlorophenethoxy)ethyl)-6,7-dimethyl-3-oxo-1,2,3,4,7,7*a*-hexahydro-3*aH*-isoindole-3*a*-carboxylate (17g)

By following the general procedure for debenzoylations, the reaction was carried out with 16g (60 mg, 0.10 mmol). FC furnished compound 17g (31 mg, 63%), transparent wax; [*α*]_D_ = −5.3° (c 0.30; CHCl_3_). ^1^H NMR (400 MHz, CDCl_3_) *δ* 7.35–7.24 (m, 3H, 2× *m*-Bn, *p*-Bn), 7.24–7.12 (m, 6H, 2× *o*-Bn, 4× Ph), 5.79 (bs, 1H, NH), 5.46 (m, 1H, H-7), 3.76 (s, 3H, COOCH_3_), 3.65 (dt, *J*_gem_ = 9.6 Hz, *J*_15b,16a_ = *J*_15b,16b_ = 6.8 Hz, 1H, H-15b), 3.53 (dt, *J*_gem_ = 9.6 Hz, *J*_15a,16a_ = *J*_15a,16b_ = 6.8 Hz, 1H, H-15a), 3.52–3.47 (m, 2H, C-14a,b), 3.24 (dt, *J*_3,10a_ = 9.2 Hz, *J*_3,4_ = *J*_3,10b_ = 4.0 Hz, 1H, H-3), 2.89 (dd, *J*_gem_ = 13.4 Hz, *J*_10b,3_ = 4.0 Hz, 1H, H-10b), 2.85–2.78 (m, 2H, H-16a,b), 2.68 (m, 1H, H-8), 2.65 (dd, *J*_gem_ = 13.4 Hz, *J*_10a,3_ = 9.2 Hz, 1H, H-10a), 2.57 (t, *J*_4,3_ = *J*_4,5_ = 4.5 Hz, 1H, H-4), 2.44 (m, 1H, H-5), 2.13–2.03 (m, 1H, H-13b), 1.95–1.83 (m, 1H, H-13a), 1.74 (m, 3H, CH_3_-12), 1.16 (d, *J*_11,5_ = 7.3 Hz, 3H, CH_3_-11) ppm. ^13^C NMR (101 MHz, CDCl_3_) *δ* 173.66 (C-1), 173.05 (*C*OOCH_3_), 139.05 (C-6), 137.94 (C–*i*-Ph), 137.30 (C–*i*-Bn), 131.69 (C–*p*-Ph), 130.34 (2× CH–*o*-Ph), 129.13 (2× CH–*o*-Bn), 128.89 (2× CH–Ar), 128.22 (2× CH–Ar), 127.02 (CH–*p*-Bn), 126.62 (CH-7), 70.88 (CH_2_-15), 69.80 (CH_2_-14), 60.31 (C-9), 55.89 (CH-3), 53.81 (CH-4), 52.72 (COO*C*H_3_), 44.82 (CH_2_-10), 36.48 (CH-8), 35.65 (CH_2_-16), 34.26 (CH-5), 29.90 (CH_2_-13), 20.23 (CH_3_-12), 14.00 (CH_3_-11) ppm. HRMS (ESI) *m*/*z* calcd for C_29_H_35_O_4_NCl^+^ [M + H]^+^ 496.2249, found 496.2247.

##### Methyl (1*S*,3*aR*,4*S*,7*S*,7*aR*)-1-benzyl-4-(2-(4-bromophenethoxy)ethyl)-6,7-dimethyl-3-oxo-1,2,3,4,7,7*a*-hexahydro-3*aH*-isoindole-3*a*-carboxylate (17h)

By following the general procedure for debenzoylations, the reaction was carried out with 16h (64 mg, 0.10 mmol). FC furnished compound 17h (37 mg, 69%), transparent wax; [*α*]_D_ = −2.1° (c 0.42; CHCl_3_). ^1^H NMR (400 MHz, CDCl_3_) *δ* 7.36 (m, 2H, 2× *m*-Ph), 7.32 (m, 2H, 2× *m*-Bn), 7.24 (m, 1H, *p*-Bn), 7.15 (m, 2H, 2× *o*-Bn), 7.10 (m, 2H, 2× *o*-Ph), 5.68 (m, 1H, NH), 5.46 (m, 1H, H-7), 3.76 (s, 3H, COOCH_3_), 3.65 (dt, *J*_gem_ = 9.2 Hz, *J*_15b,16a_ = *J*_15b,16b_ = 6.8 Hz, 1H, H-15b), 3.53 (dt, *J*_gem_ = 9.2 Hz, *J*_15a,16a_ = *J*_15a,16b_ = 6.8 Hz, 1H, H-15a), 3.54–3.46 (m, 2H, H-14a,b), 3.23 (dt, *J*_3,10a_ = 9.0 Hz, *J*_3,4_ = *J*_3,10b_ = 4.0 Hz, 1H, H-3), 2.89 (dd, *J*_gem_ = 13.5 Hz, *J*_10b,3_ = 4.0 Hz, 1H, H-10b), 2.84–2.76 (m, 2H, H-16a,b), 2.68 (m, 1H, H-8), 2.64 (dd, *J*_gem_ = 13.5 Hz, *J*_10a,3_ = 9.4 Hz, 1H, H-10a), 2.57 (t, *J*_4,3_ = *J*_4,5_ = 4.4 Hz, 1H, H-4), 2.44 (m, 1H, H-5), 2.14–2.04 (m, 1H, H-13b), 1.94–1.83 (m, 1H, H-13a), 1.74 (m, 3H, CH_3_-12), 1.16 (d, *J*_11,5_ = 7.3 Hz, 3H, CH_3_-11) ppm. ^13^C NMR (101 MHz, CDCl_3_) *δ* 173.48 (C-1), 173.14 (*C*OOCH_3_), 138.99 (C-6), 138.48 (C–*i*-Ph), 137.38 (C–*i*-Bn), 131.16 (2× CH–*m*-Ph), 130.76 (2× CH–*o*-Ph), 129.11 (2× CH–*o*-Bn), 128.87 (2× CH–*m*-Bn), 126.99 (CH–*p*-Bn), 126.65 (CH-7), 119.74 (C–*p*-Ph), 70.77 (CH_2_-15), 69.81 (CH_2_-14), 60.21 (C-9), 55.70 (CH-3), 53.86 (CH-4), 52.69 (COO*C*H_3_), 44.89 (CH_2_-10), 36.45 (CH-8), 35.71 (CH_2_-16), 34.27 (CH-5), 29.89 (CH_2_-13), 20.23 (CH_3_-12), 14.03 (CH_3_-11) ppm. HRMS (ESI) *m*/*z* calcd for C_29_H_35_O_4_NBr^+^ [M + H]^+^ 540.1744, found 540.1744.

##### Methyl (1*S*,3*aR*,4*S*,7*S*,7*aR*)-1-benzyl-4-(2-(3-bromophenethoxy)ethyl)-6,7-dimethyl-3-oxo-1,2,3,4,7,7*a*-hexahydro-3*aH*-isoindole-3*a*-carboxylate (17i)

By following the general procedure for debenzoylations, the reaction was carried out with 16i (64 mg, 0.10 mmol). FC furnished compound 17i (42 mg, 77%), transparent wax; [*α*]_D_ = −2.1° (c 0.24; CHCl_3_). ^1^H NMR (400 MHz, CDCl_3_) *δ* 7.39 (m, 1H, H-2′), 7.35–7.27 (m, 3H, 2× *m*-Bn, 1× Ph), 7.25 (m, 1H, *p*-Bn), 7.19–7.13 (m, 3H, 2× *o*-Bn, 1× Ph), 7.11 (m, 1H, 1× Ph), 5.91 (m, 1H, NH), 5.46 (m, 1H, H-7), 3.76 (s, 3H, COOCH_3_), 3.67 (dt, *J*_gem_ = 9.3 Hz, *J*_15b,16a_ = *J*_15b,16b_ = 6.8 Hz, 1H, H-15b), 3.54 (dt, *J*_gem_ = 9.3 Hz, *J*_15a,16a_ = *J*_15a,16b_ = 6.8 Hz, 1H, H-15a), 3.54–3.47 (m, 2H, H-14a,b), 3.25 (dt, *J*_3,10a_ = 8.8 Hz, *J*_3,4_ = *J*_3,10b_ = 4.2 Hz, 1H, H-3), 2.89 (dd, *J*_gem_ = 13.5 Hz, *J*_10b,3_ = 4.0 Hz, 1H, H-10b), 2.86–2.79 (m, 2H, H-16a,b), 2.70 (m, 1H, H-8), 2.66 (dd, *J*_gem_ = 13.5 Hz, *J*_10a,3_ = 9.2 Hz, 1H, H-10a), 2.58 (dd, *J*_4,3_ = *J*_4,5_ = 4.5 Hz, 1H, H-4), 2.46 (m, 1H, H-5), 2.15–2.05 (m, 1H, H-13b), 1.95–1.83 (m, 1H, H-13a), 1.75 (m, 3H, CH_3_-12), 1.13 (d, *J*_11,5_ = 7.3 Hz, 3H, CH_3_-11) ppm. ^13^C NMR (101 MHz, CDCl_3_) *δ* 173.67 (C-1), 173.06 (*C*OOCH_3_), 141.84 (C–*i*-Ph), 139.09 (C-6), 137.30 (C–*i*-Bn), 131.90 (CH-2′), 129.66 (CH–Ph), 129.16 (2× CH–*o*-Bn), 129.03 (CH–Ph), 128.82 (2× CH–*m*-Bn), 127.67 (CH–Ph), 126.95 (CH–*p*-Bn), 126.56 (CH-7), 122.19 (C-3′), 70.61 (CH_2_-15), 69.71 (CH_2_-14), 60.29 (C-9), 55.81 (CH-3), 53.68 (CH-4), 52.68 (COO*C*H_3_), 44.74 (CH_2_-10), 36.36 (CH-8), 35.93 (CH_2_-16), 34.23 (CH-5), 29.84 (CH_2_-13), 20.23 (CH_3_-12), 13.94 (CH_3_-11) ppm. HRMS (ESI) *m*/*z* calcd for C_29_H_35_O_4_NBr^+^ [M + H]^+^ 540.1744, found 540.1745.

##### Methyl (1*S*,3*aR*,4*S*,7*S*,7*aR*)-1-benzyl-6,7-dimethyl-4-(2-(4-nitrophenethoxy)ethyl)-3-oxo-1,2,3,4,7,7*a*-hexahydro-3*aH*-isoindole-3*a*-carboxylate (17j)

By following the general procedure for debenzoylations, the reaction was carried out with 16j (61 mg, 0.10 mmol). FC furnished compound 17j (40 mg, 78%), transparent wax; [*α*]_D_ = +1.3° (c 0.30; CHCl_3_). ^1^H NMR (400 MHz, CDCl_3_) *δ* 8.10 (m, 2H, 2× *m*-Ph), 7.39 (m, 2H, 2× *o*-Ph), 7.31 (m, 2H, 2× *m*-Bn), 7.24 (m, 1H, *p*-Bn), 7.14 (m, 2H, 2× *o*-Bn), 5.80 (bs, 1H, NH), 5.43 (m, 1H, H-7), 3.76 (s, 3H, COOCH_3_), 3.72 (dt, *J*_gem_ = 9.3 Hz, *J*_15b,16a_ = *J*_15b,16b_ = 6.4 Hz, 1H, H-15b), 3.58 (dt, *J*_gem_ = 9.3 Hz, *J*_15a,16a_ = *J*_15a,16b_ = 6.4 Hz, 1H, H-15a), 3.53–3.45 (m, 2H, H-14a,b), 3.24 (dt, *J*_3,10a_ = 9.2 Hz, *J*_3,4_ = *J*_3,10b_ = 4.2 Hz, 1H, H-3), 2.98–2.92 (m, 2H, H-16a,b), 2.89 (dd, *J*_gem_ = 13.5 Hz, *J*_10b,3_ = 4.0 Hz, 1H, H-10b), 2.68 (m, 1H, H-8), 2.65 (dd, *J*_gem_ = 13.5 Hz, *J*_10a,3_ = 9.2 Hz, 1H, H-10a), 2.56 (t, *J*_4,3_ = *J*_4,5_ = 4.4 Hz, 1H, H-4), 2.40 (m, 1H, H-5), 2.13–2.02 (m, 1H, H-13b), 1.93–1.81 (m, 1H, H-13a), 1.73 (m, 3H, CH_3_-12), 1.15 (d, *J*_11,5_ = 7.2 Hz, 3H, CH_3_-11) ppm. ^13^C NMR (101 MHz, CDCl_3_) *δ* 173.68 (C-1), 172.98 (*C*OOCH_3_), 147.67 (C–*i*-Ph), 146.45 (C–*p*-Ph), 139.16 (C-6), 137.23 (C–*i*-Bn), 129.87 (2× CH–*o*-Ph), 129.12 (2× CH–*o*-Bn), 128.90 (2× CH–*m*-Bn), 127.05 (CH–*p*-Bn), 126.50 (CH-7), 123.33 (2× CH–*m*-Ph), 70.02 (CH_2_-15), 69.82 (CH_2_-14), 60.30 (C-9), 55.97 (CH-3), 53.81 (CH-4), 52.76 (COO*C*H_3_), 44.79 (CH_2_-10), 36.37 (CH-8), 36.19 (CH_2_-16), 34.30 (CH-5), 29.87 (CH_2_-13), 20.22 (CH_3_-12), 13.94 (CH_3_-11) ppm. HRMS (ESI) *m*/*z* calcd for C_29_H_35_O_6_N_2_^+^ [M + H]^+^ 507.2495, found 507.2484.

##### Methyl (1*S*,3*aR*,7*aR*)-2-benzoyl-1-benzyl-4-(3-(benzyloxy)propyl)-6,7-dimethyl-3-oxo-1,2,3,4,7,7*a*-hexahydro-3*aH*-isoindole-3*a*-carboxylate (19)

AgOTf (217 mg, 0.85 mmol) was placed to a flask and dried under high vacuum by a heatgun (150–200 °C). Then, 2,6-di-*tert*-butylpyridine (190 μL, 0.85 mmol) followed by a solution of 14 (134 mg, 0.28 mmol) in dry DCM (1 mL) were added at room temperature under argon atmosphere. Benzyl iodide (18) (197 mg, 0.90 mmol) was added and the resulting yellow suspension was stirred at room temperature for 16 h. The reaction mixture was diluted with EtOAc (10 mL) and filtered through a pad of celite. The organics were washed with 1 M HCl (5 mL), a solution of NaHCO_3_ (5 mL), brine (5 mL), dried (Na_2_SO_4_) and concentrated under reduced pressure. Flash column chromatography of the residue on silica gel (24 g, 0–30% EtOAc in hexanes) furnished compound 19 (90 mg, 56%) as a 6.0/1 (*endo*/*exo*) diastereomeric mixture, white amorphous solid. ^1^H NMR (400 MHz, CDCl_3_): *δ* 7.68–7.56 (m, 2H, 2× *o*-Bz), 7.53–7.45 (m, 1H, *p*-Bz), 7.45–7.33 (m, 2H, 2× *m*-Bz), 7.38–7.22 (m, 8H, Ph, 2× *m*-Bn, *p*-Bn), 7.20–7.09 (m, 2H, 2× *o*-Bn), 5.43 (bs, 1H, H-7), 4.40 (s, 2H, H-16), 4.26 (dt, *J*_3,10b_ = 8.0 Hz, *J*_3,4_ = *J*_3,10b_ = 3.4 Hz, 1H, H-3), 3.62 (s, 3H, COOCH_3_), 3.46–3.29 (m, 2H, H-15), 3.08 (dd, *J*_gem_ = 13.3 Hz, *J*_10b,3_ = 3.3 Hz, 1H, H-10b), 2.92 (dd, *J*_gem_ = 13.3 Hz, *J*_10a,3_ = 3.3 Hz, 1H, H-10a), 2.69 (dd, *J*_4,5_ = 5.7 Hz, *J*_4,3_ = 3.3 Hz, 1H, H-4), 2.51–2.37 (m, 2H, H-8, H-5), 1.96–1.82 (m, 1H, H-13b), 1.72–1.56 (m, 1H *overlapped*, H-14b), 1.71 (s, 3H, CH_3_-12), 1.55–1.42 (m, 2H, H-13a, H-14a), 0.82 (d, *J*_11,5_ = 7.4 Hz, 3H, CH_3_-11) ppm. ^13^C NMR (101 MHz, CDCl_3_): *δ* 172.67 (C=O), 172.39 (C=O), 170.31 (C=O–Bz), 140.21 (C-6), 138.54 (C–*i*-Ph), 136.07 (C–*i*-Bn), 134.46 (C–*i*-Bz), 132.17 (CH–Ar), 130.49 (CH–Ar), 128.98 (CH–Ar), 128.43 (CH–Ar), 128.24 (CH–Ar), 127.92 (CH–Ar), 127.58 (CH–Ar), 127.38 (CH–Ar), 126.94 (CH–Ar), 126.83 (CH-7), 72.82 (CH_2_-16), 70.19 (CH_2_-15), 63.05 (C-9), 57.31 (CH-3), 52.88 (COO*C*H_3_), 47.29 (CH-4), 41.18 (CH-8), 39.83 (CH_2_-10), 34.18 (CH-5), 29.20 (CH_2_-14), 26.14 (CH_2_-13), 19.76 (CH_3_-12), 12.99 (CH_3_-11) ppm. NMR spectra of the minor isomer were not assigned due to overlapping signals. HRMS (ESI) *m*/*z*: [M + H]^+^ calcd for C_36_H_40_O_5_N^+^ 566.2901; found 566.2903.

##### Methyl (1*S*,3*aR*,4*S*,7*S*,7*aR*)-1-benzyl-4-(3-(benzyloxy)propyl)-6,7-dimethyl-3-oxo-1,2,3,4,7,7*a*-hexahydro-3*aH*-isoindole-3*a*-carboxylate (20)

A solution of KOH (71 mg, 1.27 mmol) in H_2_O (0.4 mL) was added dropwise to a stirred solution of benzyl-protected compound 19 (90 mg, 0.16 mmol) in toluene (3 mL) and MeOH (1 mL). The reaction mixture was vigorously stirred at room temperature for 24 h. Then, reaction mixture was diluted with EtOAc, extracted with water and the aqueous phase was extracted with EtOAc (3 × 5 ml). The organics were combined, washed with brine (5 mL), dried (Na_2_SO_4_) and concentrated under reduced pressure. Flash column chromatography of the residue on silica gel (0–40% EtOAc in hexanes) furnished the partially purified product (13 mg) that was further purified using HPLC (prontosil SiO_2_ column 150 × 22 mm, 20–70% EtOAc in heptane) to obtain unprotected cytochalasin analogue 20 (6.6 mg, 14%) as a single diastereomer, transparent amorphous solid. [*α*]_D_ = −10.6° (c 0.16; CHCl_3_). ^1^H NMR (400 MHz, CDCl_3_) *δ* 7.38–7.28 (m, 6H, Ph/Bn), 7.30–7.22 (m, 2H, Ph/Bn), 7.20–7.12 (m, 2H, Ph/Bn), 5.59 (d, *J* = 7.8 Hz, NH), 5.49 (bs, 1H, H-7), 4.49 (s, 2H, H-16), 3.77 (s, 3H, COOCH_3_), 3.55–3.42 (m, 2H, H-15), 3.23 (dt, *J*_3,10a_ = 8.9 Hz, *J*_3,4_ = *J*_3,10b_ = 4.0 Hz, 1H, H-3), 2.89 (dd, *J*_gem_ = 13.4 Hz, *J*_10b,3_ = 3.8 Hz, 1H, H-10b), 2.68–2.53 (m, 3H, H-4, H-8, H-10a), 2.52–2.45 (m, 1H, H-5), 1.82–1.71 (m, 3H *overlapped*, 2× H-14, H-13b), 1.76 (s, 3H, CH_3_-12), 1.67–1.58 (m, 1H, H-13a), 1.16 (d, *J*_11,5_ = 7.2 Hz, 3H, CH_3_-11) ppm. ^13^C NMR (101 MHz, CDCl_3_) *δ* 173.39 (C=O), 173.36 (C=O), 139.05 (C-6), 138.66 (C–*i*-Ph), 137.41 (C–*i*-Bn), 129.08 (CH–Ar), 128.89 (CH–Ar), 128.26 (CH–Ar), 127.63 (CH–Ar), 127.35 (CH–Ar), 127.00 (CH–Ar/CH-7), 126.97 (CH–Ar/CH-7), 72.83 (CH_2_-16), 70.49 (CH_2_-15), 60.40 (C-9), 55.70 (CH-3), 54.05 (CH-4), 52.73 (COO*C*H_3_), 44.95 (CH_2_-10), 39.57 (CH-8), 34.23 (CH-5), 29.29 (CH_2_-14), 26.67 (CH_2_-13), 20.26 (CH_3_-12), 14.06 (CH_3_-11) ppm. HRMS (ESI) *m*/*z*: [M + H]^+^ calcd for C_29_H_37_O_4_N^+^ 462.2639; found 462.2638. MS (ESI) *m*/*z* (%): 462 (38) [M + H], 484 (100) [M + Na], 945 (18) [2M + Na].

#### General procedure for epoxidations

A solution of *m*CPBA (29.6 mg, 0.12 mmol, 70% (w/w)) in DCM (0.8 mL) was dried with MgSO_4_ (20 min). Filtered dry solution of *m*CPBA was added to the solution of 16 (0.10 mmol) in DCM (0.5 mL) at 0 °C. The reaction mixture was stirred at 0 °C for 1 h, then at room temperature until full conversion (2 h, TLC). Then, the reaction mixture was diluted with DCM (10 mL), washed with a solution of NaHCO_3_ (2 × 5 mL), brine (5 mL), dried (MgSO_4_) and concentrated under reduced pressure. Column chromatography of the residue on silica gel (20% EtOAc in hexanes) furnished corresponding epoxides 21.

##### Methyl (1*aS*,2*R*,2*aS*,5*S*,5*aR*,6*S*,6*aR*)-4-benzoyl-5-benzyl-6,6*a*-dimethyl-3-oxo-2-(2-phenethoxyethyl)octahydro-2*aH*-oxireno[2,3-*f*]isoindole-2*a*-carboxylate (21a)

By following the general procedure for epoxidations, the reaction was carried out with 16a (56.7 mg, 0.10 mmol). FC furnished compound 21a (41.9 mg, 72%); white wax; and small amount of phenethyl alcohol as an impurity [*α*]_D_ = +89.8° (c 0.31; CHCl_3_). ^1^H NMR (400 MHz, CDCl_3_) *δ* 7.76 (m, 2H, 2× *o*-Bz), 7.58 (m, 1H, *p*-Bz), 7.48 (m, 2H, 2× *m*-Bz), 7.36–7.15 (m, 10H, Ph, Bn), 4.66 (m, 1H, H-3), 3.87 (s, 3H, COOCH_3_), 3.62 (dt, *J*_gem_ = 9.3 Hz, *J*_15b,14a_ = *J*_15b,14b_ = 7.2 Hz, 1H, H-15b), 3.60–3.46 (m, 3H *overlapped*, H-15a, H-14a,b), 3.22 (dd, *J*_gem_ = 13.0 Hz, *J*_10b,3_ = 3.6 Hz, 1H, H-10b), 2.86–2.83 (m, 2H *overlapped*, H-16a,b), 2.71 (d, *J*_7,8_ = 5.7 Hz, 1H, H-7), 2.67–2.61 (m, 2H *overlapped*, H-10a, H-4), 2.45–2.36 (m, 1H, H-13b), 2.15–2.10 (m, 1H, H-8), 1.78–1.67 (m, 2H *overlapped*, H-13a, H-5), 1.16 (s, 3H, CH_3_-12), 0.63 (d, *J*_11,5_ = 7.3 Hz, 3H, CH_3_-11) ppm. ^13^C NMR (101 MHz, CDCl_3_) *δ* 172.56 (*C*OOCH_3_), 171.66 (C-1), 170.28 (C=O–Bz), 139.10 (C–*i*-Ph), 135.95 (C–*i*-Bn), 133.94 (C–*i*-Bz), 132.71 (CH–*p*-Bz), 129.91 (2× CH–*o*-Bn), 129.12 (2× CH–Ar), 128.95 (2× CH–*o*-Bz), 128.70 (2× CH–Ar), 128.19 (2× CH–Ar), 128.15 (2× CH–Ar), 127.18 (CH–*p*-Bn), 125.98 (CH–*p*-Ph), 71.83 (CH_2_-15), 68.64 (CH_2_-14), 61.59 (CH-7), 60.99 (C-9), 56.29 (C-6), 56.11 (CH-3), 53.20 (COO*C*H_3_), 45.31 (CH-4), 40.70 (CH_2_-10), 39.35 (CH-8), 36.25 (CH_2_-16), 36.01 (CH-5), 29.17 (CH_2_-13), 18.71 (CH_3_-12), 11.98 (CH_3_-11) ppm. HRMS (ESI) *m*/*z* calcd for C_29_H_36_O_5_N^+^ [M + H]^+^ 478.2588, found 478.2587.

##### Methyl (1*aS*,2*R*,2*aS*,5*S*,5*aR*,6*S*,6*aR*)-4-benzoyl-5-benzyl-2-(2-(4-methoxyphenethoxy)ethyl)-6,6*a*-dimethyl-3-oxooctahydro-2*aH*-oxireno[2,3-*f*]isoindole-2*a*-carboxylate (21b)

By following the general procedure for epoxidations, the reaction was carried out with 16b (59.6 mg, 0.10 mmol). FC furnished epoxide 21b (33.0 mg, 54%); white wax; [*α*]_D_ = +86.6° (c 0.25; CHCl_3_). ^1^H NMR (400 MHz, CDCl_3_) *δ* 7.75 (m, 2H, 2× *o*-Bz), 7.57 (m, 1H, *p*-Bz), 7.47 (m, 2H, 2× *m*-Bz), 7.33 (m, 2H, 2× *m*-Bn), 7.27 (m, 1H, *p*-Bn), 7.17 (m, 2H, 2× *o*-Bn), 7.11 (m, 2H, 2× *o*-Ph), 6.79 (m, 2H, 2× *m*-Ph), 4.65 (ddd, *J*_3,10a_ = 10.2 Hz, *J*_3,10b_ = 3.6 Hz, *J*_3,4_ = 2.0 Hz, 1H, H-3), 3.87 (s, 3H, COOCH_3_), 3.75 (s, 3H, OCH_3_), 3.60–3.45 (m, 4H *overlapped*, H-15a,b, H-14a,b), 3.21 (dd, *J*_gem_ = 13.0 Hz, *J*_10b,3_ = 3.6 Hz, 1H, H-10b), 2.79–2.76 (m, 2H *overlapped*, H-16a,b), 2.70 (d, *J*_7,8_ = 5.8 Hz, 1H, H-7), 2.64 (dd, *J*_4,5_ = 6.8 Hz, *J*_4,3_ = 2.0 Hz, 1H, H-4), 2.63 (dd, *J*_gem_ = 13.0 Hz, *J*_10a,3_ = 10.2 Hz, 1H, H-10a), 2.44–2.35 (m, 1H, H-13b), 2.13–2.08 (m, 1H, H-8), 1.76–1.66 (m, 2H *overlapped*, H-13a, H-5), 1.15 (s, 3H, CH_3_-12), 0.62 (d, *J*_11,5_ = 7.3 Hz, 3H, CH_3_-11) ppm. ^13^C NMR (101 MHz, CDCl_3_) *δ* 172.58 (*C*OOCH_3_), 171.68 (C-1), 170.30 (C=O–Bz), 157.91 (C–*p*-Ph), 135.98 (C–*i*-Bn), 133.97 (C–*i*-Bz), 132.72 (CH–*p*-Bz), 131.19 (C–*i*-Ph), 129.93 (2× CH–*o*-Bn), 129.88 (2× CH–*o*-Ph), 129.13 (2× CH–*o*-Bz), 128.72 (2× CH–*m*-Bn), 128.17 (2× CH–*m*-Bz), 127.19 (CH–*p*-Bn), 113.61 (2× CH–*m*-Ph), 72.10 (CH_2_-15), 68.64 (CH_2_-14), 61.61 (CH-7), 61.01 (C-9), 56.31 (C-6), 56.12 (CH-3), 55.18 (OCH_3_), 53.21 (COO*C*H_3_), 45.35 (CH-4), 40.72 (CH_2_-10), 39.36 (CH-8), 36.04 (CH-5), 35.35 (CH_2_-16), 29.18 (CH_2_-13), 18.73 (CH_3_-12), 11.99 (CH_3_-11) ppm. HRMS (ESI) *m*/*z* calcd for C_37_H_41_O_7_NNa^+^ [M + Na]^+^ 634.2775, found 634.2770.

##### Methyl (1*aS*,2*R*,2*aS*,5*S*,5*aR*,6*S*,6*aR*)-4-benzoyl-5-benzyl-2-(2-(4-bromophenethoxy)ethyl)-6,6*a*-dimethyl-3-oxooctahydro-2*aH*-oxireno[2,3-*f*]isoindole-2*a*-carboxylate (21h)

By following the general procedure for epoxidations, the reaction was carried out with 16h (64.5 mg, 0.10 mmol). FC furnished epoxide 21h (41.6 mg, 63%); white wax; [*α*]_D_ = +73.7° (c 0.34; CHCl_3_). ^1^H NMR (400 MHz, CDCl_3_) *δ* 7.75 (m, 2H, 2× *o*-Bz), 7.58 (m, 1H, *p*-Bz), 7.47 (m, 2H, 2× *m*-Bz), 7.35 (m, 2H, 2× *m*-Ph), 7.33 (m, 2H, 2× *m*-Bn), 7.27 (m, 1H, *p*-Bn), 7.17 (m, 2H, 2× *o*-Bn), 7.07 (m, 2H, 2× *o*-Ph), 4.65 (ddd, *J*_3,10a_ = 10.0 Hz, *J*_3,10b_ = 3.6 Hz, *J*_3,4_ = 2.0 Hz, 1H, H-3), 3.86 (s, 3H, COOCH_3_), 3.60 (dt, *J*_gem_ = 9.2 Hz, *J*_15b,14a_ = *J*_15b,14b_ = 6.6 Hz, 1H, H-15b), 3.57–3.42 (m, 3H *overlapped*, H-15a, H-14a,b), 3.20 (dd, *J*_gem_ = 13.0 Hz, *J*_10b,3_ = 3.6 Hz, 1H, H-10b), 2.79–2.76 (m, 2H *overlapped*, H-16a,b), 2.67 (d, *J*_7,8_ = 5.7 Hz, 1H, H-7), 2.64 (dd, *J*_4,5_ = 6.6 Hz, *J*_4,3_ = 2.0 Hz, 1H, H-4), 2.63 (dd, *J*_gem_ = 13.0 Hz, *J*_10a,3_ = 10.0 Hz, 1H, H-10a), 2.42–2.33 (m, 1H, H-13b), 2.08–2.03 (m, 1H, H-8), 1.71–1.63 (m, 2H *overlapped*, H-13a, H-5), 1.15 (s, 3H, CH_3_-12), 0.63 (d, *J*_11,5_ = 7.3 Hz, 3H, CH_3_-11) ppm. ^13^C NMR (101 MHz, CDCl_3_) *δ* 172.54 (*C*OOCH_3_), 171.70 (C-1), 170.27 (C=O–Bz), 138.32 (C–*i*-Ph), 135.93 (C–*i*-Bn), 133.94 (C–*i*-Bz), 132.74 (CH–*p*-Bz), 131.17 (2× CH–*m*-Ph), 130.80 (2× CH–*o*-Ph), 129.93 (2× CH–*o*-Bn), 129.13 (2× CH–*o*-Bz), 128.74 (2× CH–*m*-Bn), 128.17 (2× CH–*m*-Bz), 127.20 (CH–*p*-Bn), 119.78 (C–*p*-Ph), 71.35 (CH_2_-15), 68.62 (CH_2_-14), 61.54 (CH-7), 60.98 (C-9), 56.30 (C-6), 56.12 (CH-3), 53.21 (COO*C*H_3_), 45.40 (CH-4), 40.73 (CH_2_-10), 39.31 (CH-8), 36.07 (CH-5), 35.67 (CH_2_-16), 29.14 (CH_2_-13), 18.71 (CH_3_-12), 12.01 (CH_3_-11) ppm. HRMS (ESI) *m*/*z* calcd for C_36_H_38_O_6_NBrNa^+^ [M + Na]^+^ 682.1775, found 682.1770.

##### Methyl (1*aS*,2*R*,2*aS*,5*S*,5*aR*,6*S*,6*aR*)-5-benzyl-6,6*a*-dimethyl-3-oxo-2-(2-phenethoxyethyl)octahydro-2*aH*-oxireno[2,3-*f*]isoindole-2*a*-carboxylate (22a)

By following the general procedure for debenzoylations, the reaction was carried out with 21a (58.2 mg, 0.10 mmol). FC furnished epoxide 22a (34.9 mg, 73%); white wax; [*α*]_D_ = −11.4° (c 0.18; CHCl_3_). ^1^H NMR (400 MHz, CDCl_3_) *δ* 7.34 (m, 2H, 2× *m*-Bn), 7.30–7.18 (m, 6H, Ph, *p*-Bn), 7.15 (m, 2H, 2× *o*-Bn), 5.76 (bs, 1H, NH), 3.82 (s, 3H, COOCH_3_), 3.75–3.51 (m, 5H *overlapped*, H-15a,b, H-14a,b, H-3), 2.92–2.84 (m, 2H, H-16a,b), 2.83 (d, *J*_7,8_ = 5.4 Hz, 1H, H-7), 2.82 (dd, *J*_gem_ = 13.6 Hz, *J*_10b,3_ = 5.2 Hz, 1H, H-10b), 2.73 (dd, *J*_gem_ = 13.6 Hz, *J*_10a,3_ = 8.4 Hz, 1H, H-10a), 2.70 (dd, *J*_4,5_ = 7.3 Hz, *J*_4,3_ = 4.0 Hz, 1H, H-4), 2.46 (ddd, *J*_8,13b_ = 11.1 Hz, *J*_8,7_ = 5.4 Hz, *J*_8,13a_ = 3.0 Hz, 1H, H-8), 2.17 (dm, *J*_gem_ = 14.0 Hz, 1H, H-13b), 2.01 (dq, *J*_5,4_ = *J*_5,11_ = 7.3 Hz, 1H, H-5), 1.89 (dtd, *J*_gem_ = 14.0 Hz, *J*_13a,14_ = 7.0 Hz, *J*_13a,8_ = 3.0 Hz, 1H, H-13a), 1.23 (s, 3H, CH_3_-12), 1.02 (d, *J*_11,5_ = 7.3 Hz, 3H, CH_3_-11) ppm. ^13^C NMR (101 MHz, CDCl_3_) *δ* 173.38 (C-1), 172.74 (*C*OOCH_3_), 139.11 (C–*i*-Ph), 137.03 (C–*i*-Bn), 129.10 (2× CH–Ar), 129.01 (2× CH–Ar), 128.95 (2× CH–Ar), 128.25 (2× CH–Ar), 127.19 (CH–*p*-Bn), 126.03 (CH–*p*-Ph), 71.87 (CH_2_-15), 69.31 (CH_2_-14), 61.39 (CH-7), 57.55 (C-9), 56.87 (C-6), 54.67 (CH-3), 52.99 (COO*C*H_3_), 48.68 (CH-4), 44.46 (CH_2_-10), 38.39 (CH-8), 36.32 (CH_2_-16), 34.80 (CH-5), 29.46 (CH_2_-13), 20.18 (CH_3_-12), 13.31 (CH_3_-11) ppm. HRMS (ESI) *m*/*z* calcd for C_29_H_36_O_5_N^+^ [M + H]^+^ 478.2588, found 478.2587.

##### Methyl (1*aS*,2*R*,2*aS*,5*S*,5*aR*,6*S*,6*aR*)-5-benzyl-2-(2-(4-methoxyphenethoxy)ethyl)-6,6*a*-dimethyl-3-oxooctahydro-2*aH*-oxireno[2,3-*f*]isoindole-2*a*-carboxylate (22b)

By following the general procedure for debenzoylations, the reaction was carried out with 21b (61.2 mg, 0.10 mmol). FC furnished epoxide 22b (36.6 mg, 72%); white wax; [*α*]_D_ = −14.6° (c 0.32; CHCl_3_). ^1^H NMR (400 MHz, CDCl_3_) *δ* 7.34 (m, 2H, 2× *m*-Bn), 7.27 (m, 1H, *p*-Bn), 7.16–7.12 (m, 4H *overlapped*, 2× *o*-Ph, 2× *o*-Bn), 6.81 (m, 2H, 2× *m*-Ph), 5.88 (bs, 1H, NH), 3.82 (s, 3H, COOCH_3_), 3.77 (s, 3H, OCH_3_), 3.67–3.52 (m, 5H *overlapped*, H-15a,b, H-14a,b, H-3), 2.83–2.71 (m, 5H *overlapped*, H-16a,b, H-10a,b, H-7), 2.71 (dd, *J*_4,5_ = 7.3 Hz, *J*_4,3_ = 3.8 Hz, 1H, H-4), 2.45 (ddd, *J*_8,13b_ = 11.1 Hz, *J*_8,7_ = 5.4 Hz, *J*_8,13a_ = 3.0 Hz, 1H, H-8), 2.17 (dm, *J*_gem_ = 13.9 Hz, 1H, H-13b), 1.99 (dq, *J*_5,11_ = *J*_5,4_ = 7.3 Hz, 1H, H-5), 1.88 (dm, *J*_gem_ = 13.9 Hz, 1H, H-13a), 1.23 (s, 3H, CH_3_-12), 1.00 (d, *J*_11,5_ = 7.3 Hz, 3H, CH_3_-11) ppm. ^13^C NMR (101 MHz, CDCl_3_) *δ* 173.44 (C-1), 172.74 (*C*OOCH_3_), 157.95 (C–*p*-Ph), 137.00 (C–*i*-Bn), 131.18 (C–*i*-Ph), 129.85 (2× CH–Ar), 129.12 (2× CH–Ar), 128.98 (2× CH–Ar), 127.16 (CH–*p*-Bn), 113.69 (2× CH–*m*-Ph), 72.12 (CH_2_-15), 69.29 (CH_2_-14), 61.38 (CH-7), 57.56 (C-9), 56.86 (C-6), 55.20 (OCH_3_), 54.66 (CH-3), 52.97 (COO*C*H_3_), 48.59 (CH-4), 44.39 (CH_2_-10), 38.39 (CH-8), 35.40 (CH_2_-16), 34.80 (CH-5), 29.46 (CH_2_-13), 20.16 (CH_3_-12), 13.28 (CH_3_-11) ppm. HRMS (ESI) *m*/*z* calcd for C_30_H_37_O_6_NNa^+^ [M + Na]^+^ 530.2513, found 530.2508.

##### Methyl (1*aS*,2*R*,2*aS*,5*S*,5*aR*,6*S*,6*aR*)-5-benzyl-2-(2-(4-bromophenethoxy)ethyl)-6,6*a*-dimethyl-3-oxooctahydro-2*aH*-oxireno[2,3-*f*]isoindole-2*a*-carboxylate (22h)

By following the general procedure for debenzoylations, the reaction was carried out with 21h (66.1 mg, 0.10 mmol). FC furnished epoxide 22h (51.2 mg, 92%); white wax; [*α*]_D_ = −14.1° (c 0.35; CHCl_3_). ^1^H NMR (400 MHz, CDCl_3_) *δ* 7.38 (m, 2H, 2× *m*-Ph), 7.33 (m, 2H, 2× *m*-Bn), 7.27 (m, 1H, *p*-Bn), 7.15 (m, 2H, 2× *o*-Bn), 7.10 (m, 2H, 2× *o*-Ph), 5.78 (bs, 1H, NH), 3.82 (s, 3H, COOCH_3_), 3.69–3.49 (m, 5H *overlapped*, H-15a,b, H-14a,b, H-3), 2.85–2.80 (m, 2H, H-16a,b), 2.81–2.70 (m, 3H *overlapped*, H-10a,b, H-7), 2.67 (dd, *J*_4,5_ = 7.2 Hz, *J*_4,3_ = 3.5 Hz, 1H, H-4), 2.38 (ddd, *J*_8,13b_ = 11.1 Hz, *J*_8,7_ = 5.5 Hz, *J*_8,13a_ = 3.0 Hz, 1H, H-8), 2.18 (ddt, *J*_gem_ = 13.9 Hz, *J*_13b,8_ = 11.1 Hz, *J*_13b,14a_ = *J*_13b,14b_ = 5.6 Hz, 1H, H-13b), 1.94 (dq, *J*_5,11_ = *J*_5,4_ = 7.3 Hz, 1H, H-5), 1.86 (dm, *J*_gem_ = 14.0 Hz, 1H, H-13a), 1.22 (s, 3H, CH_3_-12), 1.01 (d, *J*_11,5_ = 7.3 Hz, 3H, CH_3_-11) ppm. ^13^C NMR (101 MHz, CDCl_3_) *δ* 173.39 (C-1), 172.75 (*C*OOCH_3_), 138.31 (C–*i*-Ph), 136.97 (C–*i*-Bn), 131.23 (2× CH–*m*-Ph), 130.79 (2× CH–*o*-Ph), 129.12 (2× CH–*o*-Bn), 129.01 (2× CH–*m*-Bn), 127.20 (CH–*p*-Bn), 119.82 (C–*p*-Ph), 71.37 (CH_2_-15), 69.25 (CH_2_-14), 61.33 (CH-7), 57.50 (C-9), 56.74 (C-6), 54.57 (CH-3), 52.99 (COO*C*H_3_), 49.04 (CH-4), 44.48 (CH_2_-10), 38.42 (CH-8), 35.71 (CH_2_-16), 34.99 (CH-5), 29.41 (CH_2_-13), 20.08 (CH_3_-12), 13.23 (CH_3_-11) ppm. HRMS (ESI) *m*/*z* calcd for C_29_H_34_O_5_NBrNa^+^ [M + Na]^+^ 578.1513, found 578.1508.

#### General procedure for dihydroxylations

K_2_OsO_2_(OH)_4_ (1.8 mg, 0.05 mmol) was added to a solution of 17 (0.10 mmol) in acetone/H_2_O (9/1, 1.0 mL), followed by addition of NMO (17.6 mg, 0.15 mmol) at room temperature. The reaction mixture was vigorously stirred at RT until full conversion (*ca.* 14 h, TLC). Then, H_2_O (0.5 mL) and Na_2_S_2_O_5_ (*ca.* 50 mg) were added and the reaction mixture was stirred 30 min. After filtration through a small pad of celite, filtrate was concentrated under reduced pressure. Column chromatography of the residue on silica gel (15–70% EtOAc in hexanes) furnished corresponding diols 23.

##### Methyl (1*S*,3*aS*,4*R*,5*S*,6*R*,7*S*,7*aR*)-1-benzyl-5,6-dihydroxy-6,7-dimethyl-3-oxo-4-(2-phenethoxyethyl)octahydro-3*aH*-isoindole-3*a*-carboxylate (23a)

By following the general procedure for dihydroxylations, the reaction was carried out with 17a (46.2 mg, 0.10 mmol). FC furnished diol 23a (31.7 mg, 64%); beige solid; [*α*]_D_ = −17.5° (c 0.27; CHCl_3_). ^1^H NMR (500 MHz, CDCl_3_) *δ* 7.36–7.21 (m, 8H, Ph, 2× *m*-Bn, *p*-Bn), 7.16 (m, 2H, 2× *o*-Bn), 5.61 (bs, 1H, NH), 5.04 (d, *J*_CHOH,7_ = 4.9 Hz, 1H, CHO*H*), 3.77 (s, 3H, COOCH_3_), 3.75–3.71 (m, 2H, H-15a,b), 3.68 (dt, *J*_gem_ = 8.9 Hz, *J*_15b,16a_ = *J*_15b,16b_ = 3.4 Hz, 1H, H-14b), 3.60 (ddd, *J*_3,10a_ = 9.6 Hz, *J*_3,4_ = 5.4 Hz, *J*_3,10b_ = 3.5 Hz, 1H, H-3), 3.43 (m, 1H, H-14a), 3.06 (dd, *J*_7,8_ = 12.2 Hz, *J*_7,CHOH_ = 4.9 Hz, 1H, H-7), 2.96 (dd, *J*_gem_ = 13.5 Hz, *J*_10b,3_ = 3.6 Hz, 1H, H-10b), 2.91–2.89 (m, 2H, H-16a,b), 2.61 (bs, 1H, COH), 2.58 (dd, *J*_gem_ = 13.5 Hz, *J*_10a,3_ = 9.6 Hz, 1H, H-10a), 2.53 (ddd, *J*_8,7_ = 12.2 Hz, *J*_8,13a_ = 7.2 Hz, *J*_8,13b_ = 1.5 Hz, 1H, H-8), 2.46 (t, *J*_4,3_ = *J*_4,5_ = 5.4 Hz, 1H, H-4), 2.27 (dm, *J*_gem_ = 16.0 Hz, 1H, H-13b), 2.20–2.14 (m, 1H, H-5), 1.84–1.76 (m, 1H, H-13a), 1.20 (s, 3H, CH_3_-12), 1.11 (d, *J*_11,5_ = 7.3 Hz, 3H, CH_3_-11) ppm. ^13^C NMR (126 MHz, CDCl_3_) *δ* 172.95 (C-1), 172.74 (*C*OOCH_3_), 138.25 (C–*i*-Ph), 137.20 (C–*i*-Bn), 129.09 (2× CH–Ar), 128.95 (2× CH–Ar), 128.89 (2× CH–*o*-Ph), 128.46 (2× CH–Ar), 127.23 (CH–*p*-Bn), 126.41 (CH–*p*-Ph), 73.46 (CH-7), 72.38 (C-6), 72.20 (CH_2_-15), 71.80 (CH_2_-14), 59.15 (C-9), 54.66 (CH-3), 52.93 (COO*C*H_3_), 49.47 (CH-4), 45.33 (CH_2_-10), 38.40 (CH-8), 37.45 (CH-5), 36.06 (CH_2_-16), 27.67 (CH_2_-13), 24.97 (CH_3_-12), 14.14 (CH_3_-11) ppm. HRMS (ESI) *m*/*z* calcd for C_29_H_37_O_6_NNa^+^ [M + Na]^+^ 518.2513, found 518.2506.

##### Methyl (1*S*,3*aS*,4*R*,5*S*,6*R*,7*S*,7*aR*)-1-benzyl-5,6-dihydroxy-4-(2-(4-methoxyphenethoxy)ethyl)-6,7-dimethyl-3-oxooctahydro-3*aH*-isoindole-3*a*-carboxylate (23b)

By following the general procedure for dihydroxylations, the reaction was carried out with 17b (49.2 mg, 0.10 mmol). FC furnished diol 23b (31.6 mg, 60%); off-white wax; [*α*]_D_ = −57.4° (c 0.28; CHCl_3_). ^1^H NMR (400 MHz, CDCl_3_) *δ* 7.33 (m, 2H, 2× *m*-Bn), 7.28 (m, 1H, *p*-Bn), 7.28 (m, 2H, 2× *o*-Bn), 7.12 (m, 2H, 2× *o*-Ph), 6.84 (m, 2H, 2× *m*-Ph), 5.85 (bs, 1H, NH), 5.12 (bs, 1H, CHO*H*), 3.78 (s, 3H, OCH_3_), 3.75 (s, 3H, COOCH_3_), 3.71–3.66 (m, 3H, H-15a,b, H-14b), 3.61 (ddd, *J*_3,10a_ = 9.4 Hz, *J*_3,4_ = 5.0 Hz, *J*_3,10b_ = 3.6 Hz, 1H, H-3), 3.42 (m, 1H, H-14a), 3.07 (dd, *J*_7,8_ = 12.2 Hz, 1H, H-7), 2.96 (dd, *J*_gem_ = 13.5 Hz, *J*_10b,3_ = 3.6 Hz, 1H, H-10b), 2.85–2.81 (m, 2H, H-16a,b), 2.59 (dd, *J*_gem_ = 13.5 Hz, *J*_10a,3_ = 9.4 Hz, 1H, H-10a), 2.54 (ddd, *J*_8,7_ = 12.2 Hz, *J*_8,13a_ = 7.1 Hz, *J*_8,13b_ = 1.4 Hz, 1H, H-8), 2.46 (t, *J*_4,3_ = *J*_4,5_ = 5.0 Hz, 1H, H-4), 2.25 (dm, *J*_gem_ = 15.6 Hz, 1H, H-13b), 2.17 (qd, *J*_5,11_ = 7.3 Hz, *J*_5,4_ = 5.0 Hz, 1H, H-5), 1.79 (dddd, *J*_gem_ = 15.6 Hz, *J*_13a,14a_ = 11.2 Hz, *J*_13a,8_ = 7.1 Hz, *J*_13a,14b_ = 3.5 Hz, 1H, H-13a), 1.21 (s, 3H, CH_3_-12), 1.10 (d, *J*_11,5_ = 7.3 Hz, 3H, CH_3_-11) ppm (*H from COH is missing*). ^13^C NMR (101 MHz, CDCl_3_) *δ* 173.05 (C-1), 172.73 (*C*OOCH_3_), 158.17 (C–*p*-Ph), 137.12 (C–*i*-Bn), 130.20 (C–*i*-Ph), 129.79 (2× CH–*o*-Ph), 129.02 (2× CH–Bn), 129.00 (2× CH–Bn), 127.16 (CH–*p*-Bn), 113.86 (2× CH–*m*-Ph), 73.43 (CH-7), 72.43 (CH_2_-15), 72.37 (C-6), 71.71 (CH_2_-14), 59.14 (C-9), 55.22 (OCH_3_), 54.63 (CH-3), 52.88 (COO*C*H_3_), 49.27 (CH-4), 45.17 (CH_2_-10), 38.33 (CH-8), 37.42 (CH-5), 35.12 (CH_2_-16), 27.67 (CH_2_-13), 24.92 (CH_3_-12), 14.07 (CH_3_-11) ppm. HRMS (ESI) *m*/*z* calcd for C_30_H_39_O_7_NNa^+^ [M + Na]^+^ 548.2619, found 548.2617.

##### Methyl (1*S*,3*aS*,4*R*,5*S*,6*R*,7*S*,7*aR*)-1-benzyl-4-(2-(4-bromophenethoxy)ethyl)-5,6-dihydroxy-6,7-dimethyl-3-oxooctahydro-3*aH*-isoindole-3*a*-carboxylate (23h)

By following GP7, the reaction was carried out with 17h (54.1 mg, 0.10 mmol). FC furnished 23h (35.7 mg, 62%); off-white wax; [*α*]_D_ = −7.9° (c 0.37; CHCl_3_). ^1^H NMR (400 MHz, CDCl_3_) *δ* 7.41 (m, 2H, 2× *m*-Ph), 7.34 (m, 2H, 2× *m*-Bn), 7.27 (m, 1H, *p*-Bn), 7.16 (m, 2H, 2× *o*-Bn), 7.08 (m, 2H, 2× *o*-Ph), 5.67 (bs, 1H, NH), 4.97 (d, *J*_CHOH,7_ = 4.5 Hz, 1H, CHO*H*), 3.76 (s, 3H, COOCH_3_), 3.72–3.65 (m, 3H *overlapped*, H-15a,b, H-14b), 3.61 (ddd, *J*_3,10a_ = 9.3 Hz, *J*_3,4_ = 5.3 Hz, *J*_3,10b_ = 3.6 Hz, 1H, H-3), 3.46–3.40 (m, 1H, H-14a), 3.07 (dd, *J*_7,8_ = 12.0 Hz, *J*_7,CHOH_ = 4.5 Hz, 1H, H-7), 2.97 (dd, *J*_gem_ = 13.5 Hz, *J*_10b,3_ = 3.6 Hz, 1H, H-10b), 2.86–2.83 (m, 2H, H-16a,b), 2.68 (bs, 1H, COH), 2.59 (dd, *J*_gem_ = 13.5 Hz, *J*_10a,3_ = 9.5 Hz, 1H, H-10a), 2.53 (dm, *J*_8,7_ = 12.0 Hz, 1H, H-8), 2.47 (t, *J*_4,3_ = *J*_4,5_ = 5.3 Hz, 1H, H-4), 2.27 (dm, *J*_gem_ = 16.0 Hz, 1H, H-13b), 2.18 (dd, *J*_5,11_ = 7.3 Hz, *J*_5,4_ = 5.3 Hz, 1H, H-5), 1.79 (dm, *J*_gem_ = 16.0 Hz, 1H, H-13a), 1.22 (s, 3H, CH_3_-12), 1.12 (d, *J*_11,5_ = 7.3 Hz, 3H, CH_3_-11) ppm. ^13^C NMR (101 MHz, CDCl_3_) *δ* 172.97 (C-1), 172.72 (*C*OOCH_3_), 137.24 (C–*i*-Ph), 137.14 (C–*i*-Bn), 131.53 (2× CH–*m*-Ph), 130.61 (2× CH–*o*-Ph), 129.06 (2× CH–*m*-Bn), 128.99 (2× CH–*o*-Bn), 127.22 (CH–*p*-Bn), 120.27 (C–*p*-Ph), 73.44 (CH-7), 72.38 (C-6), 71.82 (CH_2_), 71.80 (CH_2_), 59.10 (C-9), 54.65 (CH-3), 52.93 (COO*C*H_3_), 49.35 (CH-4), 45.24 (CH_2_-10), 38.32 (CH-8), 37.44 (CH-5), 35.43 (CH_2_-16), 27.64 (CH_2_-13), 24.95 (CH_3_-12), 14.09 (CH_3_-11) ppm. HRMS (ESI) *m*/*z* calcd for C_29_H_36_O_6_NBrNa^+^ [M + Na]^+^ 596.1618, found 596.1613.

#### Synthesis of allylic alcohols

Method A: HCl in Et_2_O (12 mL, 12 mmol, 1 M) was added dropwise to a chilled flask with 22a (95.6 mg, 0.20 mmol) at 0 °C under Ar atmosphere. The reaction mixture was stirred at 0 °C for 1 h, then at room temperature until full conversion (*ca.* 2 h, TLC). Then, reaction mixture was poured to a solution of NaHCO_3_ (20 mL, gas release), the resulting solution was extracted with EtOAc (3 × 5 ml). The organics were combined, washed with brine (5 mL), dried (MgSO_4_), filtered through a small pad of silica gel and concentrated under reduced pressure. HPLC separation of the residue on silica gel (30–60% EtOAc in heptane) furnished corresponding products 24, 26 and slightly impure 25. Analytically pure sample 25 was obtained by subsequent HPLC separation on C18-reversed phase (60–90% MeOH in H_2_O).

Method B: Dowex 50 W X8 (*ca.* 100 mg, H^+^ form) was added to a solution of epoxide 22a (95.6 mg) in dry Et_2_O (2.0 mL) at RT under Ar atmosphere. The reaction mixture was stirred at RT until full conversion (2 days, TLC). Then, reaction mixture was filtered through a cotton plug and concentrated under reduced pressure. Chromatography of the residue on a short column of silica gel (30–60% EtOAc in hexanes) and subsequent HPLC separation on C18-reversed phase (60–90% MeOH in H_2_O) furnished corresponding product.

Method C: KCN (6.5 mg, 0.10 mmol) was added to a solution of acetyl derivatives 27 and 28 mixture (47.7 mg, 0.10 mmol, 1.6/1, 27/28) in dry MeOH (1.7 mL) at RT under Ar atmosphere. The reaction mixture was stirred at RT until full conversion (2 days, TLC). Then, reaction mixture was concentrated under reduced pressure, redissolved in CHCl_3_, filtered through a cotton plug and concentrated under reduced pressure. Column chromatography of the residue on silica gel (30–60% EtOAc in hexanes) furnished mixture of allylic alcohols 24 and 25 (36.4 mg, 70%) in the ratio of 1.6/1 (24/25), off-white wax.

##### Methyl (1*S*,3*aS*,4*R*,5*S*,7*S*,7*aR*)-1-benzyl-5-hydroxy-7-methyl-6-methylene-3-oxo-4-(2-phenethoxyethyl)octahydro-3*aH*-isoindole-3*a*-carboxylate (24)

HPLC furnished compound 24 (13.4 mg, 14%, Method A); off-white wax; [*α*]_D_ = −9.2° (c 0.31; CHCl_3_). ^1^H NMR (400 MHz, CDCl_3_) *δ* 7.35–7.16 (m, 8H, Ph, Bn), 7.14 (m, 2H, 2× *o*-Bn), 5.69 (s, 1H, NH), 5.27 (m, 1H, H-12b), 5.04 (m, 1H, H-12a), 4.76 (bs, 1H, OH), 3.93 (d, *J*_7,8_ = 9.5 Hz, 1H, H-7), 3.81 (s, 3H, COOCH_3_), 3.68 (t, *J*_15,16_ = 7.4 Hz, 2H, H-15a,b), 3.69–3.62 (m, 1H, H-14b), 3.49 (m, 1H, H-14a), 3.34 (m, 1H, H-3), 2.90 (t, *J*_16,15_ = 7.4 Hz, 2H *overlapped*, H-16a,b), 2.90 (m, 1H *overlapped*, H-5), 2.82 (dd, *J*_gem_ = 13.2 Hz, *J*_10b,3_ = 5.0 Hz, 1H, H-10b), 2.69 (dd, *J*_gem_ = 13.2 Hz, *J*_10a,3_ = 9.2 Hz, 1H, H-10a), 2.55–2.50 (m, 2H, H-8, H-4), 2.15–2.08 (m, 1H, H-13b), 2.05–1.94 (m, 1H, H-13a), 1.00 (d, *J*_11,5_ = 6.7 Hz, 3H, CH_3_-11) ppm. ^13^C NMR (101 MHz, CDCl_3_) *δ* 173.28 (*C*OOCH_3_), 172.81 (C-1), 148.44 (C-6), 138.26 (C–*i*-Ph), 137.20 (C–*i*-Bn), 129.14 (2× CH–*o*-Bn), 128.89 (2× CH–Ar), 128.86 (2× CH–Ar), 128.37 (2× CH–Ar), 127.06 (CH–*p*-Bn), 126.26 (CH–*p*-Ph), 113.33 (CH_2_-12), 72.86 (CH-7), 72.11 (CH_2_-15), 71.31 (CH_2_-14), 57.48 (C-9), 53.81 (CH-3), 52.92 (COO*C*H_3_), 50.22 (CH-4), 44.07 (CH_2_-10), 42.85 (CH-8), 35.94 (CH_2_-16), 31.22 (CH-5), 31.00 (CH_2_-13), 13.70 (CH_3_-11) ppm. HRMS (ESI) *m*/*z* calcd for C_29_H_35_O_5_NNa^+^ [M + Na]^+^ 500.2407, found 500.2408.

##### Methyl (1*S*,3*aS*,4*R*,5*S*,7*aR*)-1-benzyl-5-hydroxy-6,7-dimethyl-3-oxo-4-(2-phenethoxyethyl)-1,2,3,4,5,7*a*-hexahydro-3*aH*-isoindole-3*a*-carboxylate (25)

HPLC furnished compound 25 (9.6 mg, 10% (slightly impure), Method A; 12,4 mg, 13%, Method B), off-white wax; [*α*]_D_ = −15.8° (c 0.27; CHCl_3_). ^1^H NMR (400 MHz, CDCl_3_) *δ* 7.33 (m, 2H, 2× *m*-Bn), 7.29–7.24 (m, 3H, 2× *m*-Ph, *p*-Bn), 7.21–7.18 (m, 3H, 2× *o*-Ph, *p*-Ph), 7.15 (m, 2H, 2× *o*-Bn), 5.65 (bs, 1H, NH), 3.89 (m, 1H, H-7), 3.83 (s, 3H, COOCH_3_), 3.65–3.61 (m, 2H, H-15a,b), 3.57 (dt, *J*_1_ = 9.7 Hz, *J*_2_ = 5.0 Hz, 1H, H-14b), 3.43 (m, 1H, H-3), 3.40 (m, 1H, OH), 3.39–3.34 (m, 1H, H-14a), 3.16 (m, 1H, H-4), 3.01 (dd, *J*_gem_ = 13.4 Hz, *J*_10b,3_ = 5.1 Hz, 1H, H-10b), 2.87 (t, *J*_16,15_ = 7.3 Hz, 2H, H-16a,b), 2.70 (dd, *J*_gem_ = 13.4 Hz, *J*_10a,3_ = 9.3 Hz, 1H, H-10a), 2.41–2.36 (m, 1H, H-8), 1.92–1.79 (m, 2H, H-13a,b), 1.75 (m, 3H, CH_3_-12), 1.64 (m, 3H, CH_3_-11) ppm. ^13^C NMR (101 MHz, CDCl_3_) *δ* 173.46 (*C*OOCH_3_), 172.76 (C-1), 138.55 (C–*i*-Ph), 137.26 (C–*i*-Bn), 131.48 (C-6), 129.02 (2× CH–*o*-Bn), 128.94 (2× CH–*m*-Bn), 128.87 (2× CH–*o*-Ph), 128.33 (2× CH–*m*-Ph), 127.10 (CH–*p*-Bn), 126.20 (CH–*p*-Ph), 125.05 (C-5), 71.95 (CH_2_-15), 70.80 (CH-7), 70.73 (CH_2_-14), 58.65 (CH-3), 57.00 (C-9), 53.01 (COO*C*H_3_), 49.26 (CH-4), 43.25 (CH_2_-10), 42.99 (CH-8), 36.06 (CH_2_-16), 29.59 (CH_2_-13), 17.59 (CH_3_-11), 16.08 (CH_3_-12) ppm. HRMS (ESI) *m*/*z* calcd for C_29_H_35_O_5_NNa^+^ [M + Na]^+^ 500.2407, found 500.2409.

##### Methyl (1*S*,3*aS*,4*R*,5*R*,6*R*,7*S*,7*aR*)-1-benzyl-6-chloro-5-hydroxy-6,7-dimethyl-3-oxo-4-(2-phenethoxyethyl)octahydro-3*aH*-isoindole-3*a*-carboxylate (26)

HPLC furnished 26 (9.6 mg, 38%, Method A), white amorphous solid; [*α*]_D_ = −52.3° (c 0.26; CHCl_3_). ^1^H NMR (400 MHz, CDCl_3_) *δ* 7.41–7.34 (m, 2H, 2× *m*-Bn), 7.34–7.24 (m, 1H, *p*-Bn) 7.15–7.10 (m, 2H, 2× *o*-Bn) 7.06–6.99 (m, 5H, Ph), 4.76 (br s, 1H, NH), 3.95 (dt, *J*_gem_ = 8.8 Hz, *J*_15a,16b_ = *J*_15a,16b_ = 6.0 Hz, 1H, CH-15a), 3.80 (dt, *J*_gem_ = 8.4 Hz, *J*_15b,16a_ = *J*_15b,16a_ = 6.1 Hz, 1H, CH-15b), 3.71 (s, 3H, COOCH_3_), 3.68–3.56 (m, 2H, CH_2_-14), 3.58–3.46 (m, 1H, CH-3), 3.30 (d, *J*_7,8_ = 2.0 Hz, 1H, CH-7), 2.98 (*J*_gem_ = 13.6 Hz, 1H, H-10a), 2.89–2.80 (m, 1H, CH-8), 2.75 (t, *J*_16,15_ = 6.3 Hz, 2H, H-16), 2.47–2.35 (m, 2H, CH-4, CH-10b), 2.32–2.11 (m, 2H, CH_2_-13), 2.00 (qd, *J*_5,11_ = 7.6 Hz, *J*_5,4_ = 5.6 Hz, CH-5), 1.21 (s, 3H, CH_3_-12), 1.14 (d, *J*_11,5_ = 7.6 Hz, 1H, CH_3_-11) ppm. ^13^C NMR (101 MHz, CDCl_3_) *δ* 173.57 (C-1), 173.18 (*C*OOCH_3_), 138.85 (C–*i*-Ph), 137.69 (C–*i*-Bn), 129.18 (2× CH–*o*-Bn), 128.87 (2× CH–*m*-Bn), 128.49 (2× CH–*o*-Ph), 128.08 (2× CH–*m*-Ph), 126.98 (CH–*p*-Bn), 126.08 (CH–*p*-Ph), 86.13 (CH-7), *75.40* (CH_2_-15), 74.49 (C-6), *56.77* (C-9), 54.86 (CH-3), 53.01 (COO*C*H_3_), 49.81 (CH-4), 44.65 (CH_2_-10), 44.07 (CH_2_-14), 38.43 (CH-5), 37.03 (CH-8), 36.49 (CH_2_-16), 30.50 (CH_2_-13), *22.53* (CH_3_-12), 13.87 (CH_3_-11) ppm. *Signals in italics were observed indirectly through HSQC and HMBC spectra*. HRMS (ESI) *m*/*z* calcd for C_29_H_36_O_5_NClNa^+^ [M + Na]^+^ = 536.2174, found = 536.2170.

#### Acetylation and elimination of diol 23a

Ac_2_O (0.15 mL, 1.6 mmol) was added dropwise to a solution of 23a (49.6 mg, 0.10 mmol) in dry pyridine (2.5 mL). The reaction mixture was stirred at RT until full conversion (1 day, TLC). Then, reaction mixture was concentrated under reduced pressure, dissolved in EtOAc (10 mL), washed with saturated aqueous CuSO_4_ (2 × 5 ml), H_2_O (5 mL), brine (5 mL), dried (MgSO_4_) and concentrated under reduced pressure. Crude product was dissolved in dry pyridine (4.0 mL), then SOCl_2_ (80 μL, 1.10 mmol) was added dropwise at 0 °C under Ar atmosphere, followed by addition of DMAP (8.6 mg, 0.07 mmol). The reaction mixture was stirred at indicated temperature until full conversion (TLC). Then, reaction mixture was concentrated under reduced pressure, dissolved in Et_2_O/EtOAc (1/1, 15 mL), washed with saturated aqueous CuSO_4_ (2 × 5 ml), H_2_O (5 mL), brine (5 mL), dried (MgSO_4_) and concentrated under reduced pressure. Column chromatography of the residue on silica gel (75% EtOAc in hexanes) furnished corresponding products 27 and 28 as a mixture that were separated by a subsequent HPLC separation.

##### Methyl (1*S*,3*aS*,4*R*,5*S*,7*S*,7*aR*)-5-acetoxy-1-benzyl-7-methyl-6-methylene-3-oxo-4-(2-phenethoxyethyl)octahydro-3*aH*-isoindole-3*a*-carboxylate (27)

HPLC furnished 27 (18.2 mg, 35%), white wax; [*α*]_D_ = +23,8° (c 0.34; CHCl_3_). ^1^H NMR (400 MHz, CDCl_3_) *δ* 7.32 (m, 2H, 2× *m*-Bn), 7.28–7.16 (m, 6H, 2× *o*-Ph, 2× *m*-Ph, *p*-Ph, *p*-Bn), 7.14 (m, 2H, 2× *o*-Bn), 5.61 (s, 1H, NH), 5.40 (d, *J*_7,8_ = 5.3 Hz, 1H, H-7), 5.23 (m, 1H, H-12b), 5.09 (m, 1H, H-12a), 3.83 (s, 3H, COOCH_3_), 3.62 (dt, *J*_gem_ = 9.3 Hz, *J*_15b,16a_ = *J*_15b,16b_ = 7.2 Hz, 1H, H-15b), 3.57 (dt, *J*_gem_ = 9.3 Hz, *J*_15a,16a_ = *J*_15a,16b_ = 7.2 Hz, 1H, H-15a), 3.55–3.45 (m, 2H, H-14a,b), 3.39 (ddd, *J*_3,10a_ = 9.8 Hz, *J*_3,4_ = 7.3, *J*_3,10b_ = 3.6 Hz, 1H, H-3), 3.17 (dd, *J*_gem_ = 13.5 Hz, *J*_10b,3_ = 3.6 Hz, 1H, H-10b), 3.08 (t, *J*_4,3_ = *J*_4,5_ = 7.3 Hz, 1H, H-4), 3.06–2.96 (m, 2H *overlapped*, H-8, H-5), 2.88–2.84 (m, 2H, H-16b,a), 2.52 (dd, *J*_gem_ = 13.5 Hz, *J*_10b,3_ = 9.8 Hz, 1H, H-10a), 1.99 (s, 3H, OCOCH_3_), 1.85–1.77 (m, 1H, H-13b), 1.57 (dddd, *J*_gem_ = 14.0 Hz, *J*_13a,8_ = 10.3 Hz, *J*_13a,14b_ = 7.6 Hz, *J*_13a,14a_ = 6.4 Hz, 1H, H-13a), 1.21 (d, *J*_11,5_ = 6.9 Hz, 3H, CH_3_-11) ppm. ^13^C NMR (101 MHz, CDCl_3_) *δ* 171.74 (C-1), 171.58 (*C*OOCH_3_), 169.57 (O*C*OCH_3_), 143.49 (C-6), 138.82 (C–*i*-Ph), 137.27 (C–*i*-Bn), 129.04 (2× CH–*o*-Bn), 128.89 (2× CH–*m*-Bn), 128.83 (2× CH–*o*-Ph), 128.25 (2× CH–*m*-Ph), 127.18 (CH–*p*-Bn), 126.06 (CH–*p*-Ph), 114.89 (CH_2_-12), 75.96 (CH-7), 71.79 (CH_2_-15), 69.81 (CH_2_-14), 57.89 (C-9), 54.08 (CH-3), 53.08 (COO*C*H_3_), 49.21 (CH-4), 43.96 (CH_2_-10), 39.46 (CH-8), 36.17 (CH_2_-16), 29.91 (CH-5), 28.67 (CH_2_-13), 21.18 (OCO*C*H_3_), 15.42 (CH_3_-11) ppm. HRMS (ESI) *m*/*z* calcd for C_31_H_37_O_6_NNa^+^ [M + Na]^+^ 542.2513, found 542.2508.

##### Methyl (1*S*,3*aS*,4*R*,5*S*,7*aR*)-5-acetoxy-1-benzyl-6,7-dimethyl-3-oxo-4-(2-phenethoxyethyl)-1,2,3,4,5,7*a*-hexahydro-3*aH*-isoindole-3a-carboxylate (28)

HPLC furnished compound 28 (2.6 mg, 5%), white wax; [*α*]_D_ = −51.0° (c 0.20; CHCl_3_). ^1^H NMR (400 MHz, CDCl_3_) *δ* 7.34 (m, 2H, 2× *m*-Bn), 7.29–7.24 (m, 3H, 2× *m*-Ph, *p*-Bn), 7.19–7.16 (m, 5H, 2× *o*-Ph, *p*-Ph, 2× *o*-Bn), 5.55 (s, 1H, NH), 5.23 (d, *J*_7,8_ = 2.8 Hz, 1H, H-7), 3.80 (s, 3H, COOCH_3_), 3.64 (dt, *J*_1_ = 9.3 Hz, *J*_2_ = 7.4 Hz, 1H, H-15b), 3.67–3.47 (m, 5H, H-15a, H-14a,b, H-4, H-3), 3.43 (dd, *J*_gem_ = 13.4 Hz, *J*_10b,3_ = 3.0 Hz, 1H, H-10b), 2.94 (dt, *J*_8,13b_ = *J*_8,13a_ = 10.7 Hz, *J*_8,7_ = 2.8 Hz, 1H, H-8), 2.87–2.83 (m, 2H, H-16a,b), 2.66 (dd, *J*_gem_ = 13.4 Hz, *J*_10b,3_ = 10.2 Hz, 1H, H-10a), 1.99 (s, 3H, OCOCH_3_), 1.92 (m, 3H, CH_3_-11), 1.71 (m, 3H, CH_3_-12), 1.70–1.65 (m, 1H, H-13b), 1.35 (ddt, *J*_gem_ = 13.8 Hz, *J*_13a,8_ = 10.7 Hz, *J*_13a,14a_ = *J*_13a,14b_ = 7.2 Hz, 2H, H-13a) ppm. ^13^C NMR (101 MHz, CDCl_3_) *δ* 171.65 (C-1), 171.02 (*C*OOCH_3_), 170.47 (O*C*OCH_3_), 138.74 (C–*i*-Ph), 137.27 (C–*i*-Bn), 129.61 (C-6), 129.13 (2× CH–*o*-Bn), 128.87 (2× CH–Ar), 128.83 (2× CH–*o*-Ph), 128.29 (2× CH–Ar), 127.31 (CH–*p*-Bn), 126.10 (CH–*p*-Ph), 123.98 (C-5), 72.73 (CH-7), 71.90 (CH_2_-15), 69.67 (CH_2_-14), 58.07 (CH-3), 57.09 (C-9), 53.23 (COO*C*H_3_), 46.48 (CH-4), 44.62 (CH_2_-10), 37.88 (CH-8), 36.16 (CH_2_-16), 28.70 (CH_2_-13), 21.01 (OCO*C*H_3_), 18.35 (CH_3_-11), 17.82 (CH_3_-12) ppm. HRMS (ESI) *m*/*z* calcd for C_31_H_37_O_6_NNa^+^ [M + Na]^+^ 542.2513, found 542.2505.

##### (1*S*,3*aR*,4*S*,7*S*,7*aR*)-1-Benzyl-6,7-dimethyl-3-oxo-4-(2-phenethoxyethyl)-1,2,3,4,7,7*a*-hexahydro-3*aH*-isoindole-3*a*-carboxylic acid (29)

NaOH (80 mg, 2.00 mmol) in MeOH/H_2_O (4/1, 0.5 mL) was added to a solution of 16a (56.6 mg, 0.10 mmol) in MeOH (0.5 mL). The reaction mixture was stirred at 60 °C until full conversion (2 days, TLC). Then, reaction mixture was cooled to RT, acidified by 1 M HCl (5 mL) and extracted with EtOAc (2 × 5 ml). Combined organic phases were washed with brine (3 mL), dried (MgSO_4_) and concentrated under reduced pressure. HPLC separation on C18-reversed phase (50–95% MeOH in H_2_O) furnished 29 (35.8 mg, 80%), white amorphous solid; [*α*]_D_ = −31.9° (c 0.28; CHCl_3_). ^1^H NMR (400 MHz, CDCl_3_) *δ* 7.34–7.17 (m, 8H, Ph, 3× Bn), 7.14 (m, 2H, 2× *o*-Bn), 6.77 (bs, 1H, NH), 5.48 (m, 1H, H-7), 3.65 (dt, *J*_gem_ = 9.4 Hz, *J*_15b,16a_ = *J*_15b,16b_ = 7.1 Hz, 1H, H-15b), 3.57 (dt, *J*_gem_ = 9.4 Hz, *J*_15a,16a_ = *J*_15a,16b_ = 7.1 Hz, 1H, H-15a), 3.56–3.43 (m, 2H, H-14a,b), 3.25 (dt, *J*_3,10a_ = 8.9 Hz, *J*_3,4_ = *J*_3,10b_ = 4.2 Hz, 1H, H-3), 2.92 (dd, *J*_gem_ = 13.6 Hz, *J*_10b,3_ = 3.8 Hz, 1H, H-10b), 2.89–2.81 (m, 2H, H-16a,b), 2.78 (t, *J*_4,3_ = *J*_4,5_ = 4.5 Hz, 1H, H-4), 2.66 (dd, *J*_gem_ = 13.6 Hz, *J*_10a,3_ = 9.6 Hz, 1H, H-10a), 2.54 (m, 1H, H-5), 2.51 (m, 1H, H-8), 2.18–2.06 (m, 1H, H-13b), 1.90–1.79 (m, 1H, H-13a), 1.78 (m, 3H, CH_3_-12), 1.19 (d, *J*_11,5_ = 7.3 Hz, 3H, CH_3_-11) ppm. (*H from COOH is missing*) ^13^C NMR (101 MHz, CDCl_3_) *δ* 175.97 (C=O), 174.94 (C=O), 140.07 (C-6), 139.09 (C–*i*-Ph), 137.09 (C–*i*-Bn), 129.08 (2× CH–*o*-Bn), 128.92 (4× CH–Ar), 128.23 (2× CH–Ar), 127.04 (CH–*p*-Bn), 126.02 (CH-7), 125.93 (CH–*p*-Ph), 71.57 (CH_2_-15), 69.71 (CH_2_-14), 60.15 (C-9), 56.75 (CH-3), 52.87 (CH-4), 44.70 (CH_2_-10), 37.86 (CH-8), 36.27 (CH_2_-16), 34.27 (CH-5), 29.78 (CH_2_-13), 20.46 (CH_3_-12), 14.22 (CH_3_-11) ppm. HRMS (ESI) *m*/*z* calcd for C_28_H_33_O_4_NNa^+^ [M + Na]^+^ 470.2302, found 470.2302.

##### (3*S*,3*aR*,4*S*,7*S*,7*aS*)-3-Benzyl-7*a*-(hydroxymethyl)-4,5-dimethyl-7-(2-phenethoxyethyl)-2,3,3*a*,4,7,7*a*-hexahydro-1*H*-isoindol-1-one (30)

LiBH_4_ (10.9 mg, 0.50 mmol) was added to a solution of 17a (46.2 mg, 0.10 mmol) in dry THF (0.5 mL) at RT under argon atmosphere. The reaction mixture was stirred at RT until full conversion (2 days, TLC). Then, reaction mixture was quenched with saturated aqueous NH_4_Cl (5 mL) and extracted with EtOAc (3 × 5 ml). Combined organic phases were washed with brine (3 mL), dried (MgSO_4_) and concentrated under reduced pressure. Column chromatography of the residue on silica gel (20–80% EtOAc in hexanes) furnished compound 30 (25.2 mg, 58%), white wax; [*α*]_D_ = +29.0° (c 0.27; CHCl_3_). ^1^H NMR (400 MHz, CDCl_3_) 7.33–7.17 (m, 8H, Ph, 3× Bn), 7.15 (m, 2H, 2× *o*-Bn), 5.48 (bs, 1H, NH), 5.44 (m, 1H, H-7), 3.80 (d, *J*_gem_ = 11.1 Hz, 1H, CHa*Hb*OH), 3.65 (dt, *J*_gem_ = 9.4 Hz, *J*_15b,16a_ = *J*_15b,16b_ = 7.1 Hz, 1H, H-15b), 3.58 (dt, *J*_gem_ = 9.4 Hz, *J*_15a,16a_ = *J*_15a,16b_ = 7.1 Hz, 1H, H-15a), 3.58–3.53 (m, 1H, H-14b), 3.52 (d, *J*_gem_ = 11.1 Hz, 1H, C*Ha*HbOH), 3.43 (td, *J* = 9.5, 4.8 Hz, 1H, H-14a), 3.22 (ddd, *J*_3,10a_ = 10.0 Hz, *J*_3,4_ = 5.6 Hz, *J*_3,10b_ = 3.3 Hz, 1H, H-3), 3.01 (dd, *J*_gem_ = 13.6 Hz, *J*_10b,3_ = 3.3 Hz, 1H, H-10b), 2.88–2.82 (m, 2H, H-16a,b), 2.55 (dd, *J*_gem_ = 13.2 Hz, *J*_10,3_ = 10.0 Hz, 1H, H-10a), 2.55 (dd, *J*_4,3_ = 5.6 Hz, *J*_4,5_ = 4.4 Hz, 1H, H-4), 2.40 (m, 1H, H-5), 2.30–2.20 (m, 1H, H-13b), 2.00 (m, 1H, H-8), 1.77 (m, 3H, CH_3_-12), 1.59–1.49 (m, 1H, H-13a), 1.24 (d, *J*_11,5_ = 7.3 Hz, 3H, CH_3_-11) ppm (*H from OH is missing*). ^13^C NMR (101 MHz, CDCl_3_) *δ* 177.82 (C-1), 139.10 (C–*i*-Ph), 139.05 (C-6), 137.98 (C–*i*-Bn), 128.95 (2× CH–*o*-Bn), 128.87 (4× CH–Ar), 128.25 (2× CH–Ar), 126.98 (CH-7), 126.88 (CH), 126.07 (CH), 71.60 (CH_2_-15), 69.65 (CH_2_-14), 65.70 (CH_2_OH), 55.81 (CH-3), 55.25 (C-9), 51.10 (CH-4), 45.03 (CH_2_-10), 36.26 (CH_2_-16), 35.00 (CH-8), 33.90 (CH-5), 30.06 (CH_2_-13), 20.55 (CH_3_-12), 14.95 (CH_3_-11) ppm. HRMS (ESI) *m*/*z* calcd for C_28_H_35_O_3_NNa^+^ [M + Na]^+^ 456.2515, found 456.2511.

### Spheroid invasion assay

The spheroid invasion assay was performed following a previously published protocol.^63^ Briefly, BLM cells were grown as spheroids using 3D Petri Dish (Microtissues). After 2 days of spheroid formation, 1.5 mg mL^−1^ collagen solution (rat tail, collagen type I) was prepared (1.5 mg mL^−1^ rat-tail collagen, 1× DMEM, 1% (v/v) FBS) and added at the bottom of a 96-well plate. Spheroids were embedded into the collagen (one spheroid per well) and covered with another layer of the gel. Collagen was polymerized at 37 °C and subsequently overlaid with a cultivation medium. Images of spheroids were taken with Leica TCS SP2 microscope (5×/0.15 dry objective; LAS X Life Science Microscope software) immediately after their seeding into collagen and again after 24 h. The area of the spheroids at 0 and 24 h was measured using FiJi (Rasband, W.S., ImageJ, NIH, Bethesda, MD, USA). Cellular invasiveness was determined as an increase in spheroid area within 24 h. Three independent experiments were conducted with at least 8 spheroids analysed in each experiment. Statistical analysis was performed in GraphPad using one-way ANOVA. For numeric data and representative images from this assay, see ESI,† Table S1 and Fig. S1.

### Actin polymerization assay

Actin polymerization assay was performed using Actin Polymerization Biochem Kit™ (Cytoskeleton, Inc, Denver, CO) according to the manufacturer protocol. Briefly, 25 μL of G-buffer containing labelled pyrene-actin (0.4 mg mL^−1^) and 2.48 μL of either DMSO (10 μM, control) or inhibitor (10 μM) was added in 386 well plate. Actin polymerization was initiated by the addition of 2.48 μL of 10× actin polymerization buffer and analysed in time until fluorescent signal plateau was reached using a spectrophotometer (Infinite 200 PRO, Tecan Life Sciences, Tecan Group Ltd., Zürich, CHE). The fluorescence emission intensity of actin polymerization was measured at 420 nm and 360 nm was used as excitation wavelength. Statistical analysis was performed in GraphPad using one-way ANOVA. Measurements were performed in three independent repetitions each consisting of at least three technical replicates. For a representative graph from this assay, see ESI,† Fig. S2.

### Cell line cultivation

Human cells from melanoma (BLM) and human lung fibroblasts (MRC-5, Merck, USA) were used to evaluate *in vitro* cytotoxicity of the prepared compounds. The cells were maintained at the exponential phase of growth (passaging done by trypsin–EDTA solution) in DMEM and MEM, for BLM and MRC-5, respectively, with stable l-glutamine and supplemented with 10% fetal bovine serum (Thermo Fisher Scientific, USA). The cells were kept in an incubator at 37 °C, 5% CO_2_ in the atmosphere, and 95% humidity.

### Cytotoxicity evaluation

The compound cytotoxicity was determined by a WST-1 viability assay (Merck, USA). The amount of 5000 BLM and MRC-5 cells were seeded into individual wells of 96-well plates in 100 μL of cell cultivation media and maintained in an incubator for 16 h. After this period, the cells were treated with a concentration series (0–50 μM) of the tested compounds diluted in another 100 μL of cultivation media, which was added to the wells containing cells and incubated for 72 h. Then, BLM and MRC-5 cell viability was evaluated by WST-1 agent (5% v/v solution in phenol red-free DMEM media), *i.e.*, the cell media was discarded and 100 μL of WST-1 solution was added. After 2 h incubation, the absorbance of formazan, derived from the WST-1 tetrazolium salt was determined spectrophotometrically at 450 nm (reference at 650 nm) by a UV-vis spectrophotometer (Bio-Rad, USA). Untreated cells, cells treated only with a vehicle, and cells treated with cytochalasin B (1) and D (2) served as controls. The measurement occurred in three independent repetitions each of which consisted of three technical replicates. The data were plotted as dose–response curves from which the half-maximal inhibitory concentrations (IC_50_) were calculated using AAT Bioquest.

### Fluorescence microscopy of actin cytoskeleton

HT1080 cells were seeded into 12-well glass bottom plate and stained with SPY650-FastAct™ following manufacturer's protocol. Untreated cells (control) were imaged every 20 s for 15 min using Leica TCS SP2 microscope (40×/0.6 dry objective). Afterwards, an inhibitor (compound 24 at 10 μM; 50 μM or compound 1 and 2 at 10 μM) was added into the same well and its effect was recorded for 15 min (20 s/image). All video recordings were analyzed using FiJi (Rasband, W.S., ImageJ, NIH, Bethesda, MD, USA).

## Conclusions

In conclusion, we synthesized cytochalasin analogues devoid of the macrocyclic moiety and evaluated their migrastatic activity and cytotoxicity against both cancer and noncancerous cell lines. Among these analogues, compounds 20, 24, and 30 demonstrated migrastatic activity, which correlated with their ability to inhibit actin polymerization *in vitro*. Most analogues lacking oxygen substituents on the cytochalasin core exhibited moderate cytotoxicity without significant selectivity between the screened cell lines. Within the group of oxygenated analogues, only compound 24, sharing the same substitution pattern as cytochalasins B and D (1, 2), displayed the migrastatic effect, while being non-cytotoxic at 50μM concentration.

Our findings emphasize that designing biologically active compounds based on cytochalasin core is possible, although the macrocycle certainly improves the activity. The substitution pattern of the core emerged as a critical factor in modulating biological activities, shifting from cytotoxicity to migrastatic activity. Importantly, our results demonstrate that cytotoxic and migrastatic activities among cytochalasins may be at least partially independent of each other. These results lay the foundation for further advancements in the development of cytochalasin analogues as potential migrastatics.

## Author contributions

Synthesis and spectroscopic measurements: B. F., D. D., P. P.; biological activity screening: T. V., A. Š., S. R.; project conception and supervision: P. P., D. R., J. B.; HPLC purity measurements: J. H.; manuscript writing: B. F., D. D., P. P., T. V., S. R.

## Conflicts of interest

There are no conflicts to declare.

## Supplementary Material

MD-015-D3MD00535F-s001

MD-015-D3MD00535F-s002
