# Supplementary material for: Synthesis and migrastatic activity of cytochalasin analogues lacking a macrocyclic moiety
Source: RSC Med Chem. 2023 Nov 28;15(1):322–43. doi: 10.1039/d3md00535f (PMC10809383; doi:10.1039/d3md00535f)
Supplement: MD-015-D3MD00535F-s001 [file MD-015-D3MD00535F-s001.pdf]

Electronic Supplementary Material (ESI) for RSC Medicinal Chemistry.
This journal is © The Royal Society of Chemistry 2023

Supporting Information

Synthesis and migrastatic activity of cytochalasin analogues lacking a macrocyclic moiety

Bedřich Formánek,[a] Dorian Dupommier,[a] Tereza Volfová,[b] Silvie Rimpelová,[c] Aneta Škarková,[b] Jana Herciková,[a] Daniel Rösel,[b] Jan Brábek,[b] and Pavla Perlíková *[a,d]

a) *Department of Organic Chemistry, Faculty of Chemical Technology, University of Chemistry and Technology, Prague, Technická 5, 166 28 Prague, Czech Republic. E-mail: perlikop@vscht.cz*
b) *Department of Cell Biology, BIOCEV, Faculty of Science, Charles University, Vestec, Prague West 252 50, Czech Republic.*
c) *Department of Biochemistry and Microbiology, Faculty of Food and Biochemical Technology, University of Chemistry and Technology Prague, Technická 5, 166 28 Prague, The Czech Republic.*
d) *Institute of Organic Chemistry and Biochemistry, Czech Academy of Sciences, Flemingovo namesti 2, 160 00 Prague, Czech Republic.*

Contents:

Synthesis of compound **5** S2
Supplementary schemes S2-S4 .... S5
Supplementary table S1 .... S7
Supplementary figures S1-S2 .... S7
HPLC purity of final cytochalasin analogues S9
Copies of NMR spectra S22

Synthesis of compound 5

General remarks

Visualization methods for TLC**:** Compounds were visualized by irradiation with UV light (254 nm) and/or by treatment with a solution of phosphomolybdic acid (5 g), $Ce(SO_4)_2 \cdot H_2O$ (2 g), conc. H_2SO_4 (12 mL) and H_2O (188 mL) followed by heating or treatment with a solution of anisaldehyde (9 mL) in EtOH (230 mL), conc. H_2SO_4 (8 mL) and conc. AcOH (3 mL) followed by heating or treatment with a solution of ninhydrin (0.2 g) in EtOH (100 mL), conc. AcOH (0.5 mL) and H_2O (4.5 mL) followed by heating.

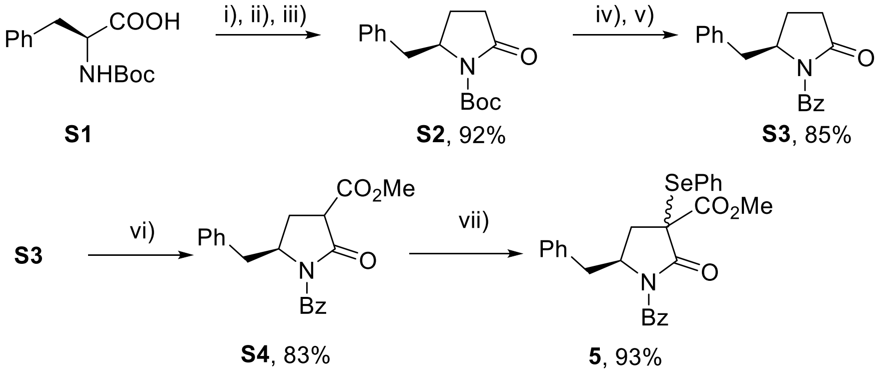

Scheme S1: Preparation of selenide **5**. Reaction conditions: i) Meldrum's acid, EDC, DMAP, DCM, 0°C to RT, 3 h; ii) $NaBH_4$, AcOH, DCM, 0°C to RT; iii) toluene, reflux, 3 h; iv) TFA, DCM, 10°C-RT, 1 h; v) BzCl, py, RT, 4 h; vi) $ClCO_2Me$, LiHMDS, THF, -78°C, 4.5 h; vii) PhSeCl, LiHMDS, THF, -78°C, 5 h.

The *tert*-butyl (*R*)-2-benzyl-5-oxopyrrolidine-1-carboxylate (**S2**) was synthesized according to the known procedures[1] in three steps starting from (tert-butoxycarbonyl)-L-phenylalanine (**S1**, 7.96 g, 30.0 mmol). All three reactions were monitored by TLC (hexanes/EtOAc/AcOH : 7/3/0.5).[1c]

First step was amide coupling of **S1** and Meldrum's acid (4.54 g, 31.5 mmol, recrystallized) proceeded with EDC (8.63 g, 45.0 mmol) and DMAP (5.50 g, 45.0 mmol) in dry DCM (90 mL) at 0°C, then RT for 3 h[1b,c], followed by evaporation of a crude extract to ca. 100 mL and reduction by $NaBH_4$ (3.40 g, 90.0 mmol) and AcOH (17.2 mL, 300 mmol) at 0°C for 1 h, then RT overnight (after 3 h, the reaction is almost complete).[1a,c] Crude product was refluxed in toluene (150 mL) for 3 h. After evaporation of the solvent, column chromatography of the residue (80 g SiO_2, 0-20% EtOAc in hexanes) furnished the **S2** (7.62 g, 92%), yellow oil. Our physical and spectroscopic data corroborated previously published materials.[1a]

The (*R*)-1-benzoyl-5-benzylpyrrolidin-2-one (**S3**) was synthesized according to the known procedures[1d], TFA (7.6 mL) was added to **S2** (7.62 g, 27.6 mmol) in DCM (30.5 mL) at 10°C dropwise. The reaction mixture was stirred at RT until full conversion (1 h, TLC, hexanes/EtOAc : 4/1). BzCl (6.35 mL, 55.2 mmol) was added dropwise to the crude

[1] a) M. Smrčina, P. Majer, E. Majerová, T. A. Guerassina and M. A. Eissenstat, *Tetrahedron*, 1997, **53**, 12867-12874; b) S. Shankar, N. A. Wani, U. P. Singh and R. Rai, *Chemistry Select*, 2016, **1**, 3675-3678; c) O. Chaloin, F. Cabart, J. Marin, H. Zhang and G. Guichard, *Organic Syntheses,* 2008, **85**, 147-157; d) B. Hao, M. J. Gunaratna, M. Zhang, S. Weerasekara, S. N. Seiwald, V. T. Nguyen, A. Meier and D. H. Hua, *Journal of the American Chemical Society*, 2016, **138**, 16839-16848.

product in pyridine (69 mL) at RT.[2] After full conversion (4 h, TLC, DCM/MeOH : 20/1), Et_2O (173 mL, 2.5 vol of py) was added, precipitate was filtered, filtrate concentrated and co-distilled with toluene (2×) under reduced pressure. Column chromatography of the residue (80 g SiO_2, 10-25% EtOAc in hexanes) furnished the **S3** (6.55 g, 85%), yellow oil. Our physical and spectroscopic data corroborated previously published materials.[3]

Methyl (5*R*)-1-benzoyl-5-benzyl-2-oxopyrrolidine-3-carboxylate (S4)

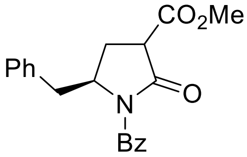

This compound was synthesized in analogy with a known procedure[4] from **S3** (0.57 g, 2.0 mmol) in dry THF (3 mL), using LiHMDS (4.0 mL, 4.0 mmol, 1M in THF) and neat methyl chloroformate (0.31 mL, 4.0 mmol). After full conversion (4 h, TLC, hexanes/EtOAc : 5/1) column chromatography of the residue on silica gel (0-20% EtOAc in hexanes) furnished **S4** (0.57 g, 83%) as a 1.7/1 diastereomeric mixture, yellow oil.

^{1}H NMR (400 MHz, $CDCl_3$)

major: δ 7.63–7.58 (m, 2H, 2×Ar), 7.58–7.50 (m, 1H *overlapped*, Ar), 7.47–7.39 (m, 2H *overlapped*, 2×Ar), 7.38–7.21 (m, 5H *overlapped*, Ar), 4.87 (dddd, $J_{5,4a}$ = 8.6 Hz, $J_{5,Ph\text{-}CHb}$ = 7.9 Hz, $J_{5,Ph\text{-}CHa}$ = 3.5 Hz, $J_{5,4b}$ = 2.7 Hz, 1H, H-5), 3.71 (s, 3H, $COOCH_3$), 3.35 (dd, $J_{3,4a}$ = 10.0 Hz, $J_{3,4b}$ = 9.1 Hz, 1H, H-3), 3.24 (dd, J_{gem} = 13.5 Hz, $J_{Ph\text{-}CHa,5}$ = 3.5 Hz, 1H, Ph-C*H*a), 2.98 (dd, J_{gem} = 13.5 Hz, $J_{Ph\text{-}CHb,5}$ = 7.9 Hz, 1H, Ph-C*H*b), 2.56 (ddd, J_{gem} = 13.2 Hz, $J_{4a,3}$ = 10.0 Hz, $J_{4a,5}$ = 8.6 Hz, 1H, H-4a), 2.34-2.18 (m, 1H *overlapped*, H-4b) ppm.

minor: δ 7.72–7.66 (m, 2H, 2×Ar), 7.58–7.50 (m, 1H *overlapped*, Ar), 7.47–7.39 (m, 2H *overlapped*, 2×Ar), 7.38–7.21 (m, 5H *overlapped*, Ar), 4.62 (dddd, $J_{5,Ph\text{-}CHb,5}$ = 9.8 Hz, $J_{5,4}$ = 7.5 Hz, $J_{5,4}$ = 7.0 Hz, $J_{5,Ph\text{-}CHb,5}$ = 3.6 Hz, 1H, H-5), 3.80 (s, 3H, $COOCH_3$), 3.53 (dd, $J_{3,4a}$ = 9.8 Hz, $J_{3,4b}$ = 8.4 Hz, 1H, H-3), 3.48 (dd, J_{gem} = 13.2 Hz, $J_{Ph\text{-}CHa,5}$ = 3.6 Hz, 1H, Ph-C*H*a), 2.78 (dd, J_{gem} = 12.9 Hz, $J_{Ph\text{-}CHb,5}$ = 9.9 Hz, 1H, Ph-C*H*b), 2.34-2.18 (m, 2H *overlapped*, H-4a,b) ppm.

^{13}C NMR (101 MHz, $CDCl_3$)

major: δ 170.02 (C=O), 169.50 (C=O), 168.97 (C=O), 136.17 (C), 134.13 (C), 132.12 (CH-Ar), 129.67 (2×CH-Ar), 128.80 (2×CH-Ar), 128.79 (2×CH-Ar), 127.88 (2×CH-Ar), 127.19 (CH-Ar), 56.60 (CH-5), 52.83 ($COOCH_3$), 48.84 (CH-3), 38.99 (Ph-*C*H_2), 26.31 (CH_2-4) ppm.

minor: δ 170.93 (C=O), 170.16 (C=O), 170.10 (C=O), 136.39 (C), 144.09 (C), 132.69 (CH-Ar), 129.59 (2×CH-Ar), 129.38 (2×CH-Ar), 128.64 (2×CH-Ar), 128.01 (2×CH-Ar), 126.89 (CH-Ar), 57.24 (CH-5), 52.98 ($COOCH_3$), 49.12 (CH-3), 39.20 (Ph-*C*H_2), 26.38 (CH_2-4) ppm.

HRMS (ESI) *m/z* calcd for $C_{20}H_{19}O_4NNa^+$ $[M+Na]^+$ 360.1206; found 360.1204.

---

[2] E. J. Thomas and M. Willis, *Organic & Biomolecular Chemistry*, 2014, **12**, 7537-7550; this approach furnished easily separable mixture and higher yields compared to BzCl/NaH procedure.
[3] S. A. Harkin, O. Singh and E. J. Thomas, *Journal of the Chemical Society, Perkin Transactions 1*, 1984, 1489-1499.
[4] X. Long, Y. Ding and J. Deng, *Angewandte Chemie, International Edition*, 2018, **57**, 14221-14224.

Methyl (5*S*)-1-benzoyl-5-benzyl-2-oxo-3-(phenylselanyl)pyrrolidine-3-carboxylate (5)

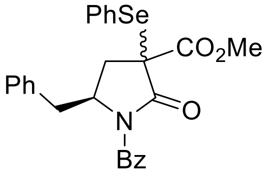

LiHMDS (4.5 mL, 4.5 mmol, 1M in THF) was added dropwise to a stirred solution of **S4** (1.71 g, 5.1 mmol) in dry THF (10 mL) at -78°C under argon atmosphere. After 40 min, a solution of PhSeCl (0.86 g, 4.5 mmol) in dry THF (9 mL) was added dropwise and the reaction mixture was stirred at -78°C until full conversion (3-4 h, TLC, hexanes/EtOAc : 5/1). Then, solution of NH_4Cl (10 mL) was added, the resulting mixture was warmed to RT, poured into a solution of NH_4Cl (40 mL) and extracted with EtOAc (2 × 15 mL). The organics were combined, washed with brine (10 mL), dried (Na_2SO_4) and concentrated under reduced pressure. Column chromatography of the residue on silica gel (12-15% EtOAc in hexanes) furnished the **5** (2.32 g, 93%) as a 1.7/1 diastereomeric mixture, red-brown densy oil.

^{1}H NMR (400 MHz, $CDCl_3$)

major: δ 7.74–7.21 (m, 13H *overlapped*, 13×Ar), 7.18–7.12 (m, 2H, 2×Ar), 4.41–4.30 (m, 1H, H-5), 3.76 (s, 3H, $COOCH_3$), 3.30 (m, 1H *overlapped*, Ph-C*H*a), 2.68–2.54 (m, 2H *overlapped* Ph-C*H*b, H-4a), 2.25–2.17 (m, 1H *overlapped*, H-4b) ppm.

minor: 7.74–7.21 (m, 13H *overlapped*, 13×Ar), 7.12–7.07 (m, 2H, 2×Ar), 4.63 (dtd, J_1 = 9.4 Hz, J_2 = 7.4 Hz, J_3 = 3.4 Hz, 1H, H-5), 3.66 (s, 3H, $COOCH_3$), 3.30 (m, 1H *overlapped*, Ph-C*H*a), 2.73 (dd, J_{gem} = 14.4 Hz, $J_{Ph\text{-}CHb,5}$ = 7.6 Hz, 1H, Ph-C*H*b), 2.68–2.54 (m, 1H *overlapped*, H-4a), 2.25–2.17 (m, 1H *overlapped*, H-4b) ppm.

^{13}C NMR (101 MHz, $CDCl_3$)

major: δ 170.79 (C=O), 169.73 (C=O) 169.61 (C=O), 137.85 (2×CH-Ar), 136.08 (CH-Ar), 134.06 (C *overlapped*), 132.56 (CH-Ar), 130.24 (CH-Ar), 129.56 (2×CH-Ar), 129.39 (2×CH-Ar), 129.19 (2×CH-Ar), 128.62 (2×CH-Ar), 127.94 (2×CH-Ar *overlapped*), 126.92 (CH-Ar), 126.29 (C), 55.71 (CH-5), 53.71 (C-3), 53.53 (COO*C*H_3), 38.46 (Ph-*C*H_2), 34.21 (CH_2-4) ppm.

minor: δ 171.18 (C=O), 169.86 (C=O), 169.02 (C=O), 137.89 (2×CH-Ar), 135.97 (CH-Ar), 134.06 (C *overlapped*), 132.70 (CH-Ar), 130.10 (CH-Ar), 129.60 (2×CH-Ar), 129.42 (2×CH-Ar), 129.25 (2×CH-Ar), 128.67 (2×CH-Ar), 127.94 (2×CH-Ar *overlapped*), 126.96 (CH-Ar), 125.64 (C), 55.92 (CH-5), 54.88 (C-3), 53.59 (COO*C*H_3), 38.76 (Ph-*C*H_2), 34.35 (CH_2-4) ppm.

HRMS (ESI) *m/z* calcd for $C_{26}H_{24}O_4NSe^+$ $[M+H]^+$ 494.0865; found 494.0861.

Scheme S2: Screening of various conditions for epoxide rearrangement.

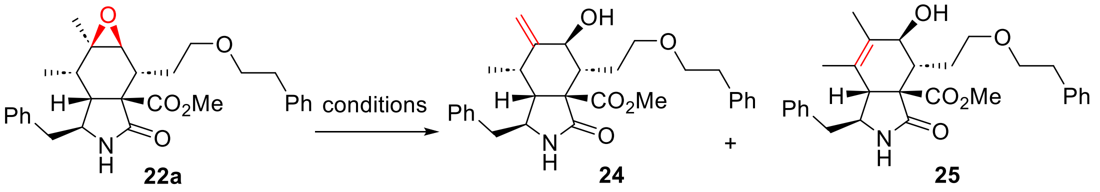

conditions	ratio[a] **24/25**	yield **24/25** (%)
HCl, Et_2O, 0°C[a]	1/1.1 + **26**	14/10 + 38
HBF_4, Et_2O, RT	n.d.	traces
TMSI, DBU, CH_3CN, reflux	n.r.	n.d.
30% H_2SO_4 aq, Et_2O, 0°C	1/1.8	58[b]
DOWEX 50W H^+, Et_2O, RT	1/3.0	53[b,c]

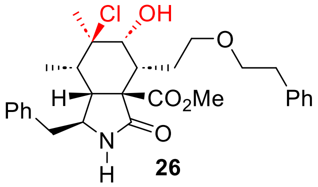

[a] Based on ^{1}H NMR spectra of the crude material. [b] Obtained as a mixture of **24** and **25**. [c] HPLC separation of the mixture furnished compound **25** in 13% yield. n.d. - not determined, n.r. - no reaction

Scheme S3: Proposed mechanism of formation of compounds **24-26** in reaction of **22a** with HCl in diethyl-ether

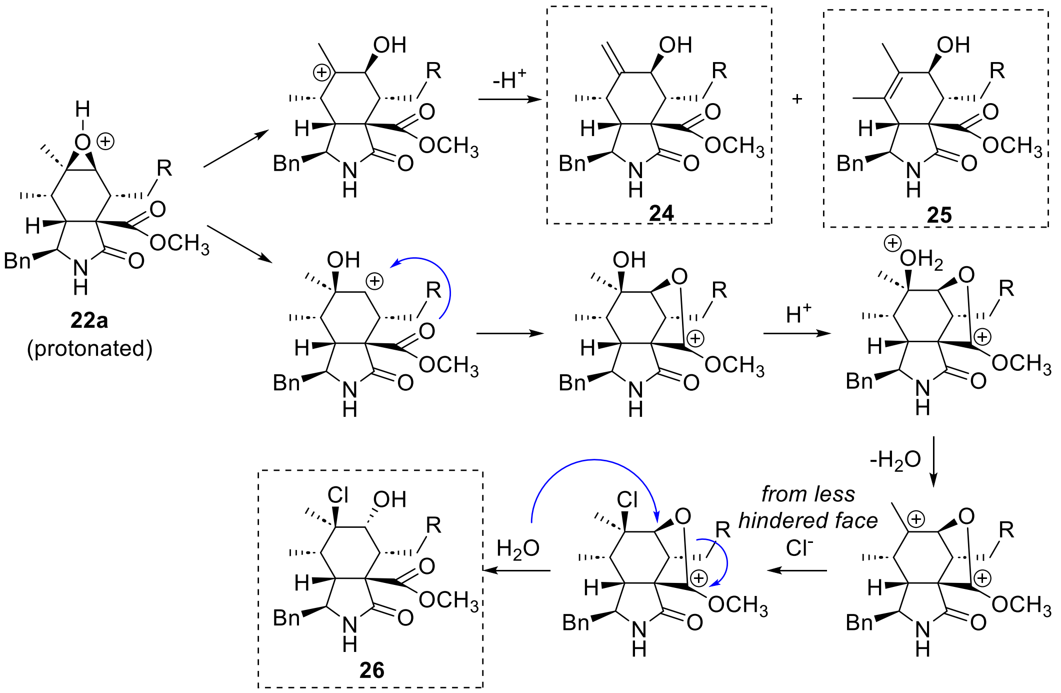

Scheme S4: Screening of various conditions for elimination reaction.

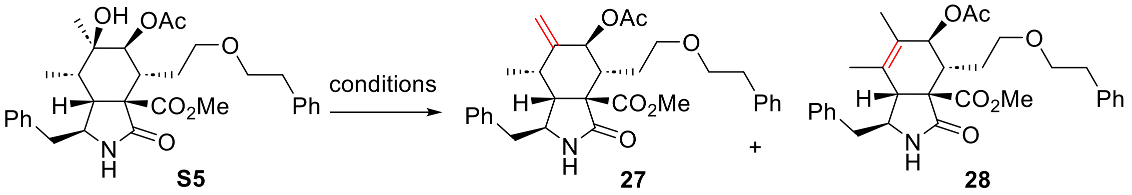

conditions	ratio[a] **27/28**	yield of **27/28**[b] (%)
$SOCl_2$, DMAP, pyridine, 0°C	1.6/1	58
$SOCl_2$, DMAP, pyridine, -40°C	2.0/1	49
$SOCl_2$, DMAP, pyridine, +40°C	1.3/1	50
triphosgene, DMAP, DCM, RT	2.3/1	15
$POCl_3$, pyridine, DCM, 0-35°C	n.d.	0 [c]

[a] Ratio based on ^1H NMR spectra of a crude product. [b] Obtained as a mixture of **27** and **28**. [c] Different product obtained. n.d. - not determined.

Table S1: Spheroid invasion assay with BLM cell line at 10 µM concentration, 24 h.

compound	DMSO	**1**	**2**	**17a**	**17b**	**17c**	**17d**	**17e**	**17f**	**17g**
rel. spheroid area	1.02	0.26	0.23	1.21	1.14	0.93	1.24	0.96	0.92	1.17
SD	0.28	0.04	0.06	0.11	0.19	0.18	0.26	0.14	0.12	0.28
compound	**17f**	**17g**	**17h**	**17i**	**17j**	**20**	**22a**	**22b**	**22h**	**23a**
rel. spheroid area	0.92	1.17	1.05	1.14	1.12	0.56	1.11	1.24	0.98	1.01
SD	0.12	0.28	0.21	0.27	0.30	0.25	0.17	0.30	0.39	0.24
compound	**23b**	**23h**	**24**	**25**	**26**	**27**	**28**	**29**	**30**	
rel. spheroid area	1.10	0.93	0.46	1.15	0.88	0.89	0.72	1.05	0.50	
SD	0.21	0.38	0.07	0.30	0.11	0.27	0.15	0.22	0.27	

Figure S1: Representative images of BLM cell spheroid invasion assay with or without inhibitor. Images were taken at 0 h and 24 h. using a Leica TCS SP2 microscope (5x/0.15 dry objective). DMSO (first column) was used as a control. Scale bar 500 µm.

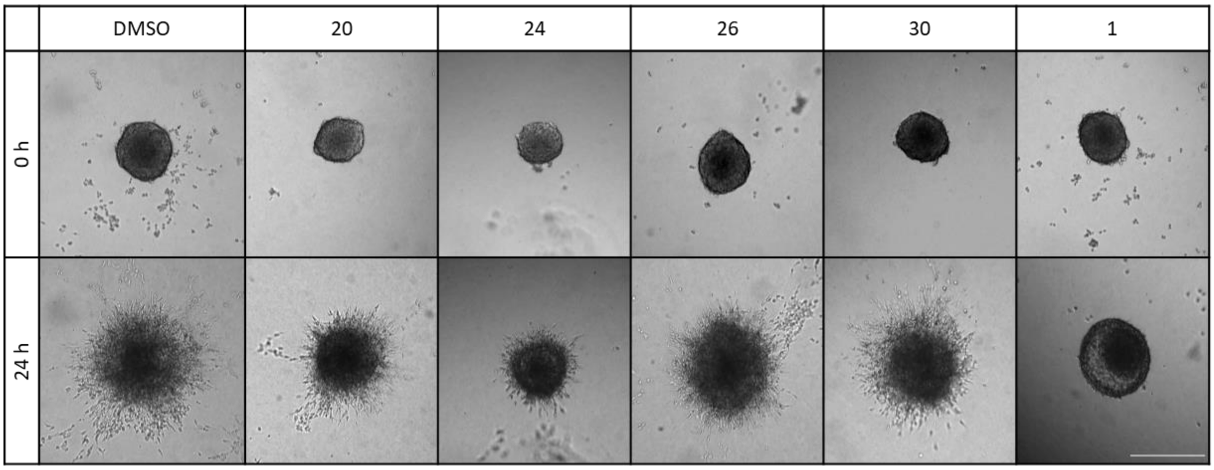

Figure S2: Actin polymerization assay at 10 μM concentration. Representative graph of one measurement.

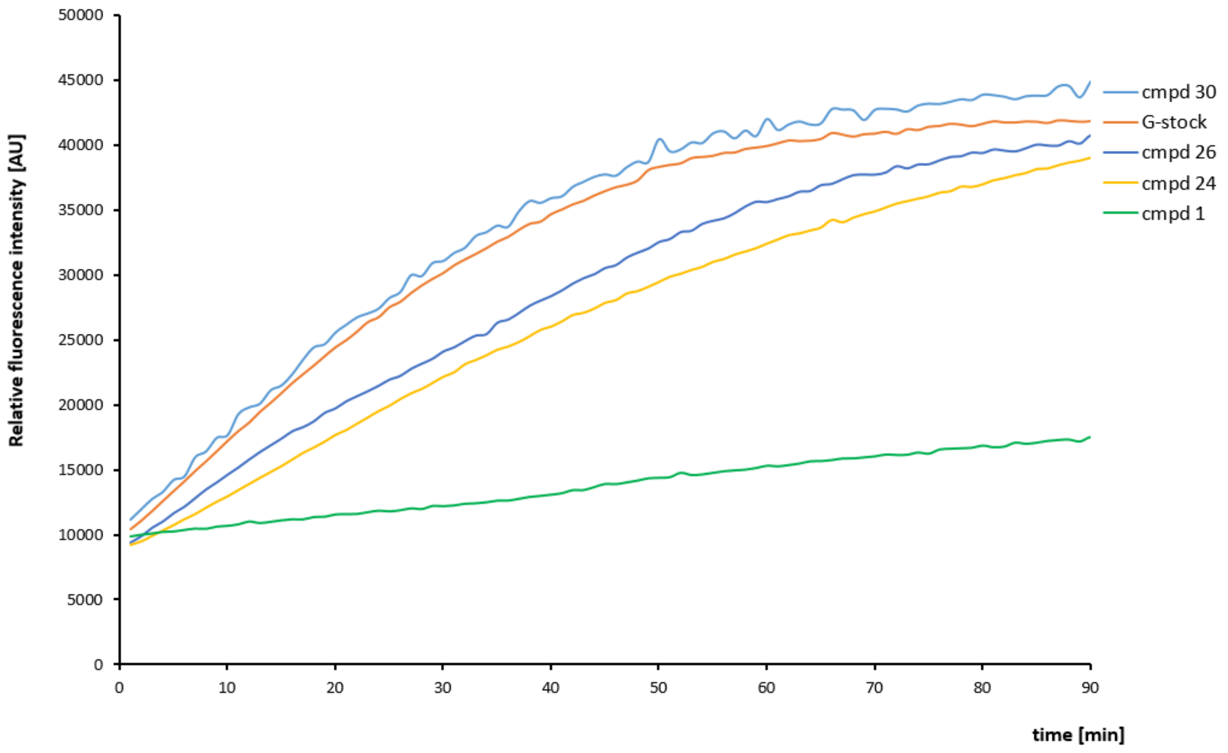

HPLC purity of final cytochalasin analogues

Separation conditions:
A: column 4x150 mm, 5 µm silica gel, 60 Å, V=1 mL/min, EtOAc/hexane – 0. min 20/80, 10. min 80/20, 12.5. min 80/20.

B: column 4x100 mm, 5 µm C18-RP, 130 Å, V=0.75 mL/min, MeOH/H_2O – 0. min 60/40, 7. min 95/5, 11. min 95/5, 14. min 60/40, 20. min 60/40.

C: column 2.1x100 mm, 1.7 µm C18-BEH, V=0.5 mL/min, ACN/H_2O – 0. min 0/100, 5. min 100/0, 6. min 100/0.

Sample	Purity (%)	Separation conditions	Retention time (min)
**17a**	98.8	A	9.12
**17b**	95.5	A	9.60
**17c**	95.1	A	8.67
**17d**	98.3	A	9.79
**17e**	98.9	A	9.65
**17f**	98.8	A	8.86
**17g**	98.0	A	10.04
**17h**	98.4	A	10.06
**17i**	98.0	A	9.72
**17j**	99.0	A	11.38
**20**	97.1	A	7.60
**22a**	98.4	A	10.62
**22b**	96.6	A	10.31
**22h**	99.9	A	10.57
**23a**	95.8	C	4.44
**23b**	99.3	A	10.91
**23h**	98.9	A	10.98
**24**	99.3	A	9.98
**25**	98.0	B	9.14
**26**	97.6	A	7.21
**27**	99.9	A	7.07
**28**	97.9	A	7.77
**29**	95.8	B	8.29
**30**	99.9	A	13.54

17a – purity 98.8 %, T_R = 9.12 min

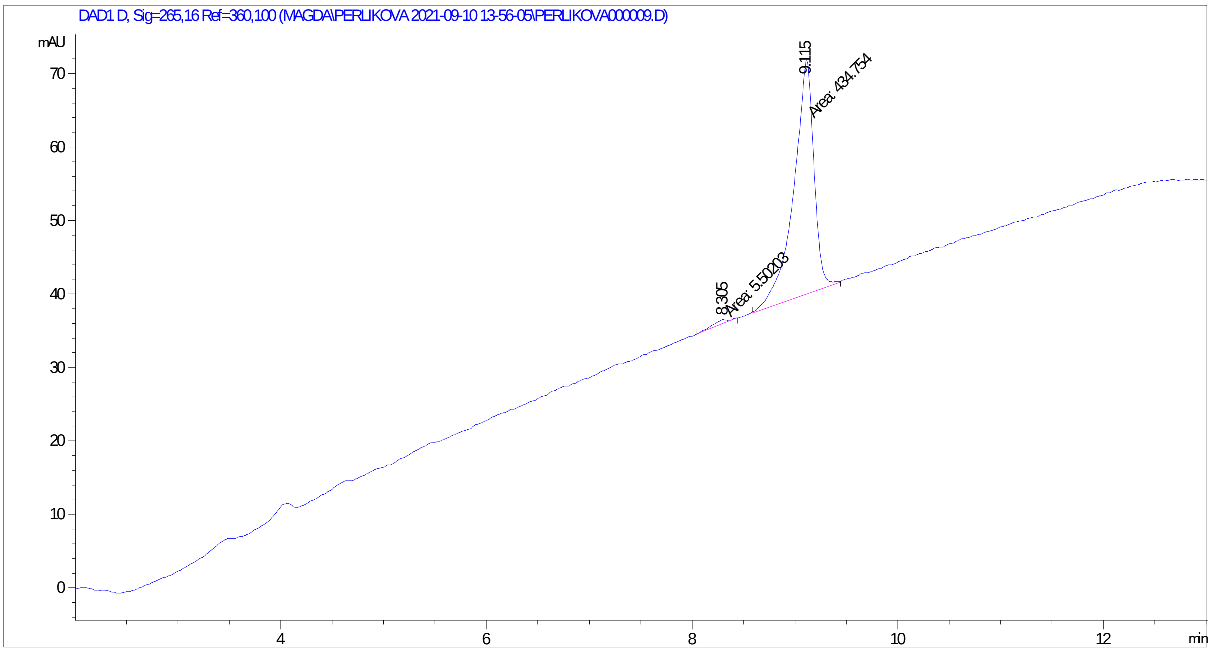

17b – purity 95.5 %, T_R = 9.60 min

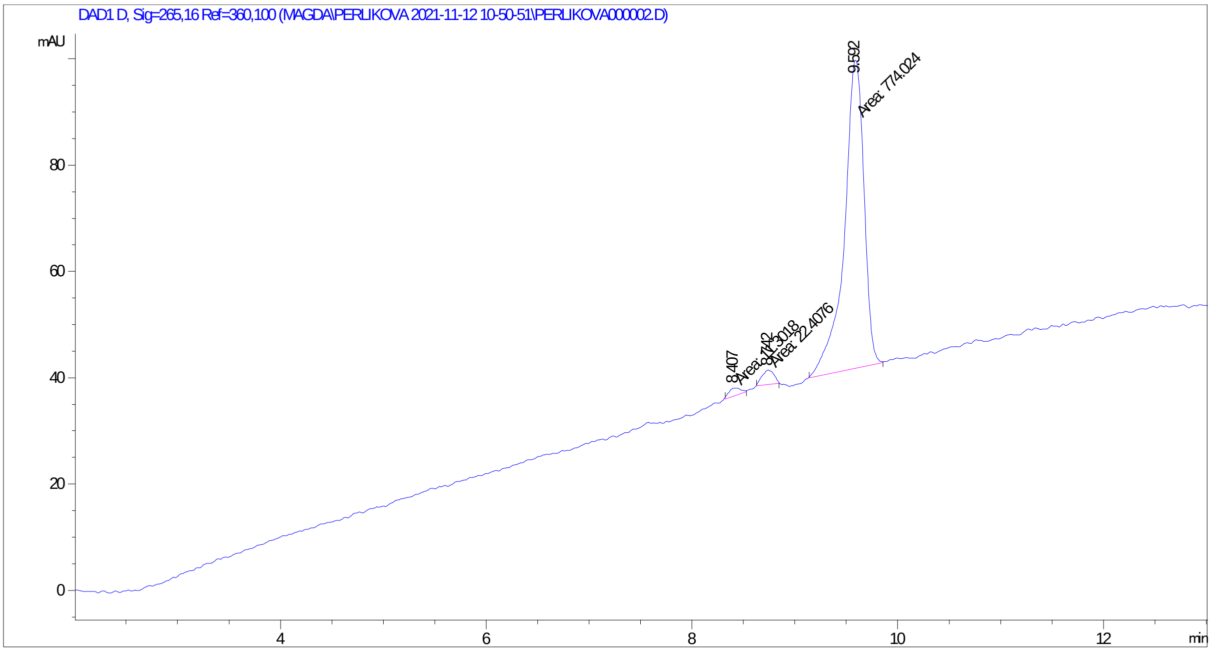

17c – purity 95.1 %, T_R = 8.67 min

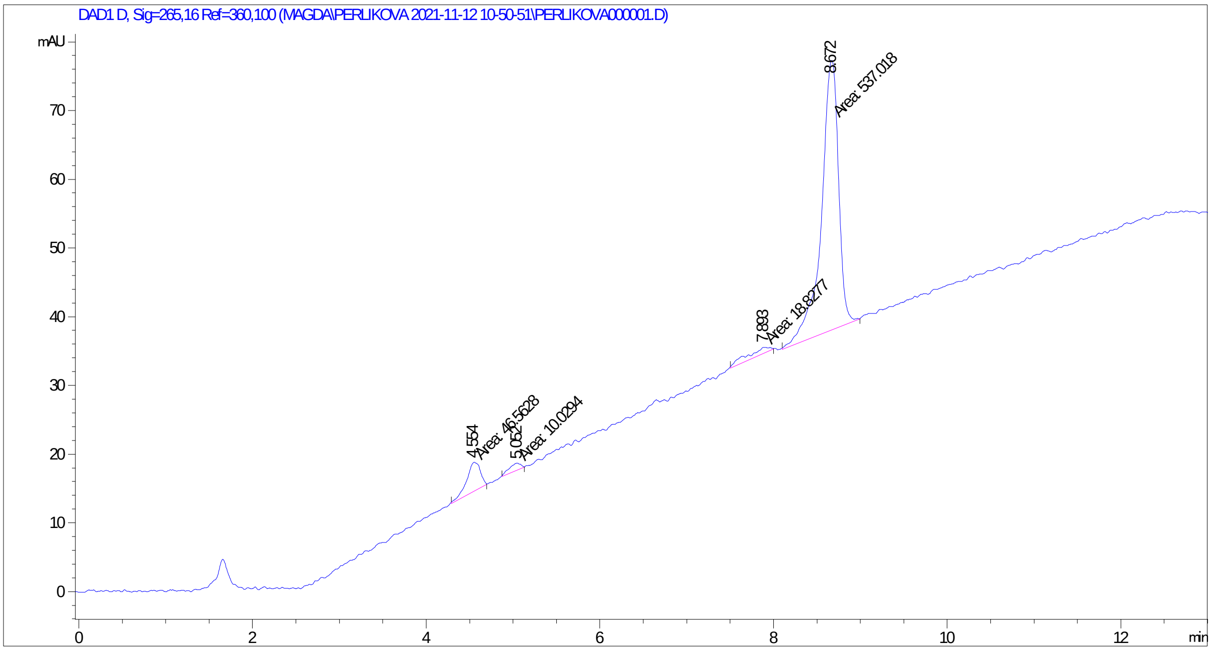

17d – purity 98.3 %, T_R = 9.79 min

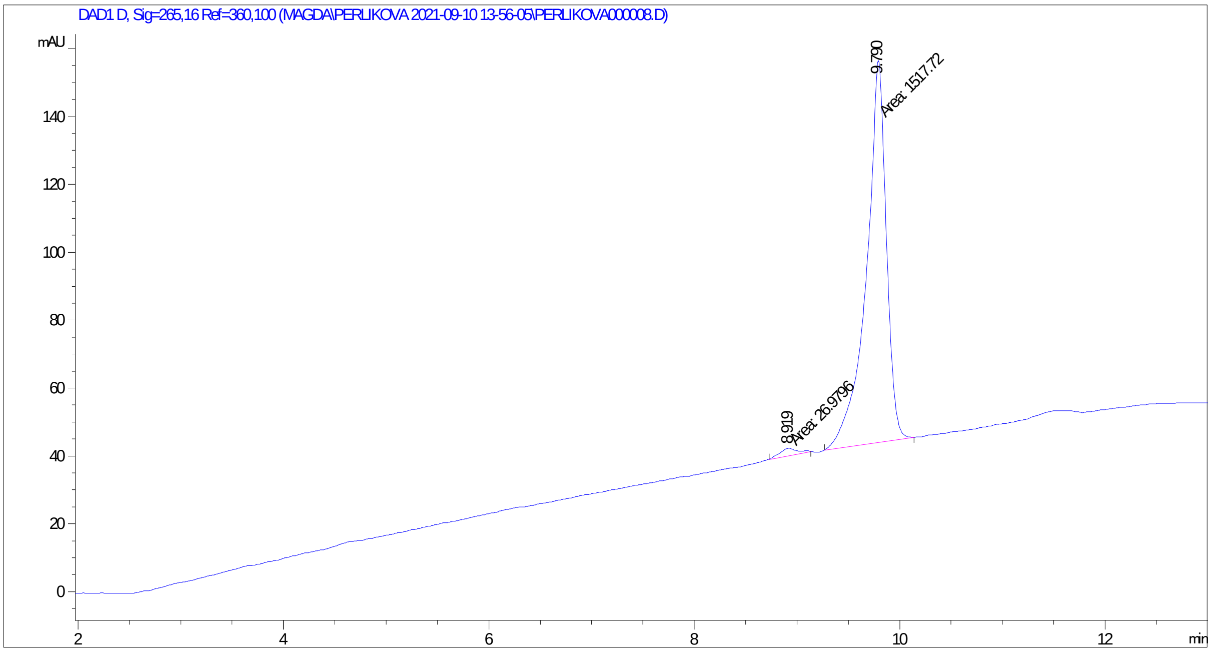

17e – purity 98.9 %, T_R = 9.65 min

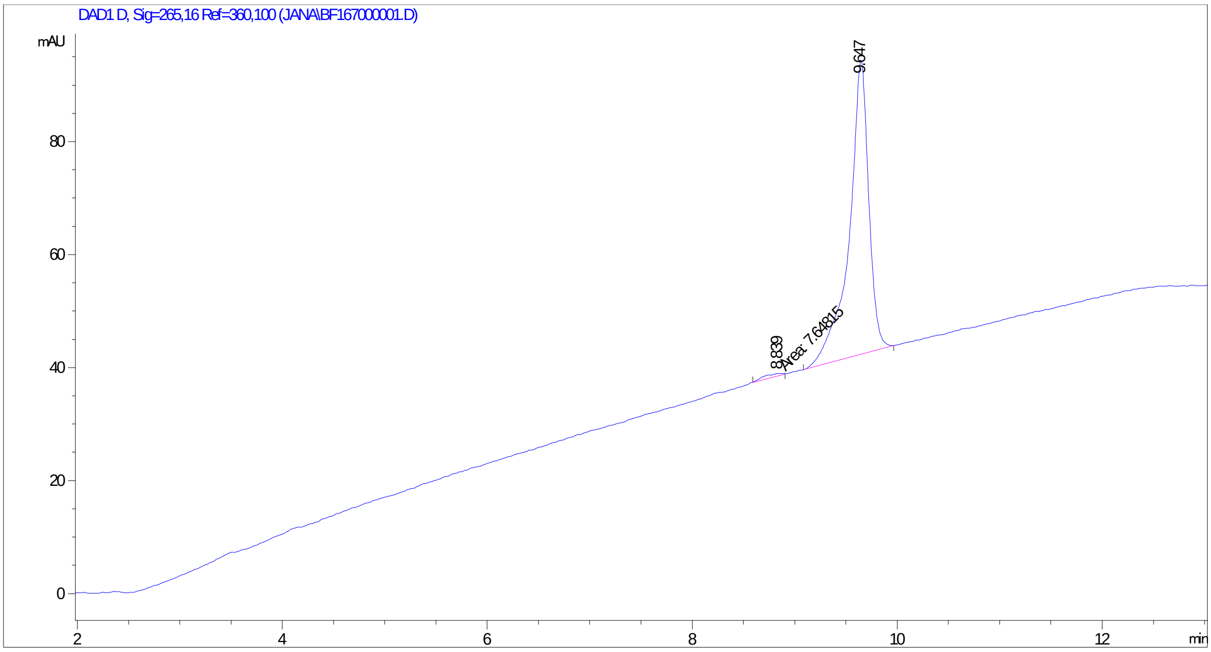

17f – purity 98.8 %, T_R = 8.86 min

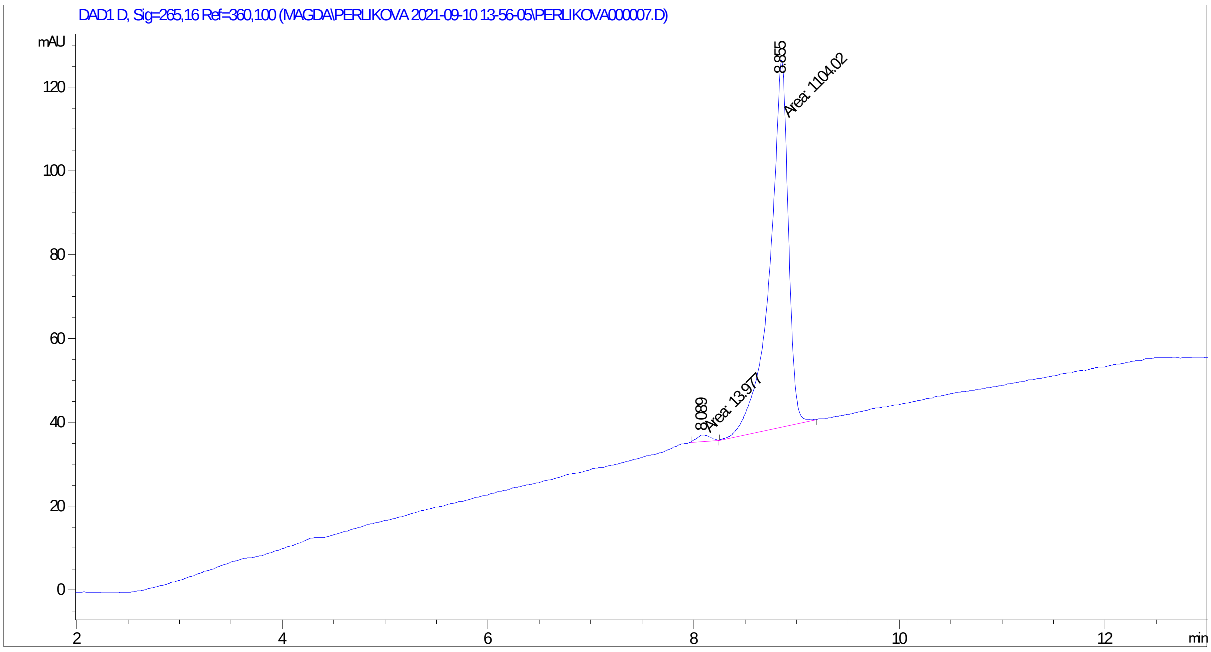

17g – purity 98.0 %, T_R = 10.04 min

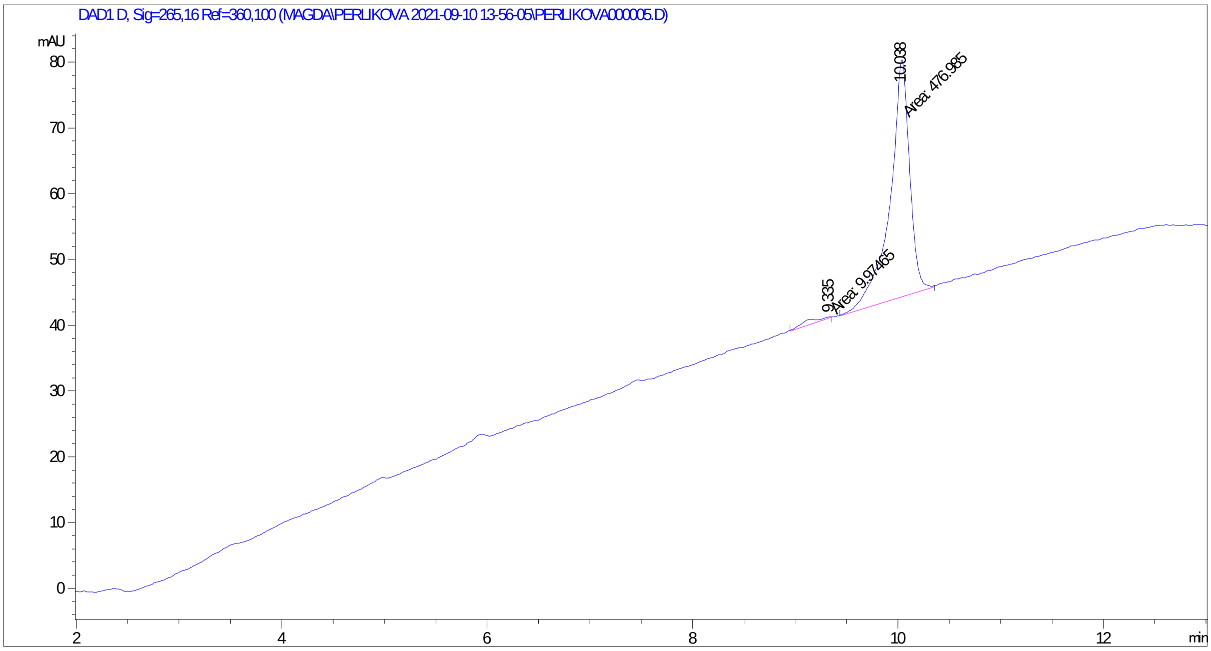

17h – purity 98.4 %, T_R = 10.06 min

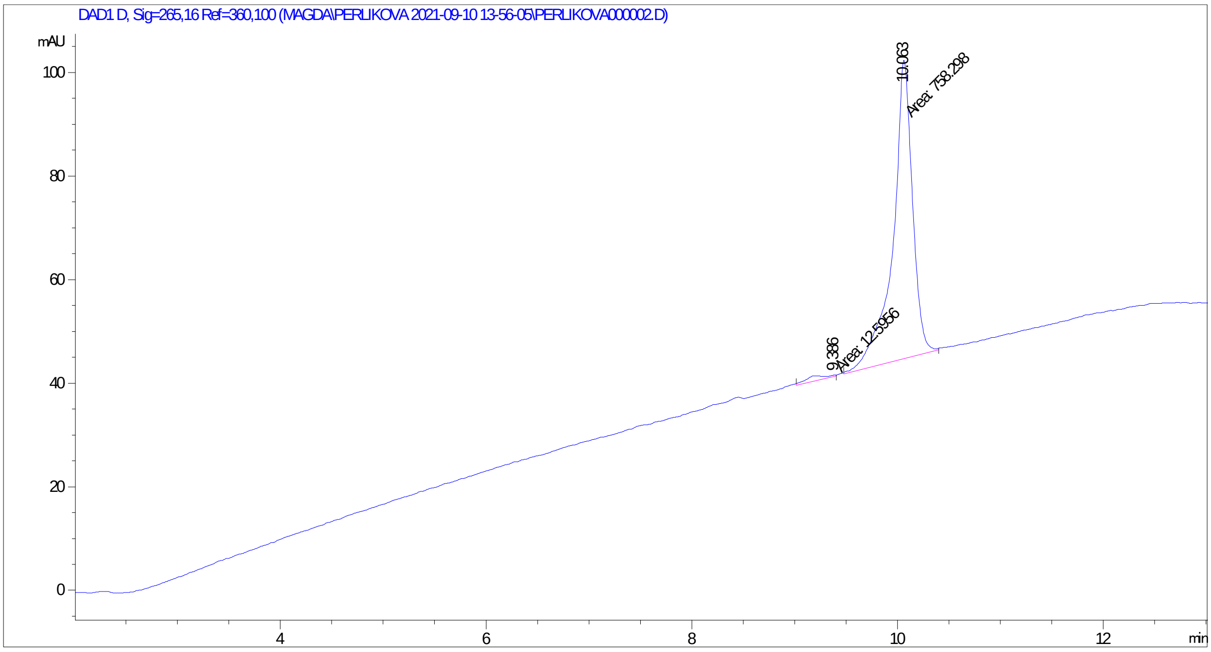

17i – purity 98.0 %, T_R = 9.72 min

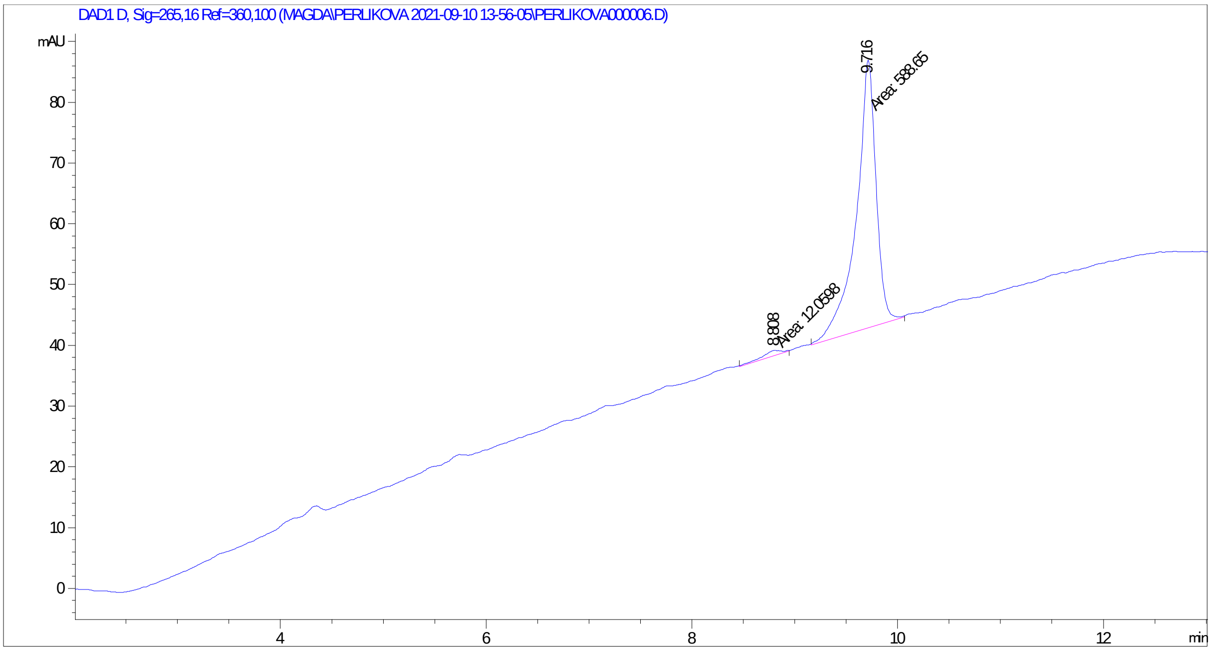

17j – purity 99.0 %, T_R = 11.38 min

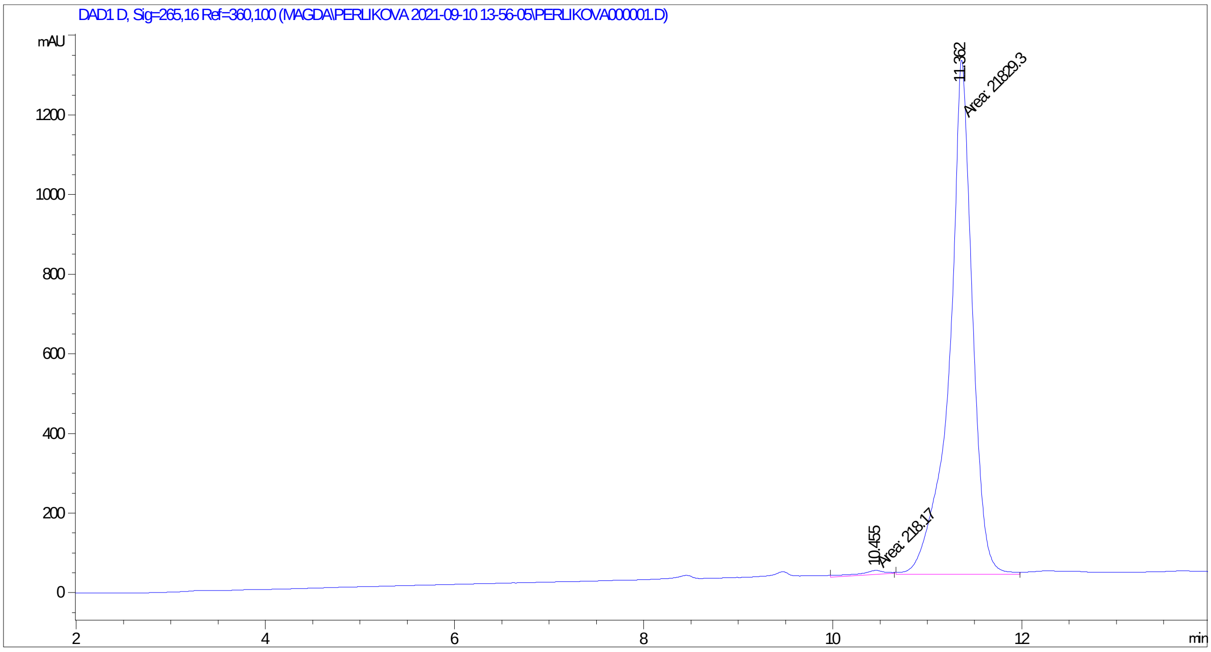

20 – purity 97.1 %, T_R = 7.60 min

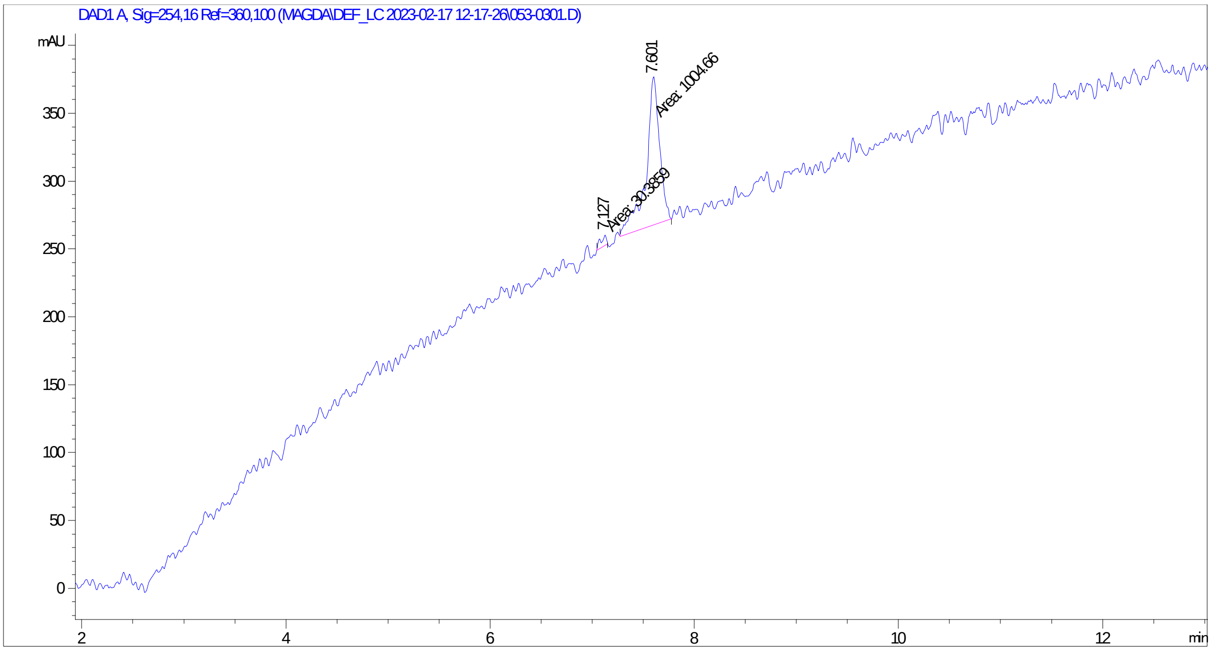

22a – purity 98.4 %, T_R = 10.62 min

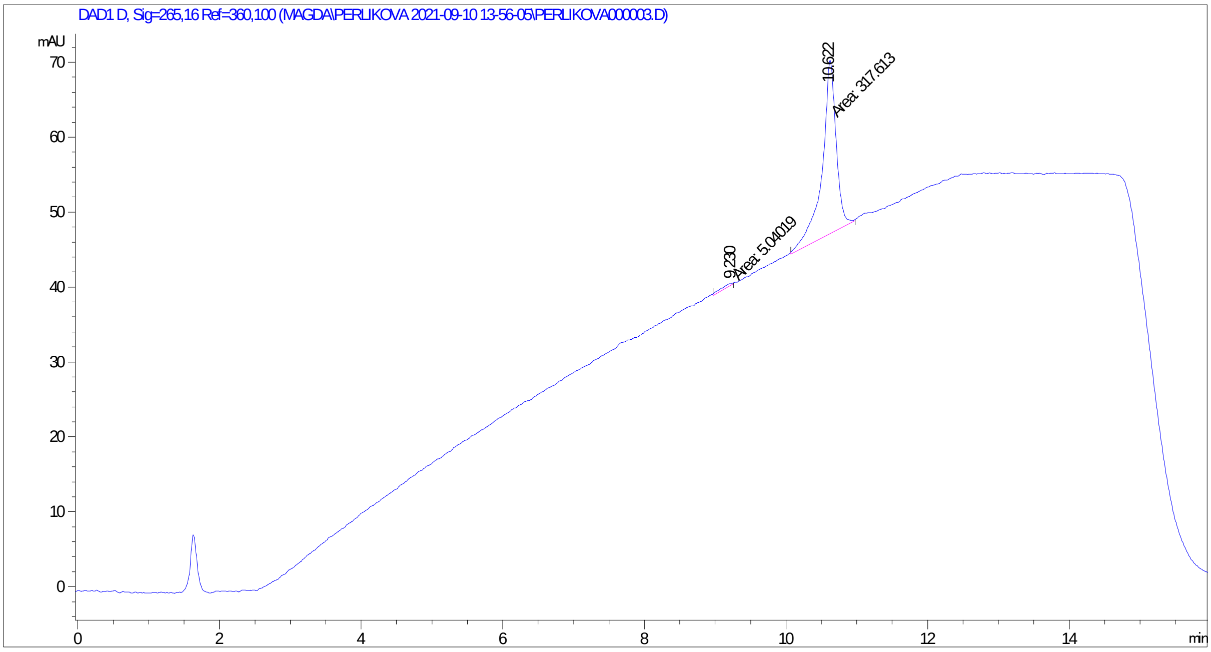

22b – purity 96.6 %, T_R = 10.31 min

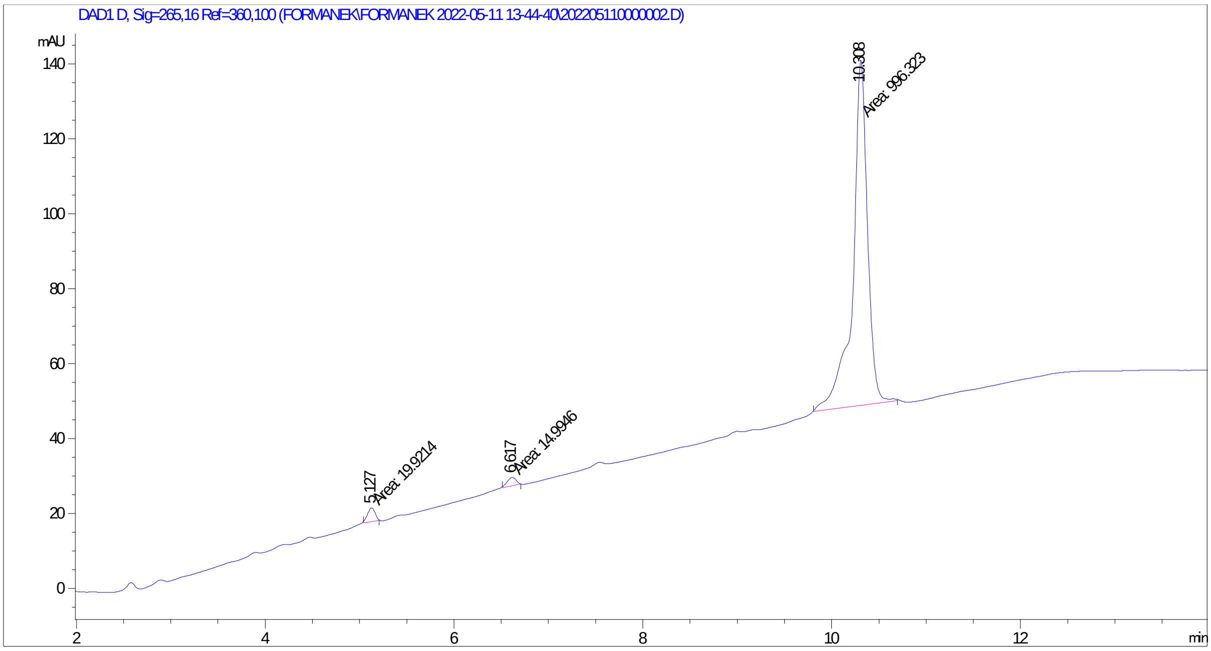

22h – purity 99.9 %, T_R = 10.57 min

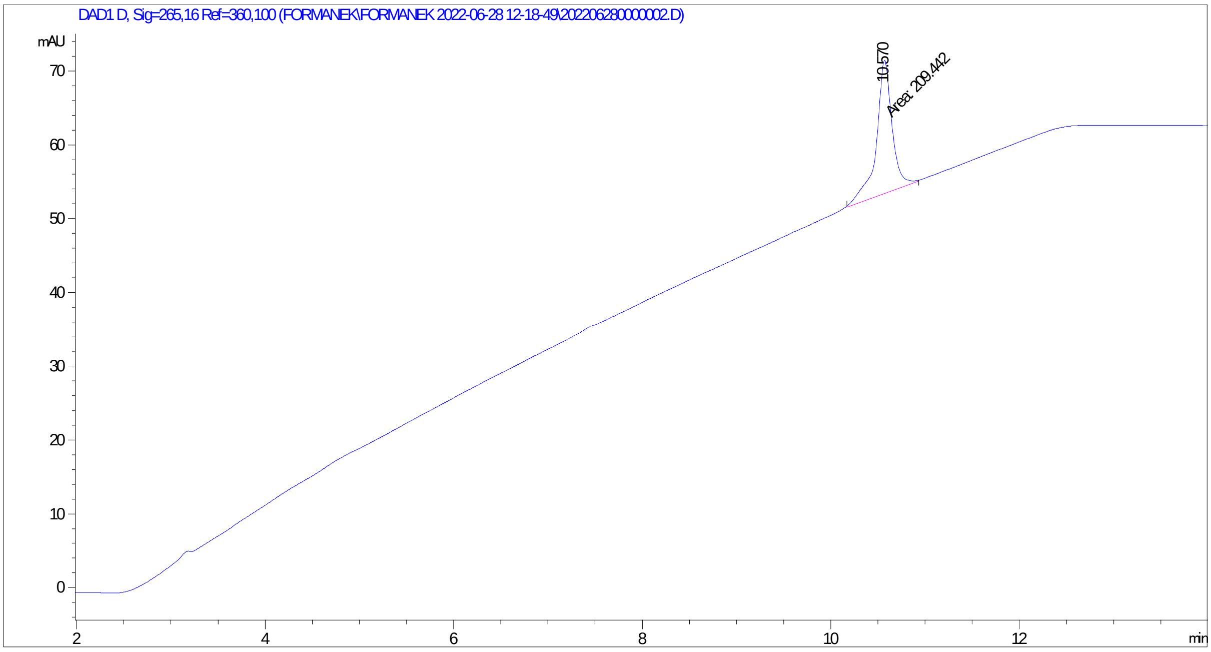

23a – purity 95.8 %, T_R = 4.44 min

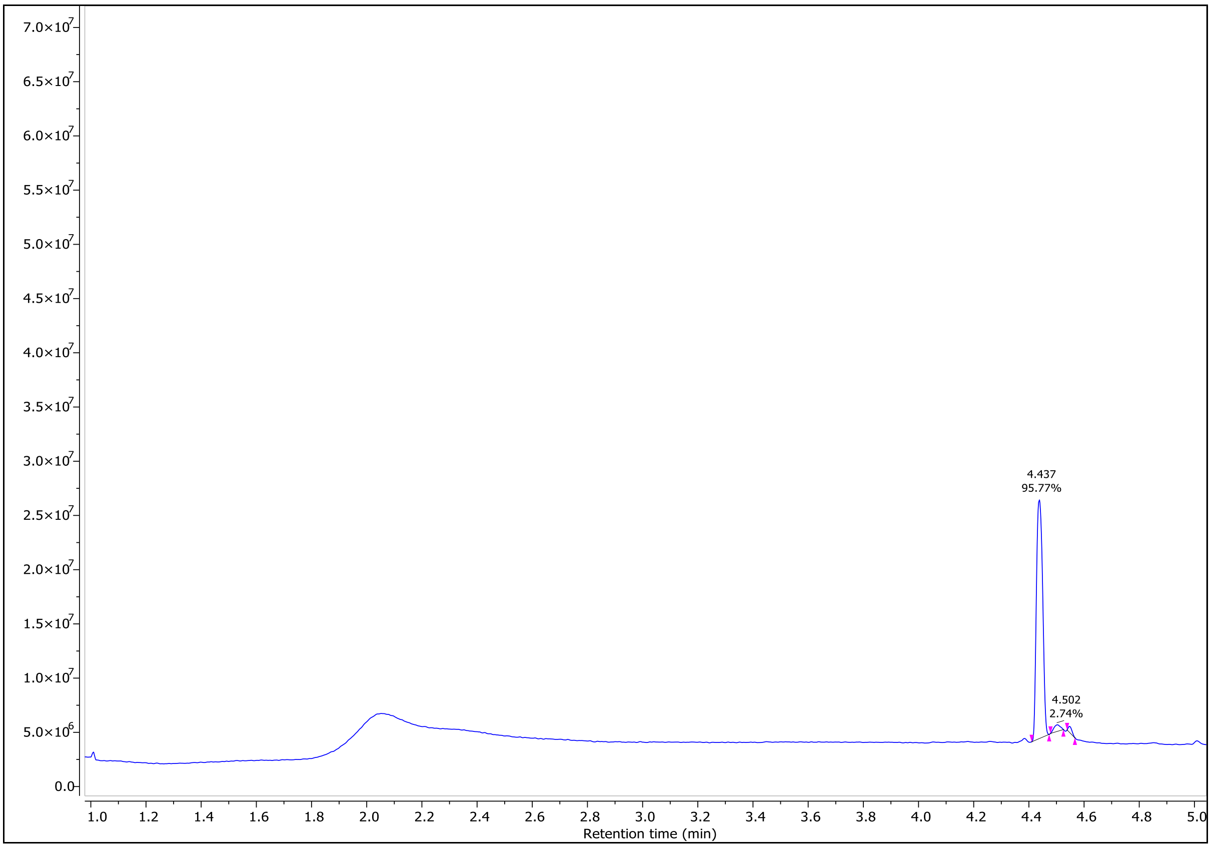

23b – purity 99.3 %, T_R = 10.91 min

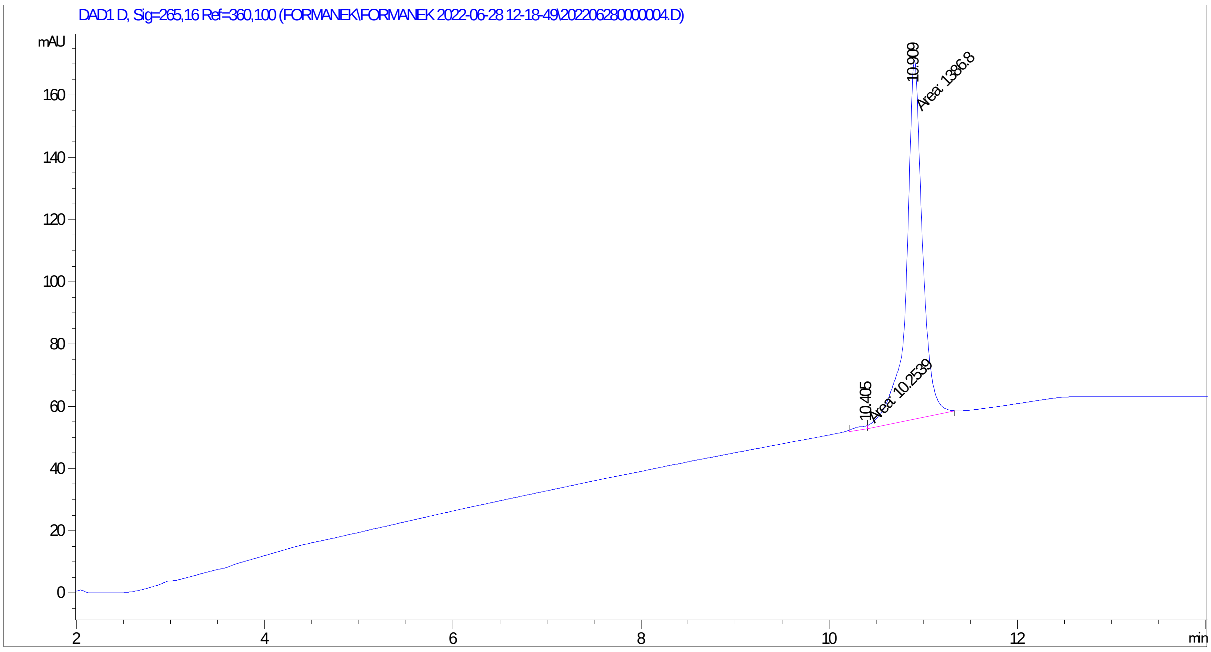

23h – purity 98.9 %, T_R = 10.98 min

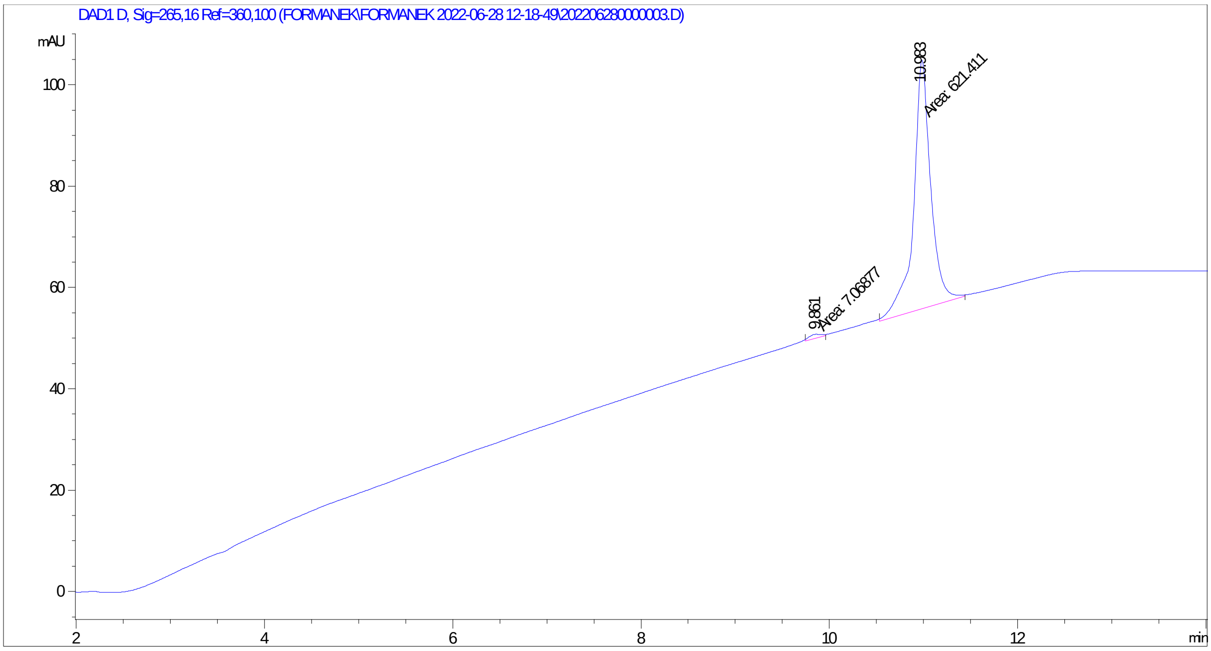

24 – purity 99.3 %, T_R = 9.98 min

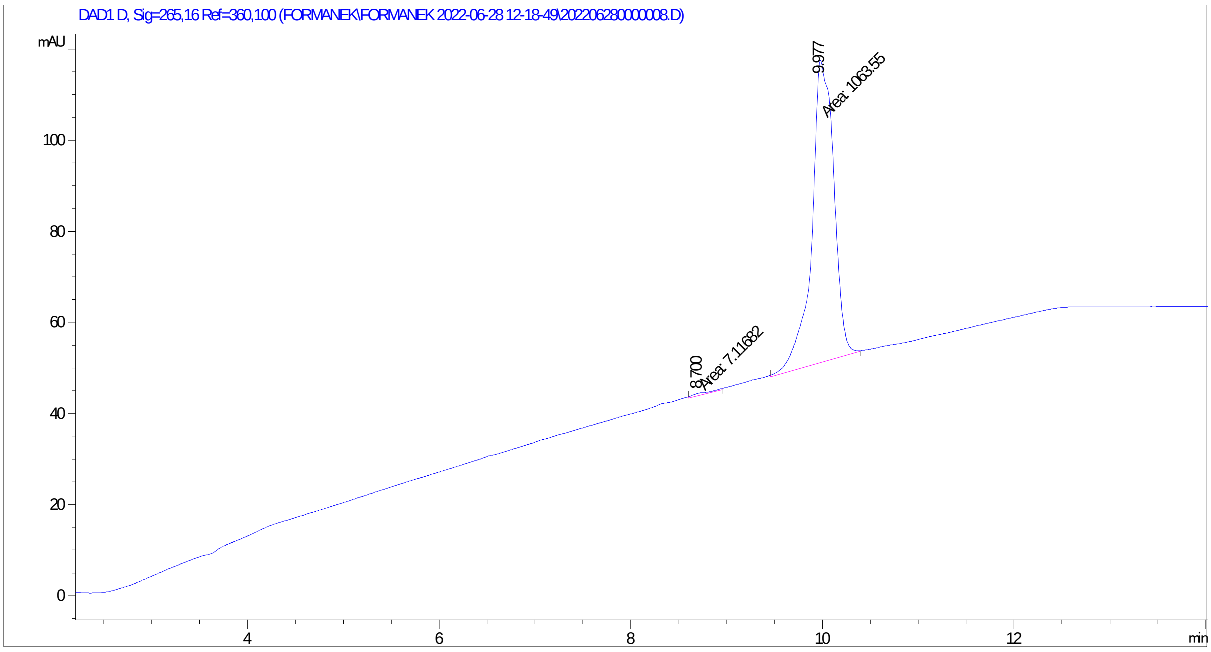

25 – purity 98.0 %, T_R = 9.14 min

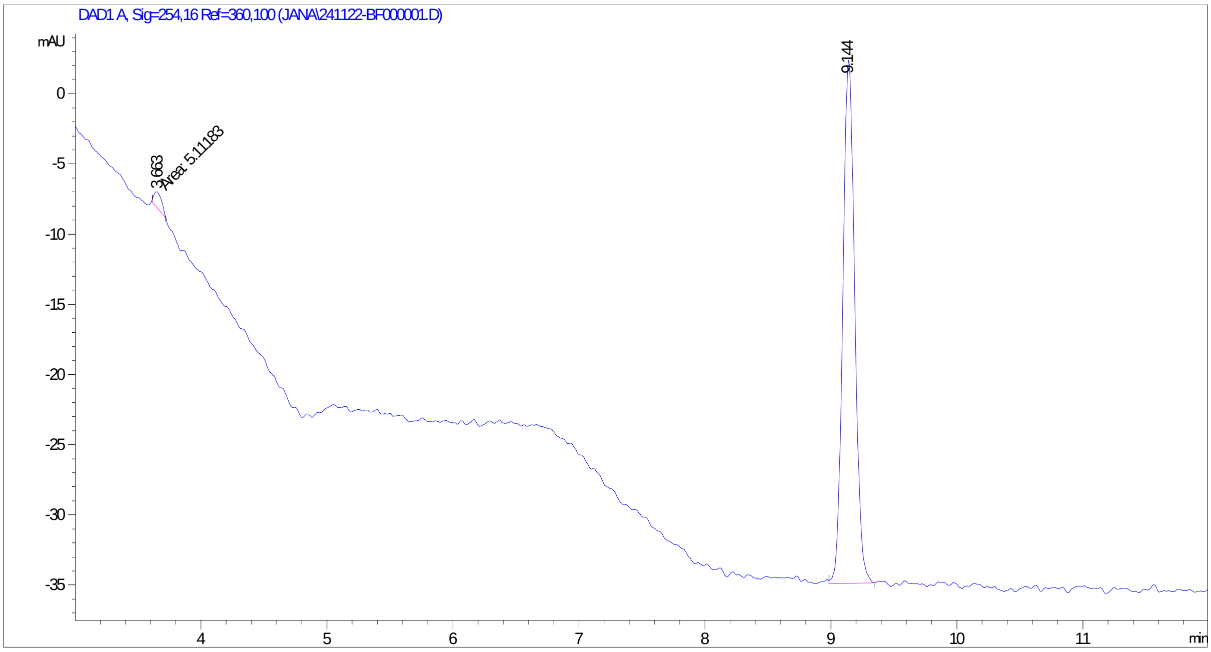

26 – purity 97.6 %, T_R = 7.21 min

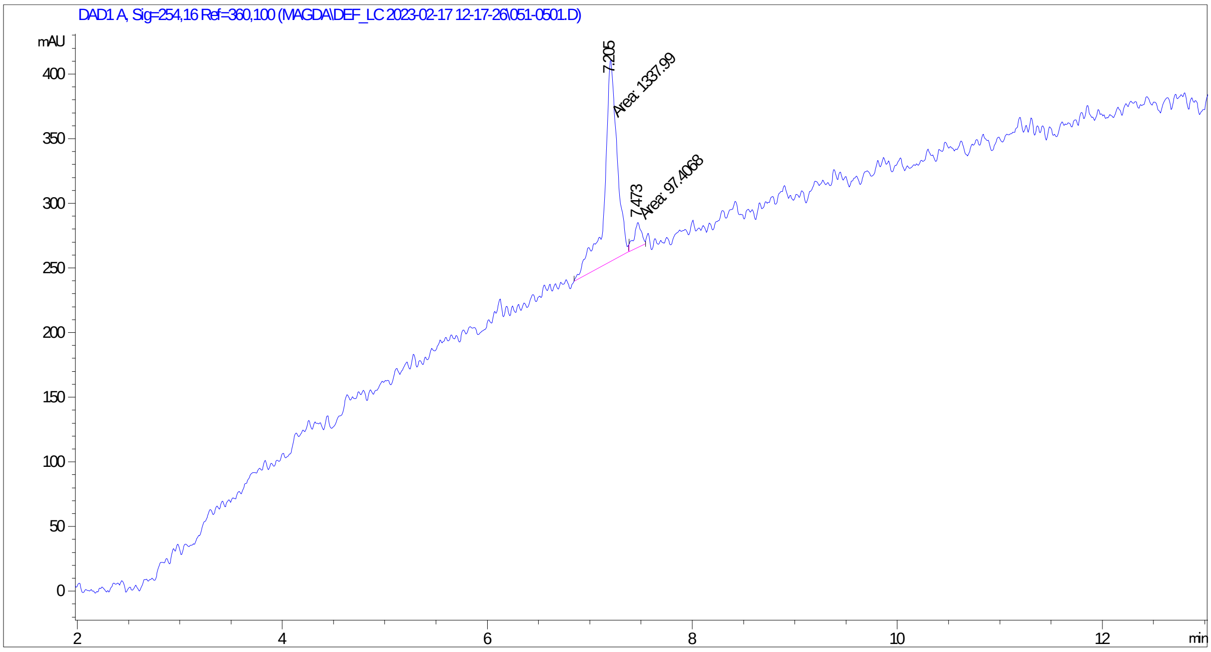

27 – purity 99.9 %, T_R = 7.07 min

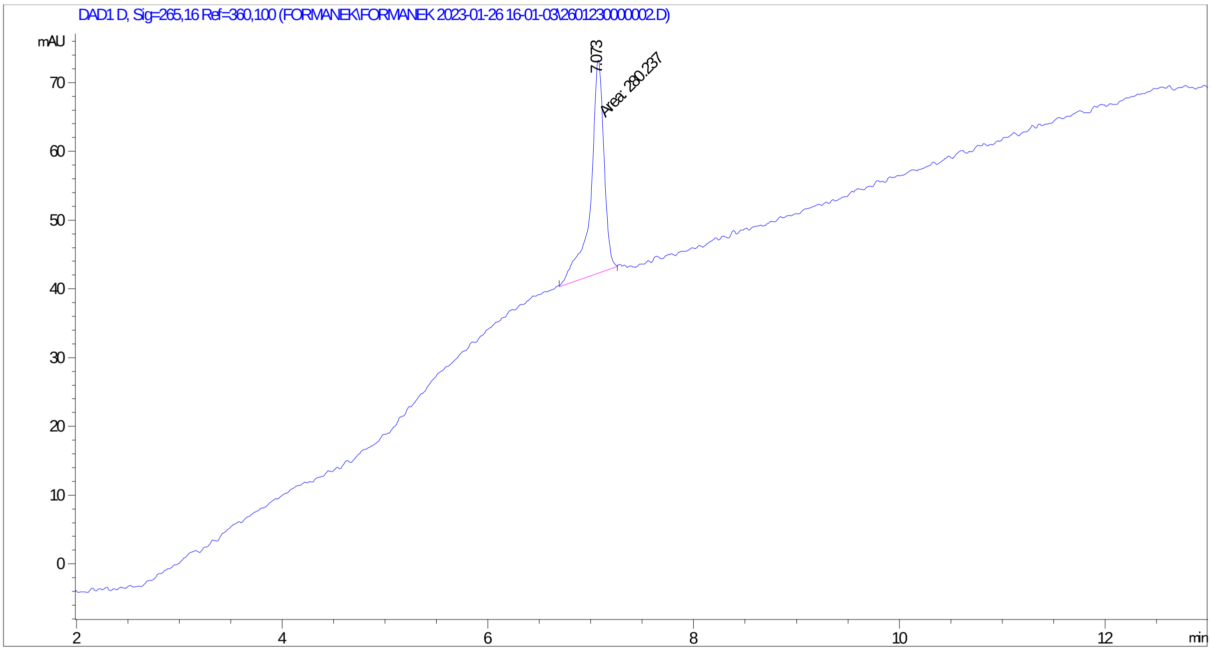

28 – purity 97.9 %, T_R = 7.77 min

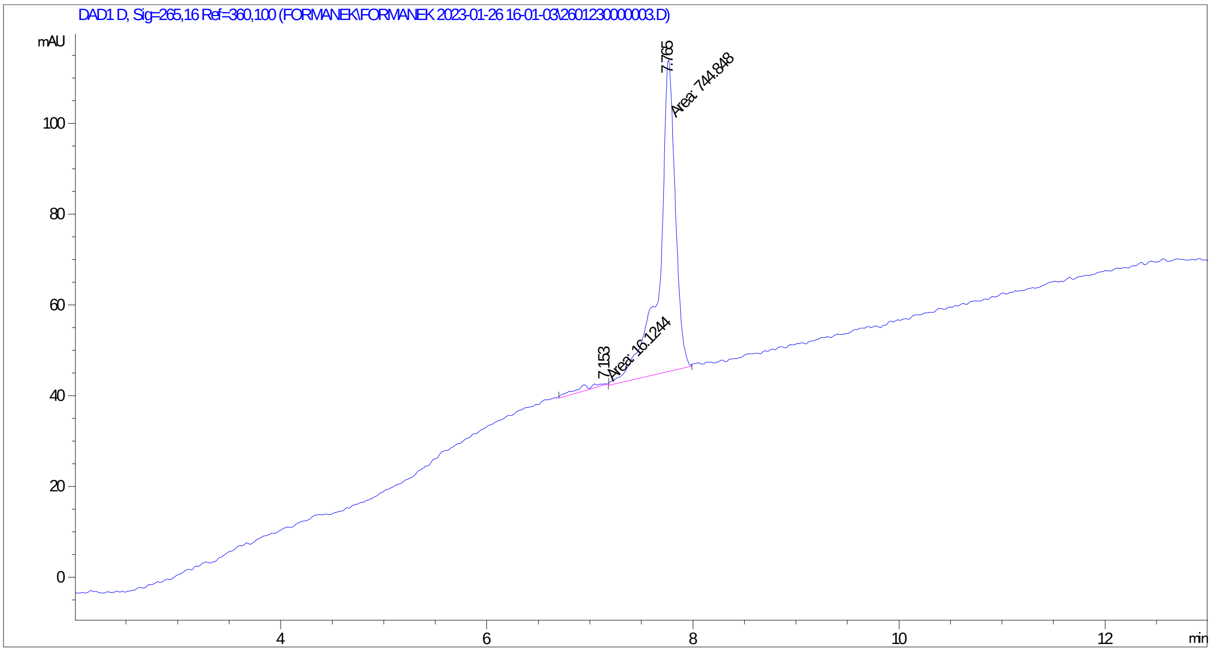

29 – purity 95.8 %, T_R = 8.29 min

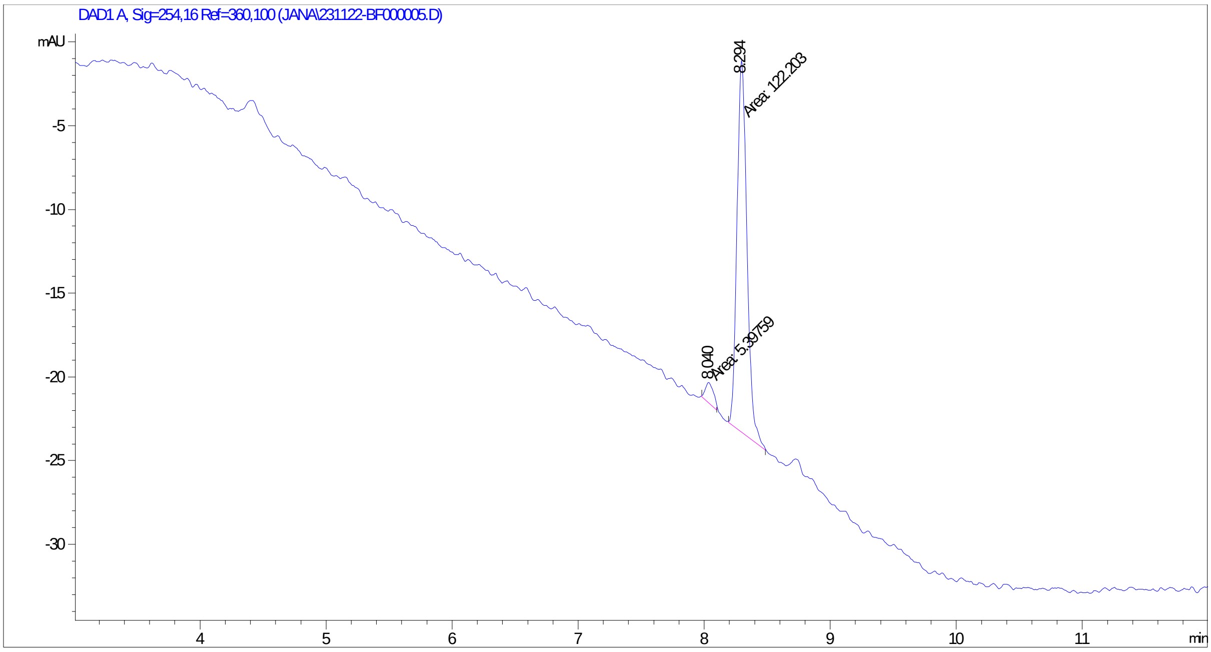

30 – purity 99.9 %, T_R = 13.54 min

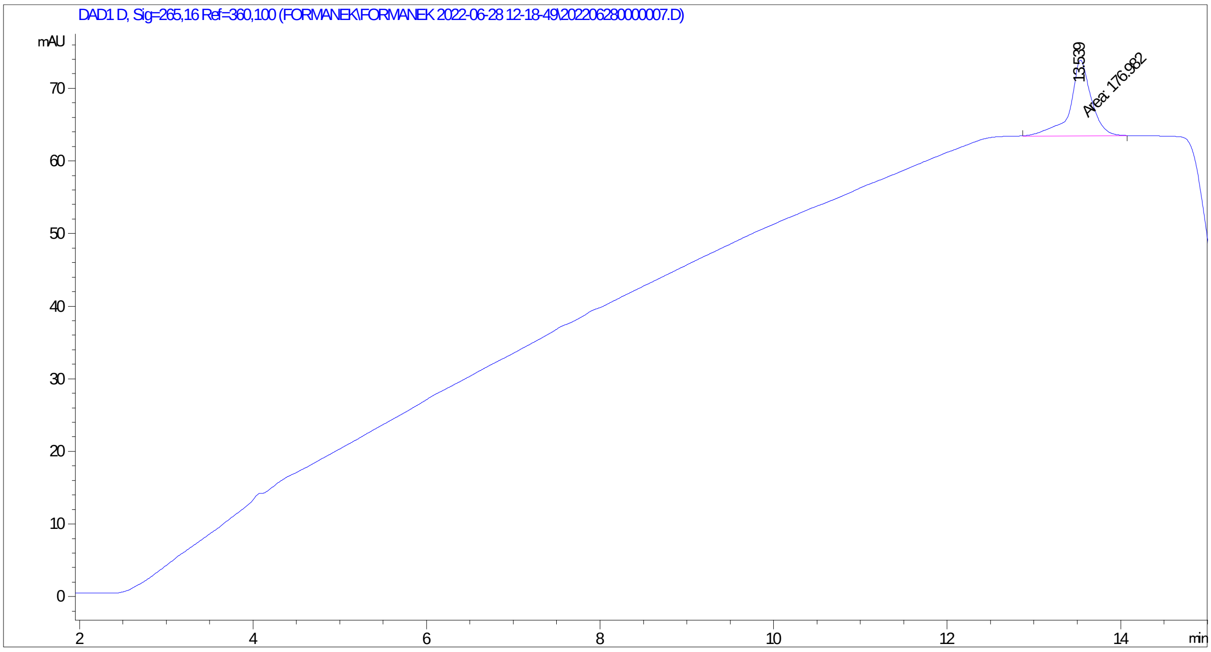

Copies of NMR spectra

S4

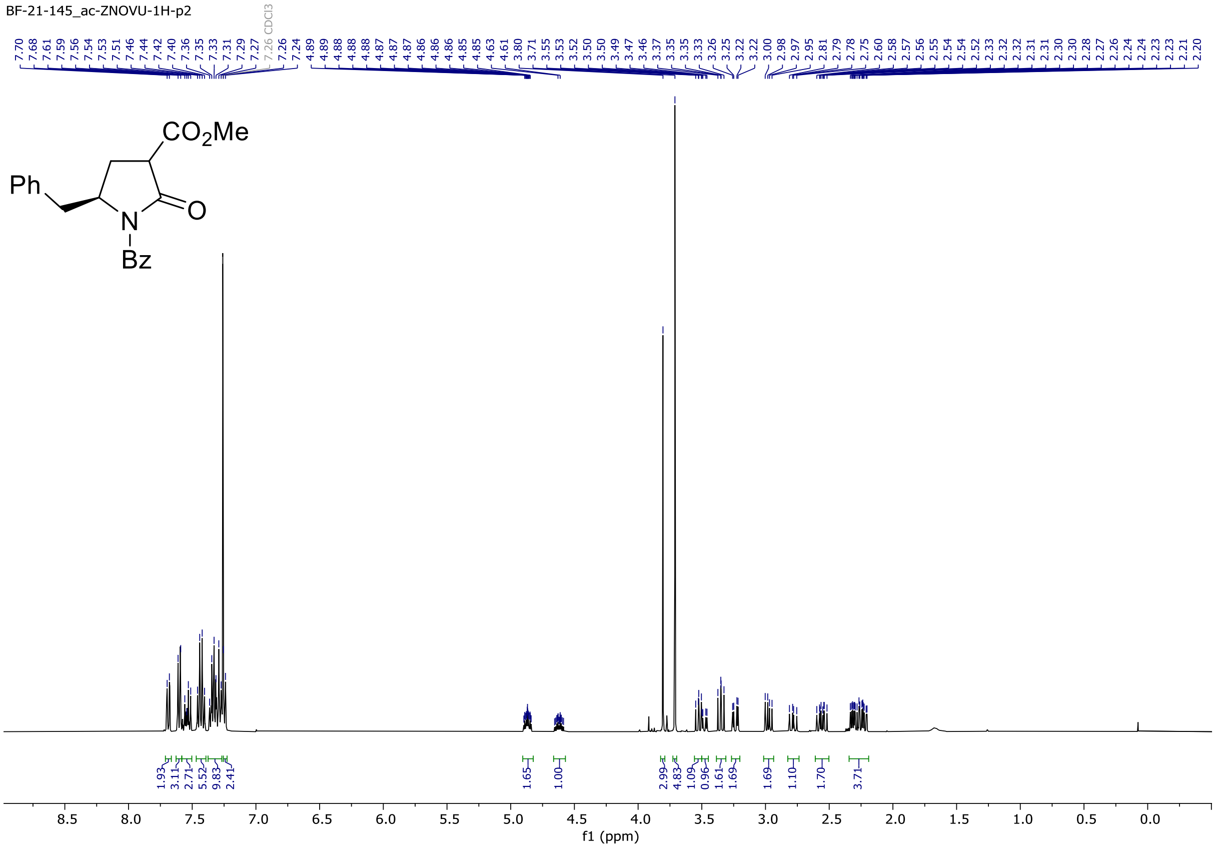

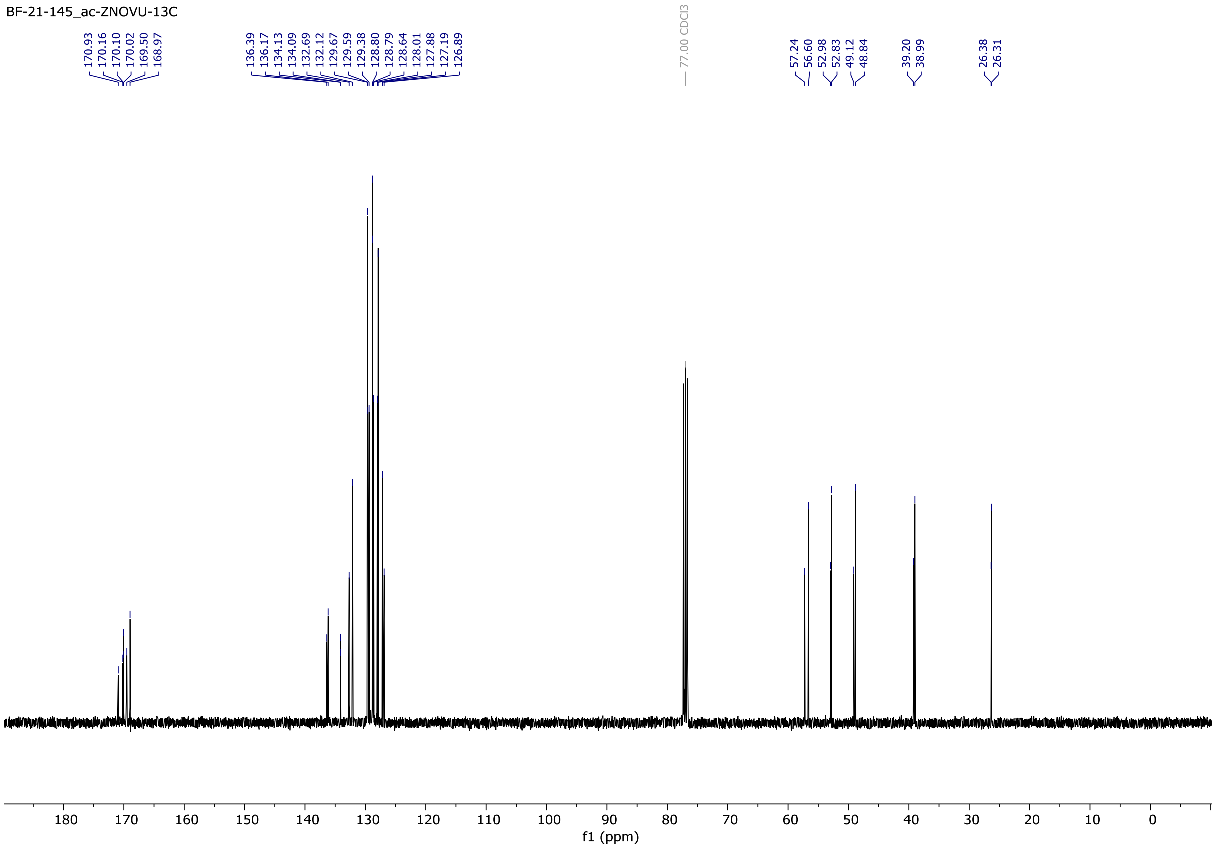

5

DD088-selenide

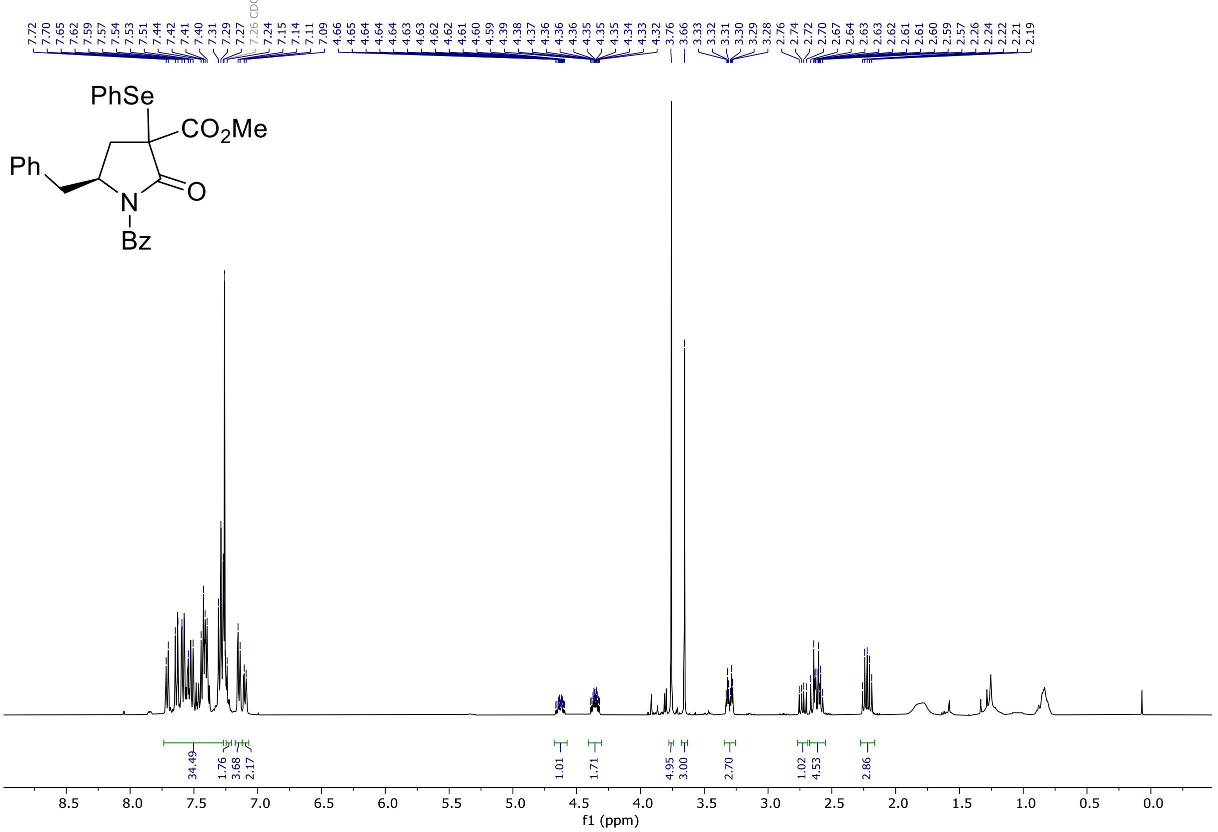

DD088-selenide

Perlikova APT Experiment

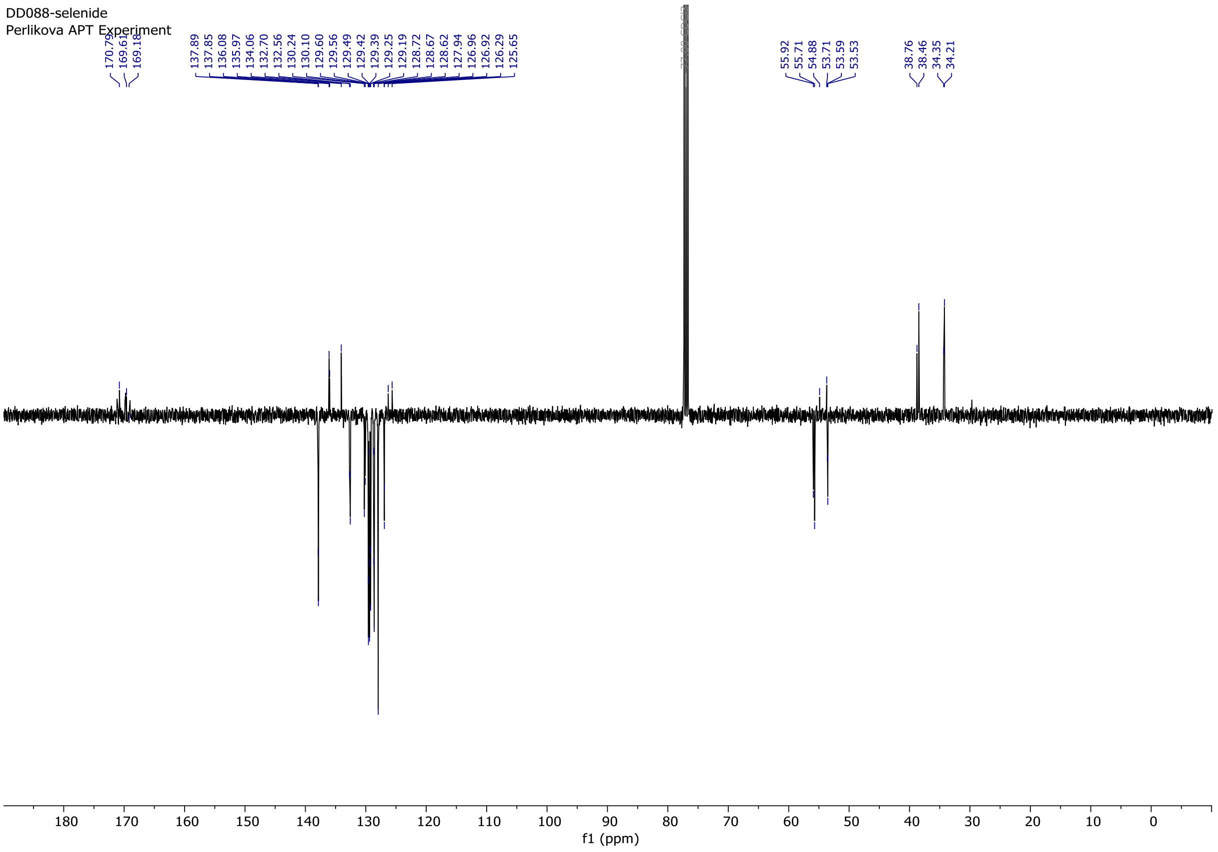

11

BF033.1.fid

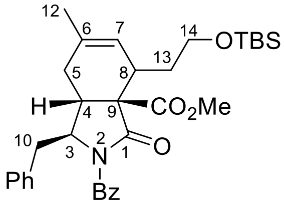

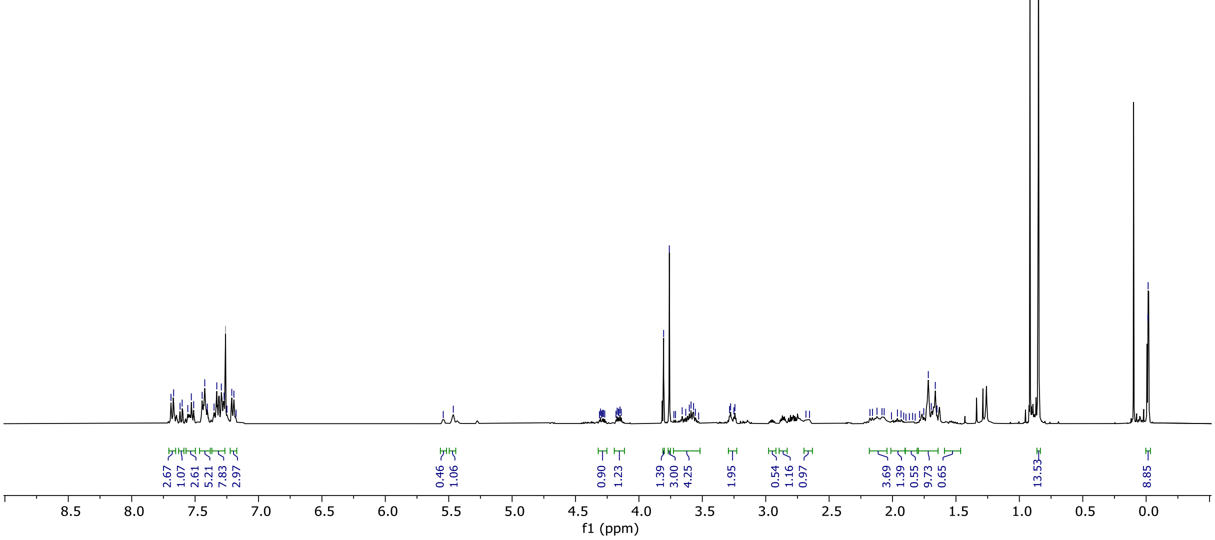

BF033.3.fid

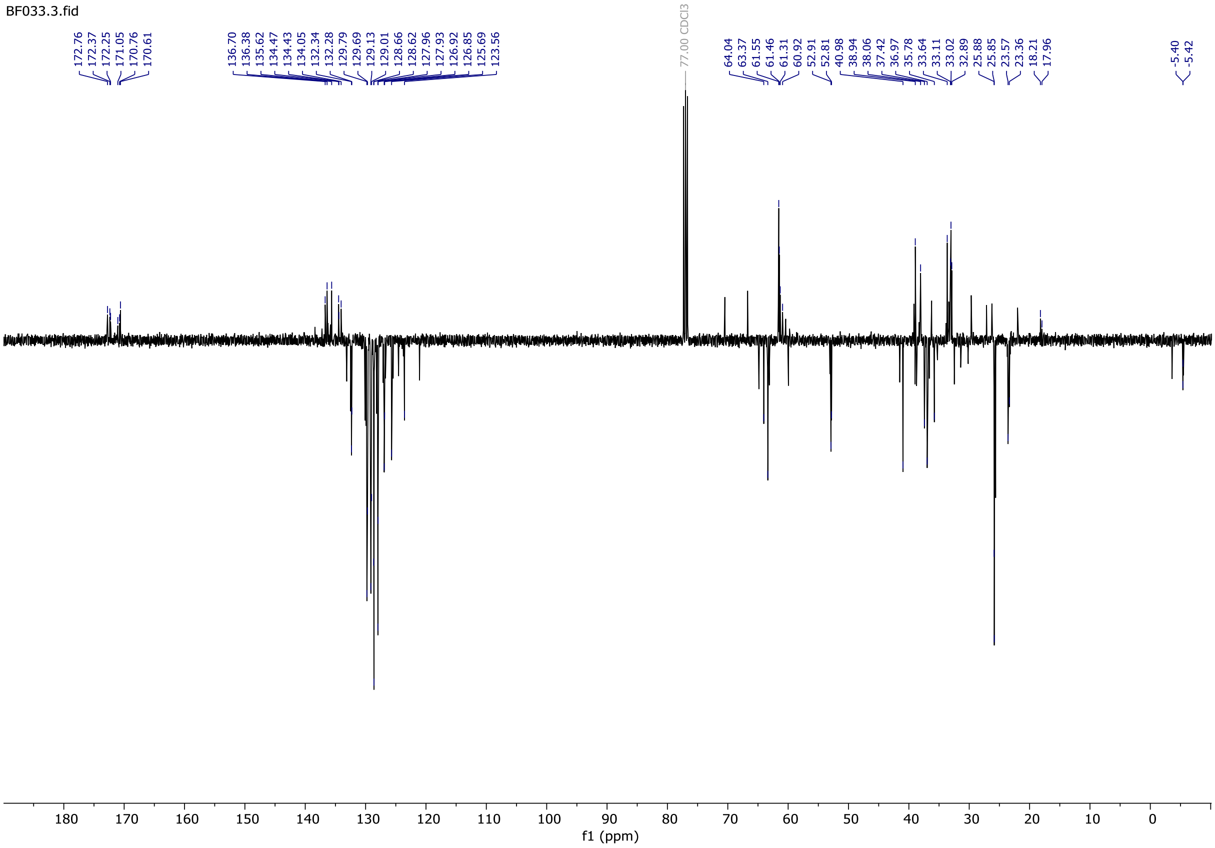

12

BF-21-070-ac-2F

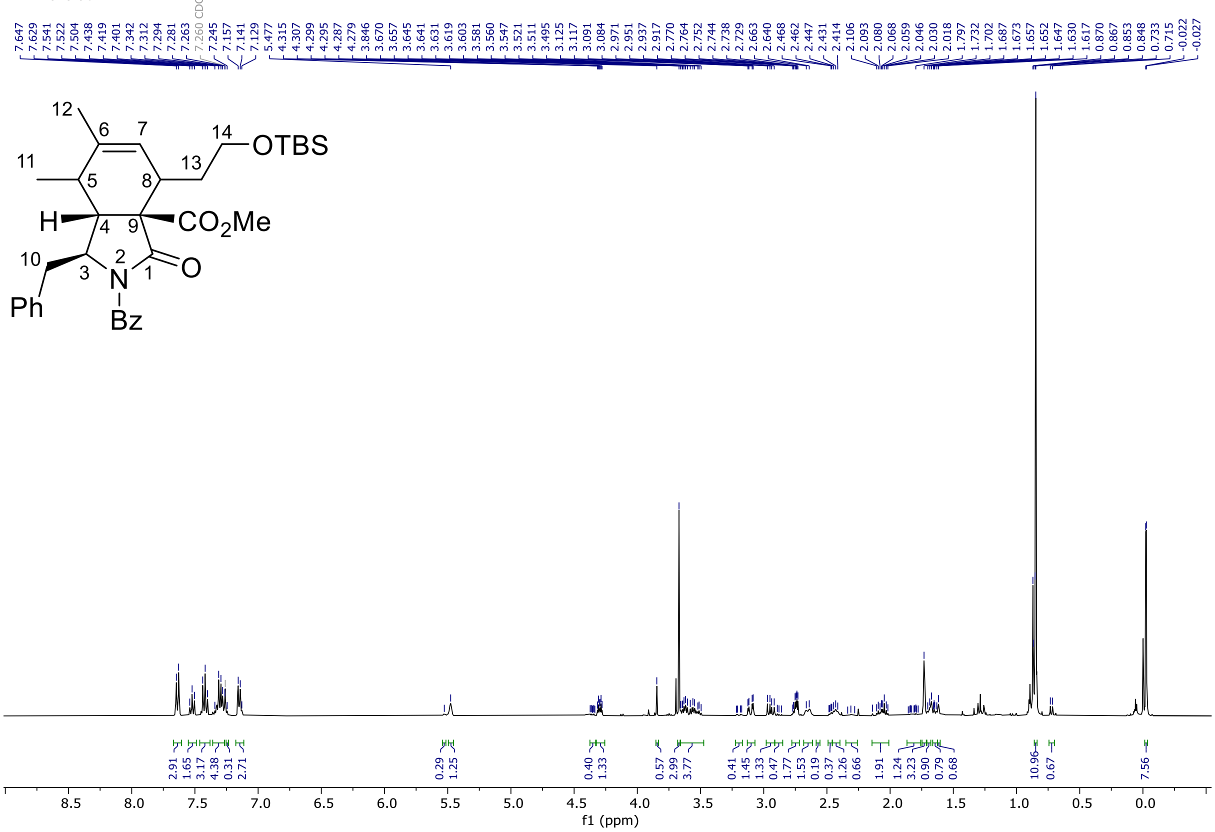

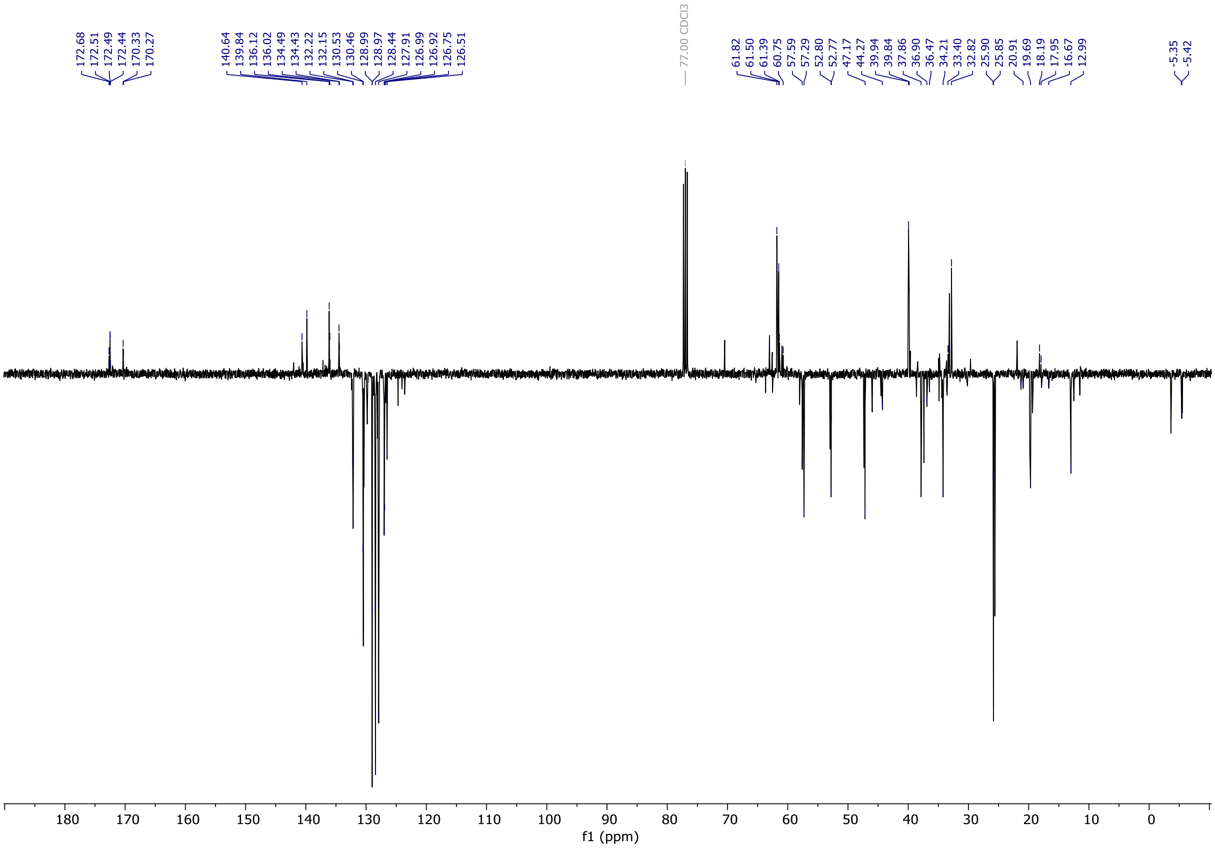

13

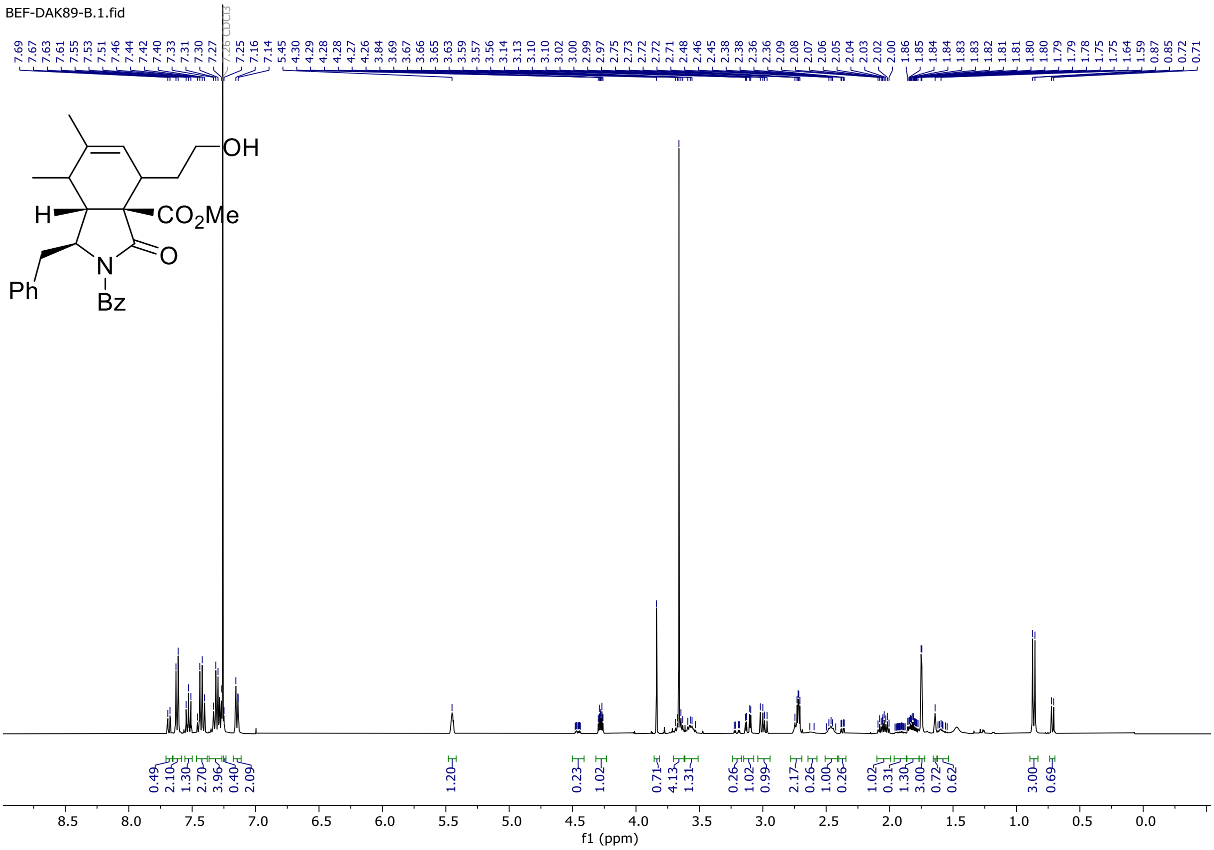
BEF-DAK89-B.1.fid
7.69 7.67 7.63 7.61 7.55 7.53 7.51 7.46 7.44 7.42 7.40 7.33 7.31 7.30 7.27 7.26 CDCl3 7.25 7.16 7.14 5.45 4.30 4.29 4.28 4.28 4.27 4.26 3.84 3.69 3.67 3.66 3.65 3.63 3.59 3.57 3.56 3.14 3.13 3.10 3.10 3.02 3.00 2.99 2.97 2.75 2.73 2.72 2.72 2.71 2.48 2.46 2.45 2.38 2.38 2.36 2.36 2.09 2.08 2.07 2.06 2.05 2.04 2.03 2.02 2.00 1.86 1.85 1.84 1.84 1.83 1.83 1.82 1.81 1.81 1.80 1.80 1.79 1.79 1.78 1.75 1.64 1.59 0.87 0.85 0.72 0.71
OH
H
CO2Me
O
N
Ph
Bz
0.49 2.10 1.30 2.70 3.96 0.40 2.09 1.20 0.23 1.02 0.71 4.13 1.31 0.26 1.02 0.99 2.17 0.26 1.00 0.26 1.02 0.31 1.30 3.00 0.72 0.62 3.00 0.69
8.5 8.0 7.5 7.0 6.5 6.0 5.5 5.0 4.5 4.0 3.5 3.0 2.5 2.0 1.5 1.0 0.5 0.0
f1 (ppm)

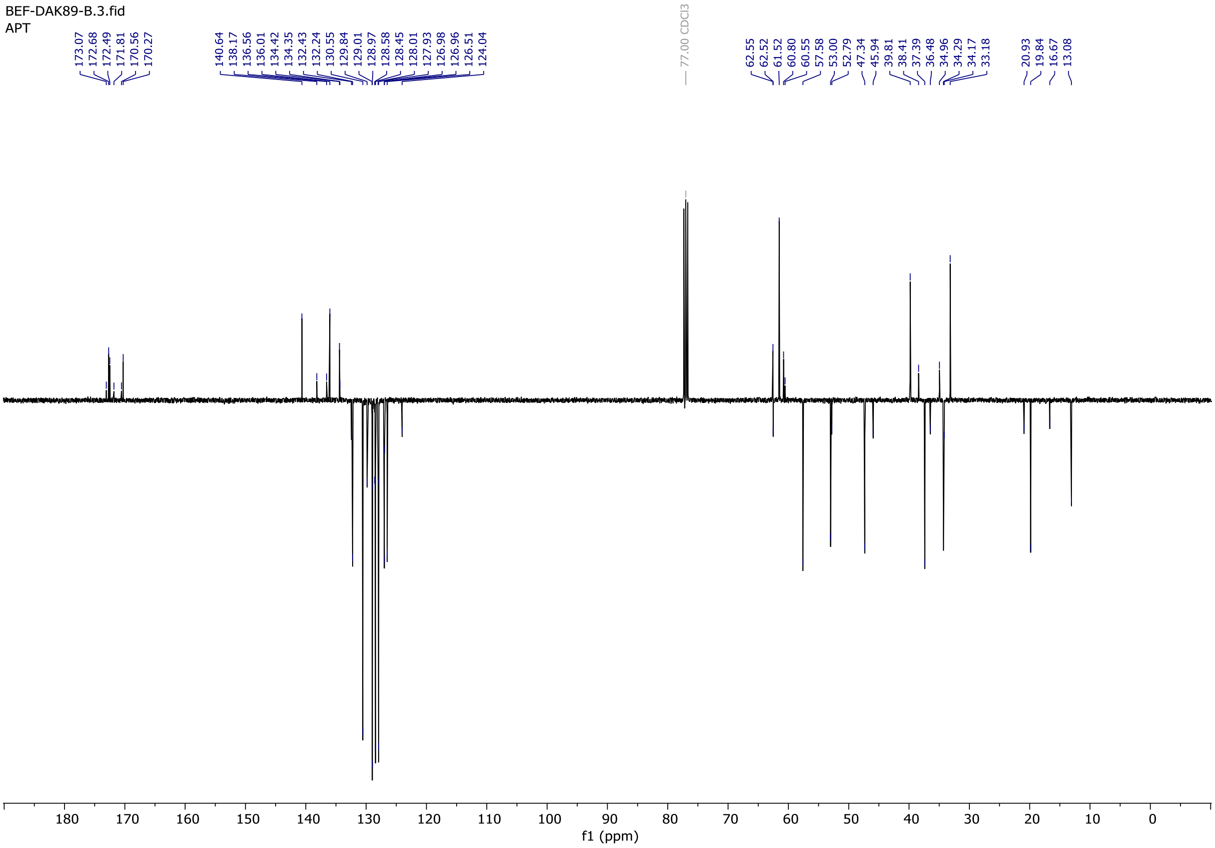
BEF-DAK89-B.3.fid
APT
173.07 172.68 172.49 171.81 170.56 170.27 140.64 138.17 136.56 136.01 134.42 134.35 132.43 132.24 130.55 129.84 129.01 128.97 128.58 128.45 128.01 127.93 126.98 126.96 126.51 124.04 77.00 CDCl3 62.55 62.52 61.52 60.80 60.55 57.58 53.00 52.79 47.34 45.94 39.81 38.41 37.39 36.48 34.96 34.29 34.17 33.18 20.93 19.84 16.67 13.08
180 170 160 150 140 130 120 110 100 90 80 70 60 50 40 30 20 10 0
f1 (ppm)

14

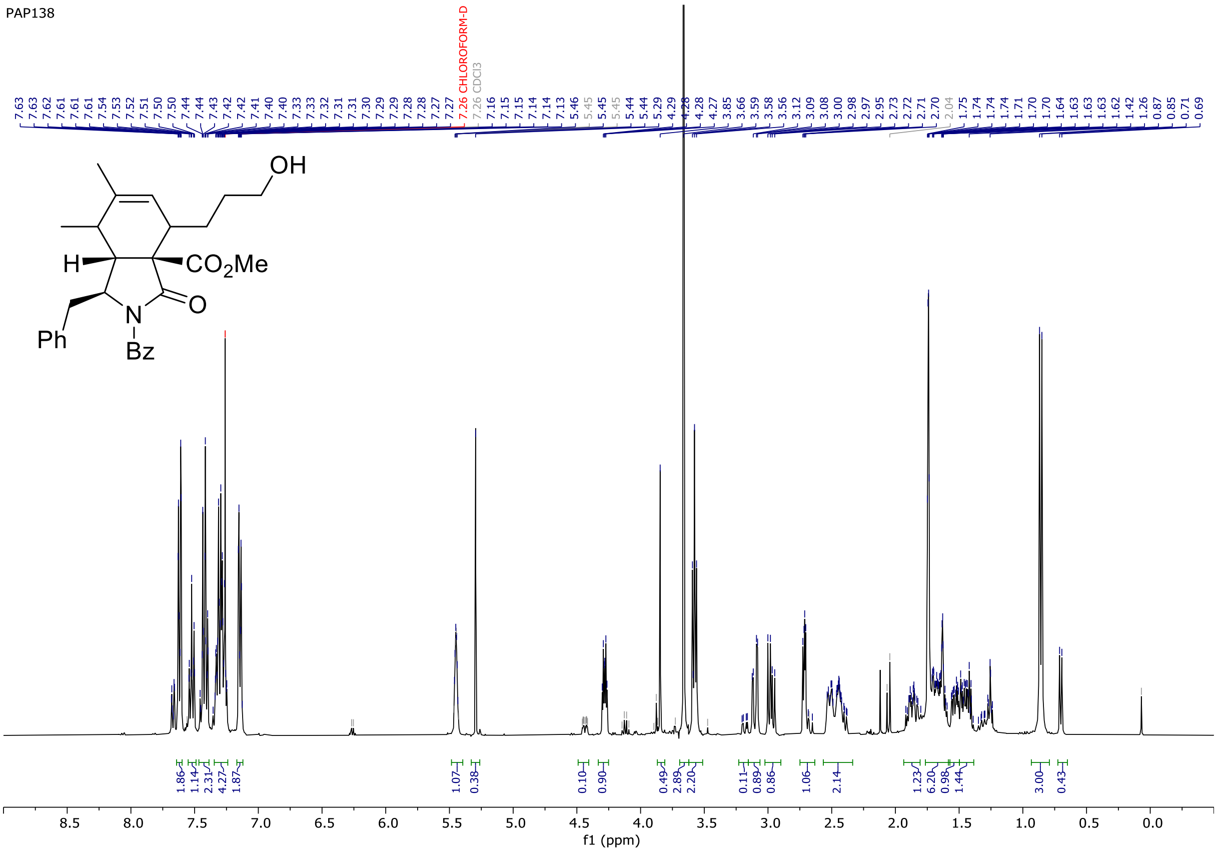
PAP138
7.63 7.63 7.62 7.61 7.61 7.61 7.54 7.53 7.52 7.51 7.50 7.50 7.44 7.44 7.43 7.42 7.42 7.41 7.40 7.40 7.33 7.33 7.32 7.31 7.31 7.30 7.29 7.29 7.28 7.28 7.27 7.27
7.26 CHLOROFORM-D
7.26 CDCl3
7.16 7.15 7.15 7.14 7.14 7.13 5.46 5.45 5.45 5.44 5.44 5.29 4.29 4.28 4.28 4.27 3.85 3.66 3.59 3.58 3.56 3.12 3.09 3.08 3.00 2.98 2.97 2.95 2.73 2.72 2.71 2.70 2.04 1.75 1.74 1.74 1.74 1.71 1.70 1.64 1.63 1.63 1.63 1.62 1.42 1.26 0.87 0.85 0.71 0.69
OH
H
CO_2Me
O
N
Ph
Bz
1.86 1.14 2.31 4.27 1.87
1.07 0.38
0.10 0.90
0.49 2.89 2.20
0.11 0.89 0.86
1.06 2.14
1.23 6.20 0.98 1.44
3.00 0.43
8.5 8.0 7.5 7.0 6.5 6.0 5.5 5.0 4.5 4.0 3.5 3.0 2.5 2.0 1.5 1.0 0.5 0.0
f1 (ppm)

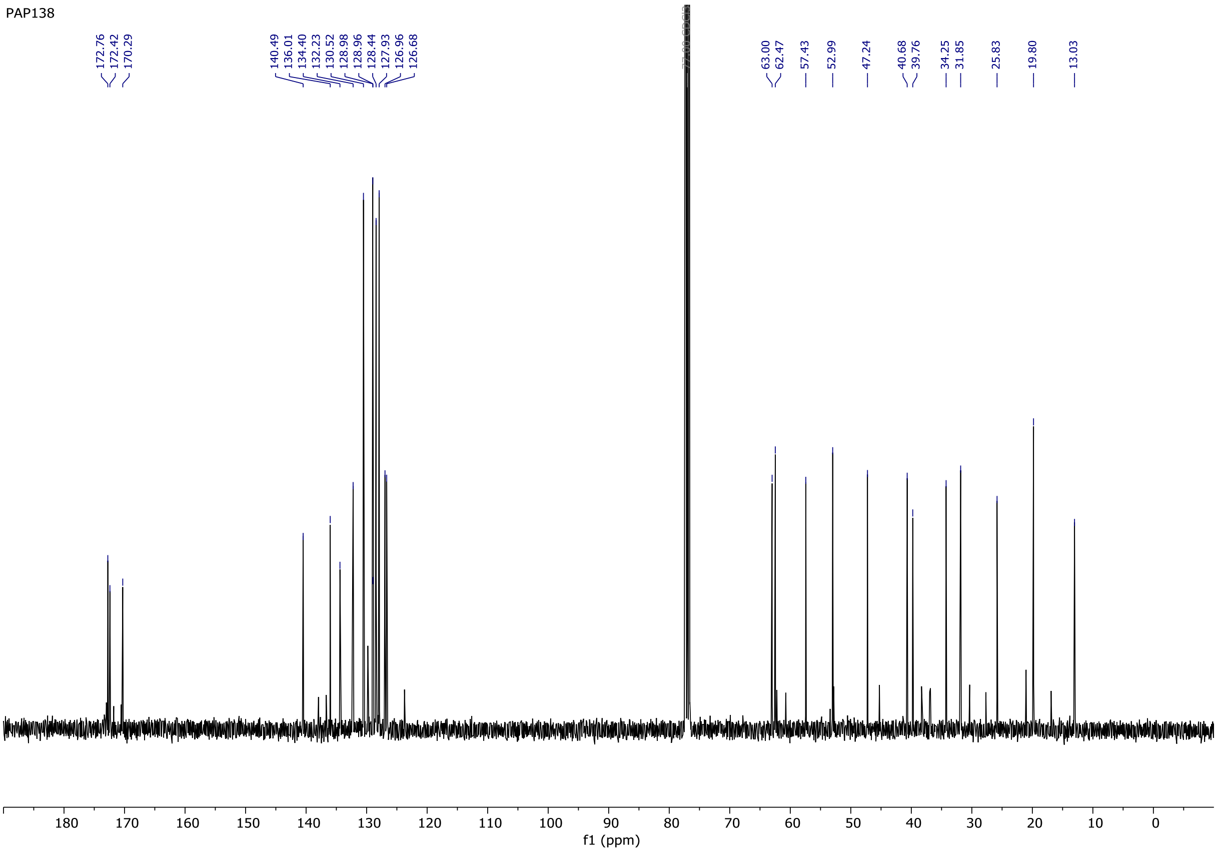
PAP138
172.76 172.42 170.29
140.49 136.01 134.40 132.23 130.52 128.98 128.96 128.44 127.93 126.96 126.68
63.00 62.47 57.43 52.99 47.24 40.68 39.76 34.25 31.85 25.83 19.80 13.03
180 170 160 150 140 130 120 110 100 90 80 70 60 50 40 30 20 10 0
f1 (ppm)

16a

BF-21-177_ac3-hlF

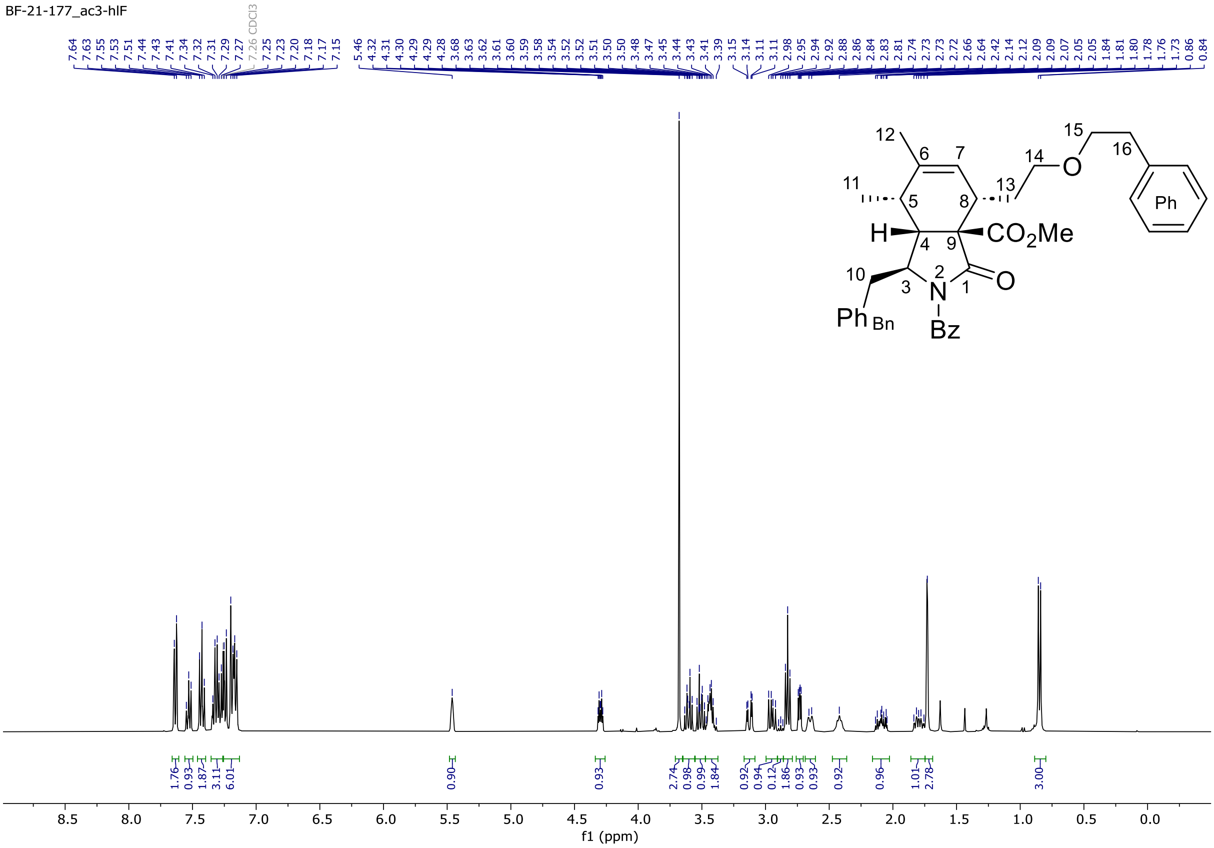

APT_01

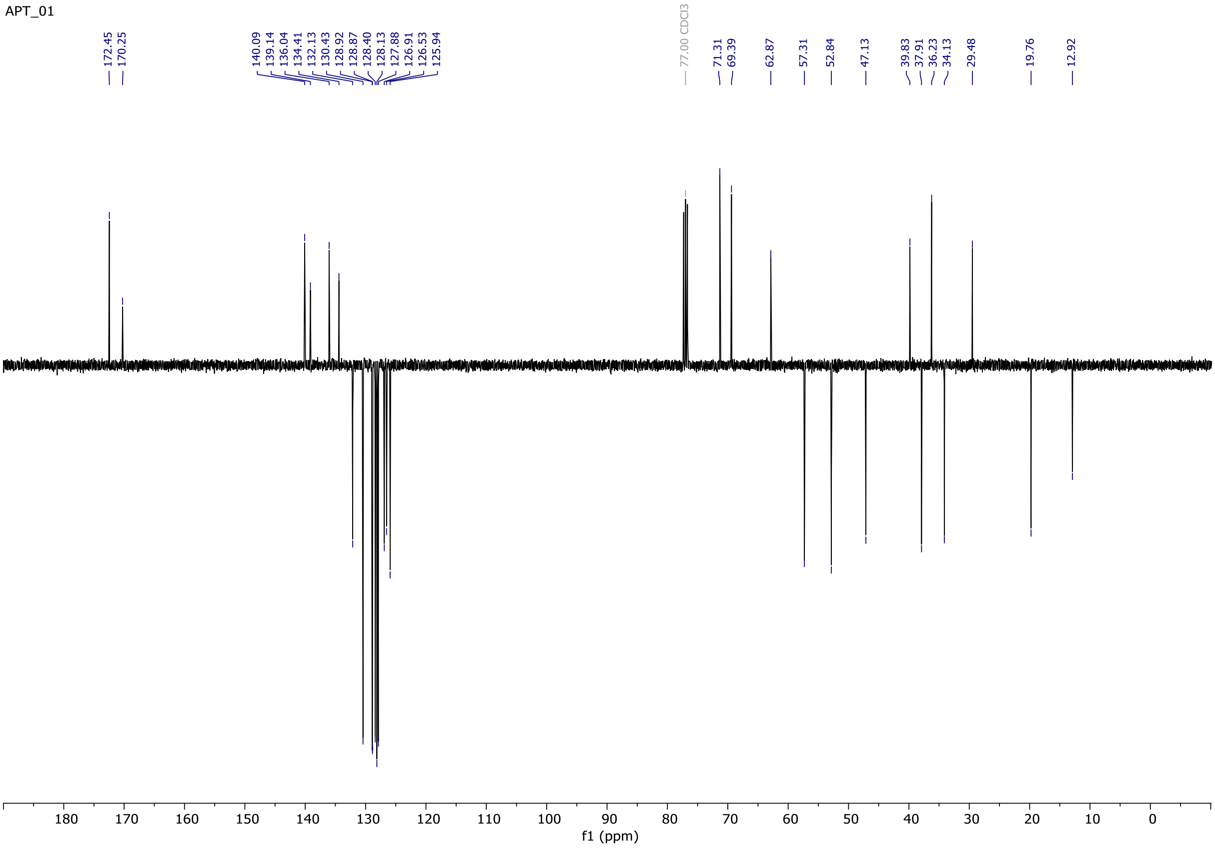

16b

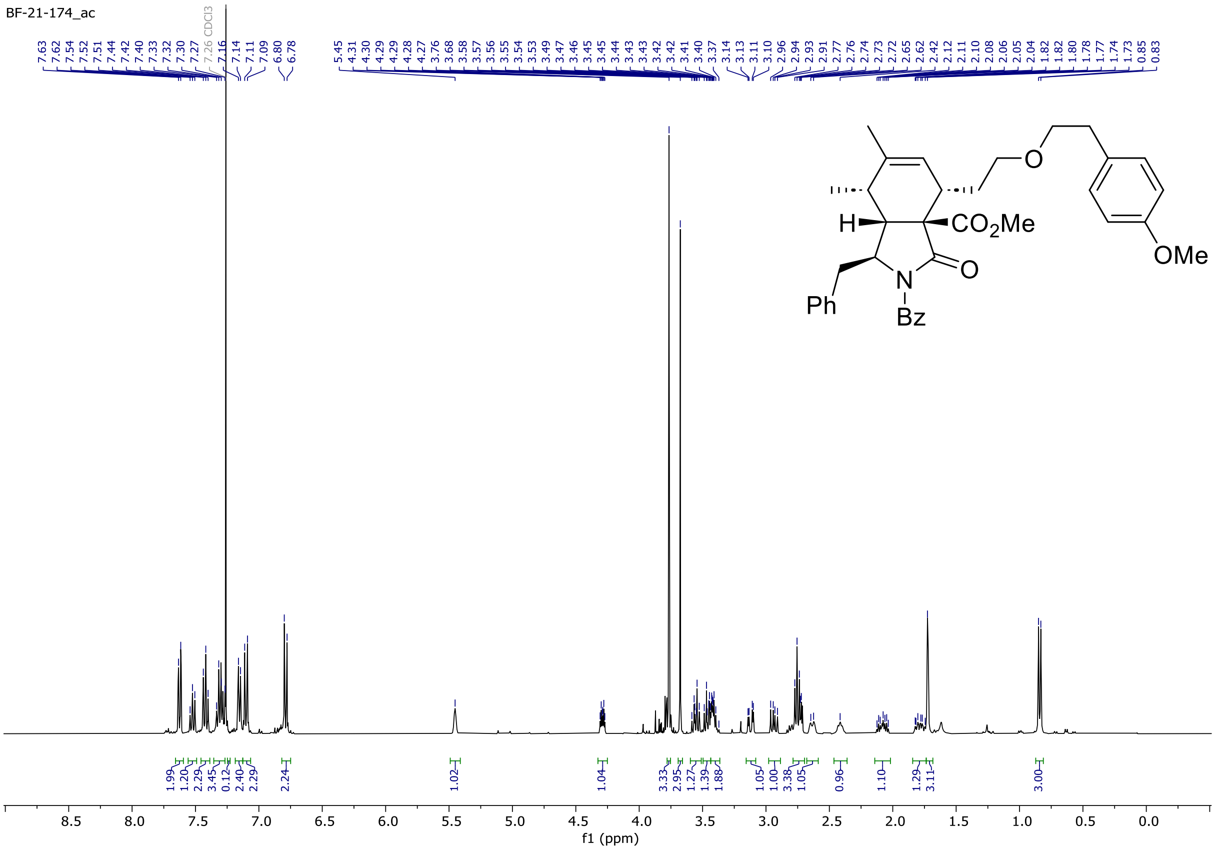

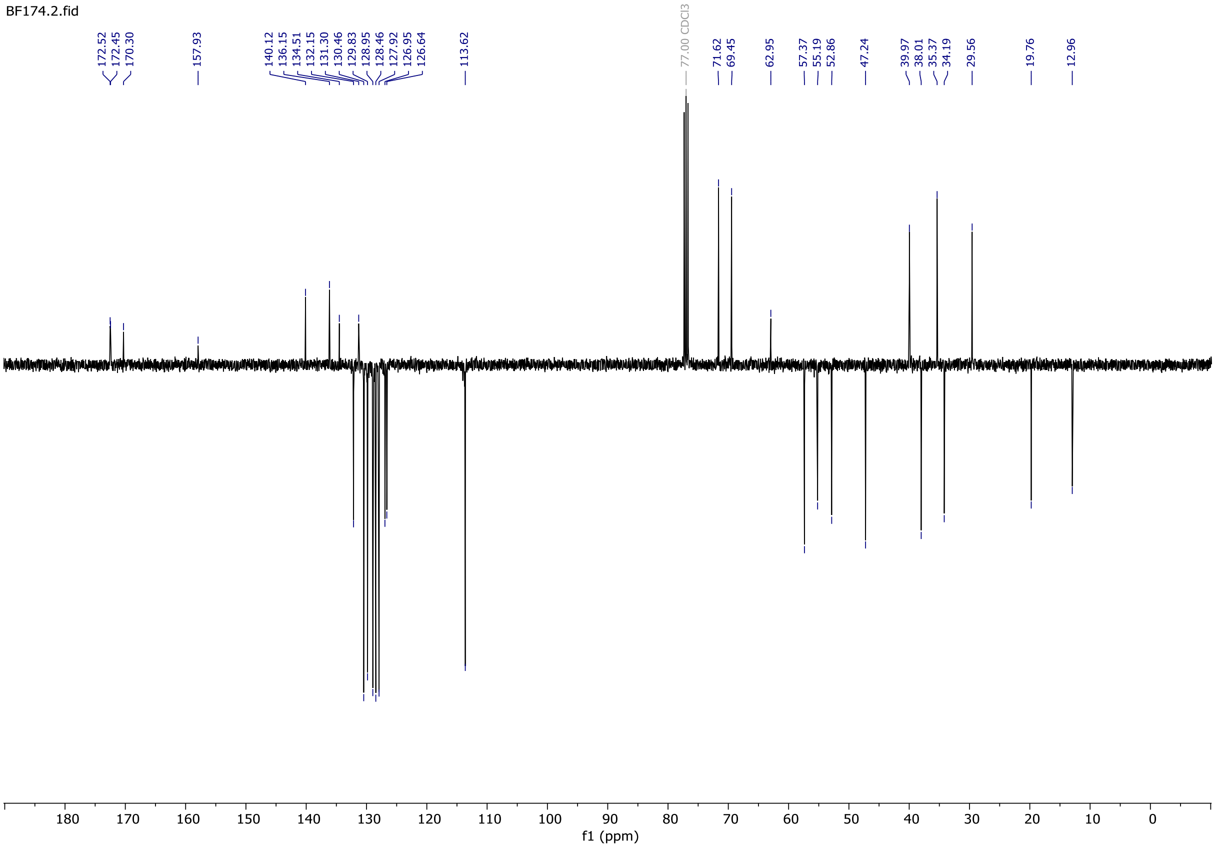

16c

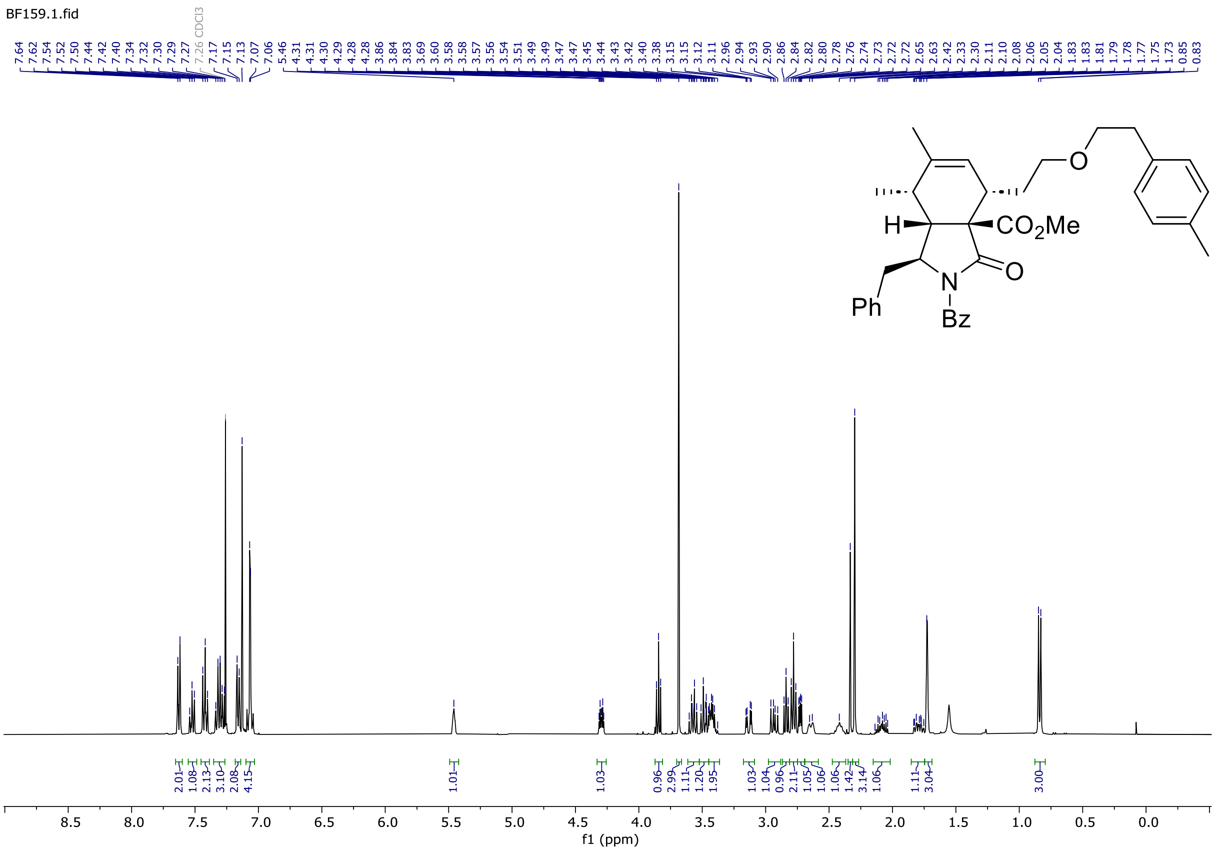
BF159.1.fid
7.64 7.62 7.54 7.52 7.50 7.44 7.42 7.40 7.34 7.32 7.30 7.29 7.27 7.26 CDCl3 7.17 7.15 7.13 7.07 7.06 5.46 4.31 4.31 4.30 4.29 4.28 4.28 3.86 3.84 3.83 3.69 3.60 3.58 3.58 3.57 3.56 3.54 3.51 3.49 3.49 3.47 3.45 3.44 3.43 3.42 3.40 3.38 3.15 3.12 3.11 2.96 2.94 2.93 2.90 2.86 2.84 2.82 2.80 2.78 2.76 2.74 2.73 2.72 2.72 2.65 2.63 2.42 2.33 2.30 2.11 2.10 2.08 2.06 2.05 2.04 1.83 1.83 1.81 1.79 1.78 1.77 1.75 1.73 0.85 0.83
H
CO_2Me
O
O
N
Ph
Bz
2.01 1.08 2.13 3.10 2.08 4.15 1.01 1.03 0.96 2.99 1.11 1.20 1.95 1.03 1.04 0.96 2.11 1.05 1.06 1.06 1.42 3.14 1.06 1.11 3.04 3.00
8.5 8.0 7.5 7.0 6.5 6.0 5.5 5.0 4.5 4.0 3.5 3.0 2.5 2.0 1.5 1.0 0.5 0.0
f1 (ppm)

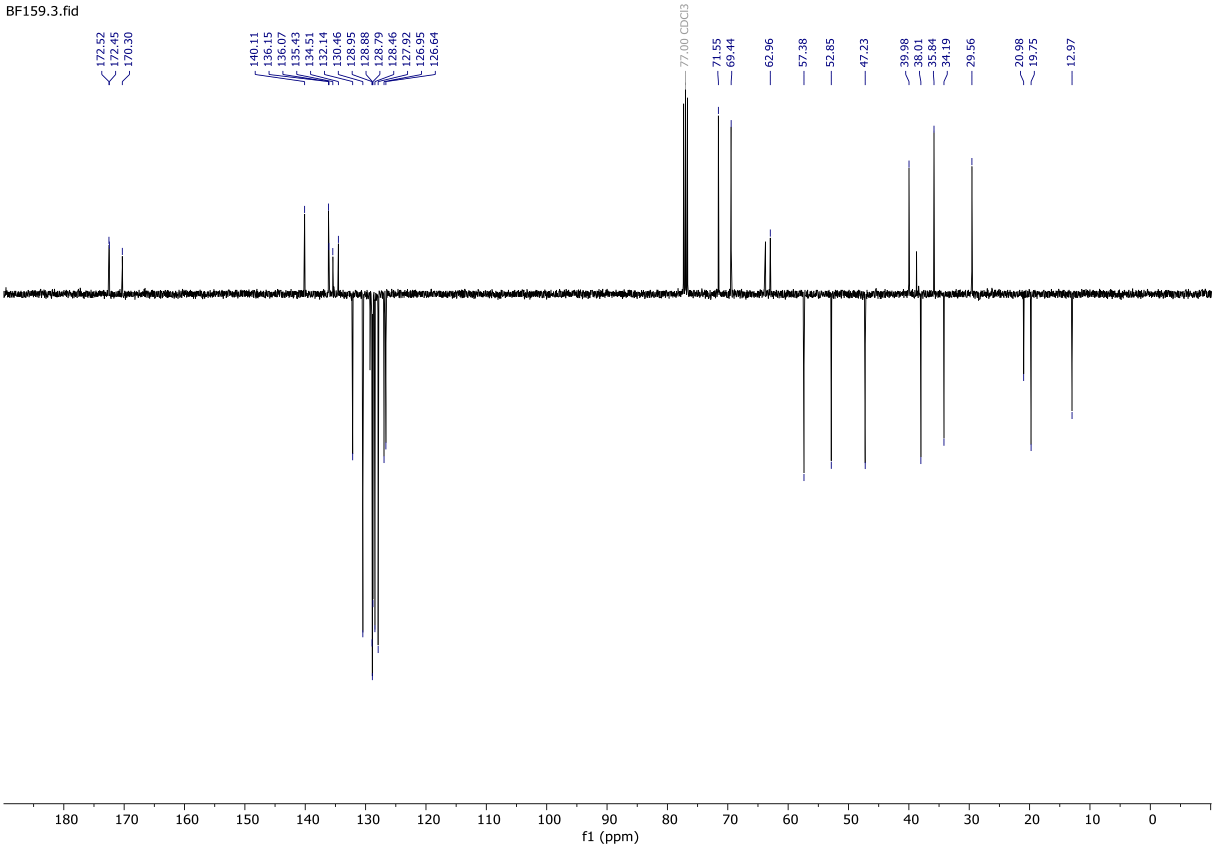
BF159.3.fid
172.52 172.45 170.30 140.11 136.15 136.07 135.43 134.51 132.14 130.46 128.95 128.88 128.79 128.46 127.92 126.95 126.64 77.00 CDCl3 71.55 69.44 62.96 57.38 52.85 47.23 39.98 38.01 35.84 34.19 29.56 20.98 19.75 12.97
180 170 160 150 140 130 120 110 100 90 80 70 60 50 40 30 20 10 0
f1 (ppm)

16d

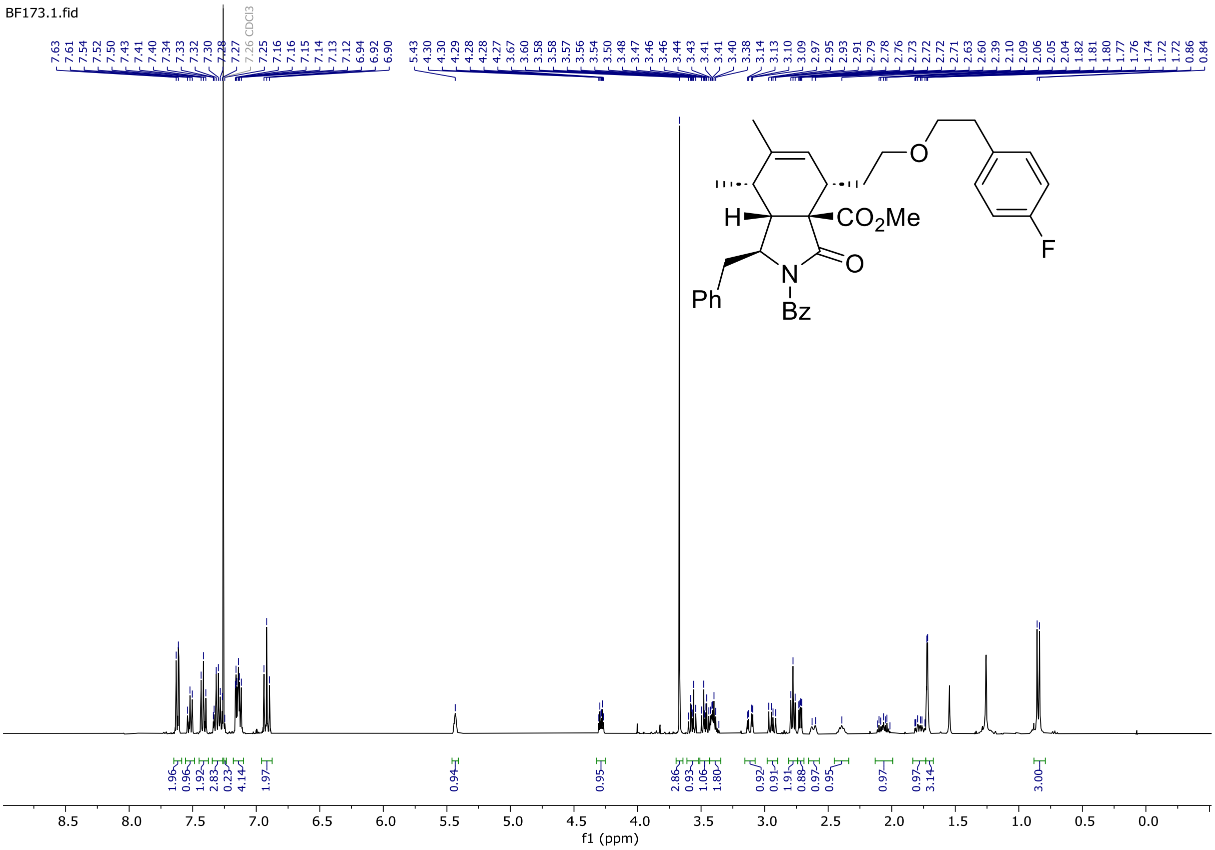

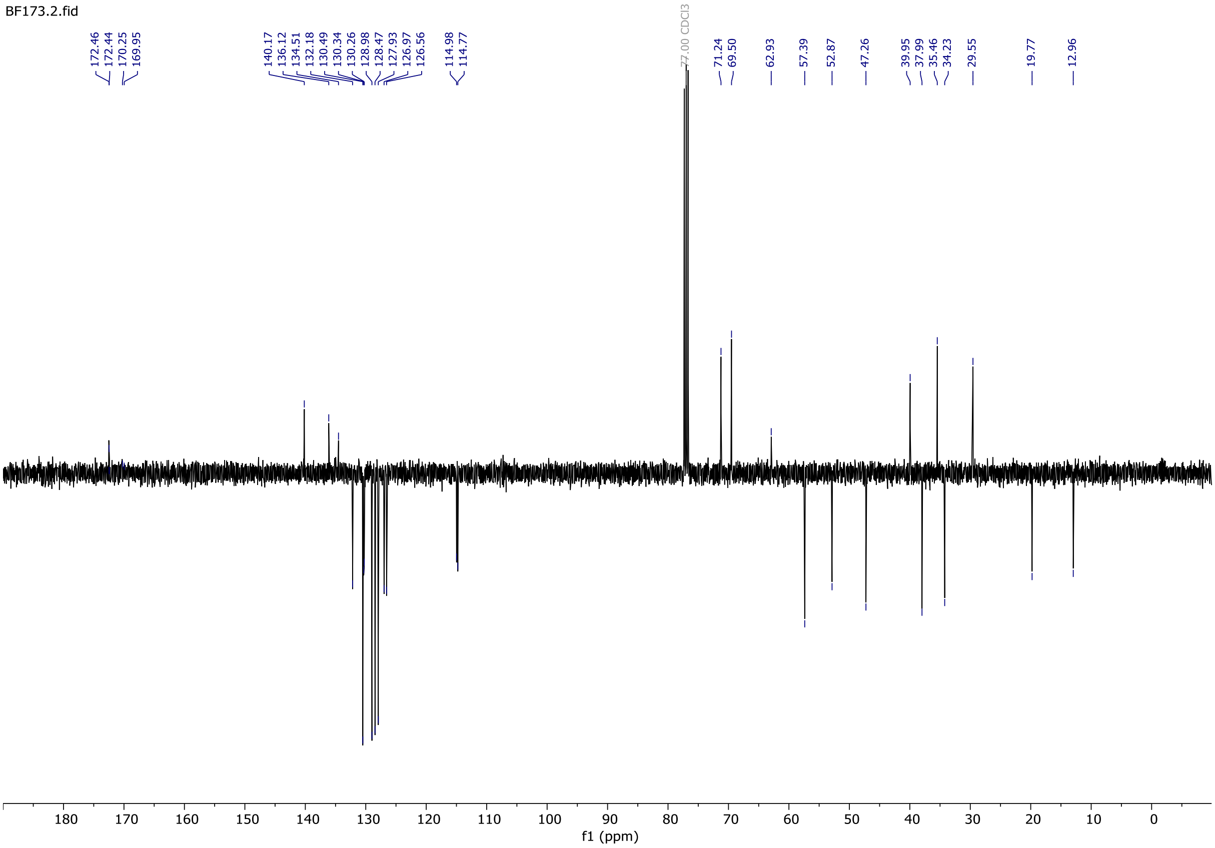

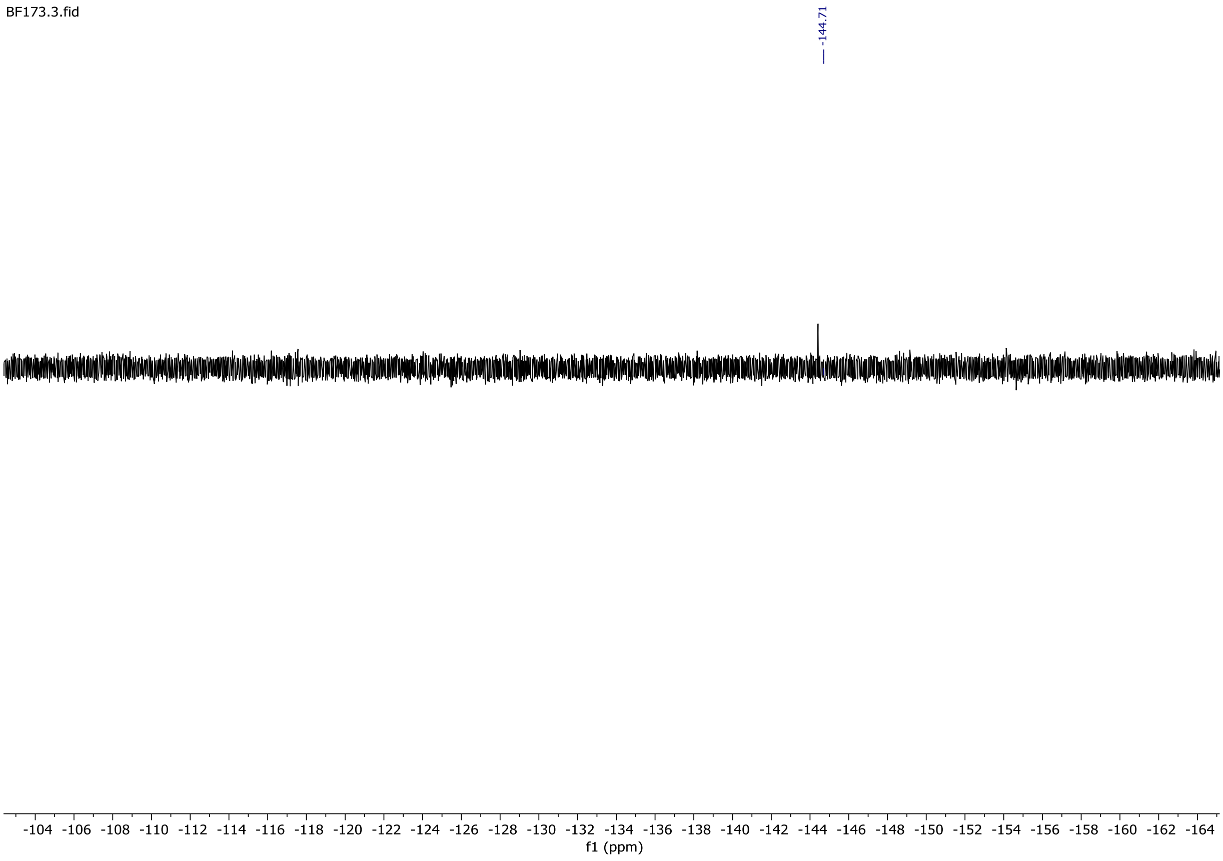
BF173.3.fid
-144.71
-104 -106 -108 -110 -112 -114 -116 -118 -120 -122 -124 -126 -128 -130 -132 -134 -136 -138 -140 -142 -144 -146 -148 -150 -152 -154 -156 -158 -160 -162 -164
f1 (ppm)

16e

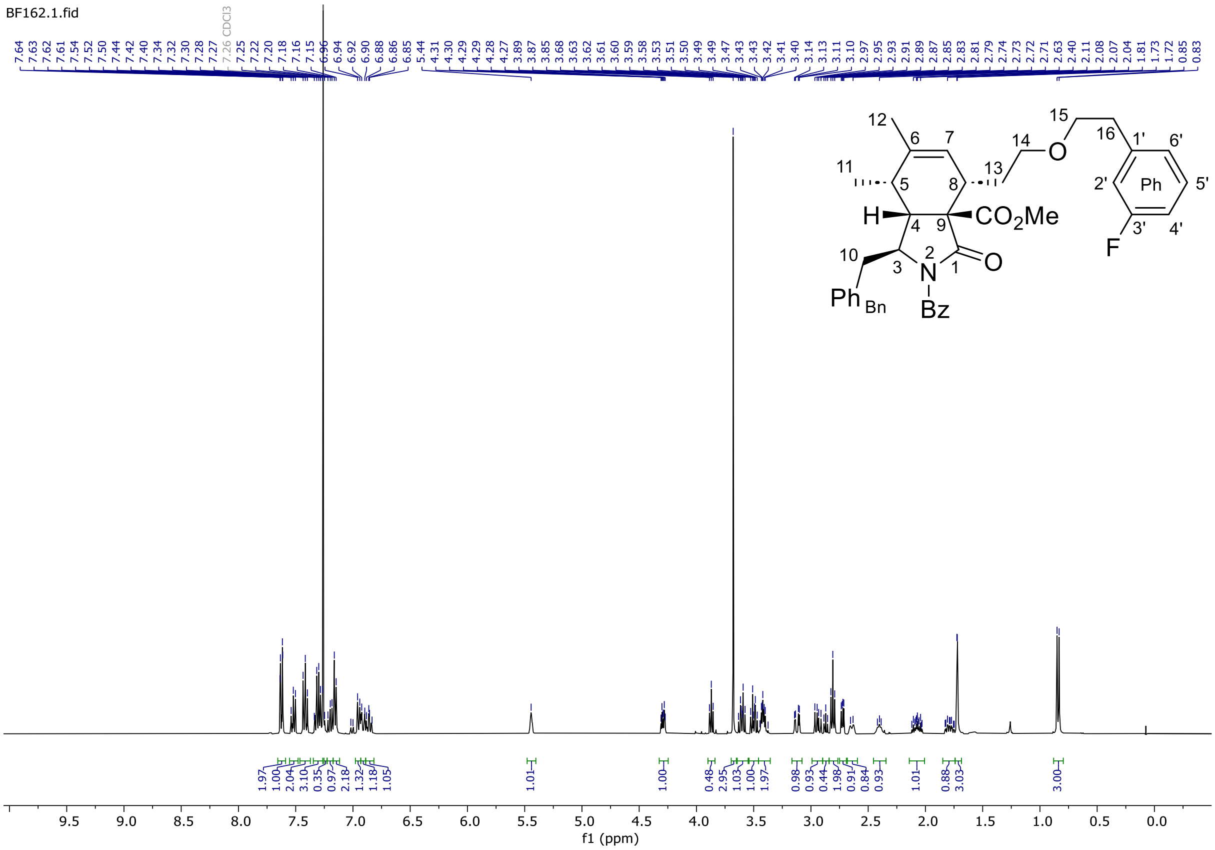

BF162.1.fid
7.64 7.63 7.62 7.61 7.54 7.52 7.50 7.44 7.42 7.40 7.34 7.32 7.30 7.28 7.27 7.26 CDCl3 7.25 7.22 7.20 7.18 7.16 7.15 6.96 6.94 6.92 6.90 6.88 6.86 6.85 5.44 4.31 4.30 4.29 4.29 4.28 4.27 3.89 3.87 3.85 3.68 3.63 3.62 3.61 3.60 3.59 3.58 3.53 3.51 3.50 3.49 3.49 3.47 3.43 3.43 3.42 3.41 3.40 3.14 3.13 3.11 3.10 2.97 2.95 2.93 2.91 2.89 2.87 2.85 2.83 2.81 2.79 2.74 2.73 2.72 2.71 2.63 2.40 2.11 2.08 2.07 2.04 1.81 1.73 1.72 0.85 0.83
1.97 1.00 2.04 3.10 0.35 0.97 2.18 1.32 1.18 1.05 1.01 1.00 0.48 2.95 1.03 1.00 1.97 0.98 0.93 0.44 1.98 0.91 0.84 0.93 1.01 0.88 3.03 3.00
f1 (ppm)
12
6
7
11
5
8
13
14
O
15
16
1'
6'
2'
Ph
5'
3'
4'
F
H
4
9
CO2Me
10
3
2
N
1
O
Ph Bn
Bz

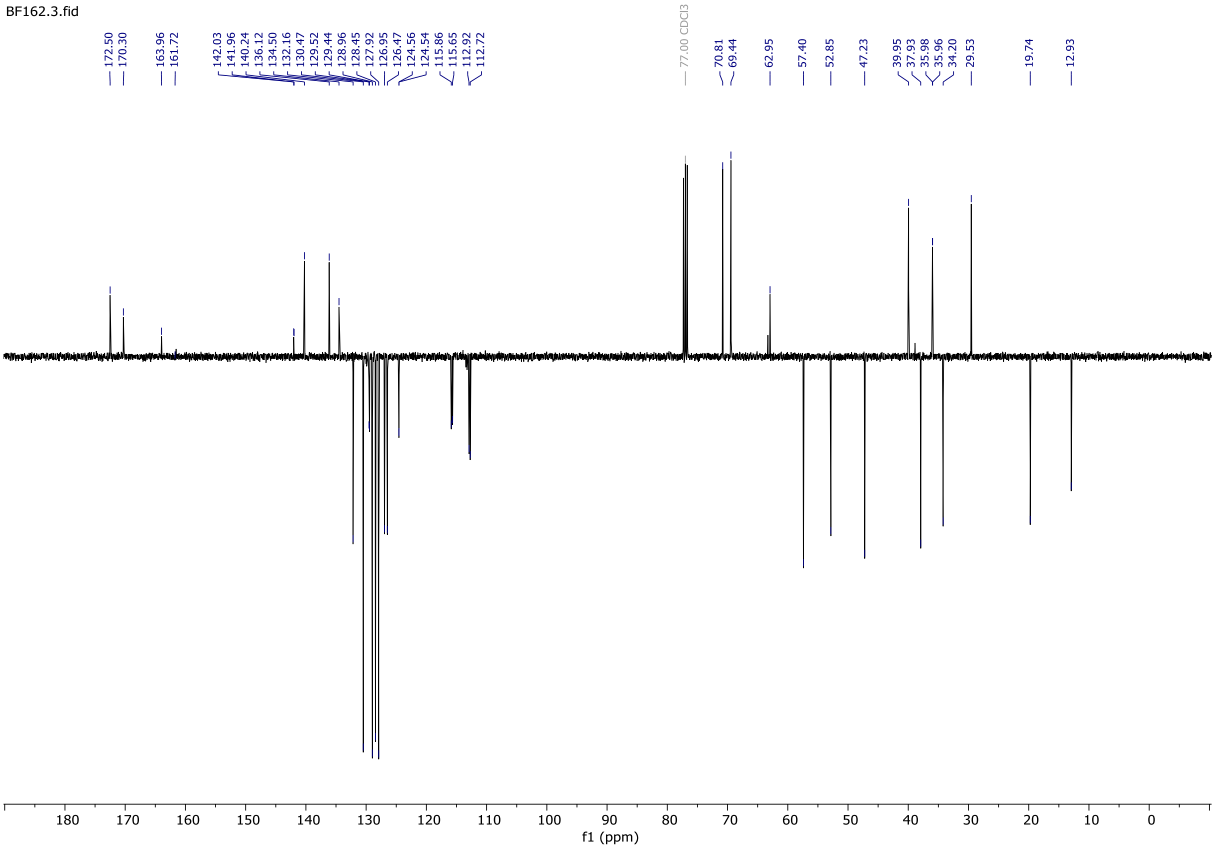

BF162.3.fid
172.50 170.30 163.96 161.72 142.03 141.96 140.24 136.12 134.50 132.16 130.47 129.52 129.44 128.96 128.45 127.92 126.95 126.47 124.56 124.54 115.86 115.65 112.92 112.72 77.00 CDCl3 70.81 69.44 62.95 57.40 52.85 47.23 39.95 37.93 35.98 35.96 34.20 29.53 19.74 12.93
f1 (ppm)

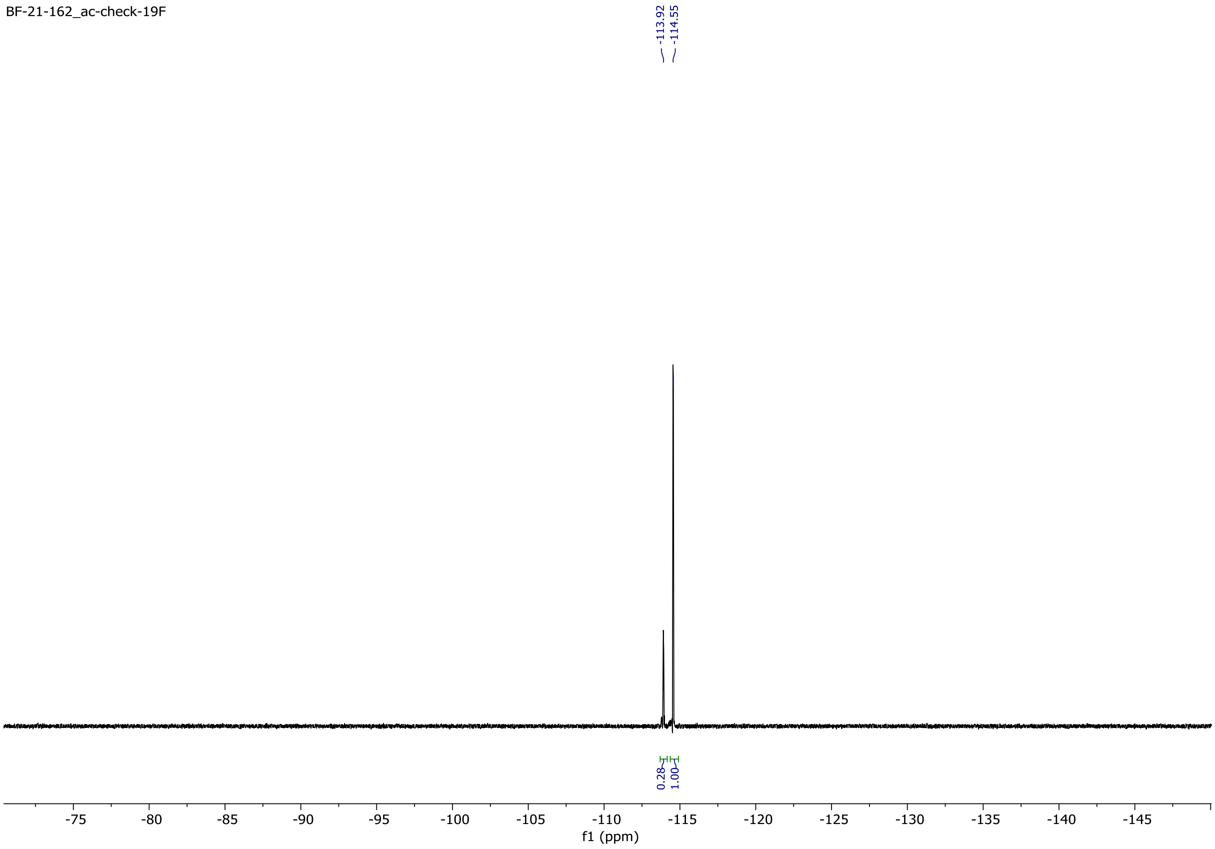

BF-21-162_ac-check-19F
-113.92
-114.55
0.28
1.00
-75
-80
-85
-90
-95
-100
-105
-110
-115
-120
-125
-130
-135
-140
-145
f1 (ppm)

16f

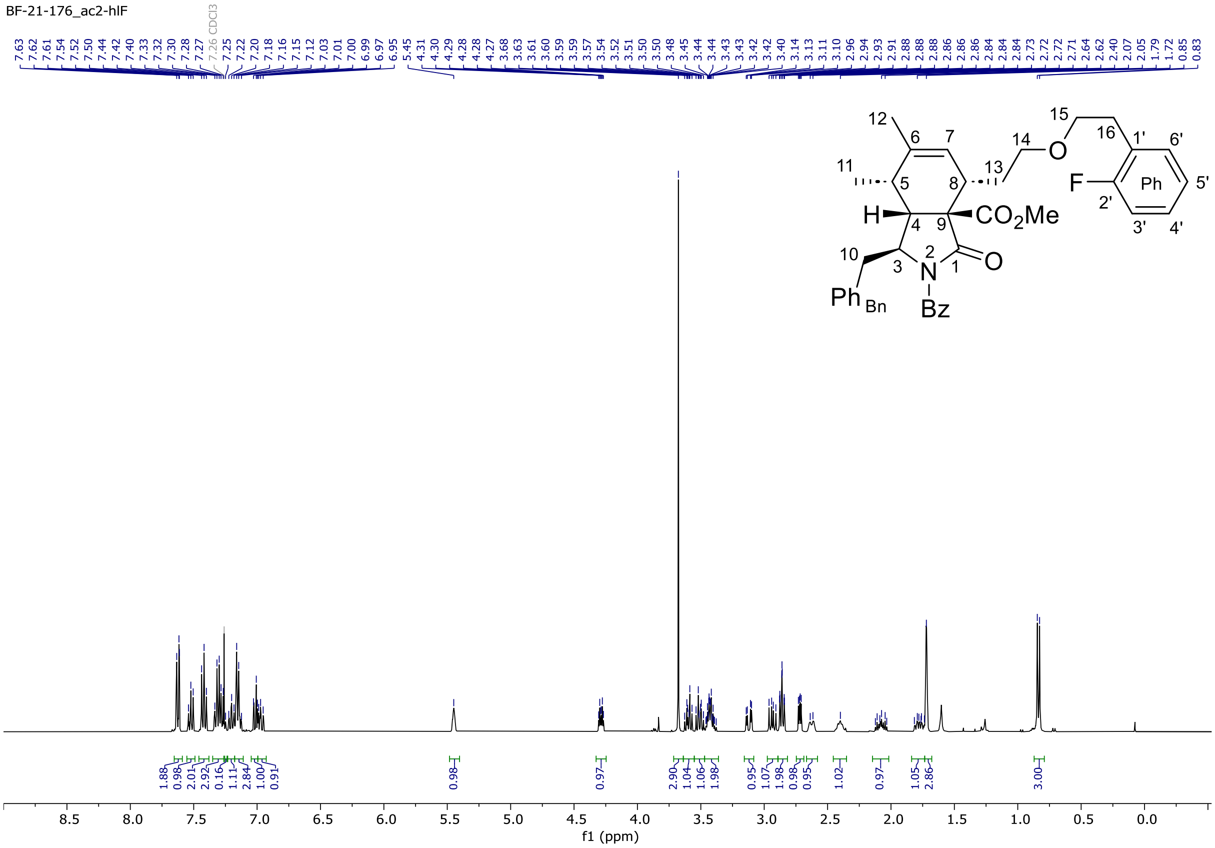
BF-21-176_ac2-hlF
7.63 7.62 7.61 7.54 7.52 7.50 7.44 7.42 7.40 7.33 7.32 7.30 7.28 7.27 7.26 CDCl3 7.25 7.22 7.20 7.18 7.16 7.15 7.12 7.03 7.01 7.00 6.99 6.97 6.95 5.45 4.31 4.30 4.29 4.28 4.28 4.27 3.68 3.63 3.61 3.60 3.59 3.59 3.57 3.54 3.52 3.51 3.50 3.48 3.45 3.44 3.44 3.43 3.43 3.42 3.42 3.40 3.14 3.13 3.11 3.10 2.96 2.94 2.93 2.91 2.88 2.88 2.86 2.86 2.86 2.84 2.84 2.84 2.73 2.72 2.72 2.71 2.64 2.62 2.40 2.07 2.05 1.79 1.72 0.85 0.83
1.88 0.98 2.01 2.92 0.16 1.11 2.84 1.00 0.91 0.98 0.97 2.90 1.04 1.06 1.98 0.95 1.07 1.98 0.98 0.95 1.02 0.97 1.05 2.86 3.00
8.5 8.0 7.5 7.0 6.5 6.0 5.5 5.0 4.5 4.0 3.5 3.0 2.5 2.0 1.5 1.0 0.5 0.0
f1 (ppm)
12 6 7 11 5 8 13 14 15 16 1' 6' H 4 9 CO2Me F Ph 5' 2' 3' 4' 10 3 2 1 O N Ph Bn Bz

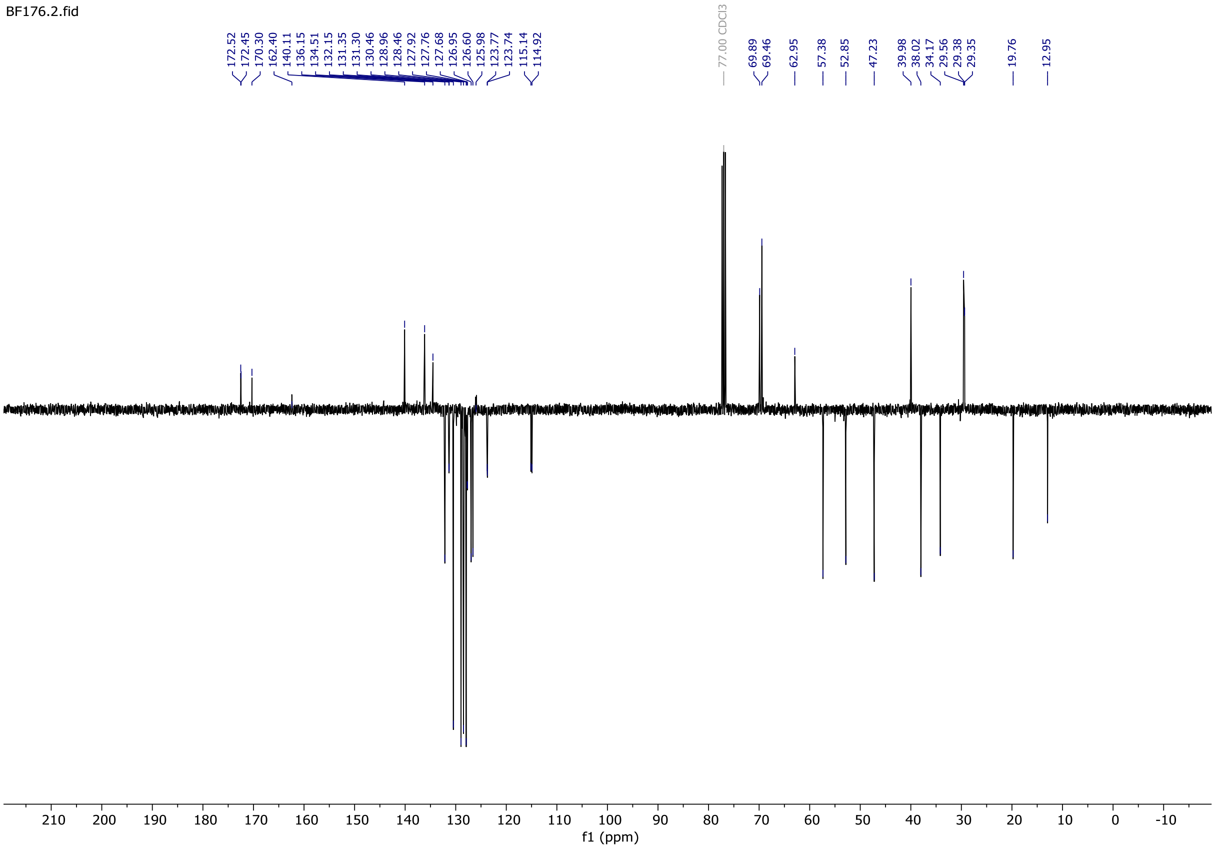
BF176.2.fid
172.52 172.45 170.30 162.40 140.11 136.15 134.51 132.15 131.35 131.30 130.46 128.96 128.46 127.92 127.76 127.68 126.95 126.60 125.98 123.77 123.74 115.14 114.92
77.00 CDCl3 69.89 69.46 62.95 57.38 52.85 47.23 39.98 38.02 34.17 29.56 29.38 29.35 19.76 12.95
210 200 190 180 170 160 150 140 130 120 110 100 90 80 70 60 50 40 30 20 10 0 -10
f1 (ppm)

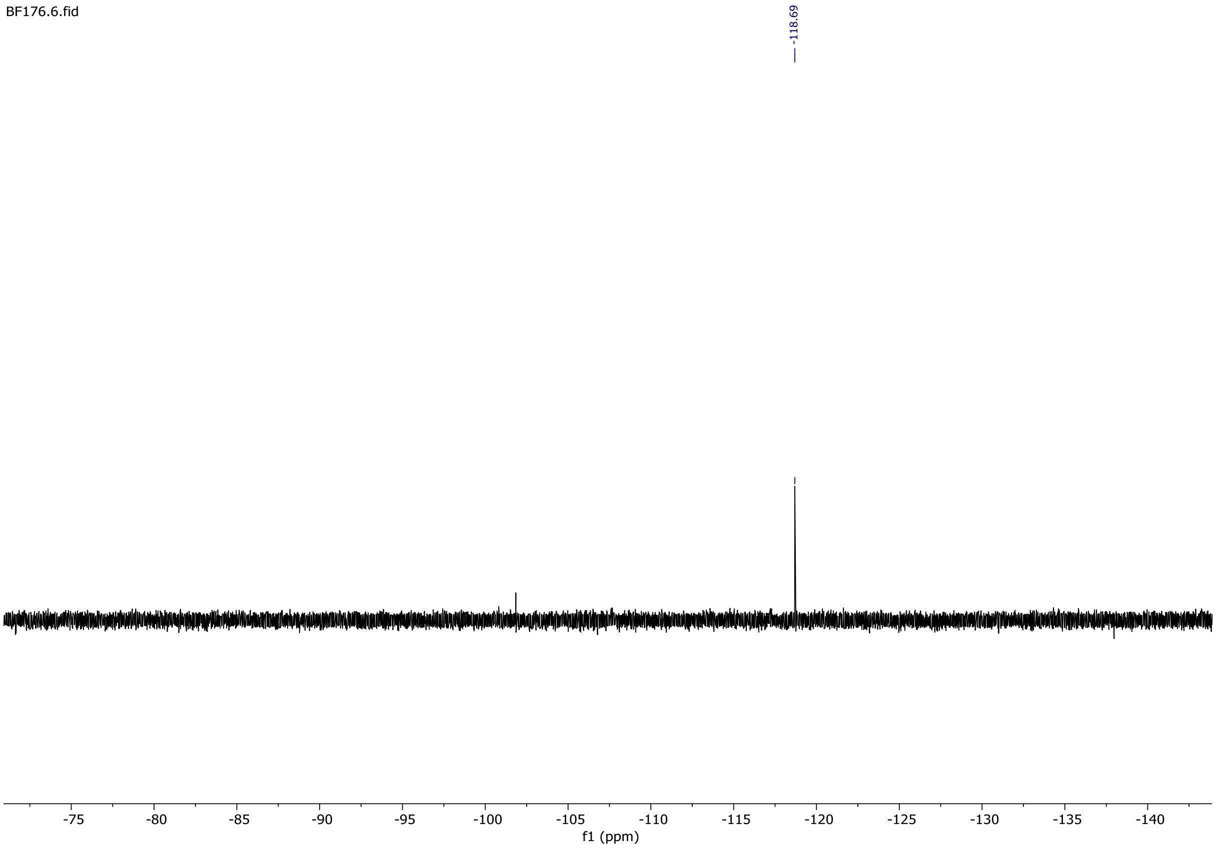
BF176.6.fid
-118.69
-75
-80
-85
-90
-95
-100
-105
-110
-115
-120
-125
-130
-135
-140
f1 (ppm)

16g

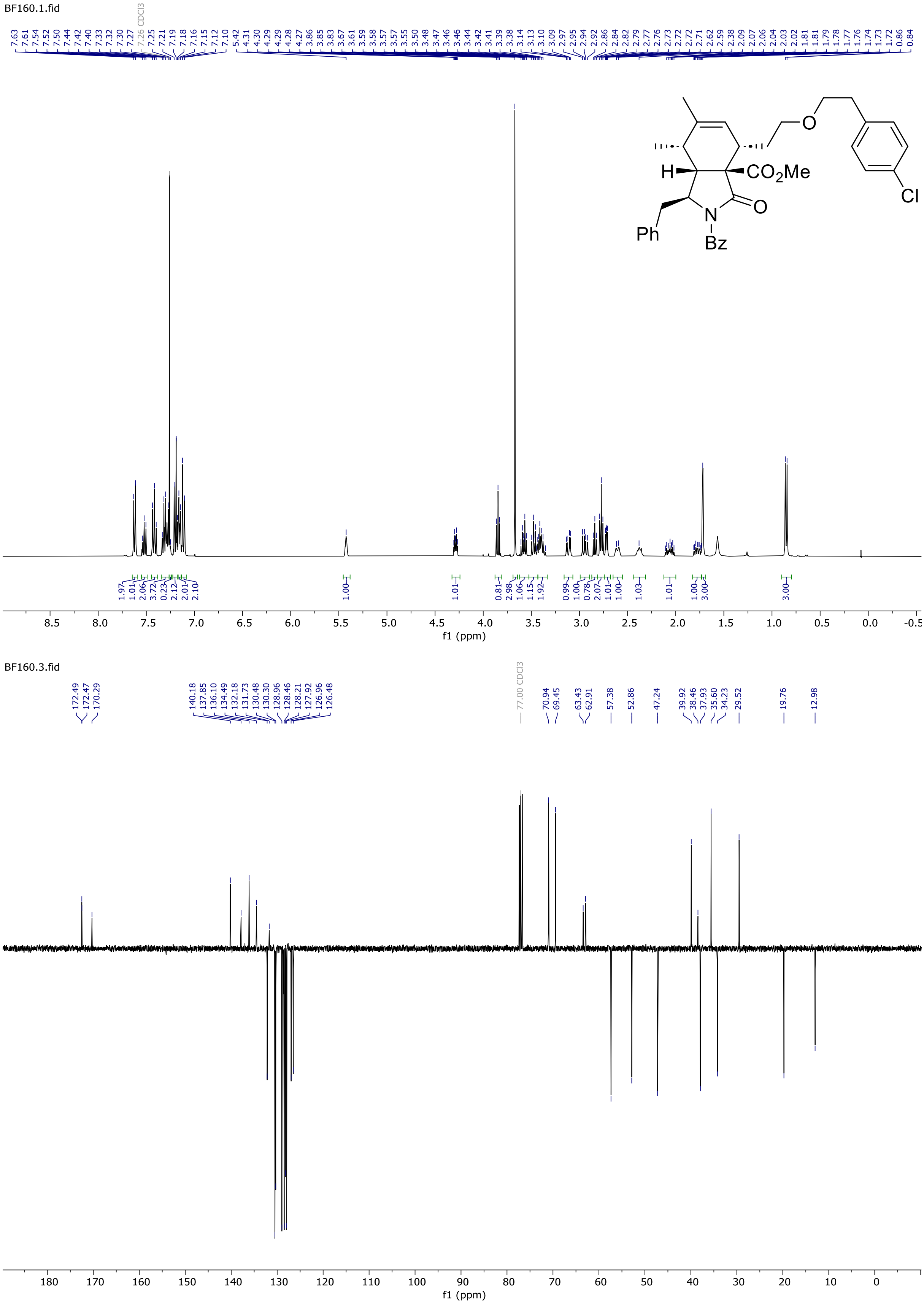

BF160.1.fid
7.63 7.61 7.54 7.52 7.50 7.44 7.42 7.40 7.33 7.32 7.30 7.27 7.26 CDCl3 7.25 7.21 7.19 7.18 7.16 7.15 7.12 7.10 5.42 4.31 4.30 4.29 4.29 4.28 4.27 3.86 3.85 3.83 3.67 3.61 3.59 3.58 3.57 3.55 3.50 3.48 3.47 3.46 3.46 3.44 3.42 3.41 3.39 3.38 3.14 3.13 3.10 3.09 2.97 2.95 2.94 2.92 2.86 2.84 2.82 2.79 2.77 2.76 2.73 2.72 2.72 2.71 2.62 2.59 2.38 2.09 2.07 2.06 2.04 2.03 2.02 1.81 1.79 1.78 1.77 1.76 1.74 1.73 1.72 0.86 0.84
H
CO2Me
O
Cl
Ph
N
Bz
1.97 1.01 2.06 3.72 0.23 2.12 2.01 2.10 1.00 1.01 0.81 2.98 1.06 1.15 1.92 0.99 1.00 0.78 2.07 1.01 1.00 1.03 1.01 1.00 3.00 3.00
f1 (ppm)
BF160.3.fid
172.49 172.47 170.29 140.18 137.85 136.10 134.49 132.18 131.73 130.48 130.30 128.96 128.46 128.21 127.92 126.96 126.48 77.00 CDCl3 70.94 69.45 63.43 62.91 57.38 52.86 47.24 39.92 38.46 37.93 35.60 34.23 29.52 19.76 12.98
f1 (ppm)

16h

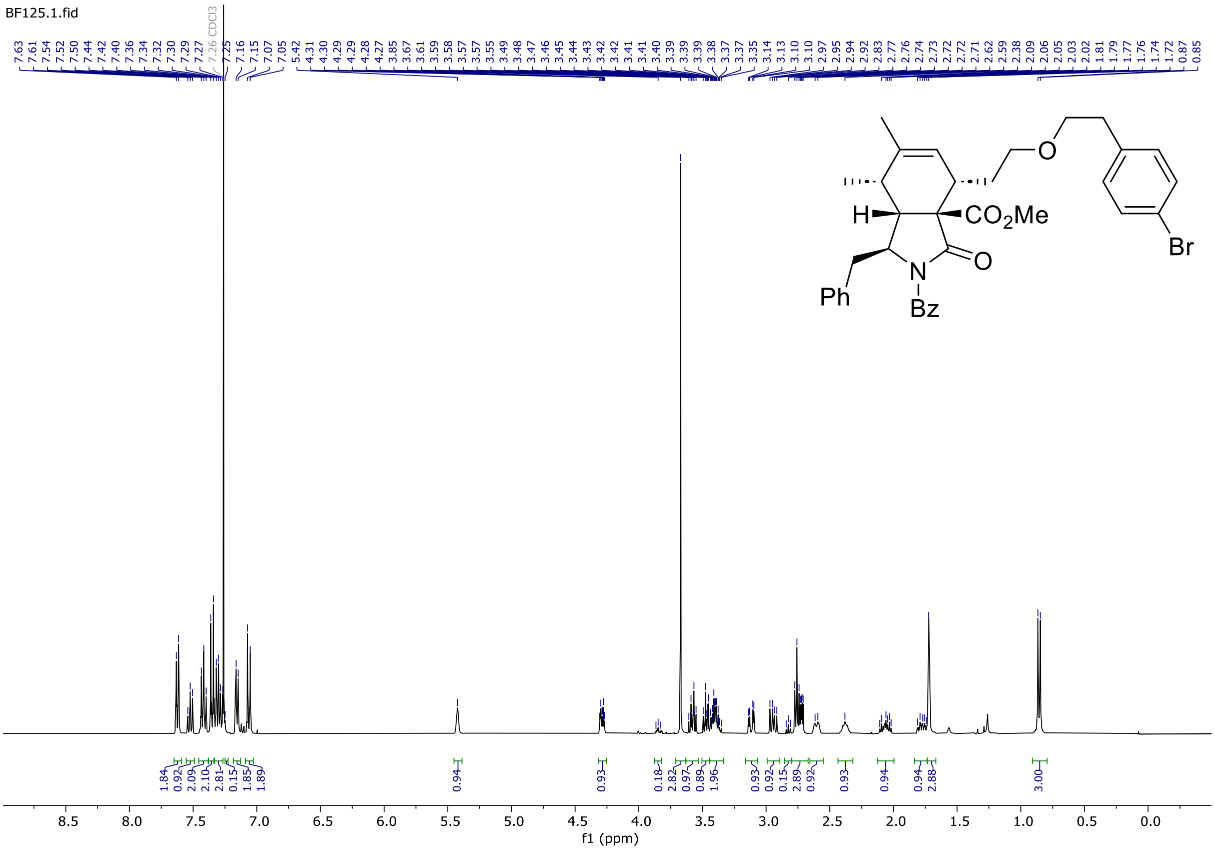
BF125.1.fid
7.63 7.61 7.54 7.52 7.50 7.44 7.42 7.40 7.36 7.34 7.32 7.30 7.29 7.27 7.26 CDCl3 7.25 7.16 7.15 7.07 7.05 5.42 4.31 4.30 4.29 4.29 4.28 4.27 3.85 3.67 3.61 3.59 3.58 3.57 3.57 3.55 3.49 3.48 3.47 3.46 3.45 3.44 3.43 3.42 3.42 3.41 3.40 3.39 3.39 3.39 3.38 3.37 3.37 3.35 3.14 3.13 3.10 3.10 2.97 2.95 2.94 2.92 2.83 2.77 2.76 2.74 2.73 2.72 2.72 2.71 2.62 2.59 2.38 2.09 2.06 2.05 2.03 2.02 1.81 1.79 1.77 1.76 1.74 1.72 0.87 0.85
H
CO_2Me
O
O
N
Ph
Bz
Br
1.84 0.92 2.09 2.10 2.81 0.15 1.85 1.89 0.94 0.93 0.18 2.82 0.97 0.89 1.96 0.93 0.92 0.15 2.89 0.92 0.93 0.94 0.94 2.88 3.00
8.5 8.0 7.5 7.0 6.5 6.0 5.5 5.0 4.5 4.0 3.5 3.0 2.5 2.0 1.5 1.0 0.5 0.0
f1 (ppm)

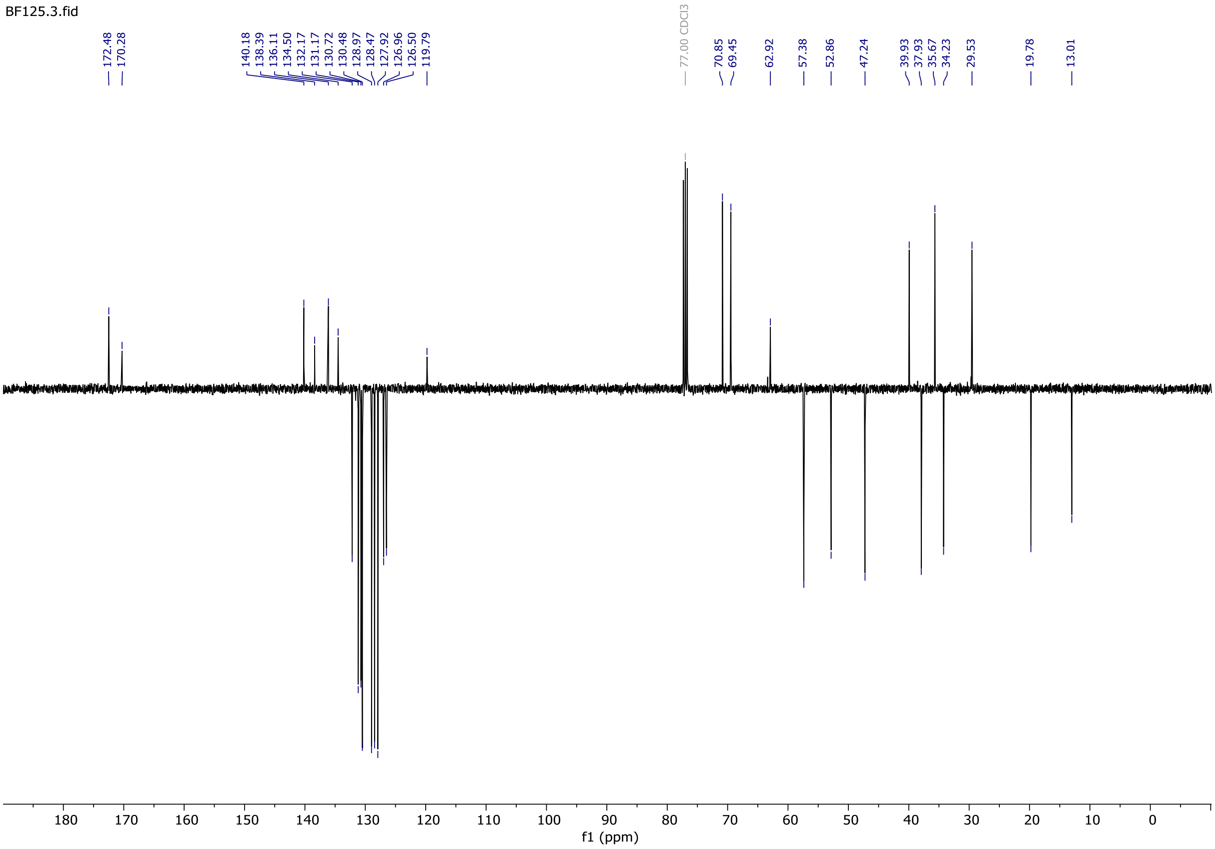
BF125.3.fid
172.48 170.28 140.18 138.39 136.11 134.50 132.17 131.17 130.72 130.48 128.97 128.47 127.92 126.96 126.50 119.79 77.00 CDCl3 70.85 69.45 62.92 57.38 52.86 47.24 39.93 37.93 35.67 34.23 29.53 19.78 13.01
180 170 160 150 140 130 120 110 100 90 80 70 60 50 40 30 20 10 0
f1 (ppm)

16i

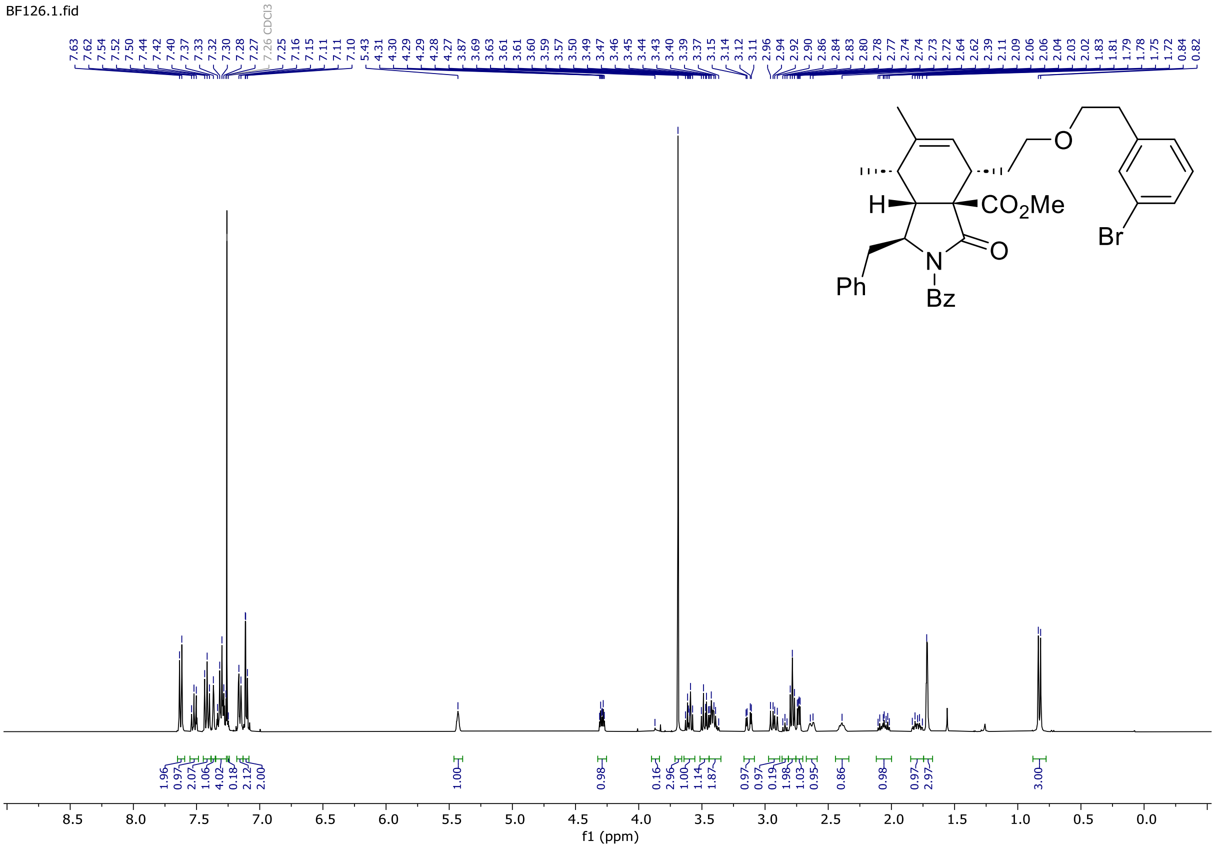
BF126.1.fid
7.63 7.62 7.54 7.52 7.50 7.44 7.42 7.40 7.37 7.33 7.32 7.30 7.28 7.27 7.26 CDCl3 7.25 7.16 7.15 7.11 7.11 7.10 5.43 4.31 4.30 4.29 4.29 4.28 4.27 3.87 3.69 3.63 3.61 3.61 3.60 3.59 3.57 3.50 3.49 3.47 3.46 3.45 3.44 3.43 3.40 3.39 3.37 3.15 3.14 3.12 3.11 2.96 2.94 2.92 2.90 2.86 2.84 2.83 2.80 2.78 2.77 2.74 2.74 2.73 2.72 2.64 2.62 2.39 2.11 2.09 2.06 2.04 2.03 2.02 1.83 1.81 1.79 1.78 1.75 1.72 0.84 0.82
H
CO2Me
O
Br
Ph
N
Bz
1.96 0.97 2.07 1.06 4.02 0.18 2.12 2.00 1.00 0.98 0.16 2.96 1.00 1.14 1.87 0.97 0.97 0.19 1.98 1.03 0.95 0.86 0.98 0.97 2.97 3.00
8.5 8.0 7.5 7.0 6.5 6.0 5.5 5.0 4.5 4.0 3.5 3.0 2.5 2.0 1.5 1.0 0.5 0.0
f1 (ppm)

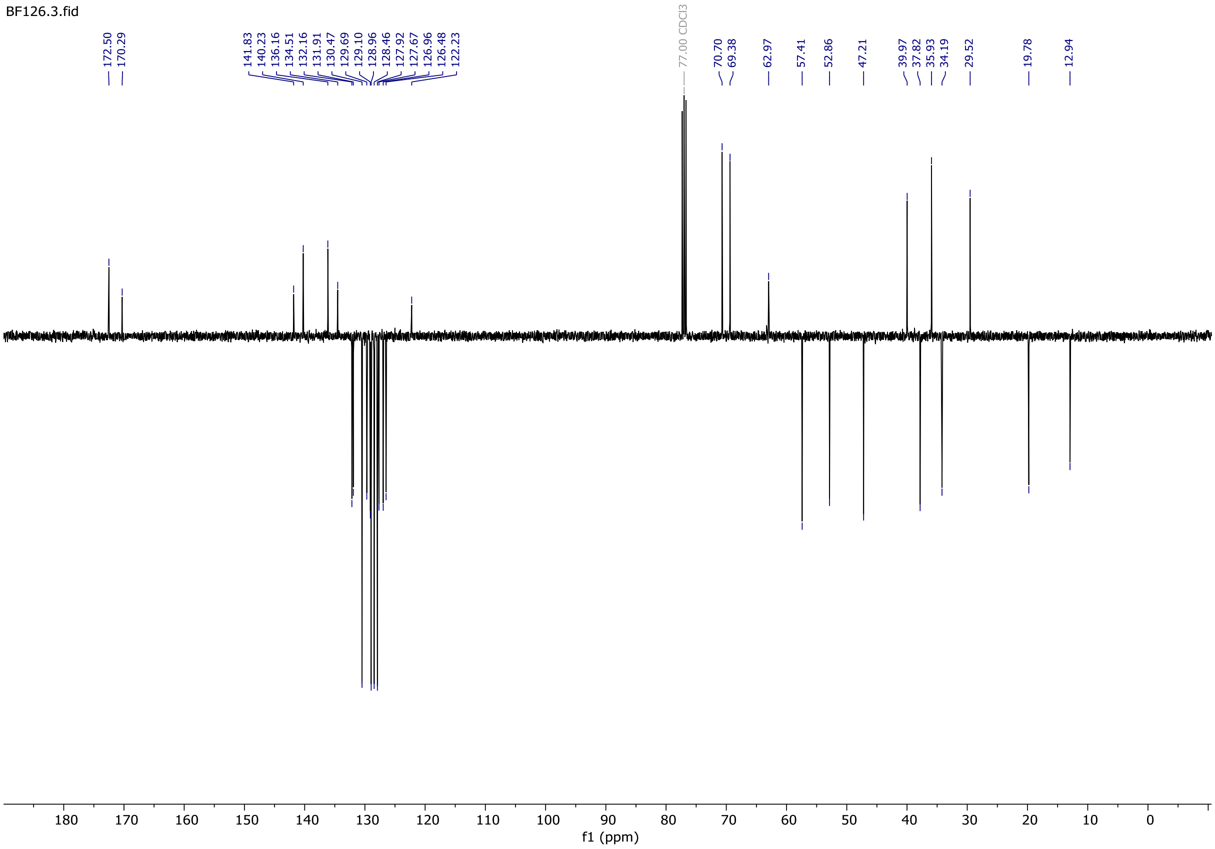
BF126.3.fid
172.50 170.29 141.83 140.23 136.16 134.51 132.16 131.91 130.47 129.69 129.10 128.96 128.46 127.92 127.67 126.96 126.48 122.23 77.00 CDCl3 70.70 69.38 62.97 57.41 52.86 47.21 39.97 37.82 35.93 34.19 29.52 19.78 12.94
180 170 160 150 140 130 120 110 100 90 80 70 60 50 40 30 20 10 0
f1 (ppm)

16j

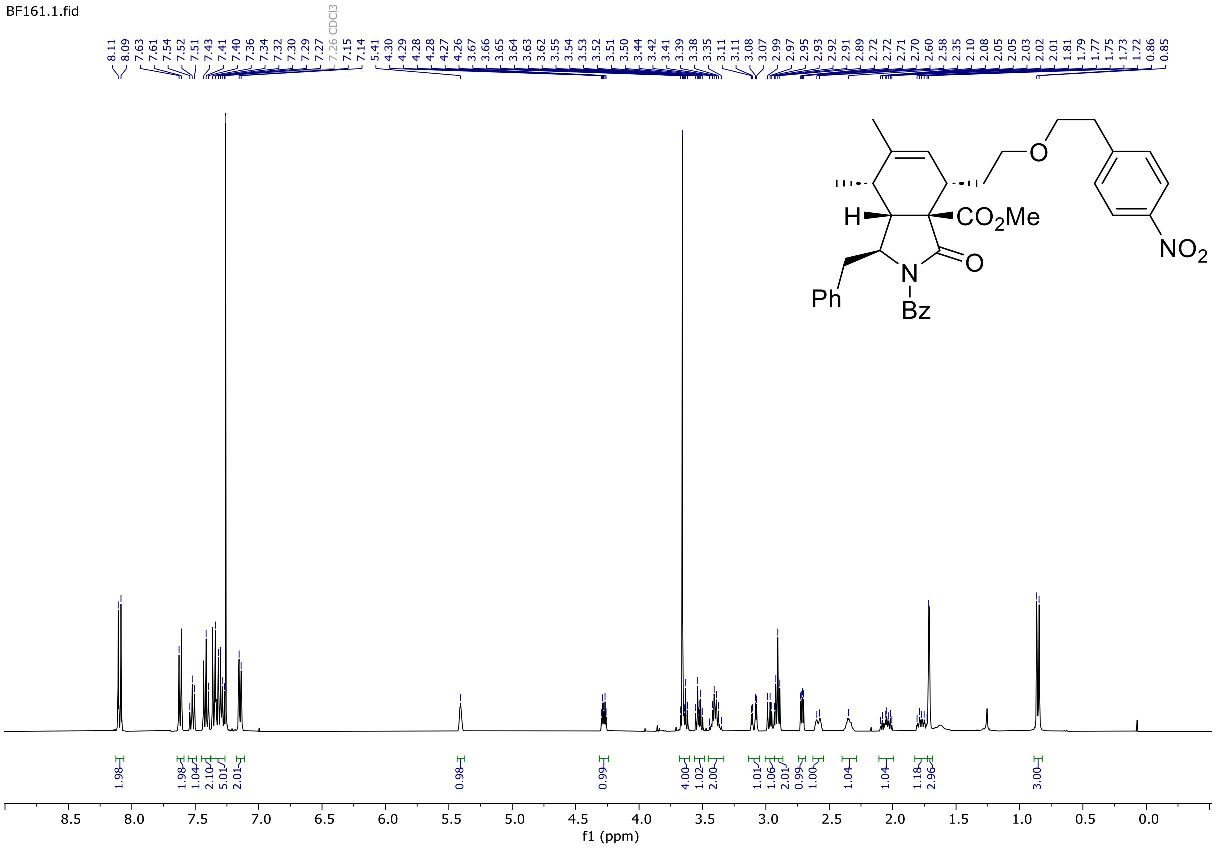
BF161.1.fid
8.11 8.09 7.63 7.61 7.54 7.52 7.51 7.43 7.41 7.40 7.36 7.34 7.32 7.30 7.29 7.27 7.26 CDCl3 7.15 7.14 5.41 4.30 4.29 4.28 4.28 4.27 4.26 3.67 3.66 3.65 3.64 3.63 3.62 3.55 3.54 3.54 3.53 3.52 3.51 3.50 3.44 3.42 3.41 3.39 3.38 3.35 3.11 3.11 3.08 3.07 2.99 2.97 2.95 2.93 2.92 2.91 2.89 2.72 2.72 2.71 2.70 2.60 2.58 2.35 2.10 2.08 2.05 2.05 2.03 2.02 2.01 1.81 1.79 1.77 1.75 1.73 1.72 0.86 0.85
CO2Me
O
NO2
H
O
N
Ph
Bz
1.98 1.98 1.04 2.10 5.01 2.01 0.98 0.99 4.00 1.02 2.00 1.01 1.06 2.01 0.99 1.00 1.04 1.04 1.18 2.96 3.00
8.5 8.0 7.5 7.0 6.5 6.0 5.5 5.0 4.5 4.0 3.5 3.0 2.5 2.0 1.5 1.0 0.5 0.0
f1 (ppm)

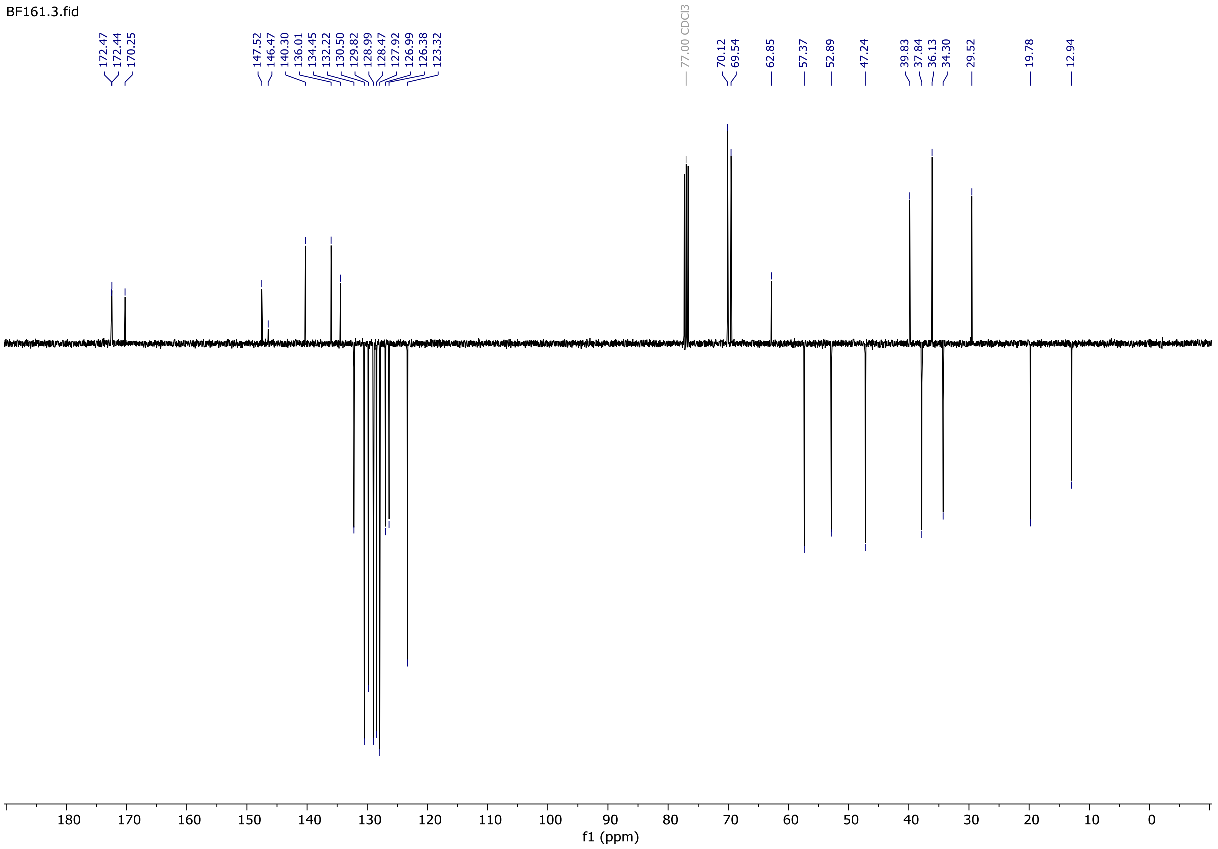
BF161.3.fid
172.47 172.44 170.25 147.52 146.47 140.30 136.01 134.45 132.22 130.50 129.82 128.99 128.47 127.92 126.99 126.38 123.32 77.00 CDCl3 70.12 69.54 62.85 57.37 52.89 47.24 39.83 37.84 36.13 34.30 29.52 19.78 12.94
180 170 160 150 140 130 120 110 100 90 80 70 60 50 40 30 20 10 0
f1 (ppm)

17a

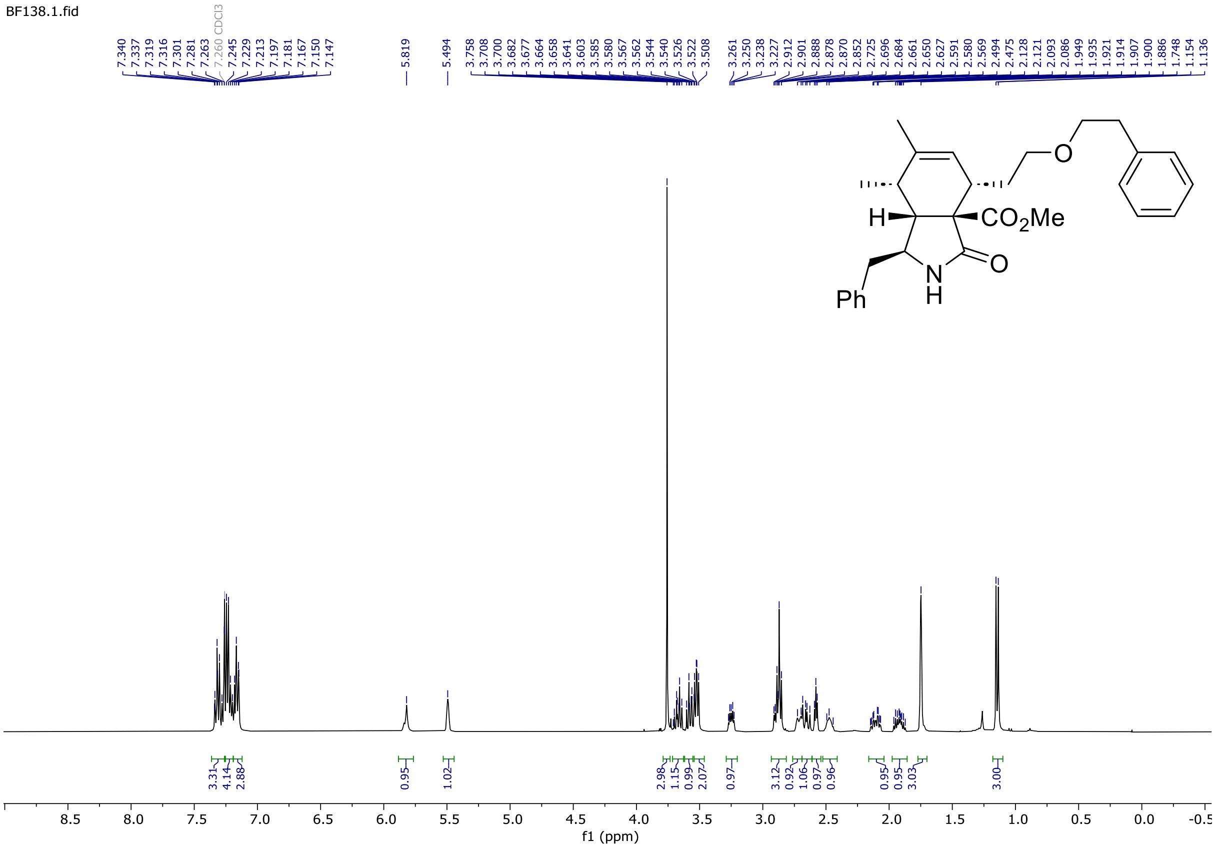
BF138.1.fid
7.340
7.337
7.319
7.316
7.301
7.281
7.263
7.260 CDCl3
7.245
7.229
7.213
7.197
7.181
7.167
7.150
7.147
5.819
5.494
3.758
3.708
3.700
3.682
3.677
3.664
3.658
3.641
3.603
3.585
3.580
3.567
3.562
3.544
3.540
3.526
3.522
3.508
3.261
3.250
3.238
3.227
2.912
2.901
2.888
2.878
2.870
2.852
2.725
2.696
2.684
2.661
2.650
2.627
2.591
2.580
2.569
2.494
2.475
2.128
2.121
2.093
2.086
1.949
1.935
1.921
1.914
1.907
1.900
1.886
1.748
1.154
1.136
H
CO2Me
O
N
H
Ph
3.31
4.14
2.88
0.95
1.02
2.98
1.15
0.99
2.07
0.97
3.12
0.92
1.06
0.97
0.96
0.95
0.95
3.03
3.00
f1 (ppm)

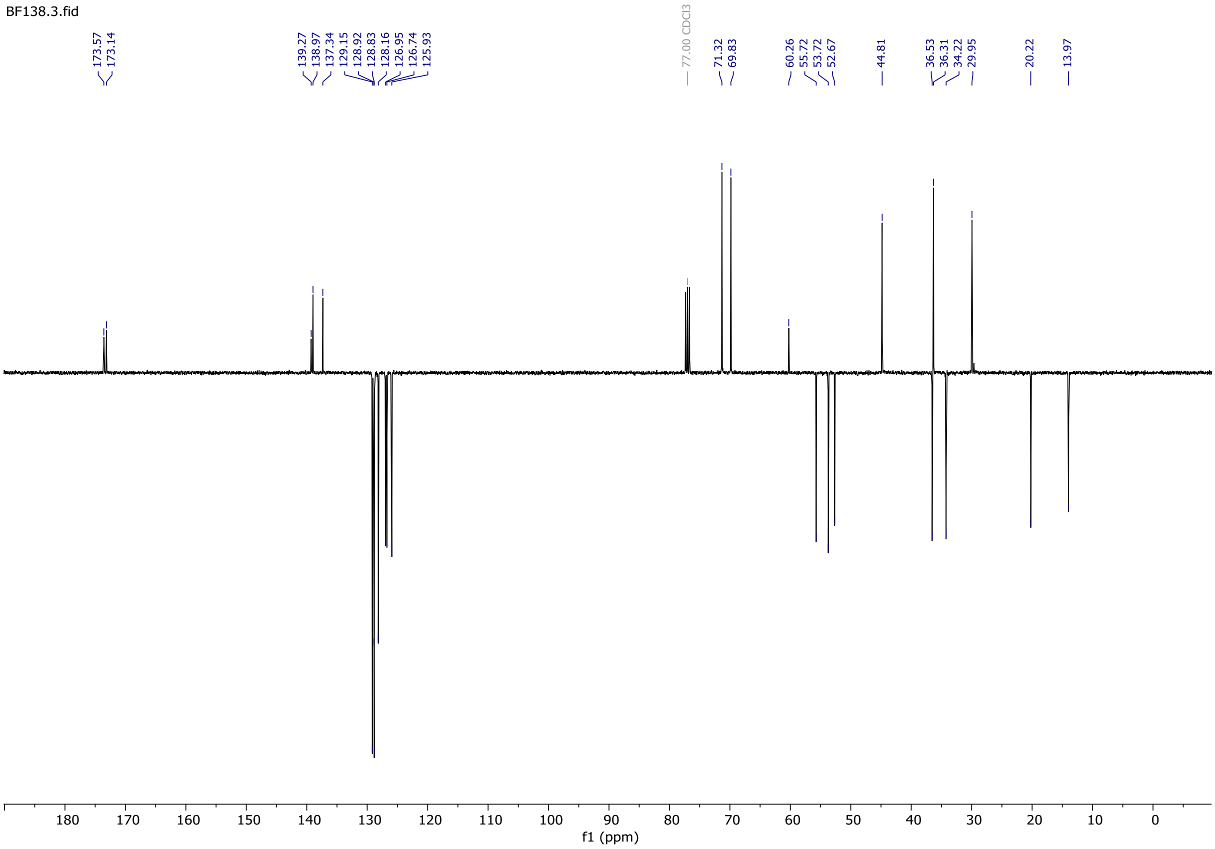
BF138.3.fid
173.57
173.14
139.27
138.97
137.34
129.15
128.92
128.83
128.16
126.95
126.74
125.93
77.00 CDCl3
71.32
69.83
60.26
55.72
53.72
52.67
44.81
36.53
36.31
34.22
29.95
20.22
13.97
f1 (ppm)

17b

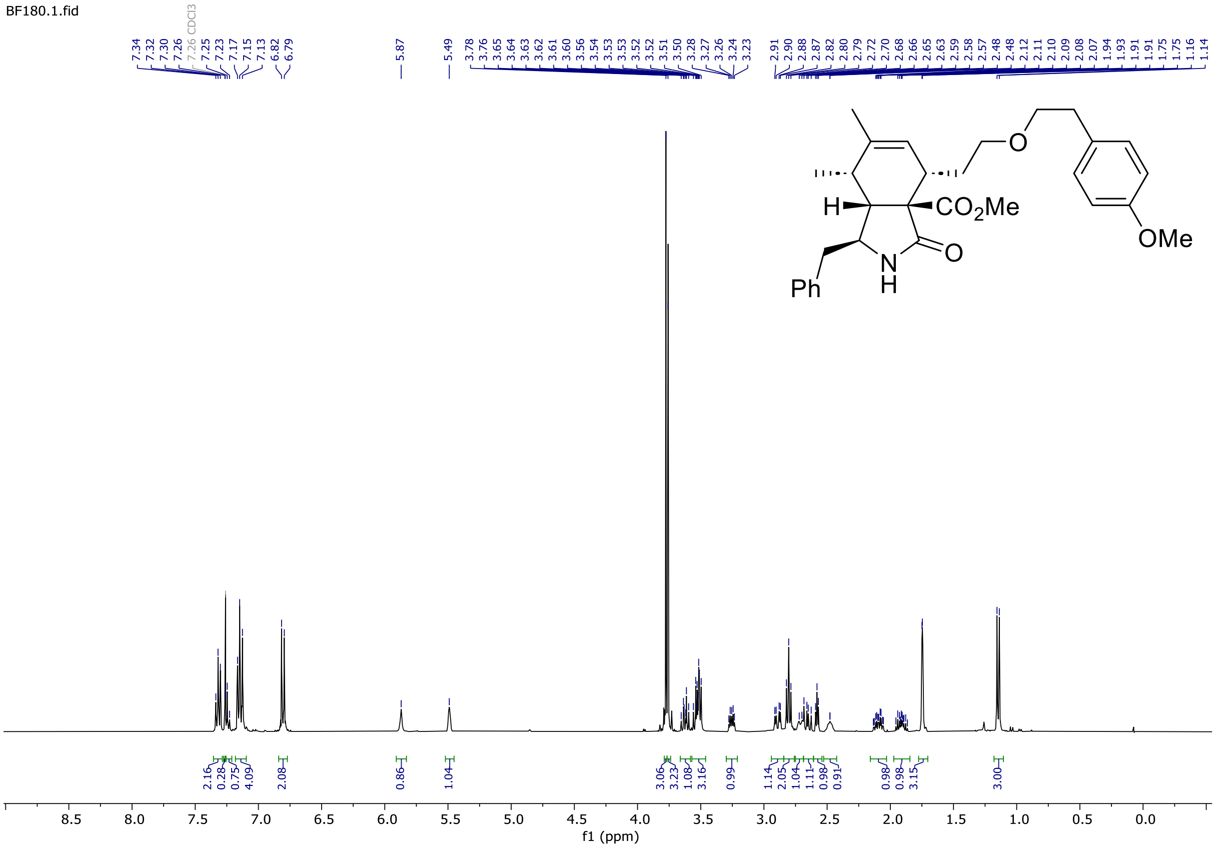
BF180.1.fid
7.34 7.32 7.30 7.26 CDCl3 7.25 7.23 7.17 7.15 7.13 6.82 6.79
5.87
5.49
3.78 3.76 3.65 3.64 3.63 3.62 3.61 3.60 3.56 3.54 3.53 3.53 3.52 3.52 3.51 3.50 3.28 3.27 3.26 3.24 3.23
2.91 2.90 2.88 2.87 2.82 2.80 2.79 2.72 2.70 2.68 2.66 2.65 2.63 2.59 2.58 2.57 2.48 2.48 2.12 2.11 2.10 2.09 2.08 2.07 1.94 1.93 1.91 1.91 1.91 1.75 1.75 1.16 1.14
CO2Me
H
N
H
O
Ph
O
OMe
2.16 0.28 0.75 4.09 2.08 0.86 1.04 3.06 3.23 1.08 3.16 0.99 1.14 2.05 1.04 1.11 0.98 0.91 0.98 0.98 3.15 3.00
f1 (ppm)

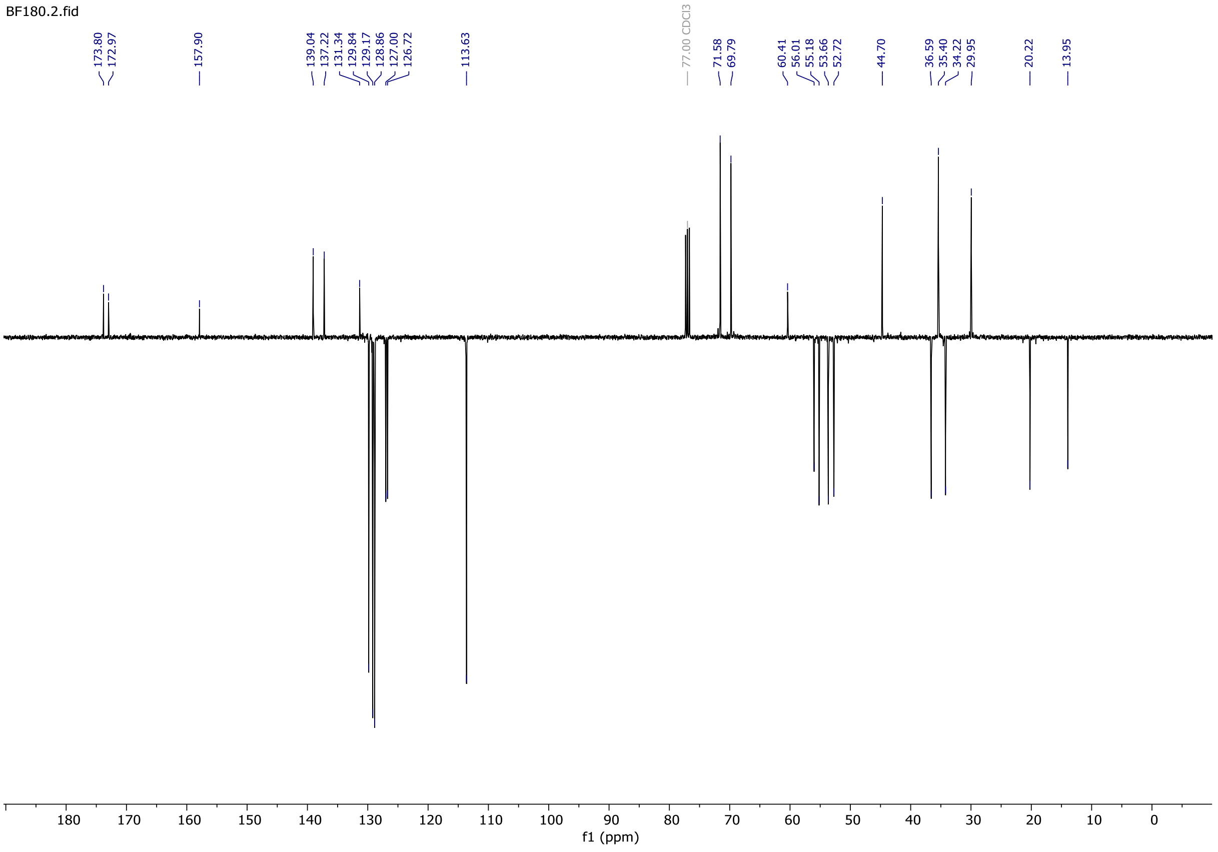
BF180.2.fid
173.80 172.97
157.90
139.04 137.22 131.34 129.84 129.17 128.86 127.00 126.72
113.63
77.00 CDCl3
71.58 69.79
60.41 56.01 55.18 53.66 52.72
44.70
36.59 35.40 34.22 29.95
20.22
13.95
f1 (ppm)

17c

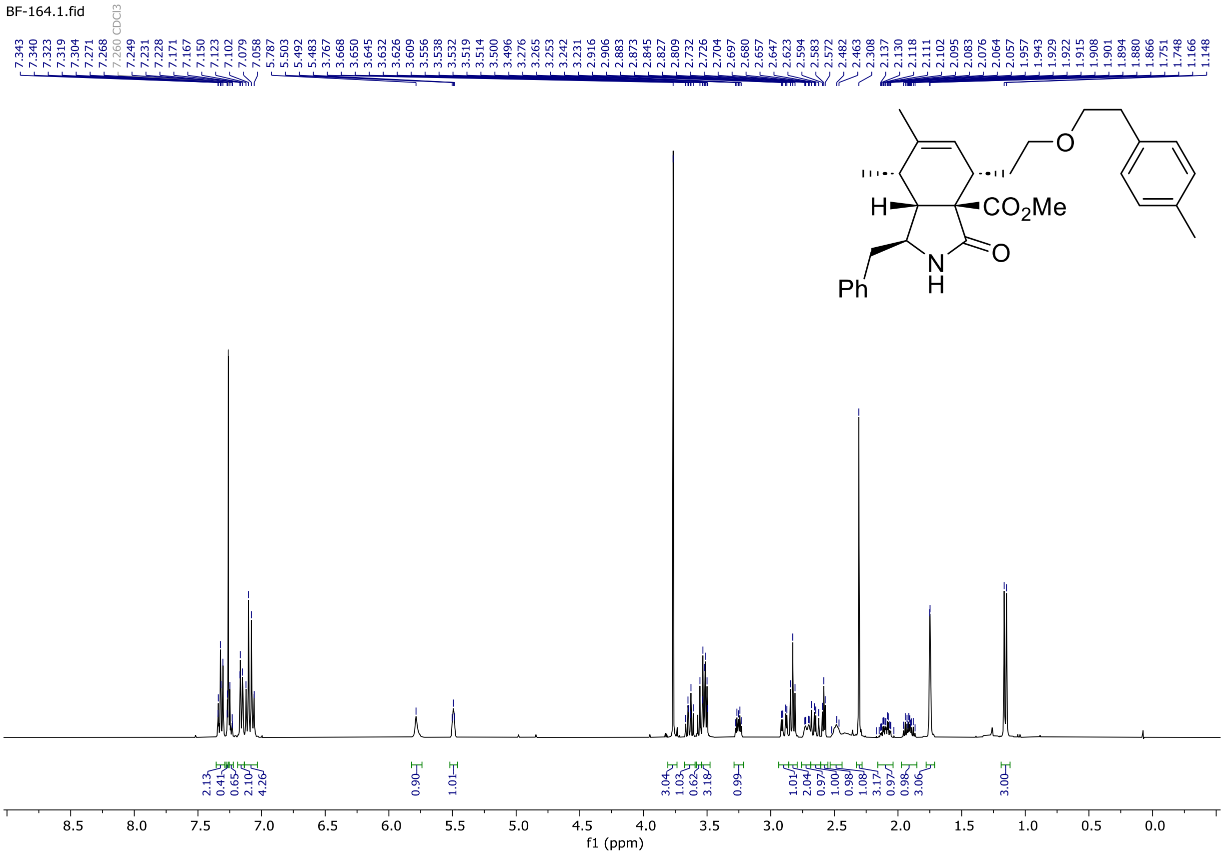

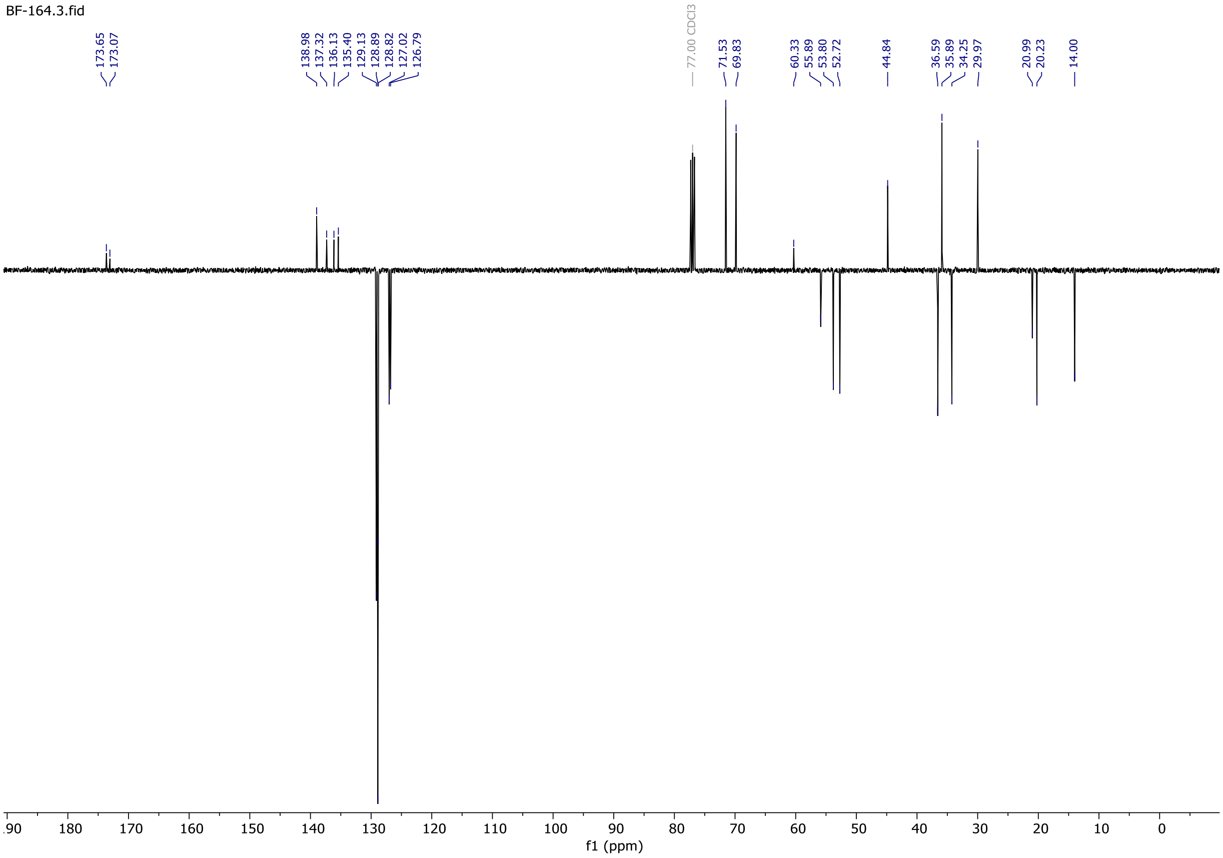

17d

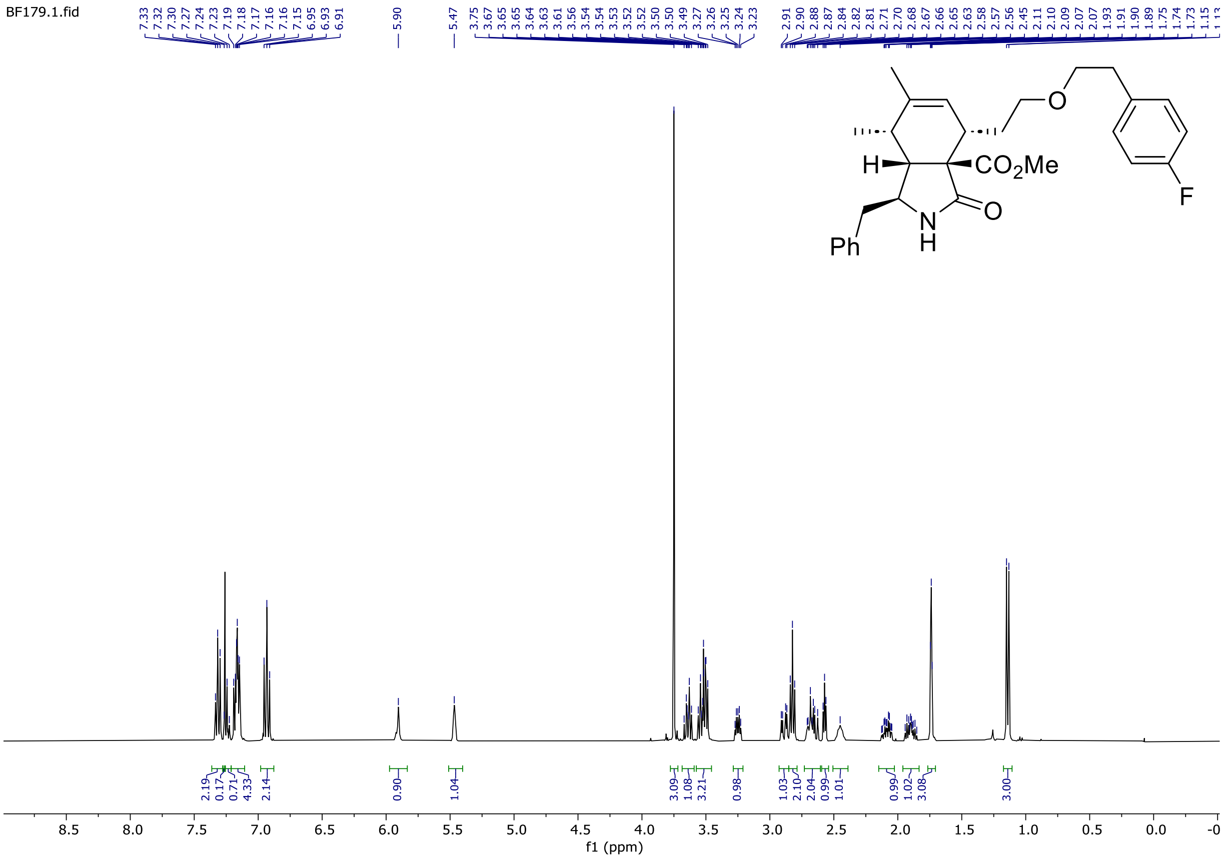

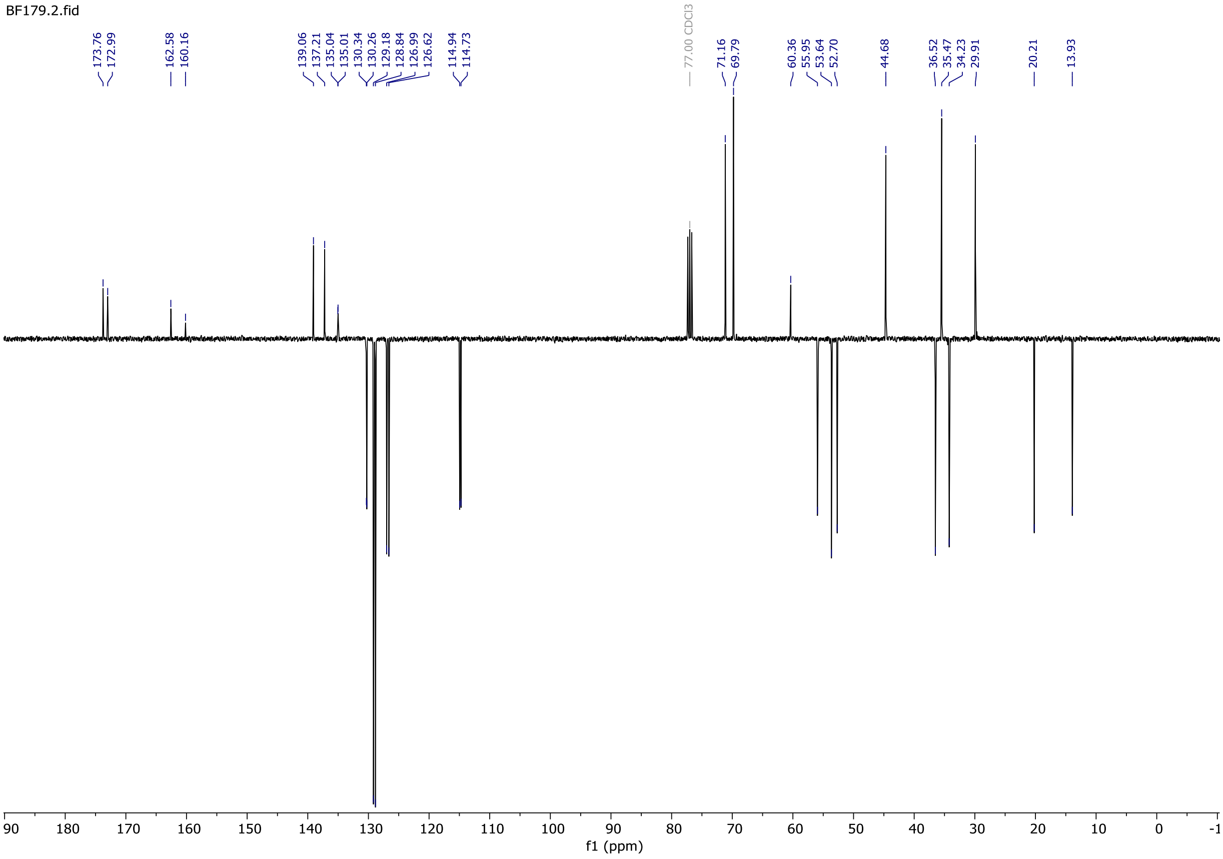

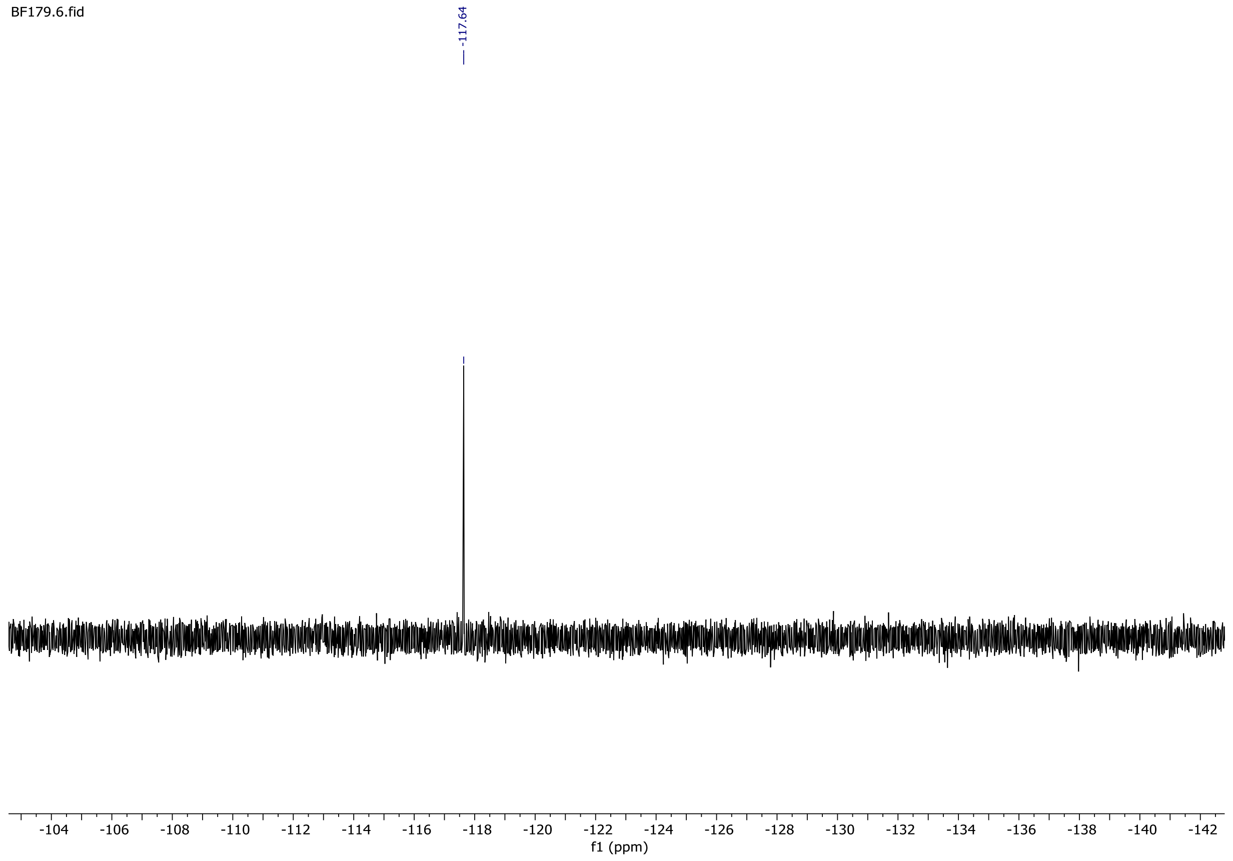
BF179.6.fid
-117.64
-104
-106
-108
-110
-112
-114
-116
-118
-120
-122
-124
-126
-128
-130
-132
-134
-136
-138
-140
-142
f1 (ppm)

17e

BF-167.1.fid

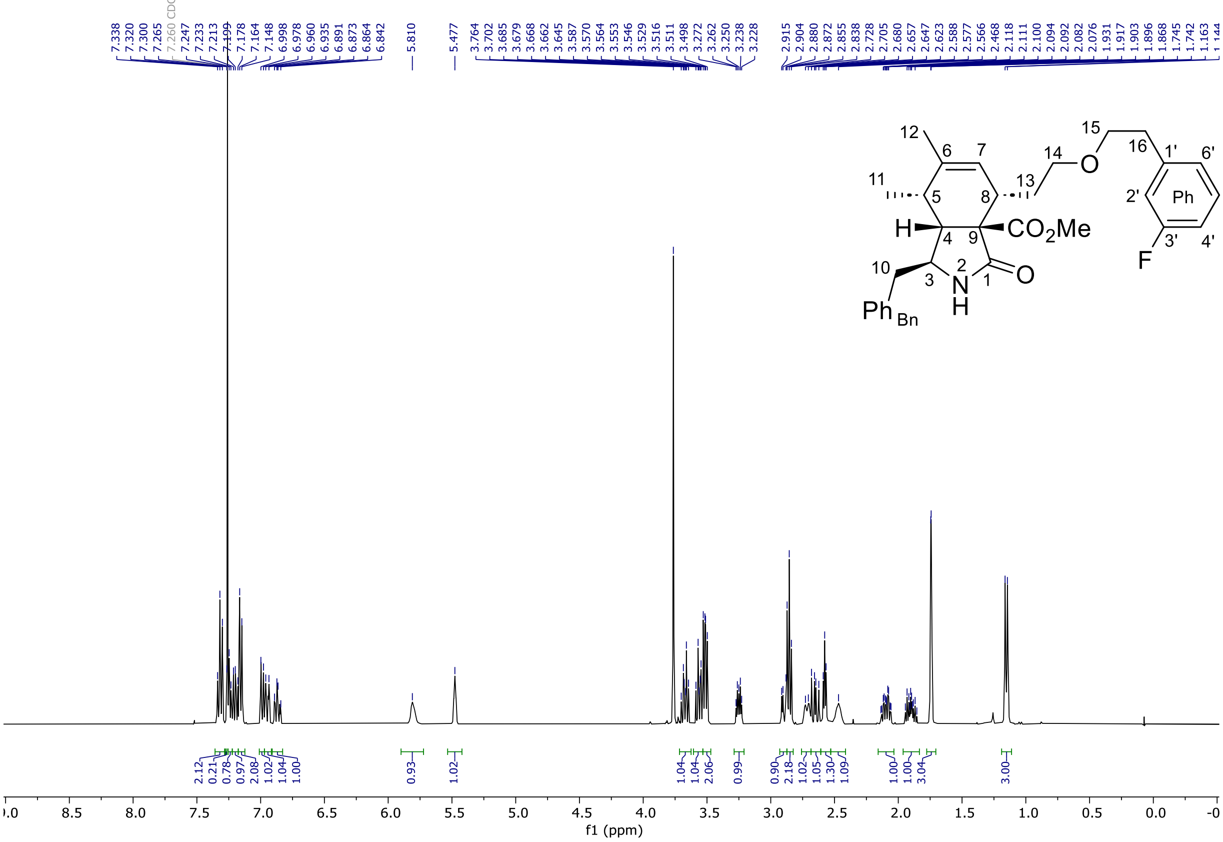

BF-167.4.fid

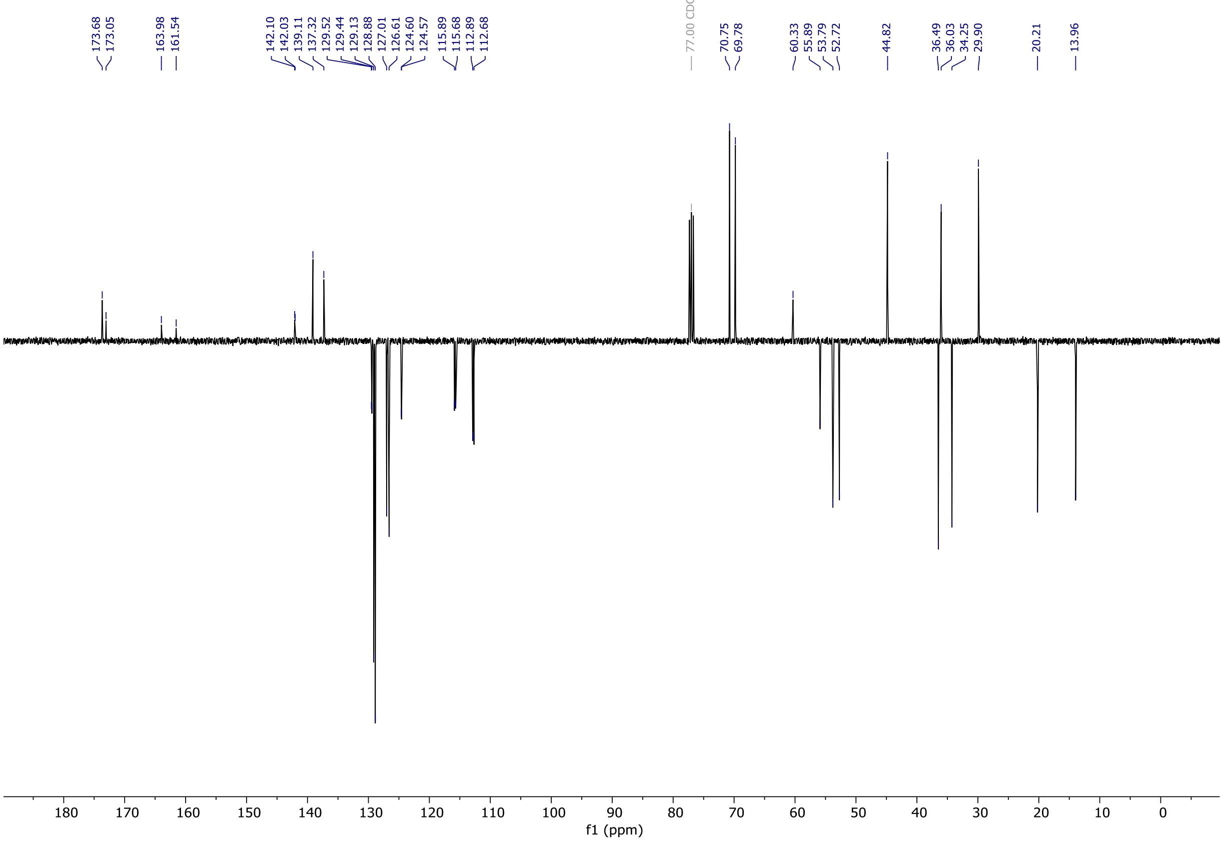

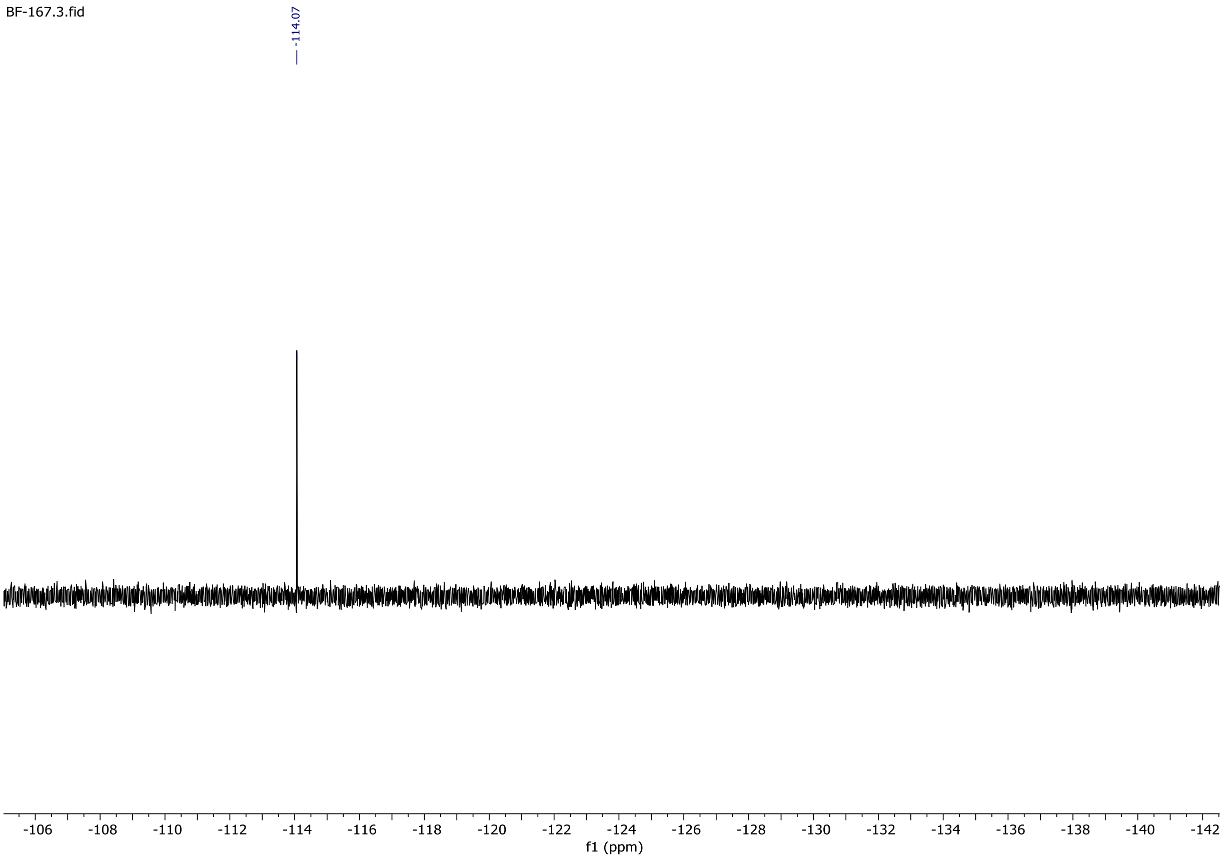

BF-167.3.fid
-114.07
-106
-108
-110
-112
-114
-116
-118
-120
-122
-124
-126
-128
-130
-132
-134
-136
-138
-140
-142
f1 (ppm)

17f

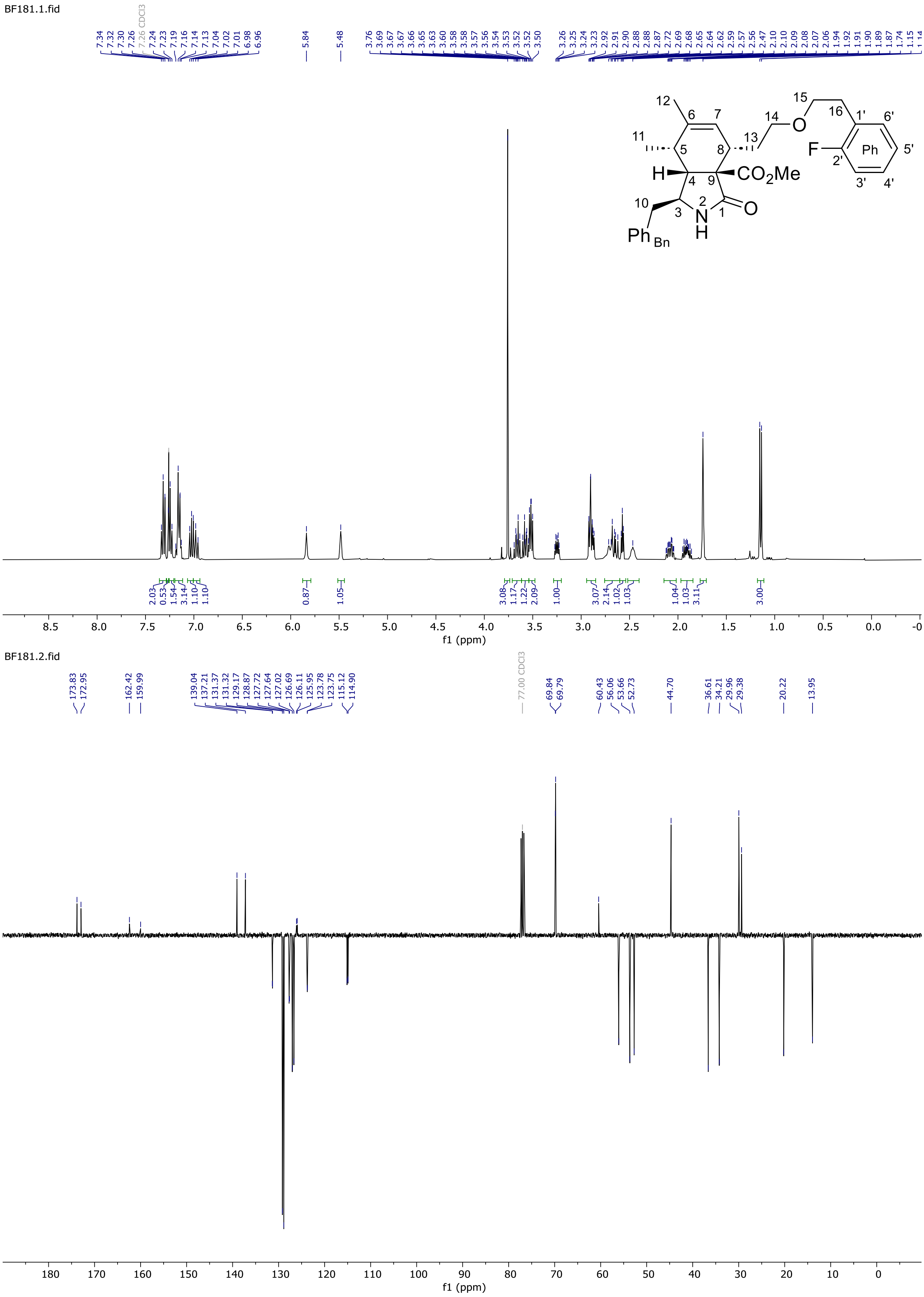

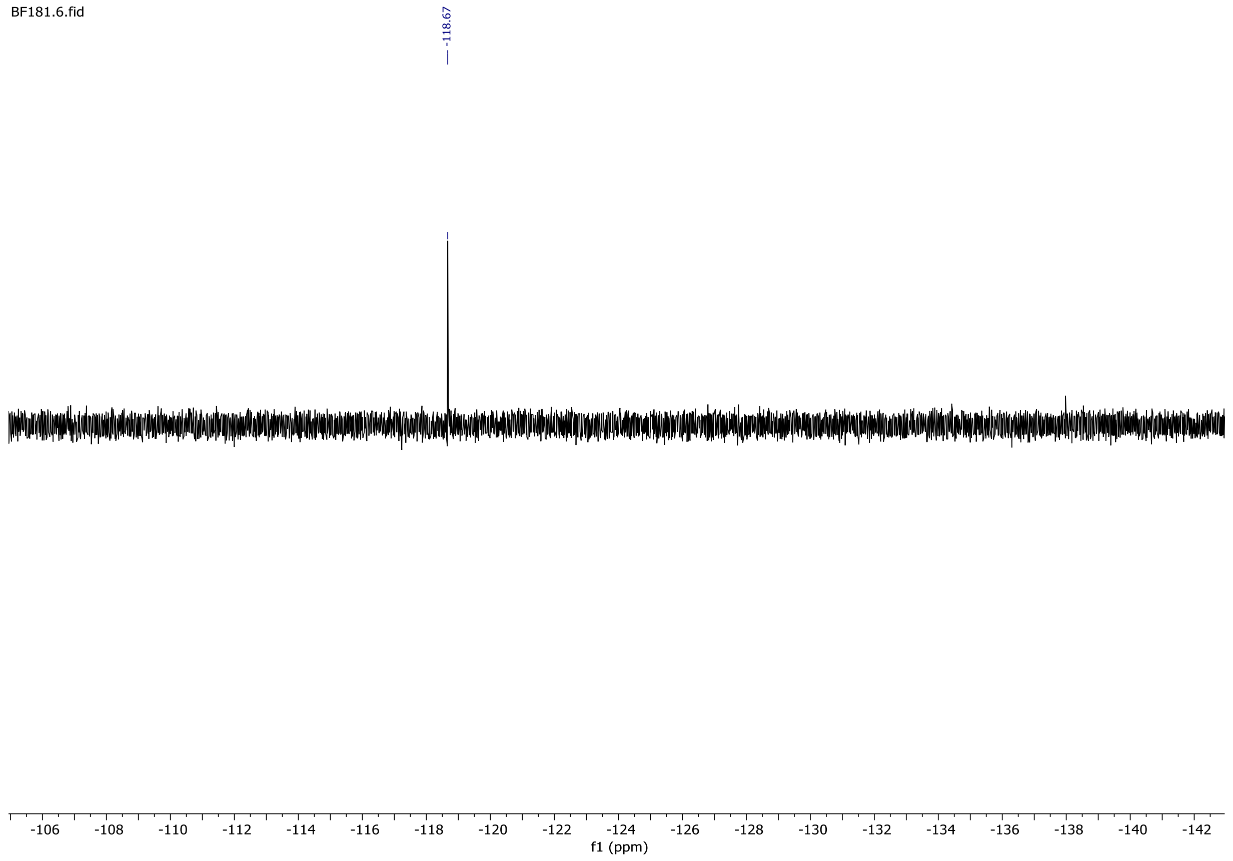
BF181.6.fid
-118.67
-106
-108
-110
-112
-114
-116
-118
-120
-122
-124
-126
-128
-130
-132
-134
-136
-138
-140
-142
f1 (ppm)

17g

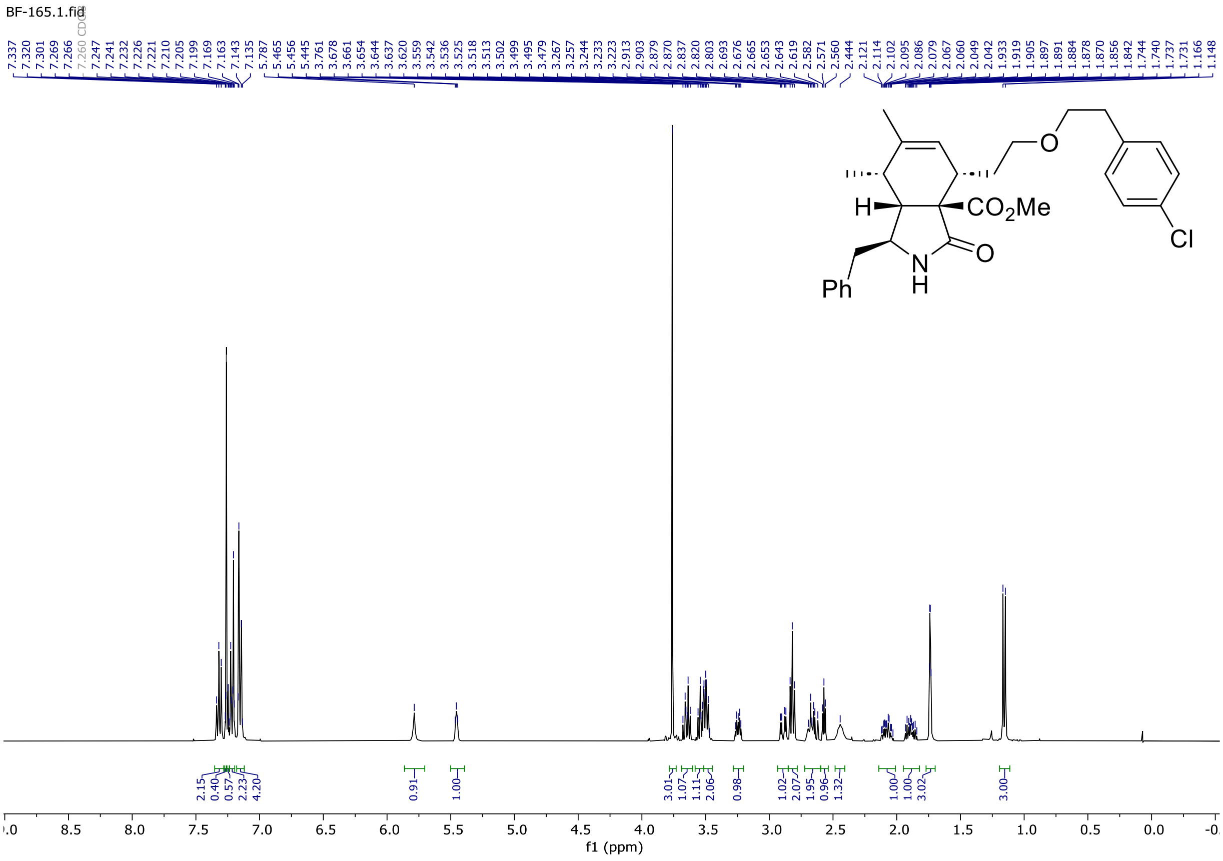

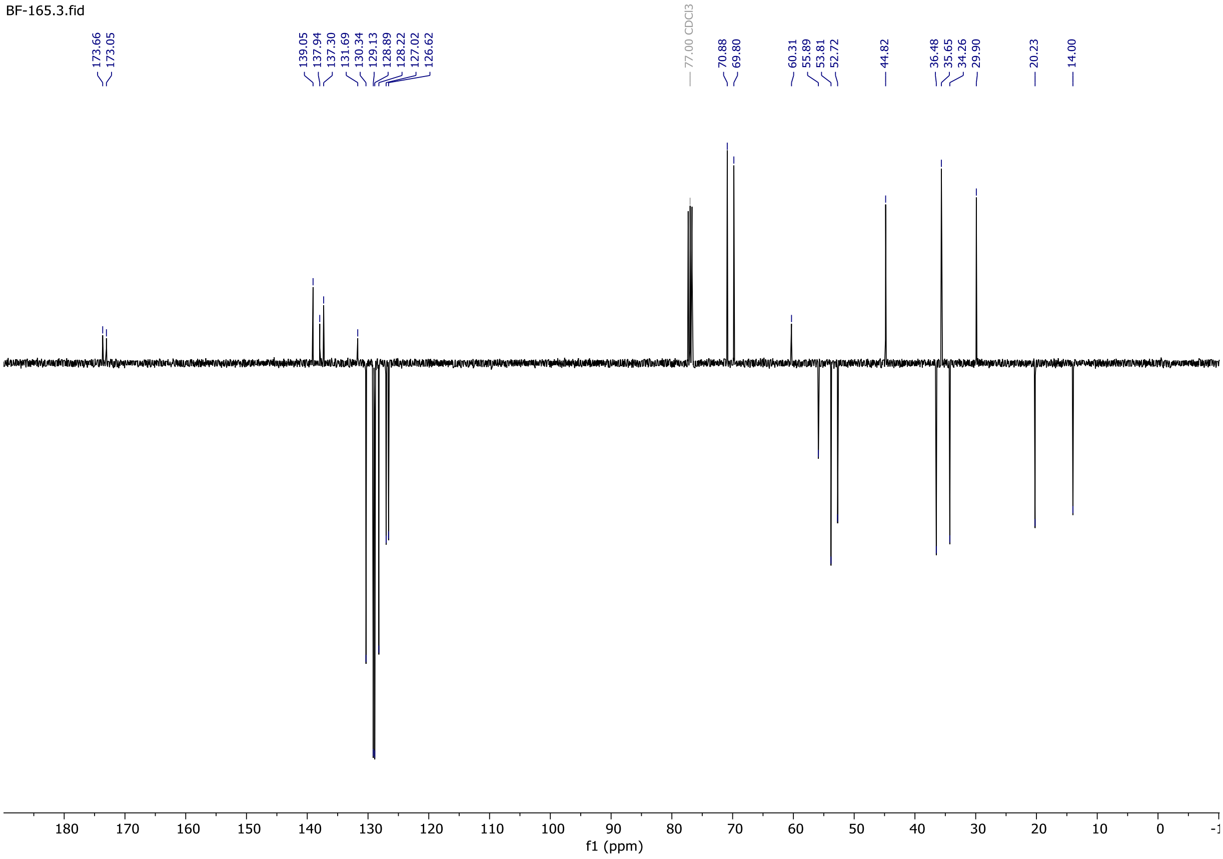

17h

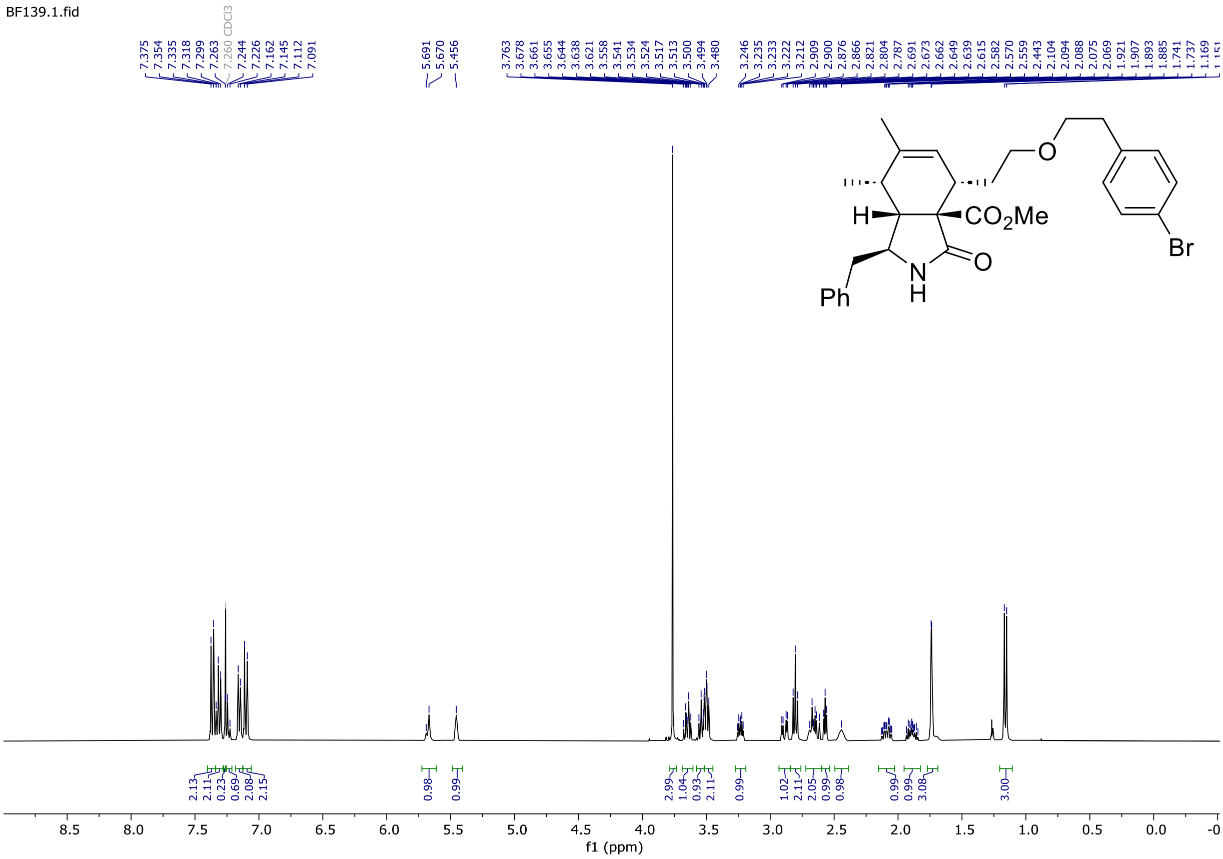
BF139.1.fid
7.375
7.354
7.335
7.318
7.299
7.263
7.260 CDCl3
7.244
7.226
7.162
7.145
7.112
7.091
5.691
5.670
5.456
3.763
3.678
3.661
3.655
3.644
3.638
3.621
3.558
3.541
3.534
3.524
3.517
3.513
3.500
3.494
3.480
3.246
3.235
3.233
3.222
3.212
2.909
2.900
2.876
2.866
2.821
2.804
2.787
2.691
2.673
2.662
2.649
2.639
2.615
2.582
2.570
2.559
2.443
2.104
2.094
2.088
2.075
2.069
1.921
1.907
1.893
1.885
1.741
1.737
1.169
CO2Me
H
O
N
H
Ph
O
Br
2.13
2.11
0.23
0.69
2.08
2.15
0.98
0.99
2.99
1.04
0.93
2.11
0.99
1.02
2.11
2.05
0.99
0.98
0.99
0.99
3.08
3.00
f1 (ppm)

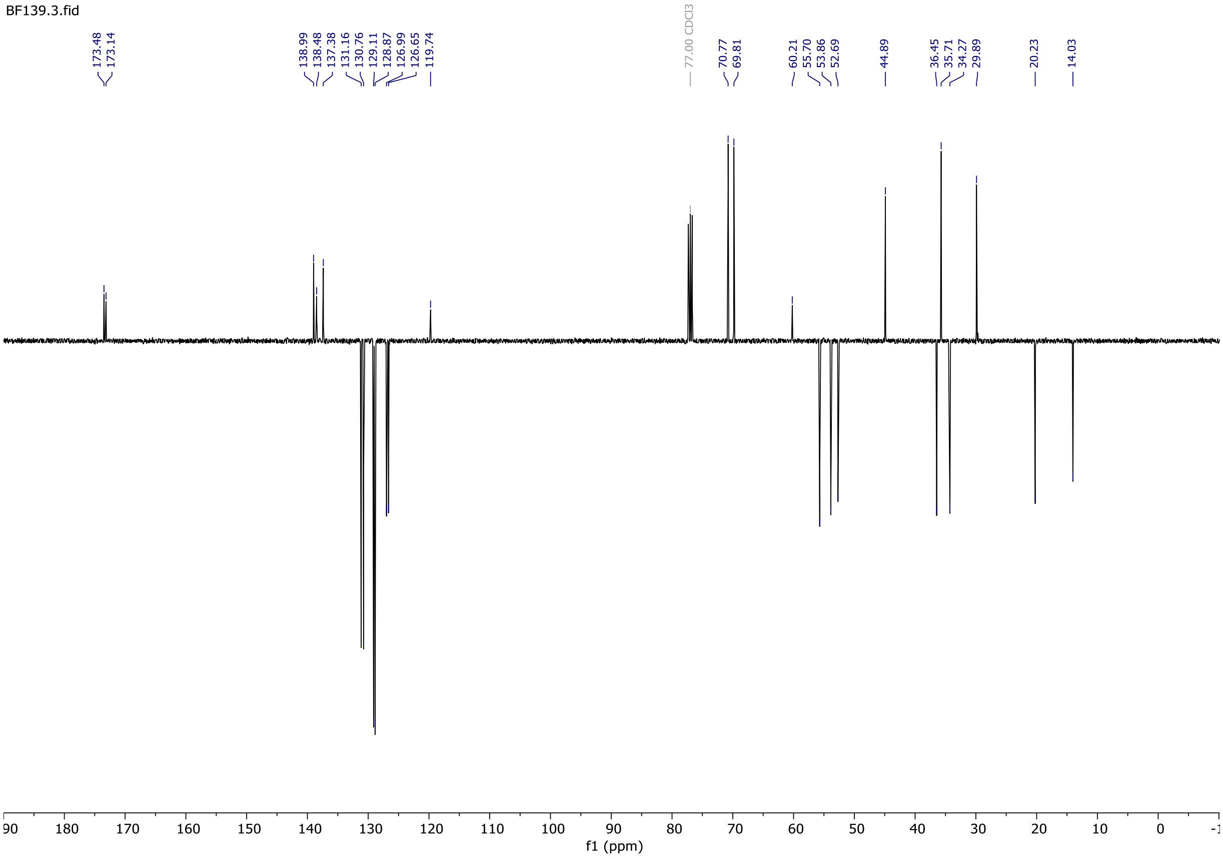
BF139.3.fid
173.48
173.14
138.99
138.48
137.38
131.16
130.76
129.11
128.87
126.99
126.65
119.74
77.00 CDCl3
70.77
69.81
60.21
55.70
53.86
52.69
44.89
36.45
35.71
34.27
29.89
20.23
14.03
f1 (ppm)

17i

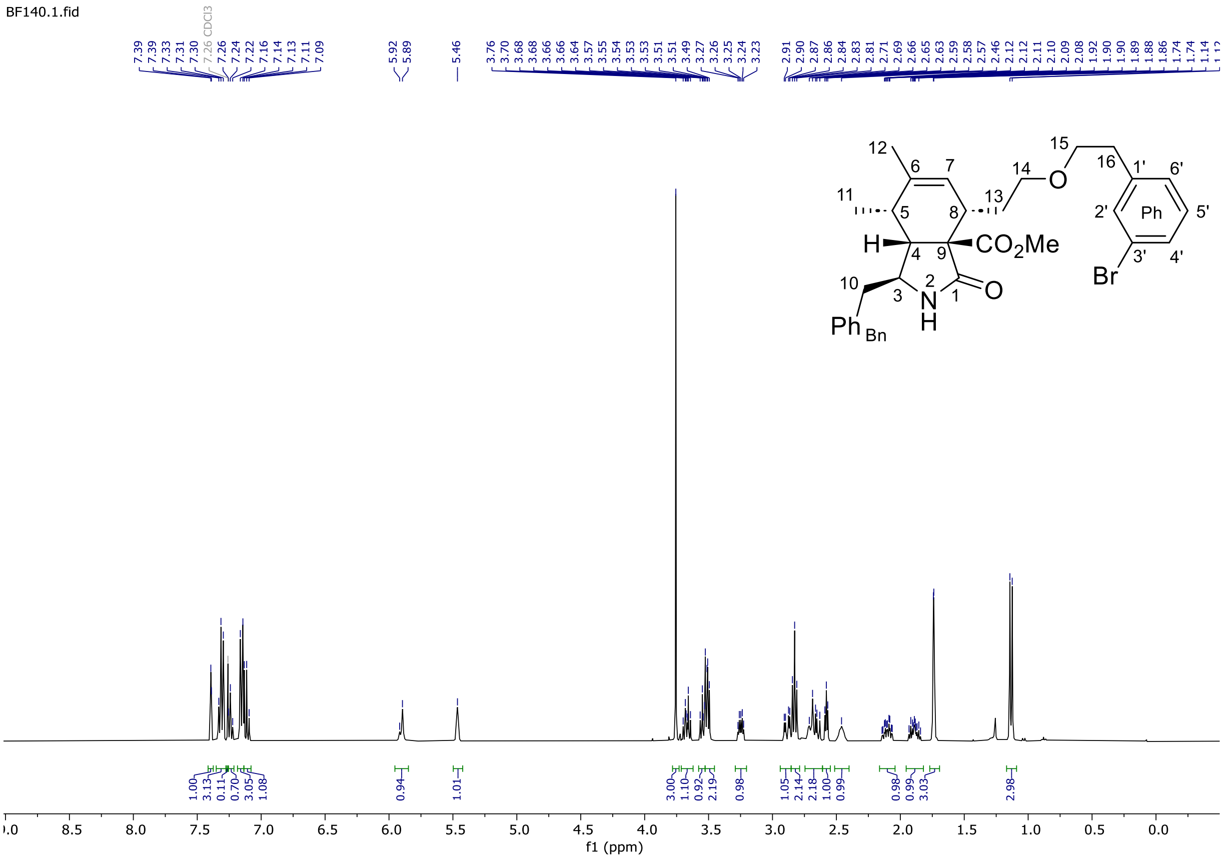
BF140.1.fid
7.39 7.39 7.33 7.31 7.30 7.26 CDCl3 7.26 7.24 7.22 7.16 7.14 7.13 7.11 7.09
5.92 5.89
5.46
3.76 3.70 3.68 3.68 3.66 3.66 3.64 3.57 3.55 3.54 3.53 3.53 3.51 3.51 3.49 3.27 3.26 3.25 3.24 3.23
2.91 2.90 2.87 2.86 2.84 2.83 2.81 2.71 2.69 2.66 2.65 2.63 2.59 2.58 2.57 2.46 2.12 2.12 2.11 2.10 2.09 2.08 1.92 1.90 1.89 1.88 1.86 1.74 1.74 1.14
12
11
6
7
5
8
13
14
O
15
16
1'
6'
2'
Ph
5'
3'
4'
Br
H
4
9
CO2Me
10
3
2
N
H
1
O
Ph
Bn
1.00 3.13 0.11 0.70 3.05 1.08
0.94
1.01
3.00 1.10 0.92 2.19
0.98
1.05 2.14 2.18 1.00 0.99
0.98 0.99 3.03
2.98
f1 (ppm)

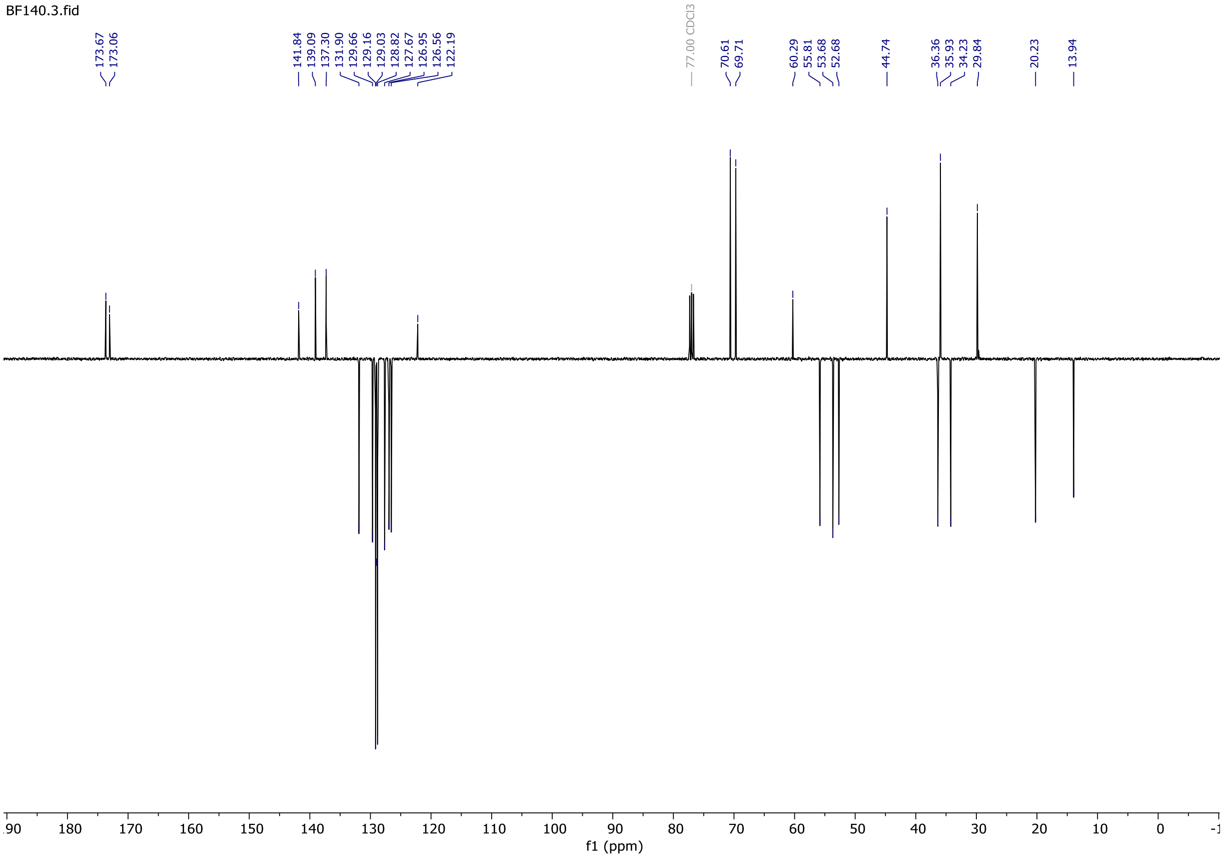
BF140.3.fid
173.67 173.06
141.84 139.09 137.30 131.90 129.66 129.16 129.03 128.82 127.67 126.95 126.56 122.19
77.00 CDCl3
70.61 69.71
60.29 55.81 53.68 52.68
44.74
36.36 35.93 34.23 29.84
20.23
13.94
f1 (ppm)

17j

BF-168.1.fid

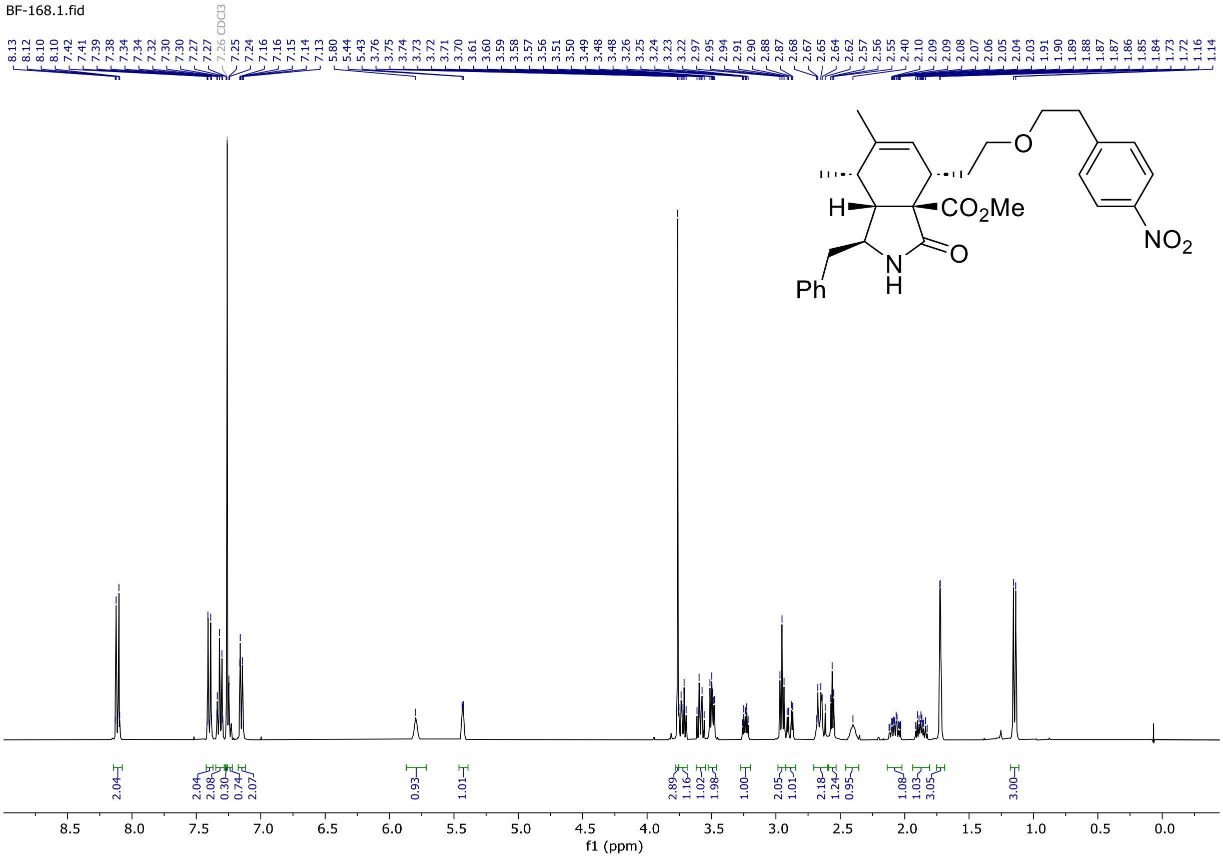

BF-168.3.fid

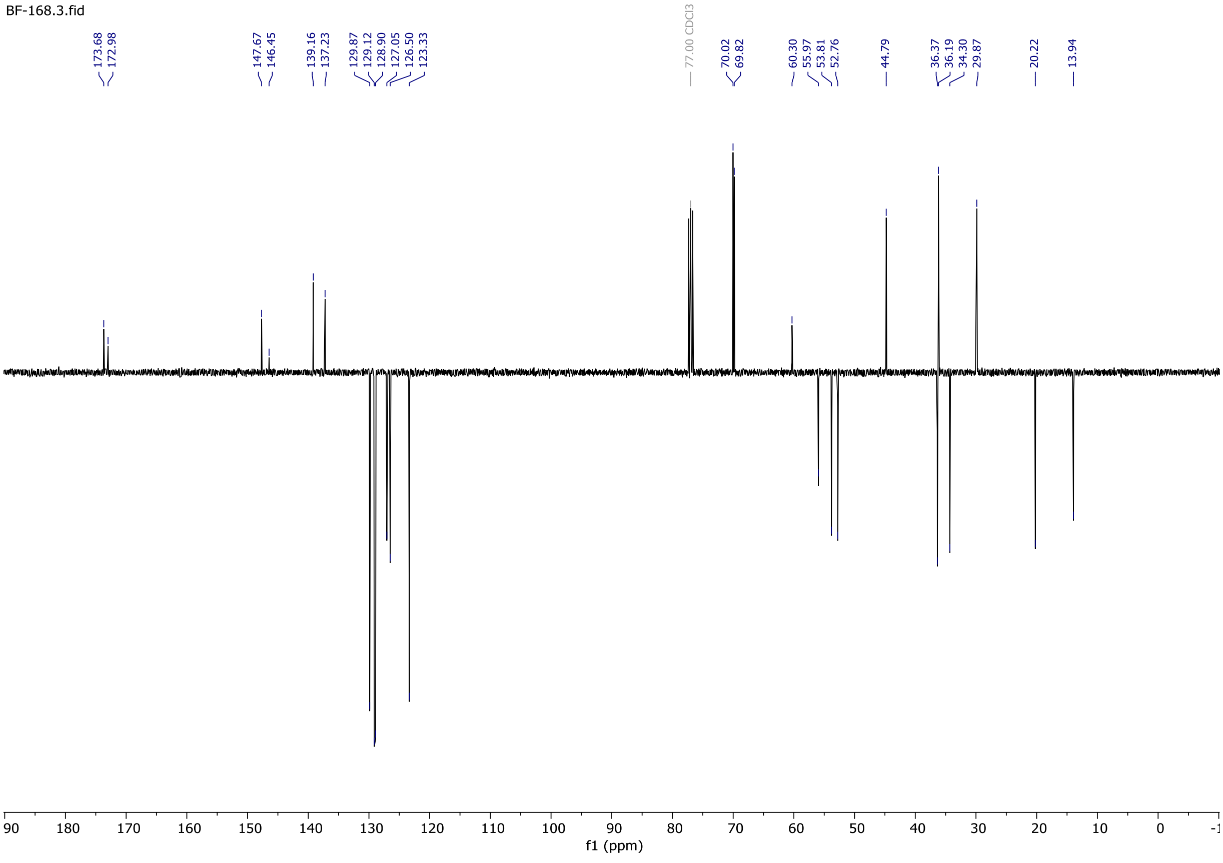

19

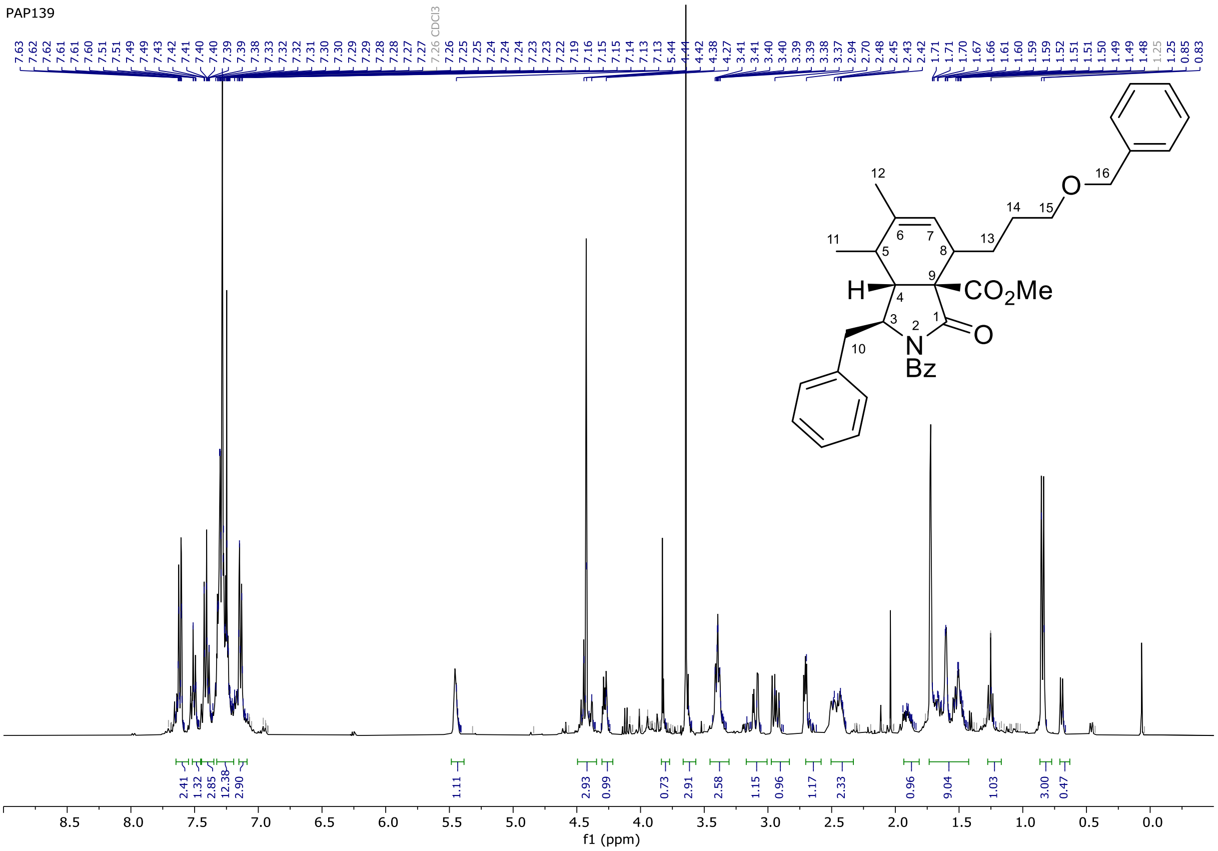

PAP139
7.63 7.62 7.62 7.61 7.61 7.60 7.51 7.51 7.49 7.49 7.43 7.42 7.41 7.40 7.40 7.39 7.39 7.38 7.33 7.32 7.32 7.31 7.30 7.30 7.29 7.29 7.28 7.28 7.27 7.27 7.26 CDCl3 7.26 7.25 7.25 7.24 7.24 7.24 7.23 7.23 7.22 7.19 7.16 7.15 7.15 7.14 7.13 7.13 5.44 4.44 4.42 4.38 4.27 3.41 3.41 3.40 3.40 3.39 3.39 3.38 3.37 2.94 2.70 2.48 2.45 2.43 2.42 1.71 1.71 1.70 1.67 1.66 1.61 1.60 1.59 1.59 1.52 1.51 1.51 1.50 1.49 1.49 1.48 1.25 1.25 0.85 0.83
2.41 1.32 2.85 12.38 2.90 1.11 2.93 0.99 0.73 2.91 2.58 1.15 0.96 1.17 2.33 0.96 9.04 1.03 3.00 0.47
CO2Me
O
N
Bz
H
O
f1 (ppm)

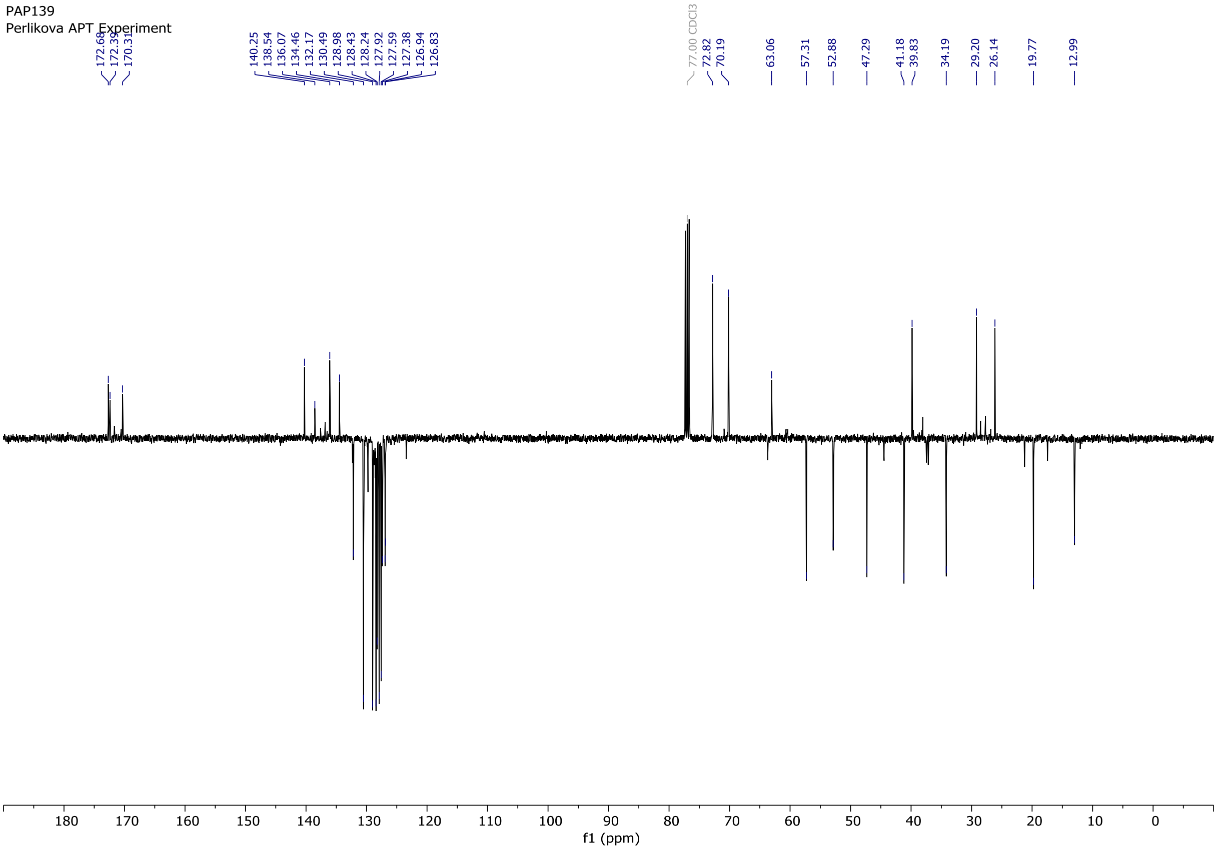

PAP139
Perlikova APT Experiment
172.68 172.39 170.31
140.25 138.54 136.07 134.46 132.17 130.49 128.98 128.43 128.24 127.92 127.59 127.38 126.94 126.83
77.00 CDCl3 72.82 70.19
63.06 57.31 52.88 47.29 41.18 39.83 34.19 29.20 26.14 19.77 12.99
f1 (ppm)

20

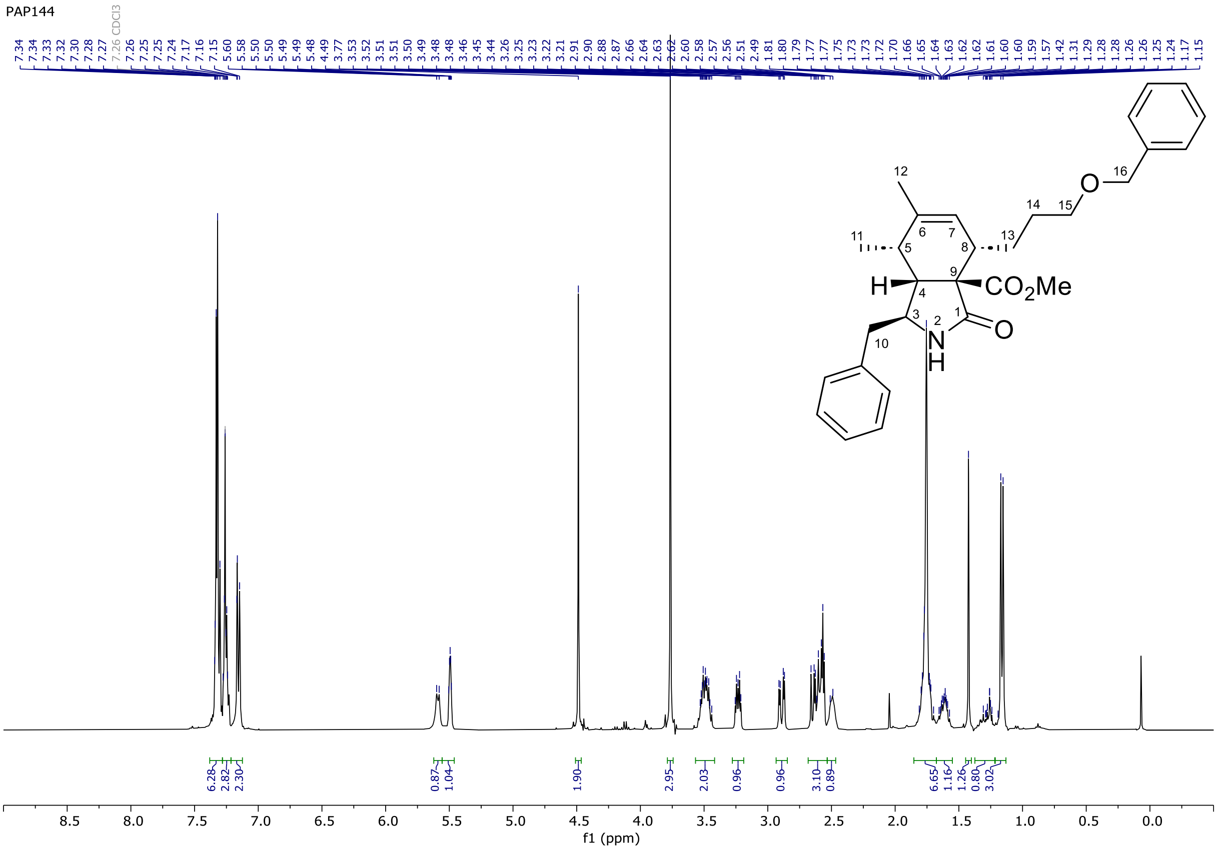

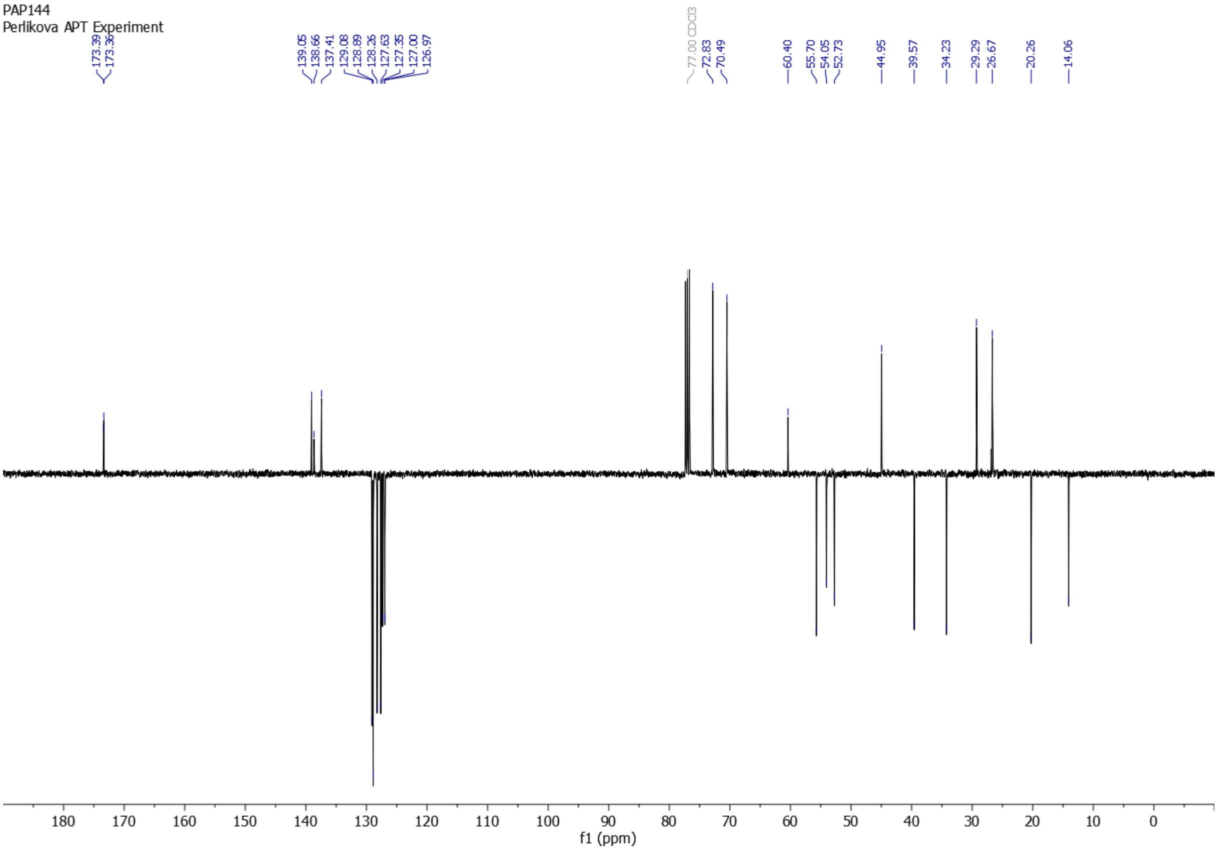

21a

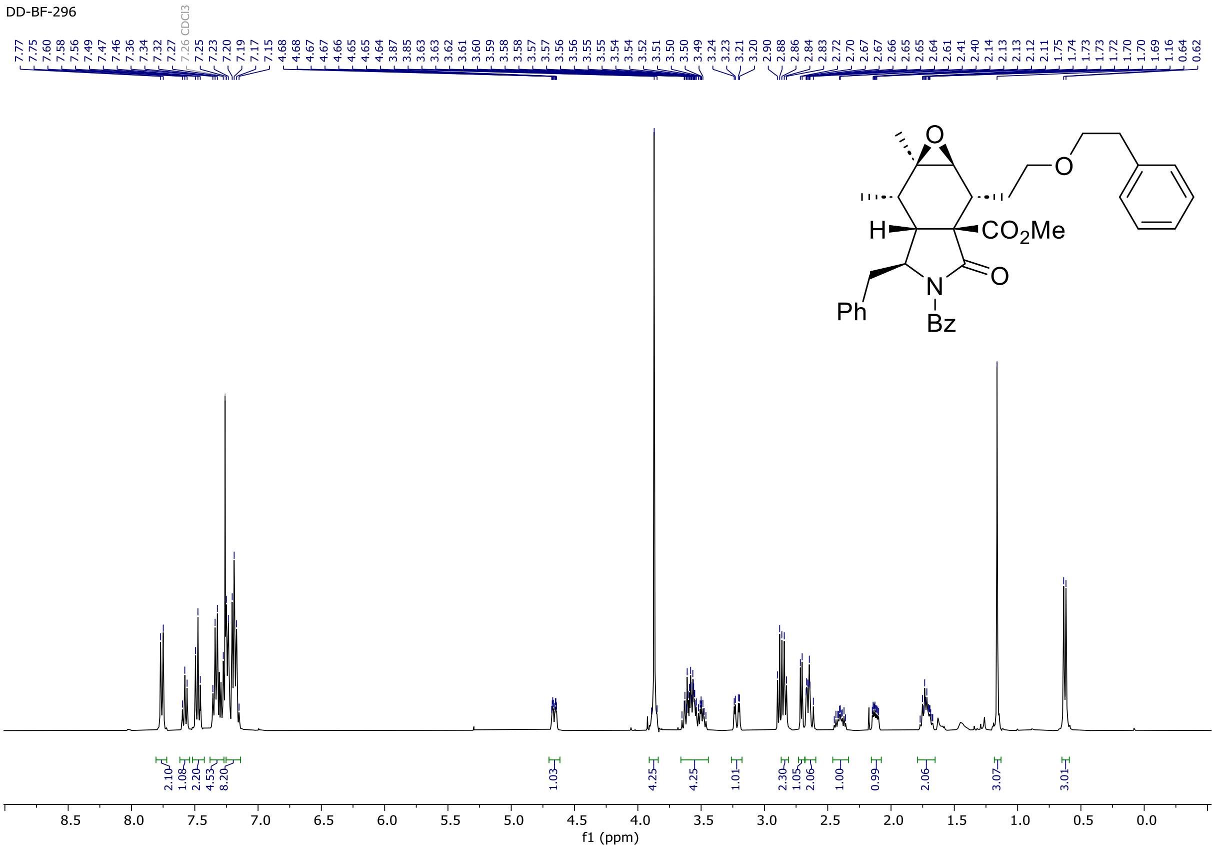
DD-BF-296
7.77 7.75 7.60 7.58 7.56 7.49 7.47 7.46 7.36 7.34 7.32 7.27 7.26 CDCl3 7.25 7.23 7.20 7.19 7.17 7.15 4.68 4.67 4.67 4.66 4.65 4.65 4.64 3.87 3.85 3.63 3.63 3.62 3.61 3.60 3.59 3.58 3.58 3.57 3.57 3.56 3.56 3.55 3.54 3.54 3.52 3.51 3.50 3.50 3.49 3.24 3.23 3.21 3.20 2.90 2.88 2.86 2.84 2.83 2.72 2.70 2.67 2.67 2.66 2.65 2.65 2.64 2.61 2.41 2.40 2.14 2.13 2.13 2.12 2.11 1.75 1.74 1.73 1.73 1.72 1.70 1.70 1.69 1.16 0.64 0.62
O
O
H
CO_2Me
N
O
Ph
Bz
2.10 1.08 2.20 4.53 8.20 1.03 4.25 4.25 1.01 2.30 1.05 2.06 1.00 0.99 2.06 3.07 3.01
8.5 8.0 7.5 7.0 6.5 6.0 5.5 5.0 4.5 4.0 3.5 3.0 2.5 2.0 1.5 1.0 0.5 0.0
f1 (ppm)

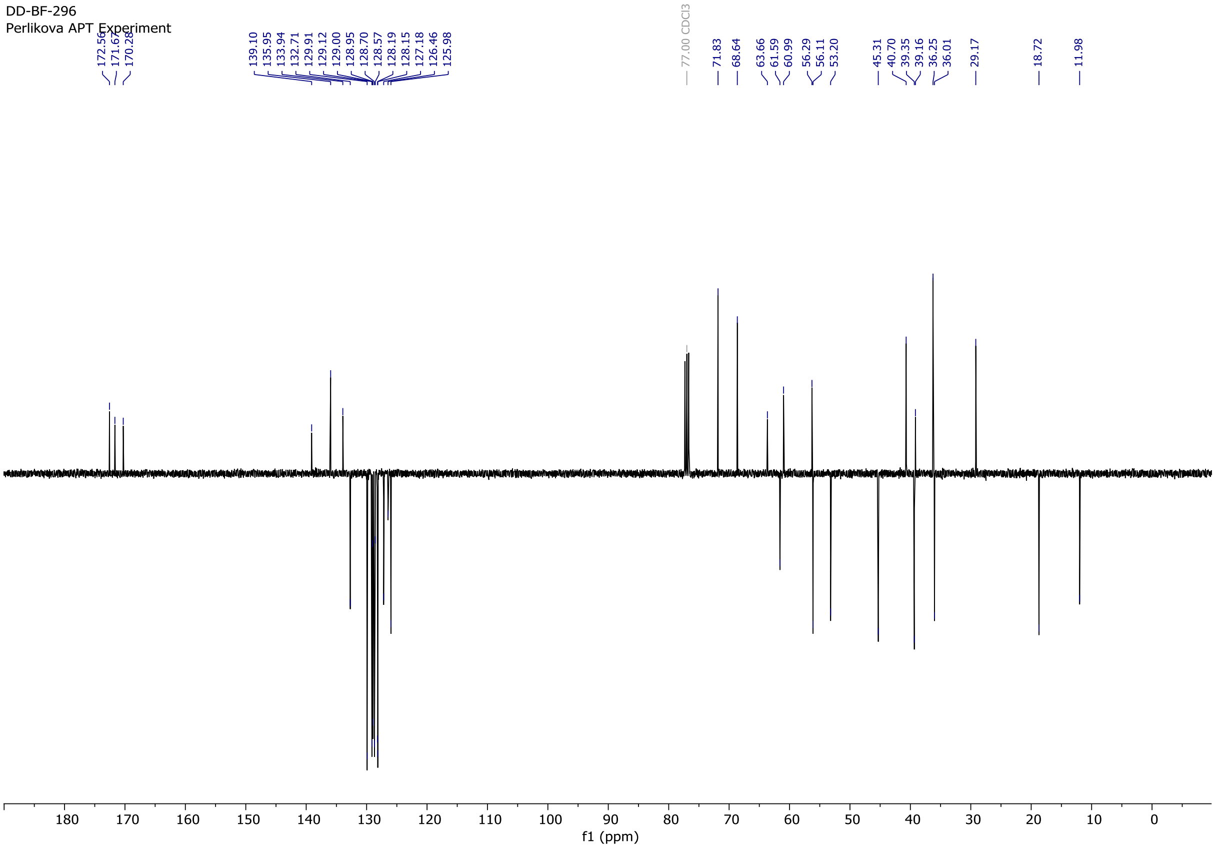
DD-BF-296
Perlikova APT Experiment
172.56 171.67 170.28 139.10 135.95 133.94 132.71 129.91 129.12 129.00 128.95 128.70 128.57 128.19 128.15 127.18 126.46 125.98 77.00 CDCl3 71.83 68.64 63.66 61.59 60.99 56.29 56.11 53.20 45.31 40.70 39.35 39.16 36.25 36.01 29.17 18.72 11.98
180 170 160 150 140 130 120 110 100 90 80 70 60 50 40 30 20 10 0
f1 (ppm)

21b

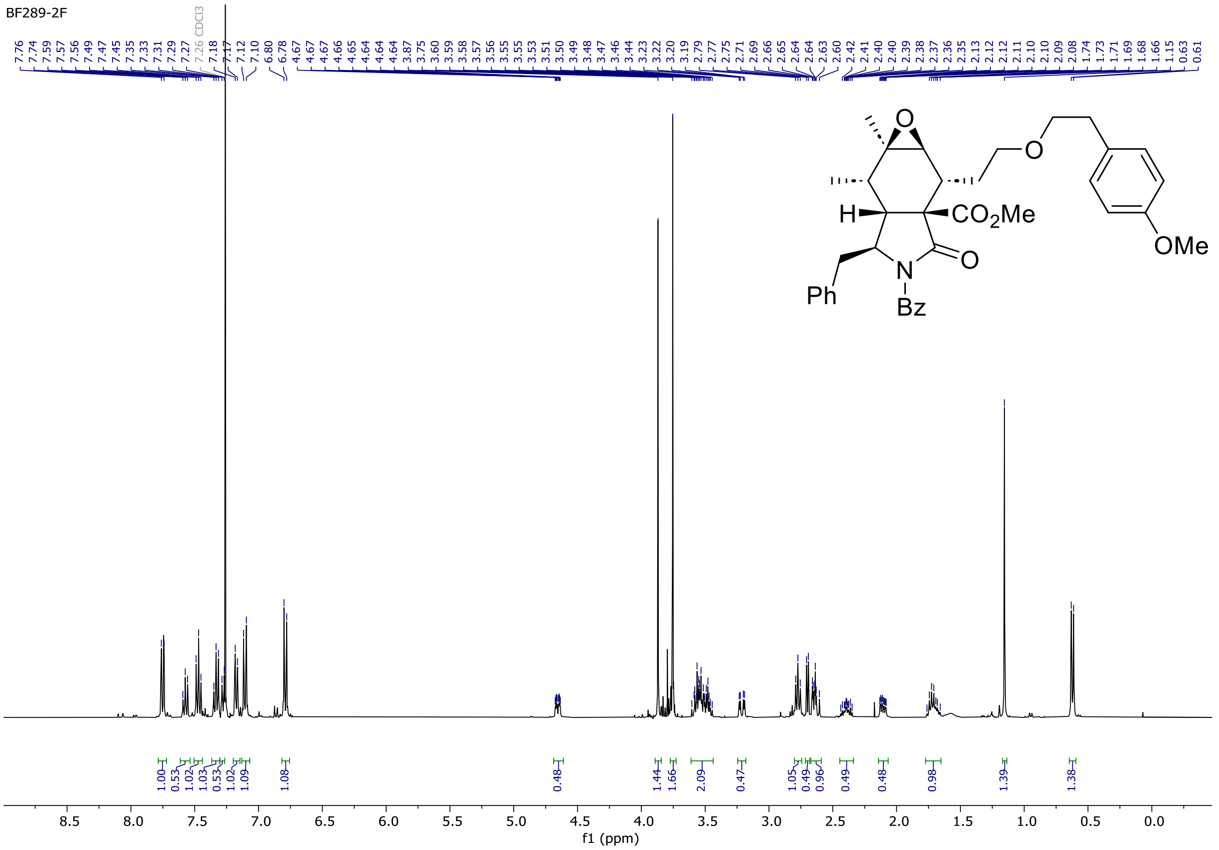
BF289-2F
7.76 7.74 7.59 7.57 7.56 7.49 7.47 7.45 7.35 7.33 7.31 7.29 7.27 7.26 CDCl3 7.18 7.17 7.12 7.10 6.80 6.78 4.67 4.67 4.66 4.65 4.64 4.64 4.64 3.87 3.75 3.60 3.59 3.58 3.57 3.56 3.55 3.55 3.53 3.51 3.50 3.49 3.48 3.47 3.46 3.44 3.23 3.22 3.20 3.19 2.79 2.77 2.75 2.71 2.69 2.66 2.65 2.64 2.64 2.63 2.60 2.42 2.41 2.40 2.40 2.39 2.38 2.37 2.36 2.35 2.13 2.12 2.12 2.11 2.10 2.10 2.09 2.08 1.74 1.73 1.71 1.69 1.68 1.66 1.15 0.63 0.61
O
CO2Me
H
O
OMe
N
Ph
Bz
1.00 0.53 1.02 1.03 0.53 1.02 1.09 1.08 0.48 1.44 1.66 2.09 0.47 1.05 0.49 0.96 0.49 0.48 0.98 1.39 1.38
8.5 8.0 7.5 7.0 6.5 6.0 5.5 5.0 4.5 4.0 3.5 3.0 2.5 2.0 1.5 1.0 0.5 0.0
f1 (ppm)

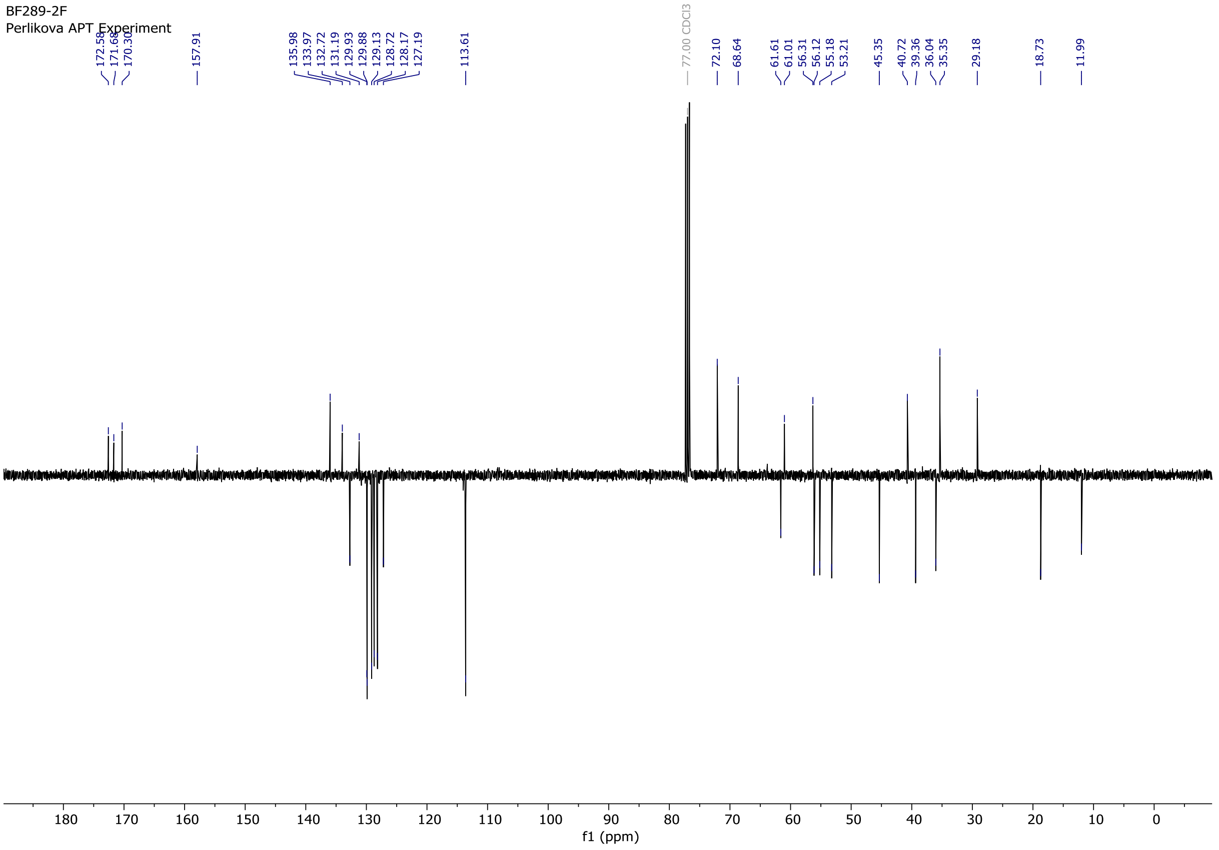
BF289-2F
Perlikova APT Experiment
172.58 171.68 170.30 157.91 135.98 133.97 132.72 131.19 129.93 129.88 129.13 128.72 128.17 127.19 113.61 77.00 CDCl3 72.10 68.64 61.61 61.01 56.31 56.12 55.18 53.21 45.35 40.72 39.36 36.04 35.35 29.18 18.73 11.99
180 170 160 150 140 130 120 110 100 90 80 70 60 50 40 30 20 10 0
f1 (ppm)

21h

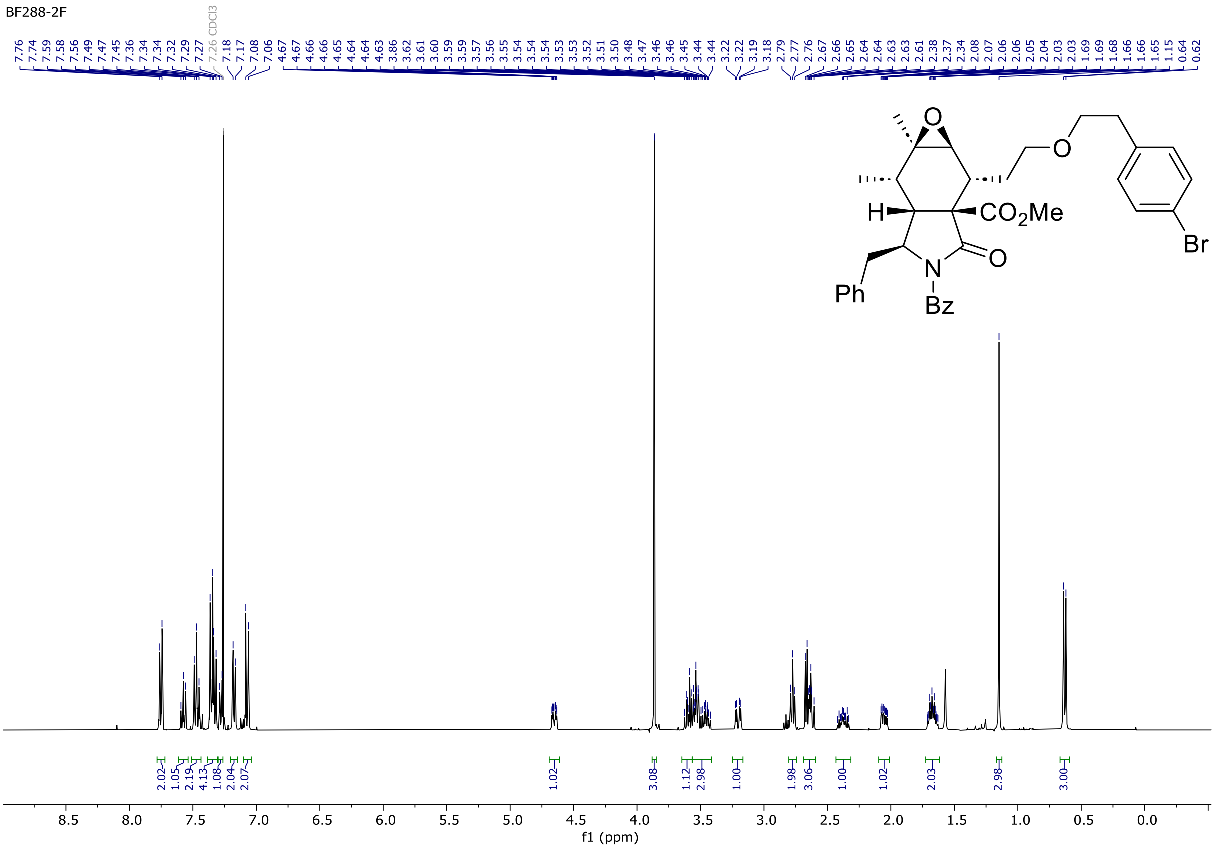
BF288-2F
7.76 7.74 7.59 7.58 7.56 7.49 7.47 7.45 7.36 7.34 7.34 7.32 7.29 7.27 7.26 CDCl3 7.18 7.17 7.08 7.06 4.67 4.67 4.66 4.66 4.65 4.64 4.64 4.63 3.86 3.62 3.61 3.60 3.59 3.59 3.57 3.56 3.55 3.54 3.54 3.54 3.53 3.53 3.52 3.51 3.50 3.48 3.47 3.46 3.46 3.45 3.44 3.44 3.22 3.22 3.19 3.18 2.79 2.77 2.76 2.67 2.66 2.65 2.64 2.64 2.63 2.61 2.38 2.37 2.34 2.08 2.07 2.06 2.06 2.05 2.04 2.03 2.03 1.69 1.69 1.68 1.66 1.66 1.65 1.15 0.64 0.62
O
CO2Me
H
Br
O
N
Ph
Bz
2.02 1.05 2.19 4.13 1.08 2.04 2.07 1.02 3.08 1.12 2.98 1.00 1.98 3.06 1.00 1.02 2.03 2.98 3.00
8.5 8.0 7.5 7.0 6.5 6.0 5.5 5.0 4.5 4.0 3.5 3.0 2.5 2.0 1.5 1.0 0.5 0.0
f1 (ppm)

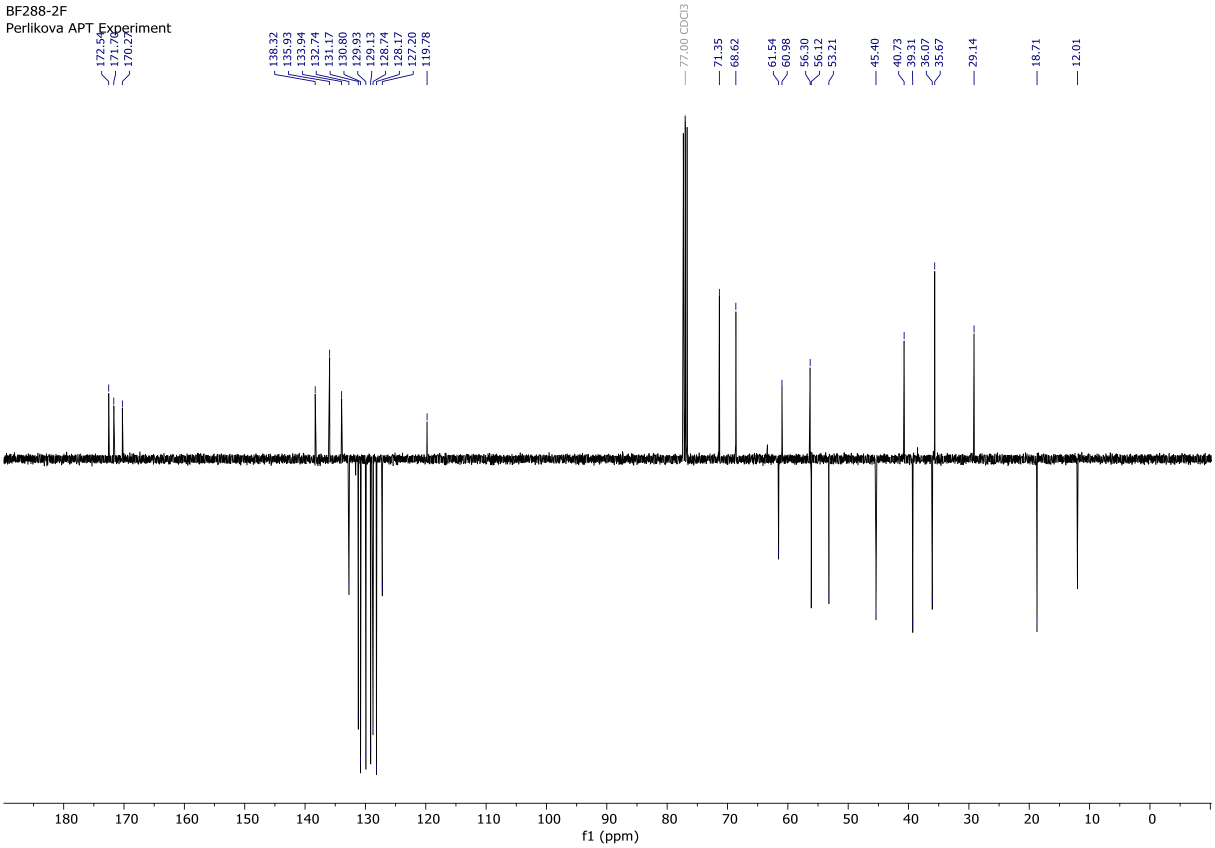
BF288-2F
Perlikova APT Experiment
172.54 171.76 170.27 138.32 135.93 133.94 132.74 131.17 130.80 129.93 129.13 128.74 128.17 127.20 119.78 77.00 CDCl3 71.35 68.62 61.54 60.98 56.30 56.12 53.21 45.40 40.73 39.31 36.07 35.67 29.14 18.71 12.01
180 170 160 150 140 130 120 110 100 90 80 70 60 50 40 30 20 10 0
f1 (ppm)

22a

BF141.1.fid

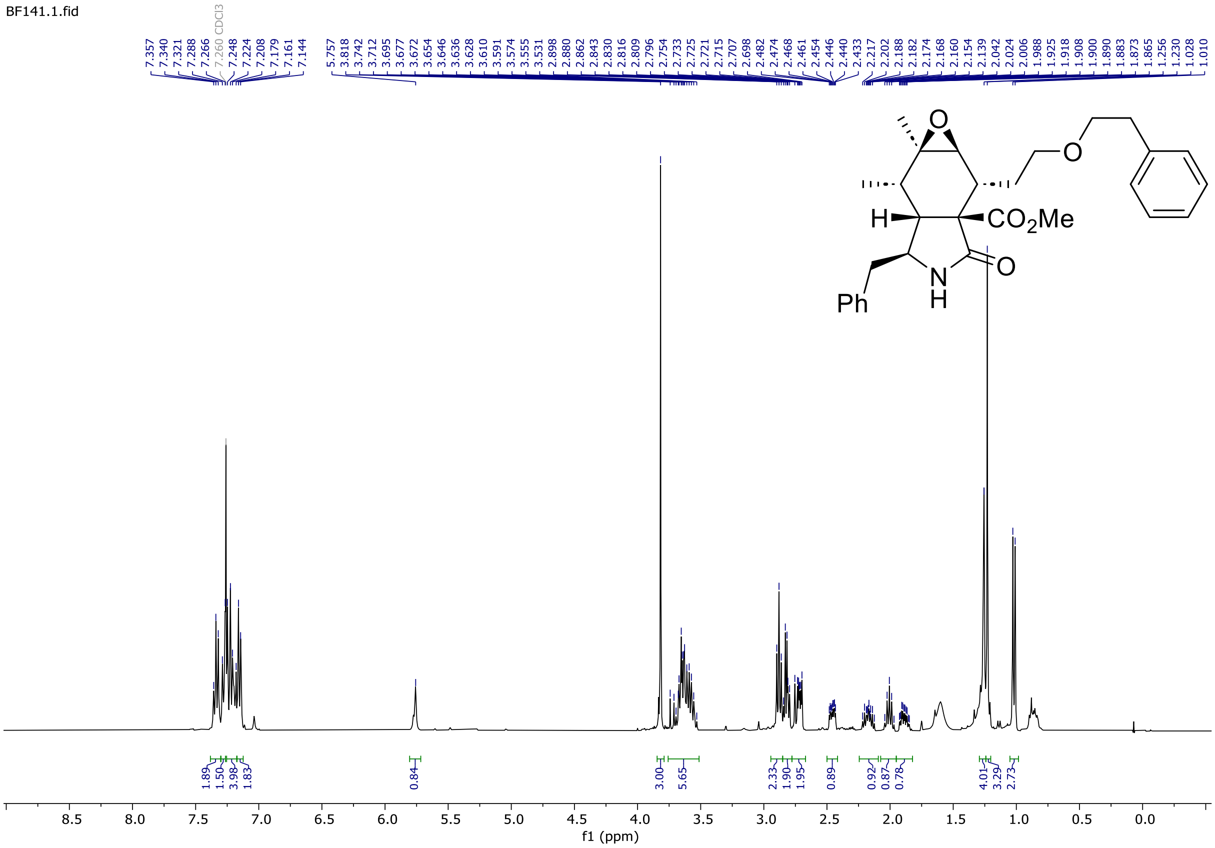

BF141.2.fid

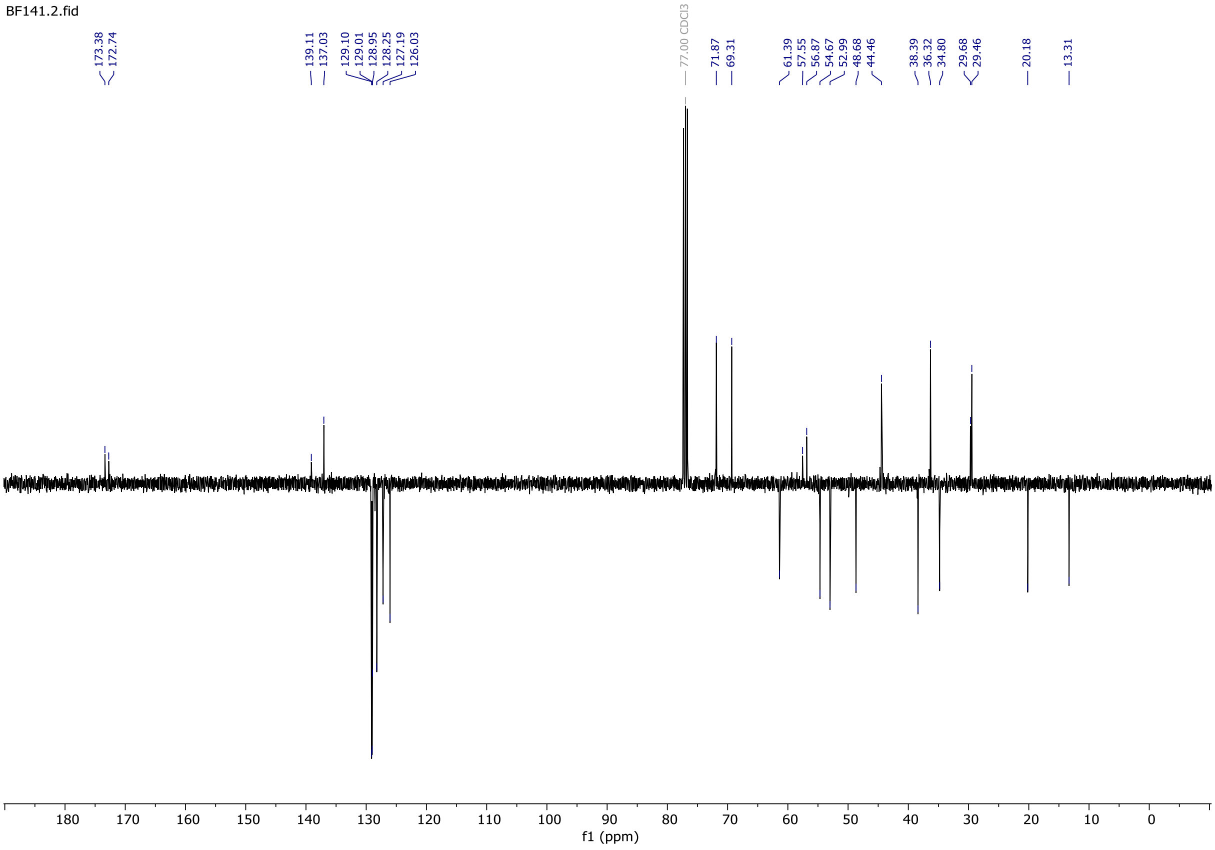

22b

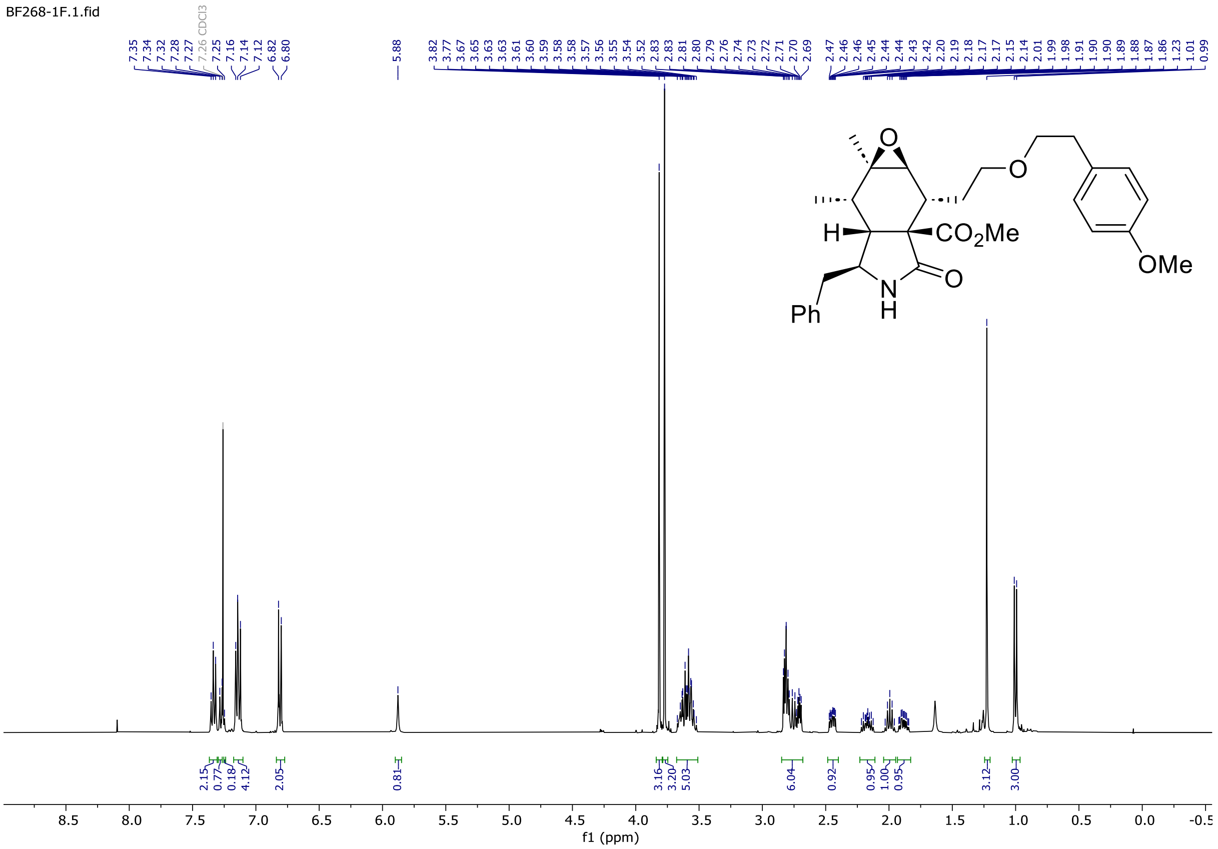

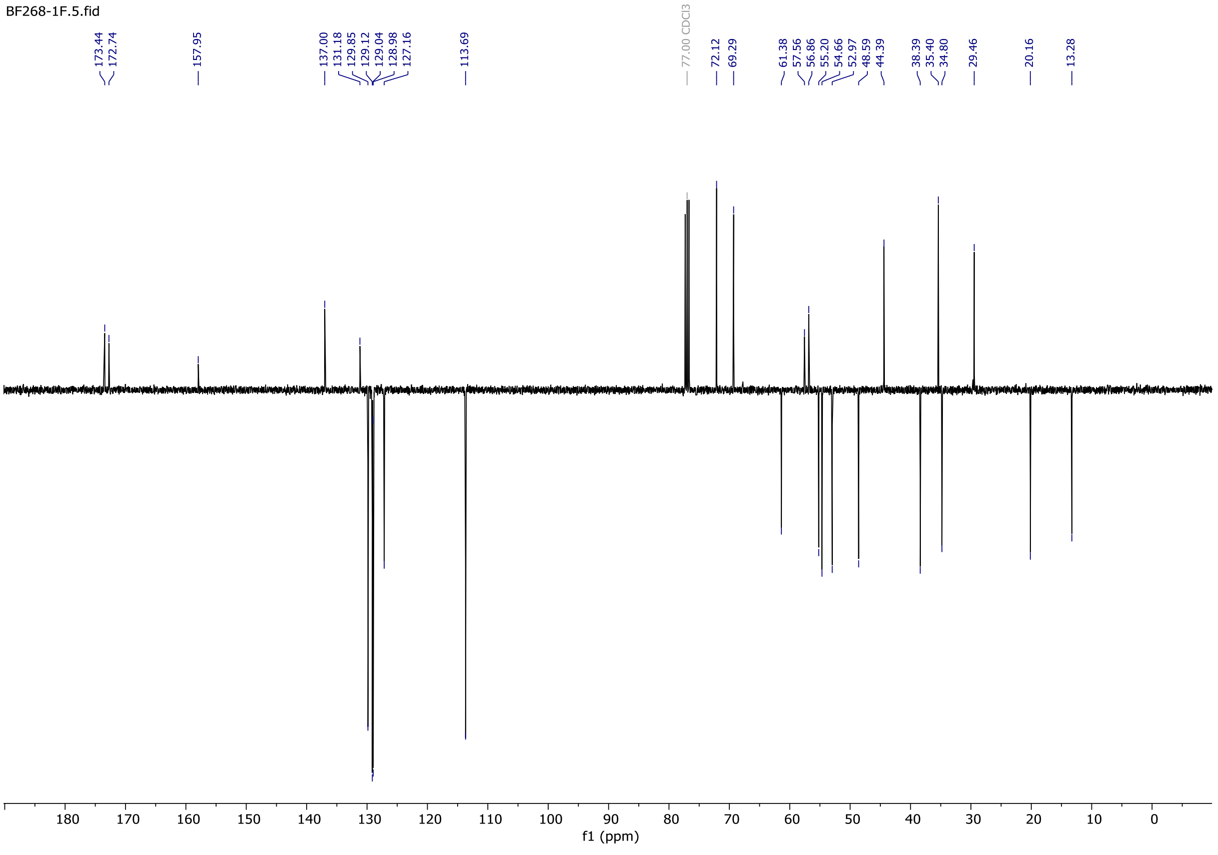

22h

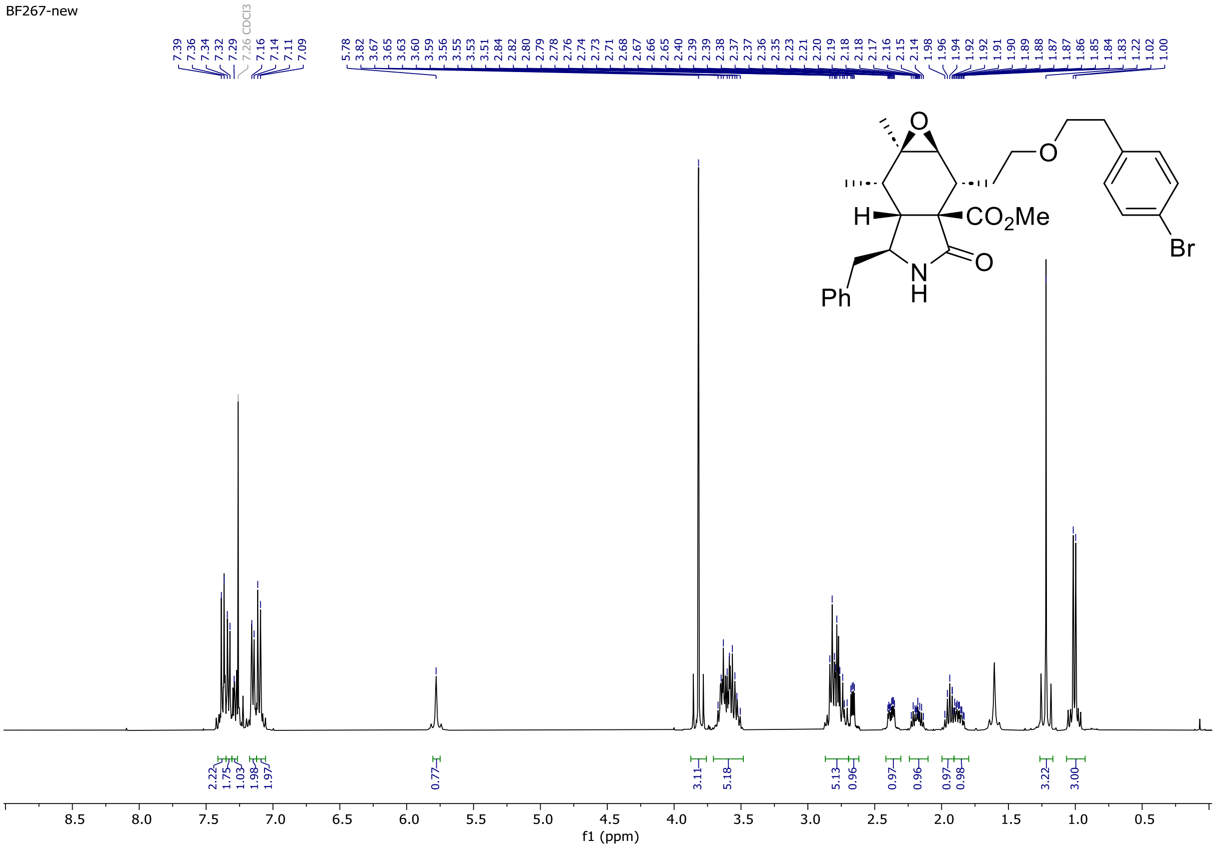

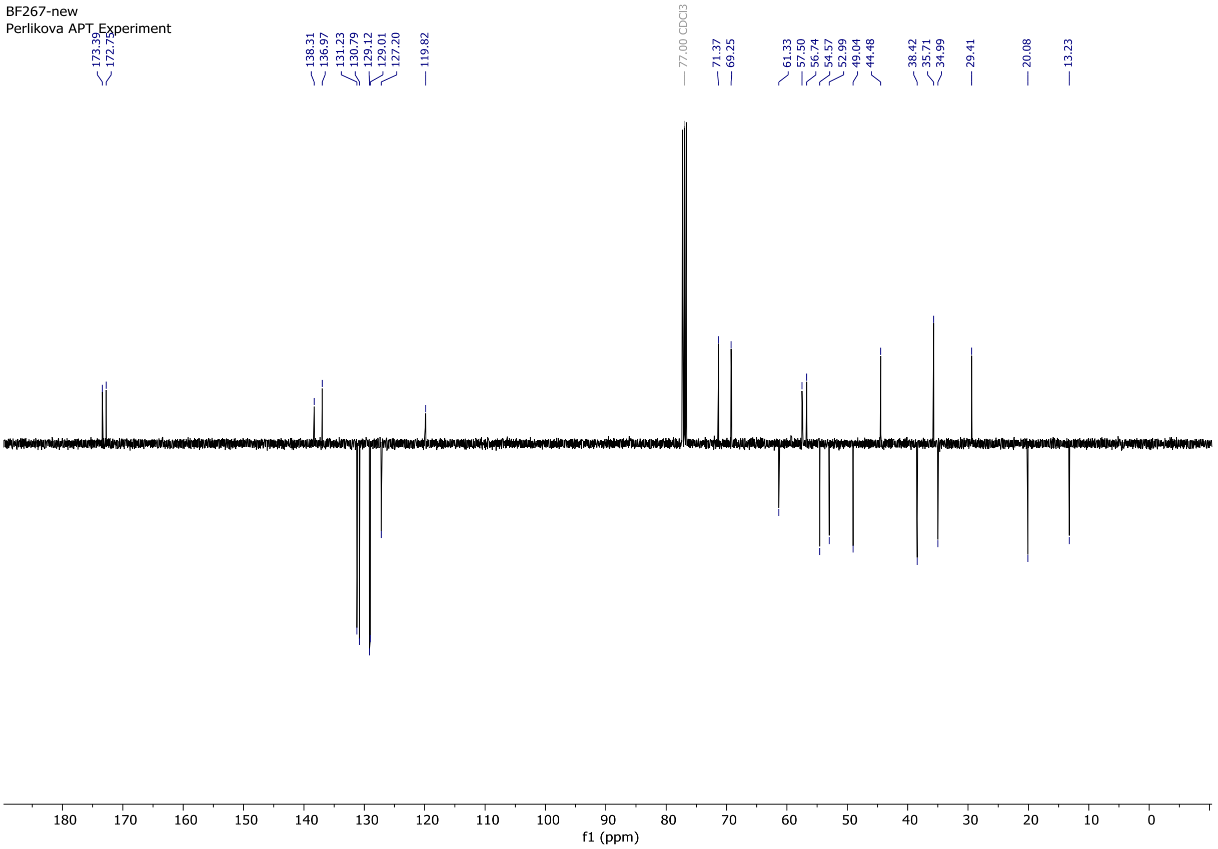

23a

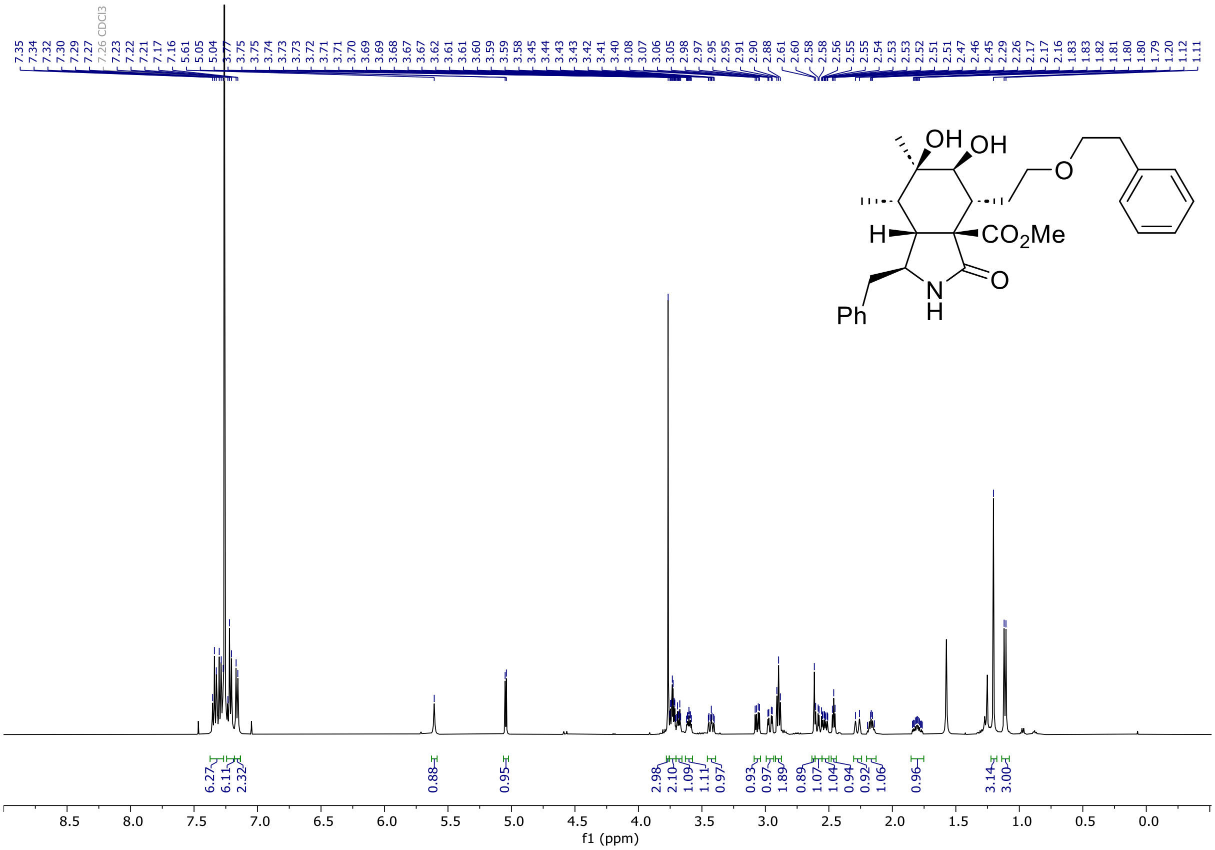
7.35 7.34 7.32 7.30 7.29 7.27 7.26 CDCl3 7.23 7.22 7.21 7.17 7.16 5.61 5.05 5.04 3.77 3.75 3.75 3.74 3.73 3.73 3.72 3.71 3.71 3.70 3.69 3.69 3.68 3.67 3.67 3.62 3.61 3.61 3.60 3.59 3.59 3.58 3.45 3.44 3.43 3.43 3.42 3.41 3.40 3.08 3.07 3.06 3.05 2.98 2.97 2.95 2.95 2.91 2.90 2.88 2.61 2.60 2.58 2.56 2.55 2.55 2.54 2.53 2.53 2.52 2.51 2.51 2.47 2.46 2.45 2.29 2.26 2.17 2.16 1.83 1.83 1.82 1.81 1.80 1.79 1.20 1.12 1.11
OH
OH
O
H
CO2Me
O
N
H
Ph
6.27 6.11 2.32 0.88 0.95 2.98 2.10 1.09 1.11 0.97 0.93 0.97 1.89 0.89 1.07 1.04 0.94 0.92 1.06 0.96 3.14 3.00
8.5 8.0 7.5 7.0 6.5 6.0 5.5 5.0 4.5 4.0 3.5 3.0 2.5 2.0 1.5 1.0 0.5 0.0
f1 (ppm)

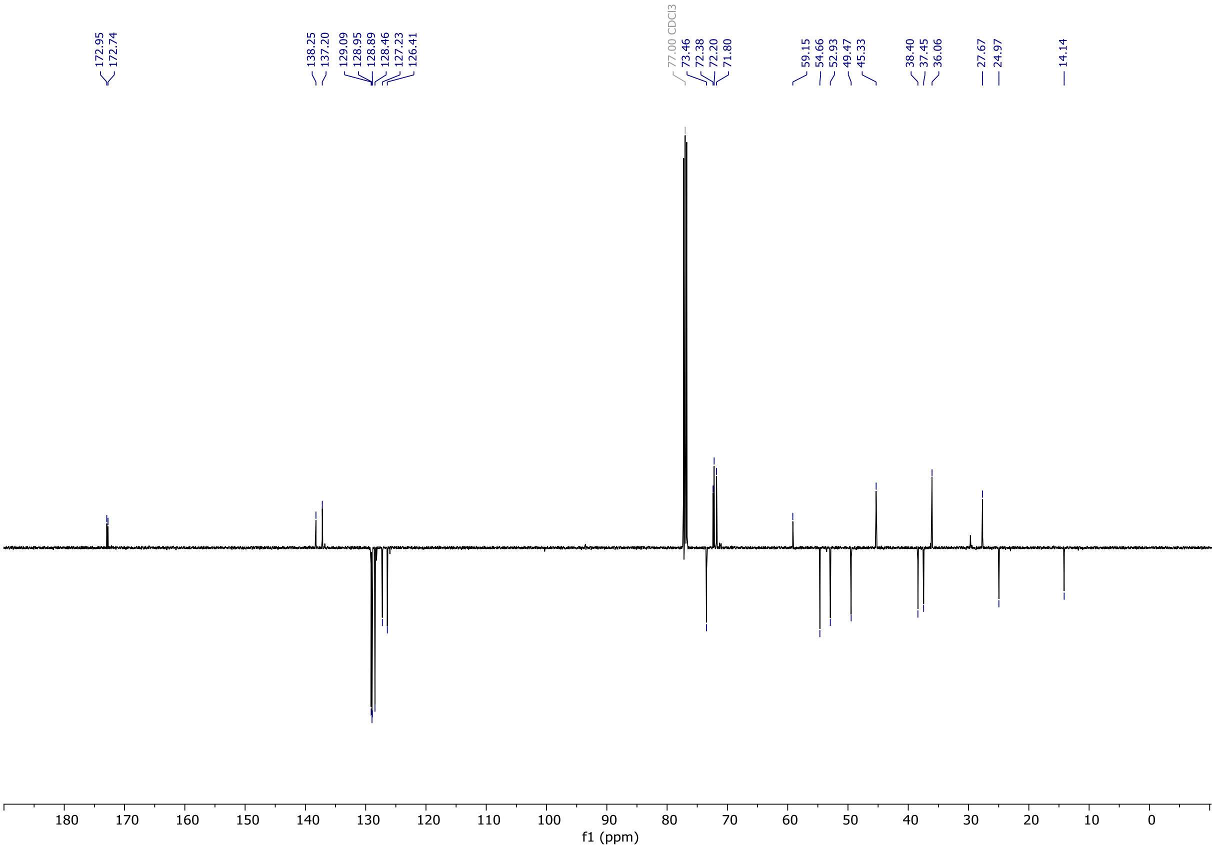
172.95 172.74 138.25 137.20 129.09 128.95 128.89 128.46 127.23 126.41 77.00 CDCl3 73.46 72.38 72.20 71.80 59.15 54.66 52.93 49.47 45.33 38.40 37.45 36.06 27.67 24.97 14.14
180 170 160 150 140 130 120 110 100 90 80 70 60 50 40 30 20 10 0
f1 (ppm)

23b

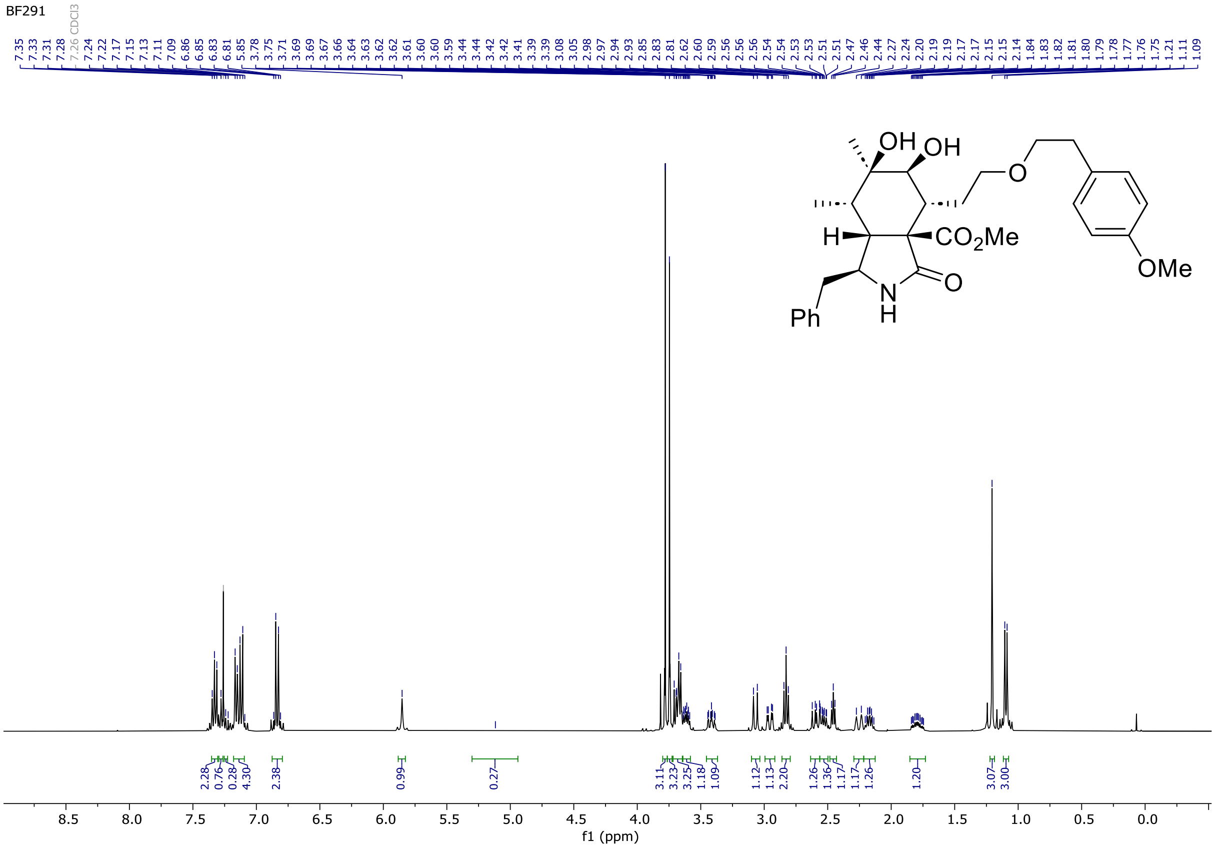
BF291
CDCl3
7.35 7.33 7.31 7.28 7.26 7.24 7.22 7.17 7.15 7.13 7.11 7.09 6.86 6.85 6.83 6.81 5.85 3.78 3.75 3.71 3.69 3.69 3.67 3.66 3.64 3.63 3.62 3.62 3.61 3.60 3.60 3.59 3.44 3.44 3.42 3.42 3.41 3.39 3.39 3.08 3.05 2.98 2.97 2.94 2.93 2.85 2.83 2.81 2.62 2.60 2.59 2.56 2.56 2.56 2.54 2.54 2.53 2.53 2.51 2.51 2.47 2.46 2.44 2.27 2.24 2.20 2.19 2.19 2.17 2.17 2.15 2.15 2.14 1.84 1.83 1.82 1.81 1.80 1.79 1.78 1.77 1.76 1.75 1.21 1.11 1.09
OH
OH
O
H
CO2Me
OMe
N
H
O
Ph
2.28 0.76 0.28 4.30 2.38 0.99 0.27 3.11 3.23 3.25 1.18 1.09 1.12 1.13 2.20 1.26 1.36 1.17 1.17 1.26 1.20 3.07 3.00
8.5 8.0 7.5 7.0 6.5 6.0 5.5 5.0 4.5 4.0 3.5 3.0 2.5 2.0 1.5 1.0 0.5 0.0
f1 (ppm)

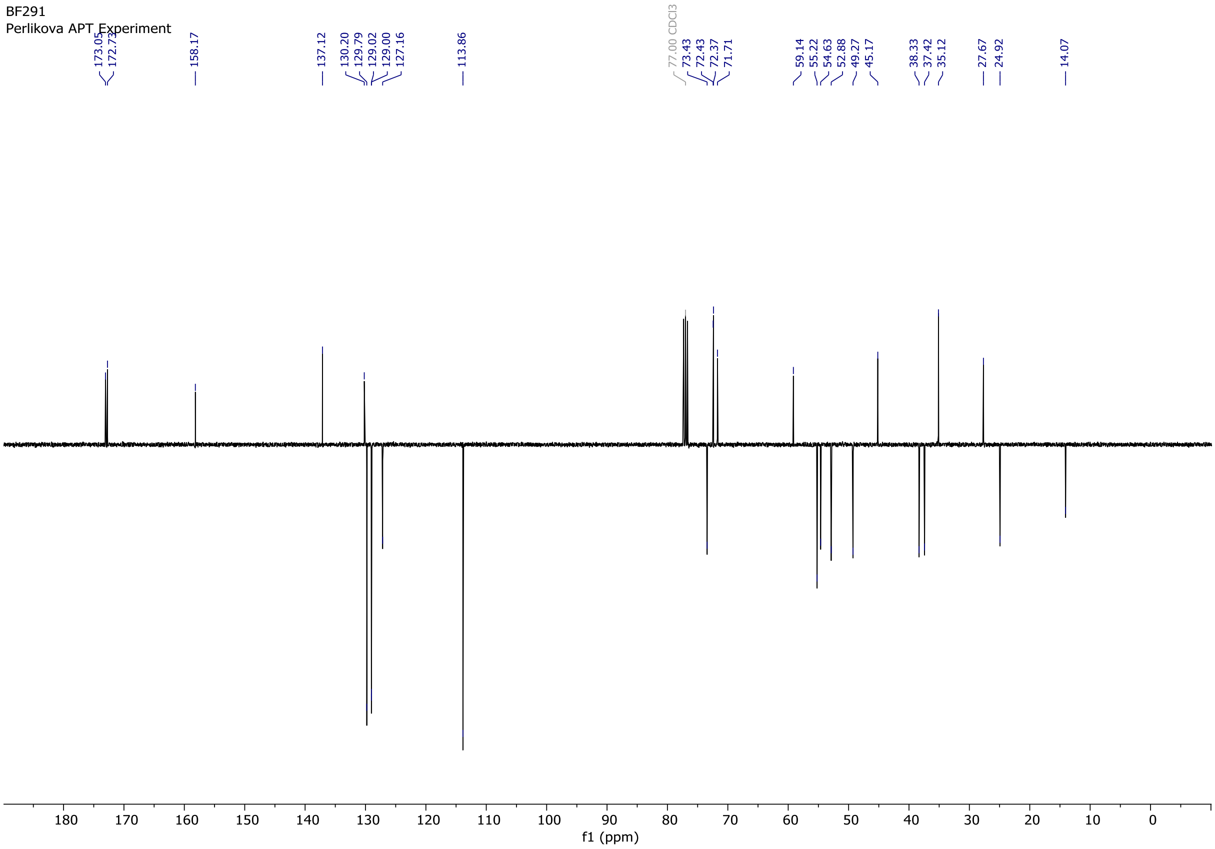
BF291
Perlikova APT Experiment
173.05 172.72 158.17 137.12 130.20 129.79 129.02 129.00 127.16 113.86 77.00 CDCl3 73.43 72.43 72.37 71.71 59.14 55.22 54.63 52.88 49.27 45.17 38.33 37.42 35.12 27.67 24.92 14.07
180 170 160 150 140 130 120 110 100 90 80 70 60 50 40 30 20 10 0
f1 (ppm)

23h

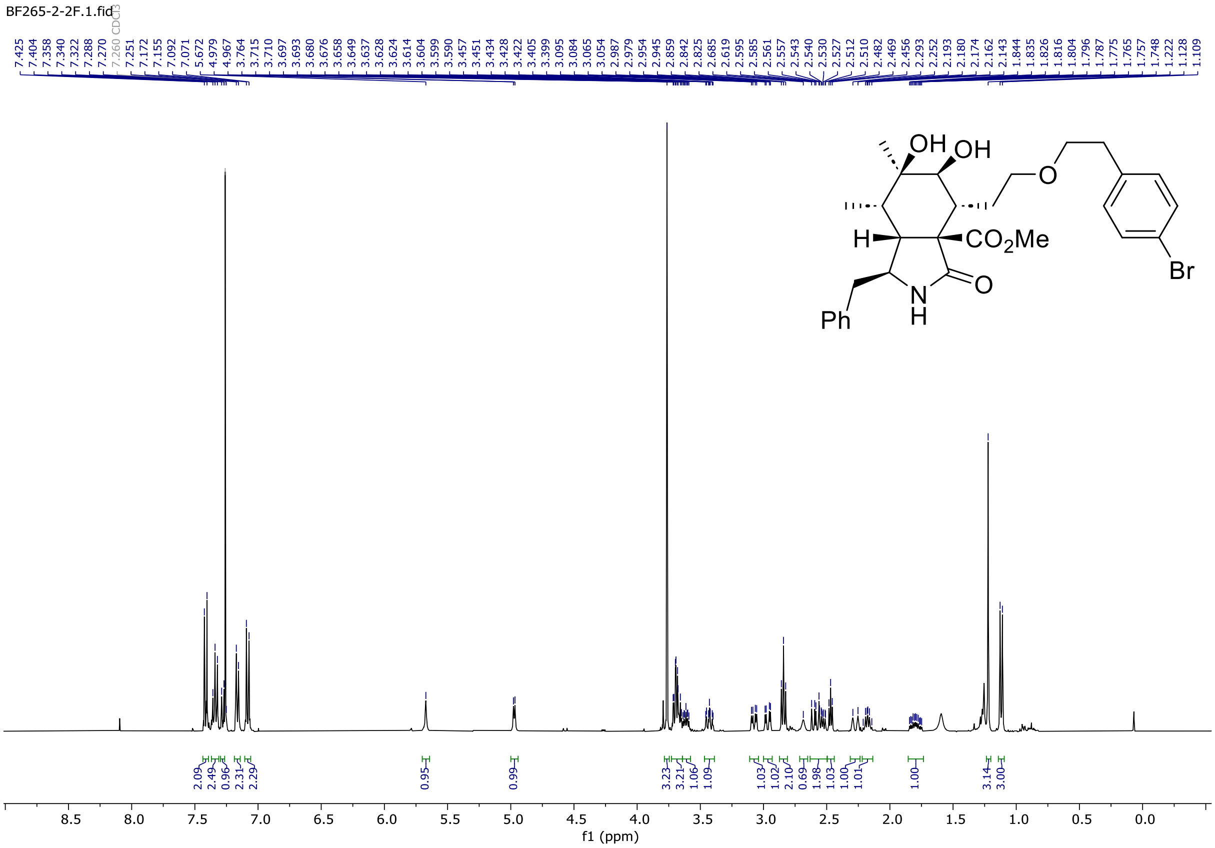
BF265-2-2F.1.fid
7.260 CDCl3
7.425 7.404 7.358 7.340 7.322 7.288 7.270 7.251 7.172 7.155 7.092 7.071 5.672 4.979 4.967 3.764 3.715 3.710 3.697 3.693 3.680 3.676 3.658 3.649 3.637 3.628 3.624 3.614 3.604 3.599 3.590 3.457 3.451 3.434 3.428 3.422 3.405 3.399 3.095 3.084 3.065 3.054 2.987 2.979 2.954 2.945 2.859 2.842 2.825 2.685 2.619 2.595 2.585 2.561 2.557 2.543 2.540 2.530 2.527 2.512 2.510 2.482 2.469 2.456 2.293 2.252 2.193 2.180 2.174 2.162 2.143 1.844 1.835 1.826 1.816 1.804 1.796 1.787 1.775 1.765 1.757 1.748 1.222 1.128 1.109
OH
OH
O
H
CO2Me
Br
Ph
N
H
O
2.09 2.49 0.96 2.31 2.29 0.95 0.99 3.23 3.21 1.06 1.09 1.03 1.02 2.10 0.69 1.98 1.03 1.00 1.01 1.00 3.14 3.00
8.5 8.0 7.5 7.0 6.5 6.0 5.5 5.0 4.5 4.0 3.5 3.0 2.5 2.0 1.5 1.0 0.5 0.0
f1 (ppm)

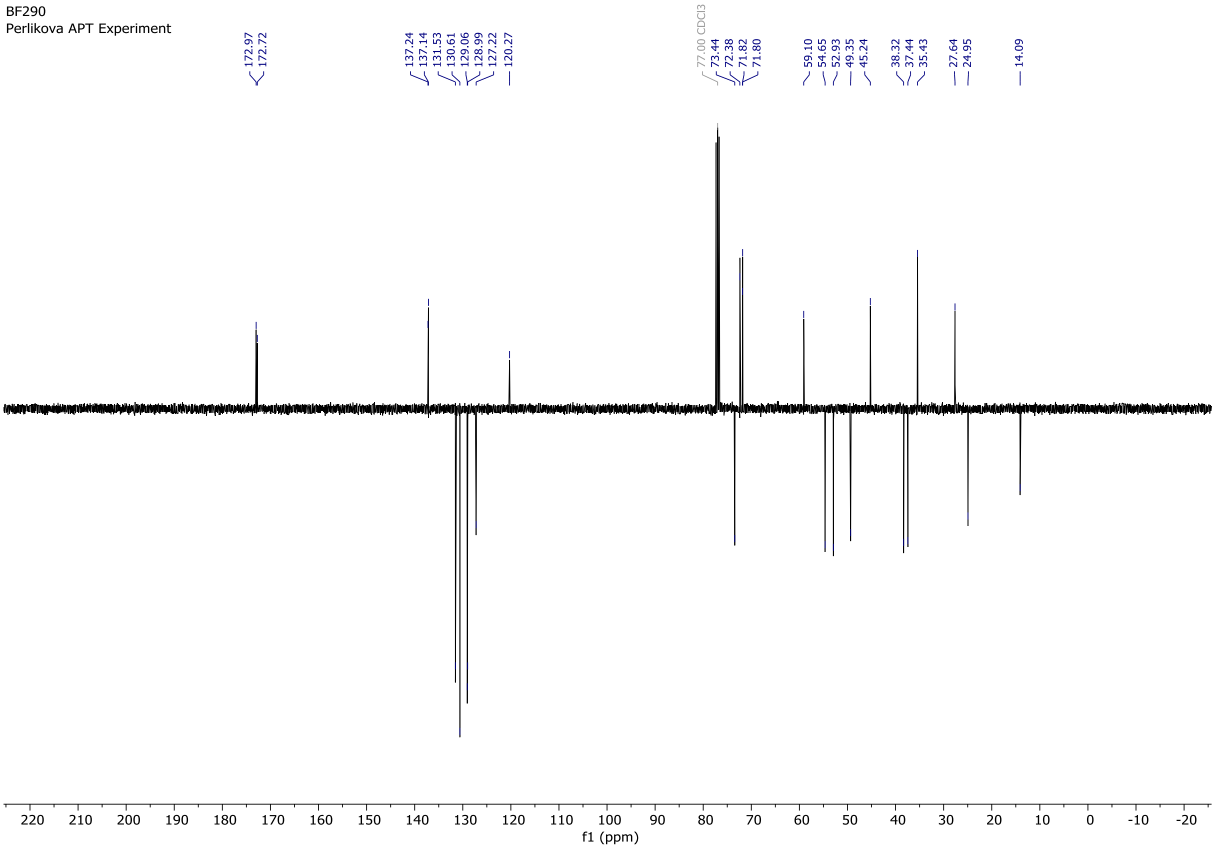
BF290
Perlikova APT Experiment
172.97 172.72
137.24 137.14 131.53 130.61 129.06 128.99 127.22 120.27
77.00 CDCl3
73.44 72.38 71.82 71.80
59.10 54.65 52.93 49.35 45.24
38.32 37.44 35.43
27.64 24.95
14.09
220 210 200 190 180 170 160 150 140 130 120 110 100 90 80 70 60 50 40 30 20 10 0 -10 -20
f1 (ppm)

24

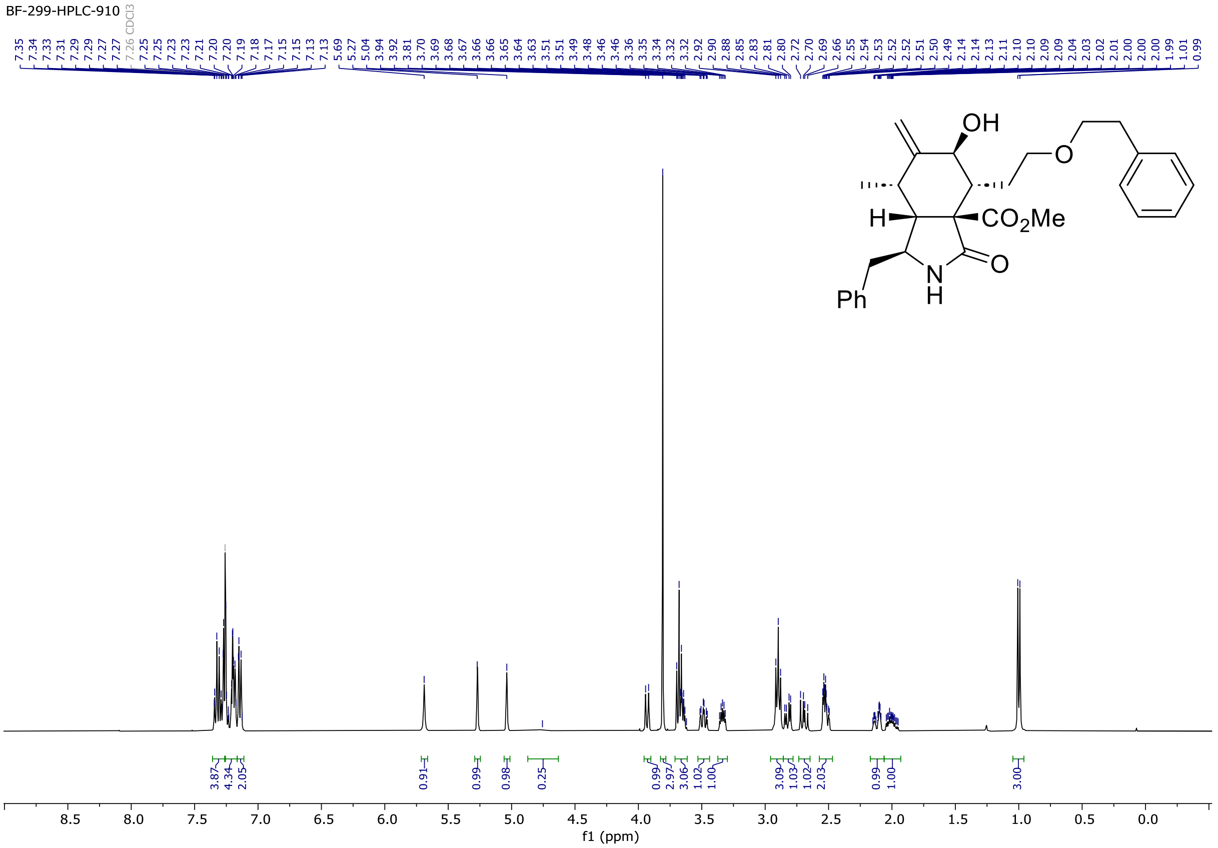
BF-299-HPLC-910
7.35 7.34 7.33 7.31 7.29 7.29 7.27 7.27 7.26 CDCl3 7.25 7.25 7.23 7.23 7.21 7.20 7.20 7.19 7.18 7.17 7.15 7.15 7.13 7.13 5.69 5.27 5.04 3.94 3.92 3.81 3.70 3.69 3.68 3.67 3.66 3.66 3.65 3.64 3.63 3.51 3.51 3.49 3.48 3.46 3.46 3.36 3.35 3.34 3.32 3.32 2.92 2.90 2.88 2.85 2.83 2.81 2.80 2.72 2.70 2.69 2.66 2.55 2.54 2.53 2.52 2.52 2.51 2.50 2.49 2.14 2.14 2.13 2.11 2.10 2.10 2.09 2.09 2.04 2.03 2.02 2.01 2.00 2.00 2.00 1.99 1.01 0.99
OH
O
H
CO2Me
O
N
H
Ph
3.87 4.34 2.05 0.91 0.99 0.98 0.25 0.99 2.97 3.06 1.02 1.00 3.09 1.03 1.02 2.03 0.99 1.00 3.00
8.5 8.0 7.5 7.0 6.5 6.0 5.5 5.0 4.5 4.0 3.5 3.0 2.5 2.0 1.5 1.0 0.5 0.0
f1 (ppm)

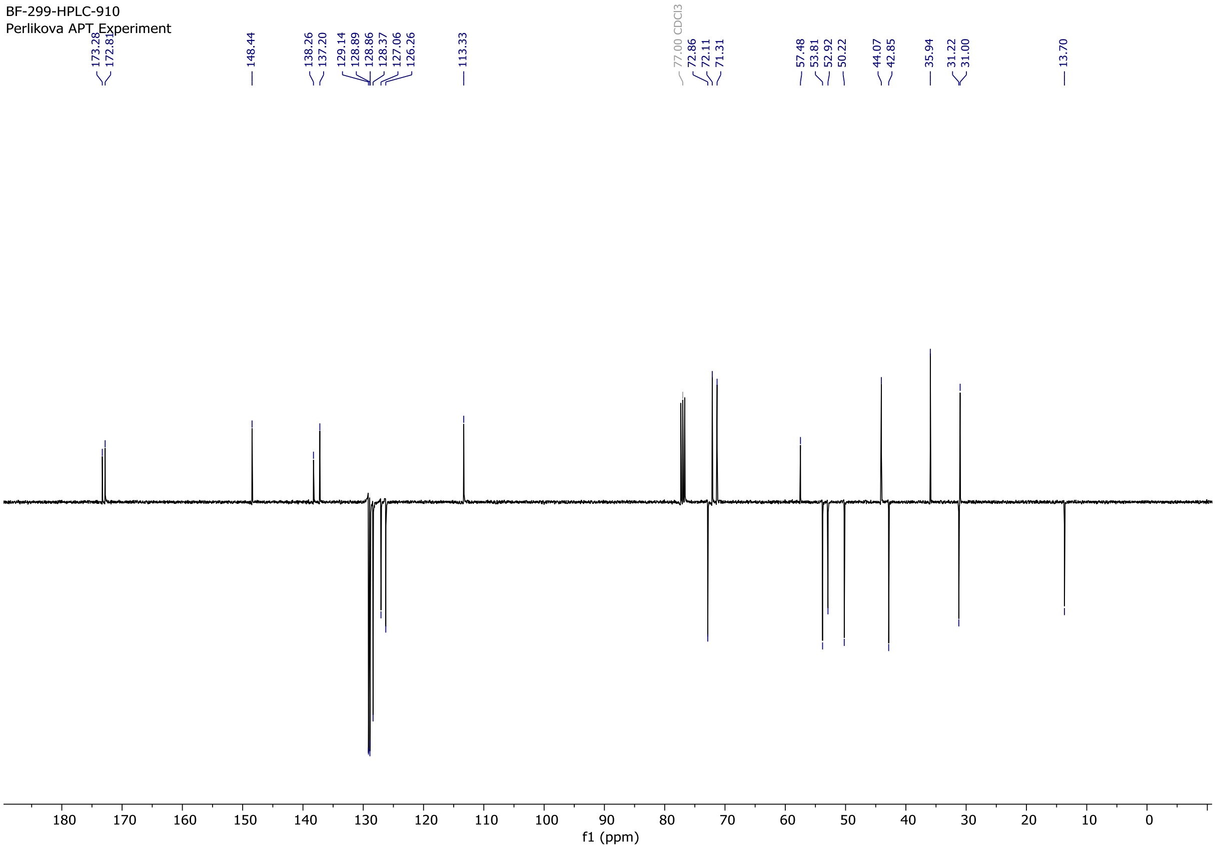
BF-299-HPLC-910
Perlikova APT Experiment
173.28 172.81 148.44 138.26 137.20 129.14 128.89 128.86 128.37 127.06 126.26 113.33 77.00 CDCl3 72.86 72.11 71.31 57.48 53.81 52.92 50.22 44.07 42.85 35.94 31.22 31.00 13.70
180 170 160 150 140 130 120 110 100 90 80 70 60 50 40 30 20 10 0
f1 (ppm)

25

BF-22-365_HPLC-f8

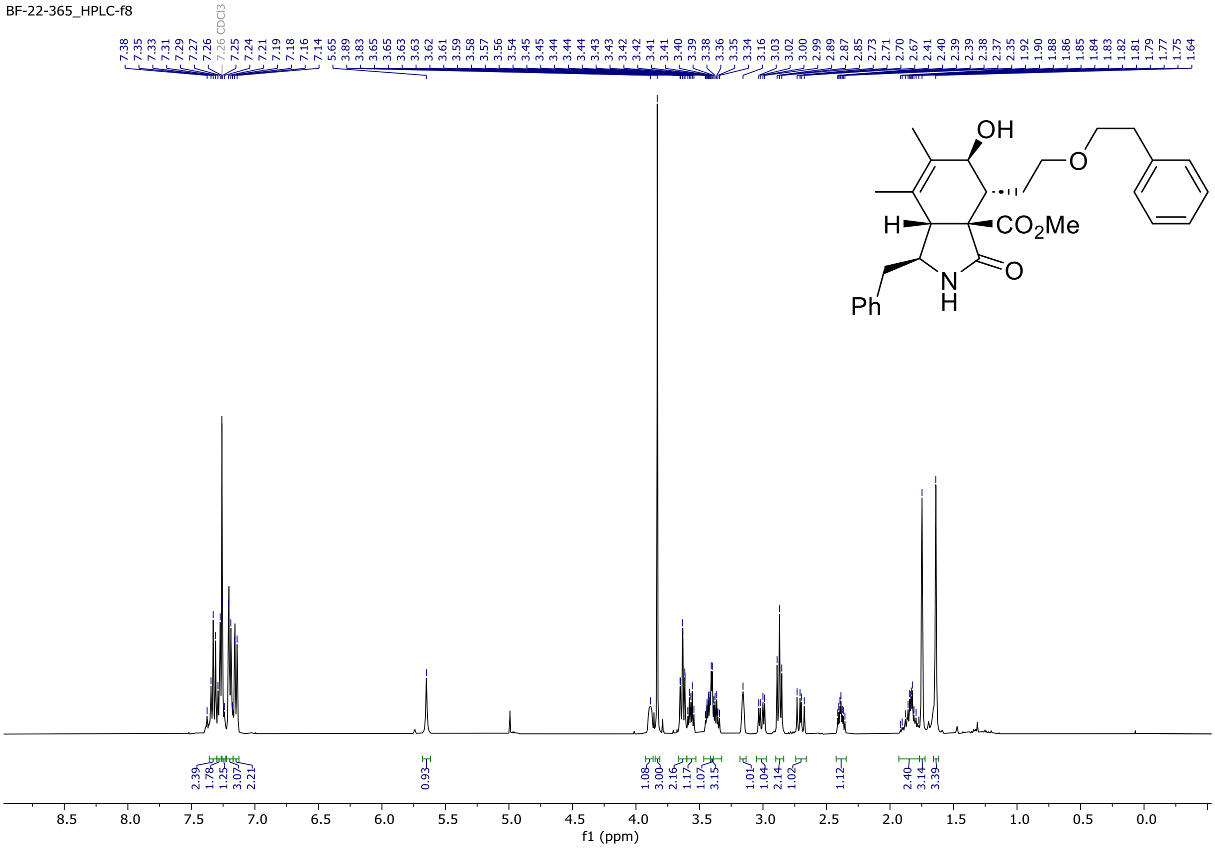

BF-22-365_HPLC-f8-APT

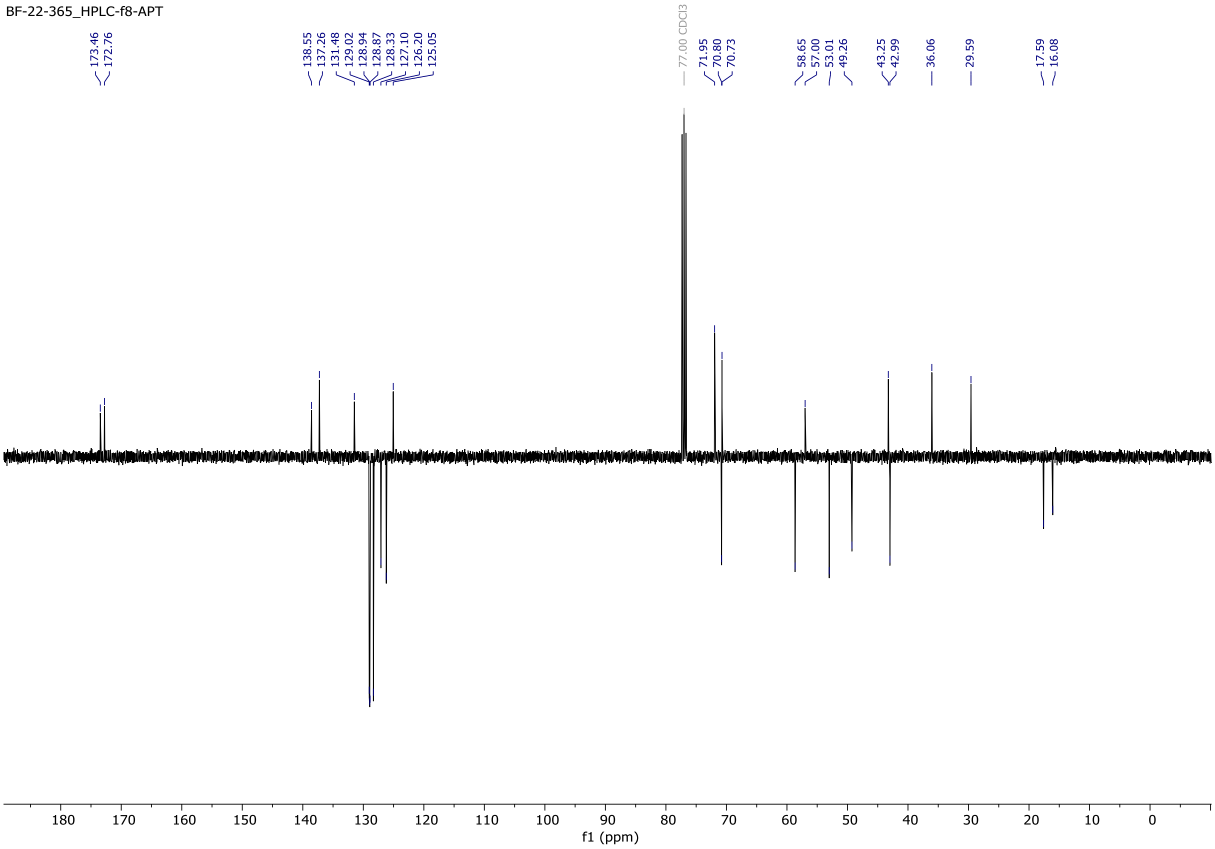

26

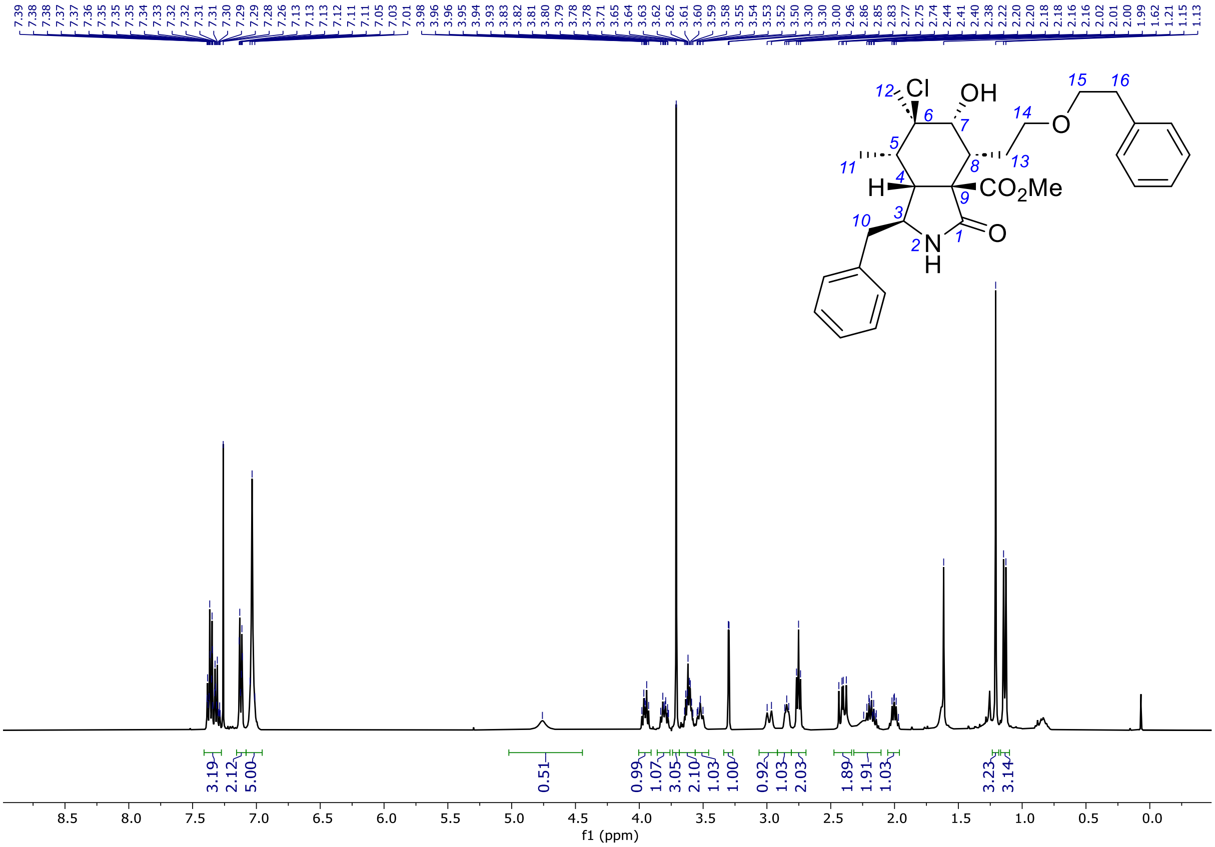

7.39 7.38 7.38 7.37 7.37 7.36 7.35 7.35 7.35 7.34 7.33 7.32 7.32 7.31 7.31 7.30 7.29 7.29 7.28 7.26 7.13 7.13 7.13 7.13 7.12 7.11 7.11 7.05 7.03 7.01 3.98 3.96 3.95 3.94 3.93 3.83 3.82 3.81 3.80 3.79 3.78 3.78 3.71 3.65 3.64 3.63 3.62 3.62 3.61 3.60 3.59 3.58 3.55 3.54 3.53 3.52 3.50 3.30 3.30 3.00 2.96 2.86 2.85 2.83 2.77 2.75 2.74 2.44 2.41 2.40 2.38 2.22 2.20 2.18 2.16 2.16 2.02 2.01 2.00 1.99 1.62 1.21 1.15 1.13
Cl
OH
O
H
CO_2Me
N
H
O
3.19 2.12 5.00 0.51 0.99 1.07 3.05 2.10 1.03 1.00 0.92 1.03 2.03 1.89 1.91 1.03 3.23 3.14
f1 (ppm)

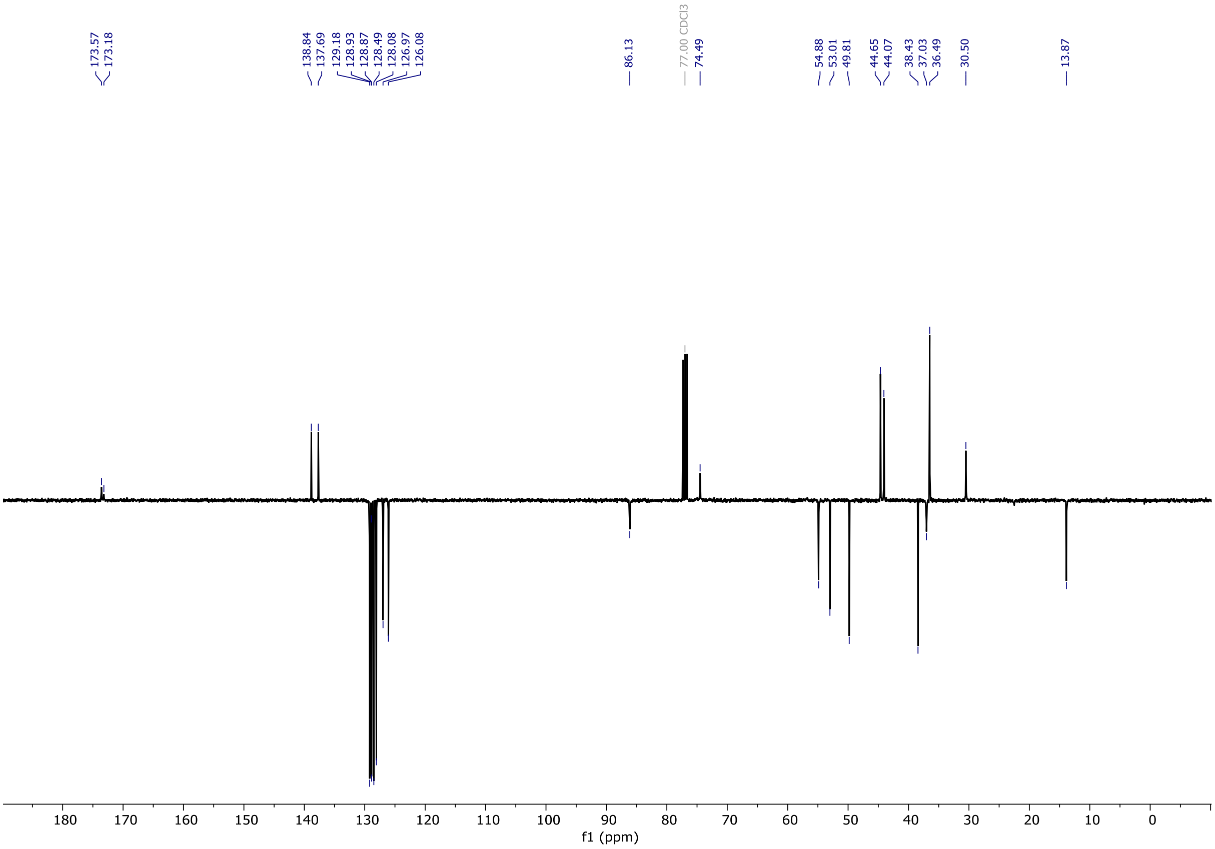

173.57 173.18 138.84 137.69 129.18 128.93 128.87 128.49 128.08 126.97 126.08 86.13 77.00 CDCl3 74.49 54.88 53.01 49.81 44.65 44.07 38.43 37.03 36.49 30.50 13.87
f1 (ppm)

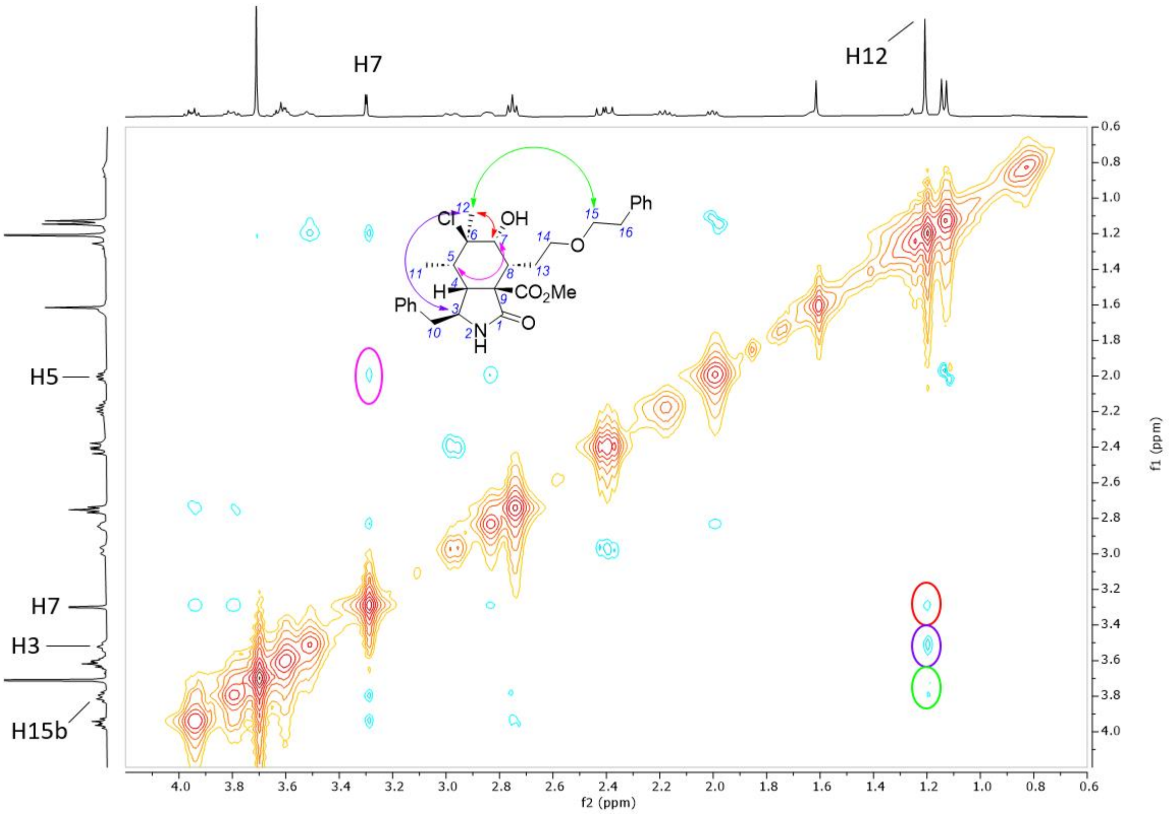
H7
H12
H5
H7
H3
H15b
f2 (ppm)
f1 (ppm)

27

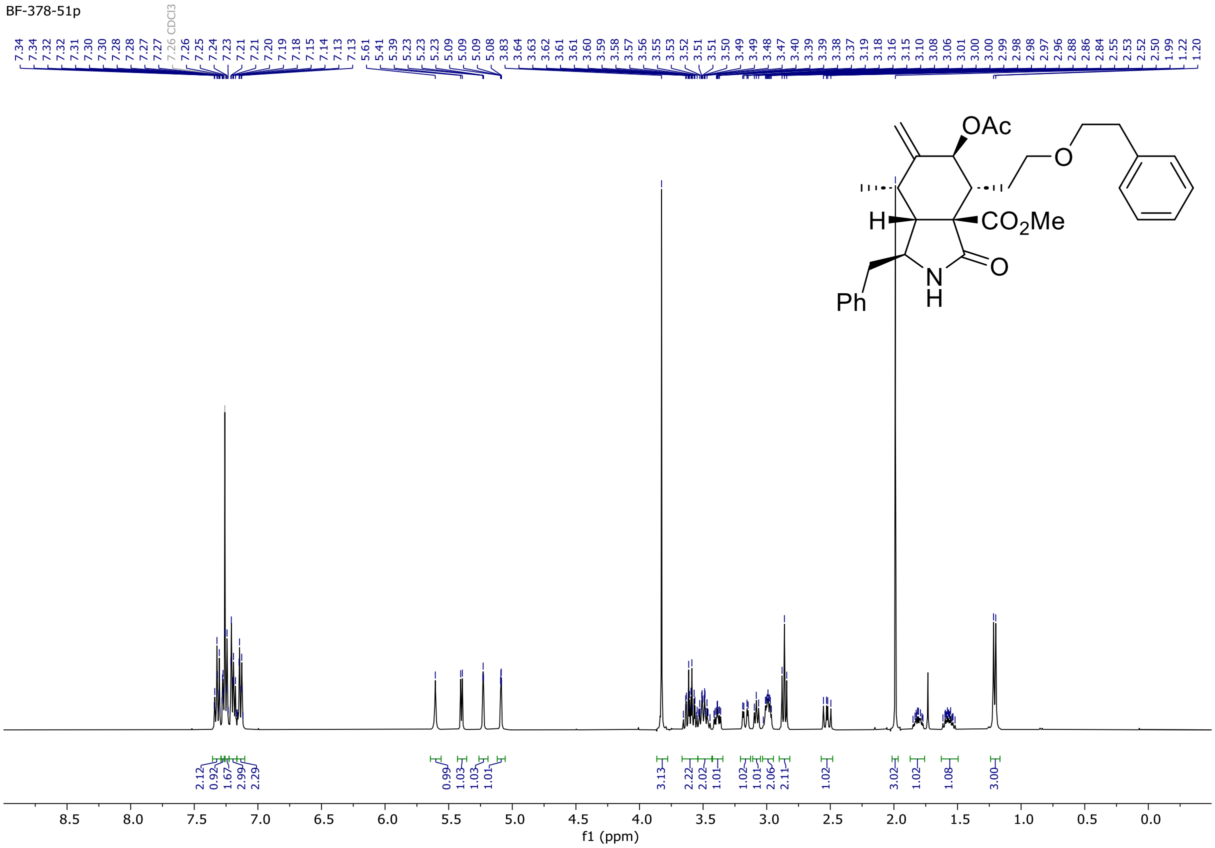
BF-378-51p
7.34 7.34 7.32 7.32 7.31 7.30 7.30 7.28 7.28 7.27 7.27 7.26 CDCl3 7.26 7.25 7.24 7.23 7.21 7.21 7.20 7.19 7.18 7.15 7.14 7.13 7.13 5.61 5.41 5.39 5.23 5.23 5.23 5.09 5.09 5.09 5.08 3.83 3.64 3.63 3.62 3.61 3.61 3.60 3.59 3.58 3.57 3.56 3.55 3.53 3.52 3.51 3.51 3.50 3.49 3.49 3.48 3.47 3.40 3.39 3.39 3.38 3.37 3.19 3.18 3.16 3.15 3.10 3.08 3.06 3.01 3.00 3.00 2.99 2.98 2.98 2.97 2.96 2.88 2.86 2.84 2.55 2.53 2.52 2.50 1.99 1.22 1.20
OAc
O
H
CO2Me
O
N
H
Ph
2.12 0.92 1.67 2.99 2.29 0.99 1.03 1.03 1.01 3.13 2.22 2.02 1.01 1.02 1.01 2.06 2.11 1.02 3.02 1.02 1.08 3.00
8.5 8.0 7.5 7.0 6.5 6.0 5.5 5.0 4.5 4.0 3.5 3.0 2.5 2.0 1.5 1.0 0.5 0.0
f1 (ppm)

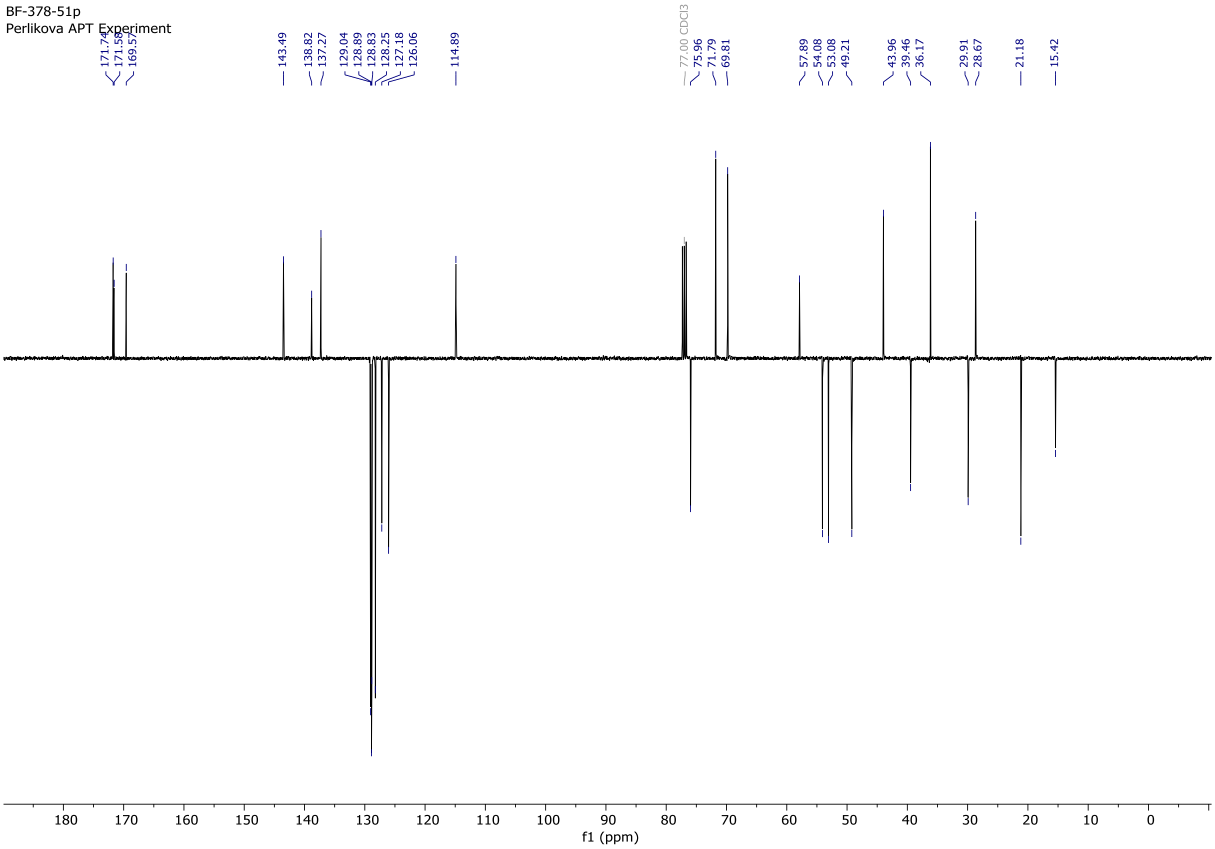
BF-378-51p
Perlikova APT Experiment
171.74 171.58 169.57 143.49 138.82 137.27 129.04 128.89 128.83 128.25 127.18 126.06 114.89 77.00 CDCl3 75.96 71.79 69.81 57.89 54.08 53.08 49.21 43.96 39.46 36.17 29.91 28.67 21.18 15.42
180 170 160 150 140 130 120 110 100 90 80 70 60 50 40 30 20 10 0
f1 (ppm)

28

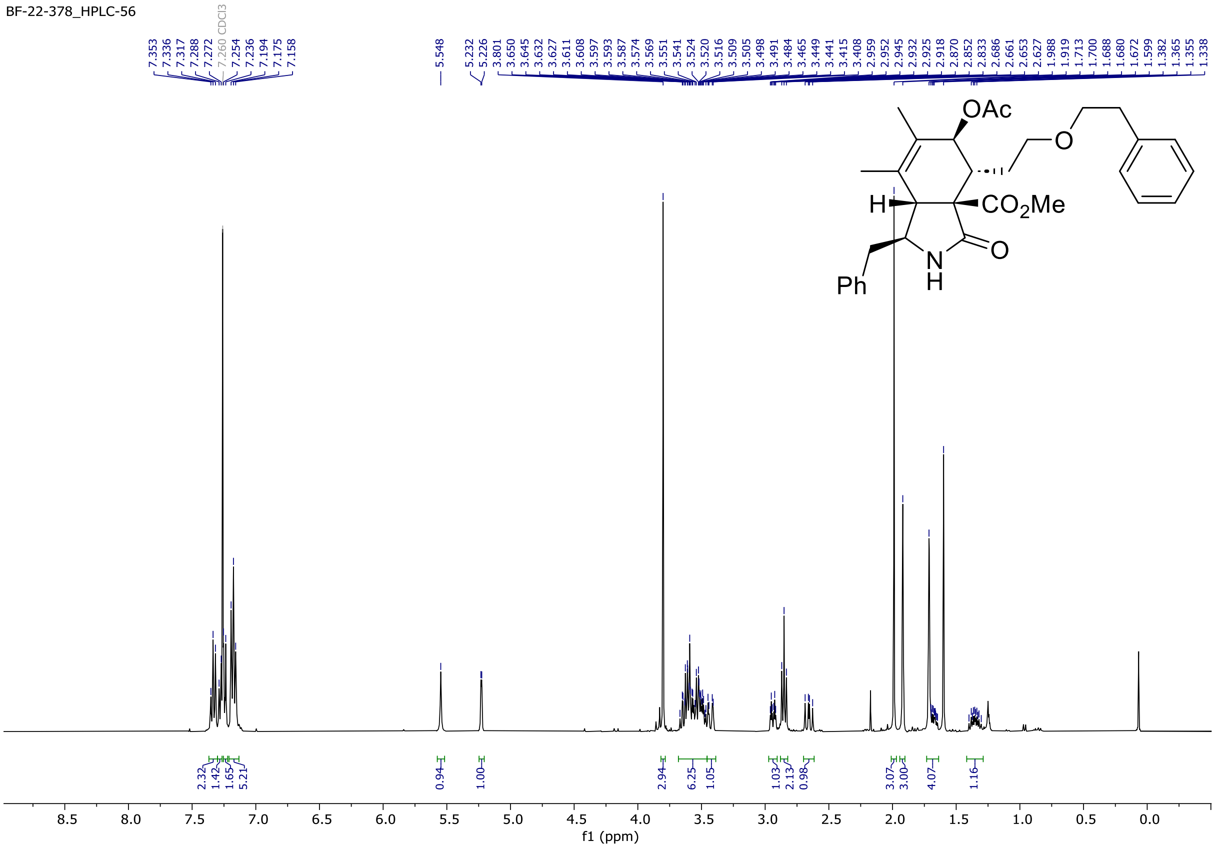

BF-22-378_HPLC-56
7.353
7.336
7.317
7.288
7.272
7.260 CDCl3
7.254
7.236
7.194
7.175
7.158
5.548
5.232
5.226
3.801
3.650
3.645
3.632
3.627
3.611
3.608
3.597
3.593
3.587
3.574
3.569
3.551
3.541
3.524
3.520
3.516
3.509
3.505
3.498
3.491
3.484
3.465
3.449
3.441
3.415
3.408
2.959
2.952
2.945
2.932
2.925
2.918
2.870
2.852
2.833
2.686
2.661
2.653
2.627
1.988
1.919
1.713
1.700
1.688
1.680
1.672
1.599
1.382
1.365
1.355
1.338
OAc
O
H
CO2Me
O
N
H
Ph
2.32
1.42
1.65
5.21
0.94
1.00
2.94
6.25
1.05
1.03
2.13
0.98
3.07
3.00
4.07
1.16
8.5
8.0
7.5
7.0
6.5
6.0
5.5
5.0
4.5
4.0
3.5
3.0
2.5
2.0
1.5
1.0
0.5
0.0
f1 (ppm)

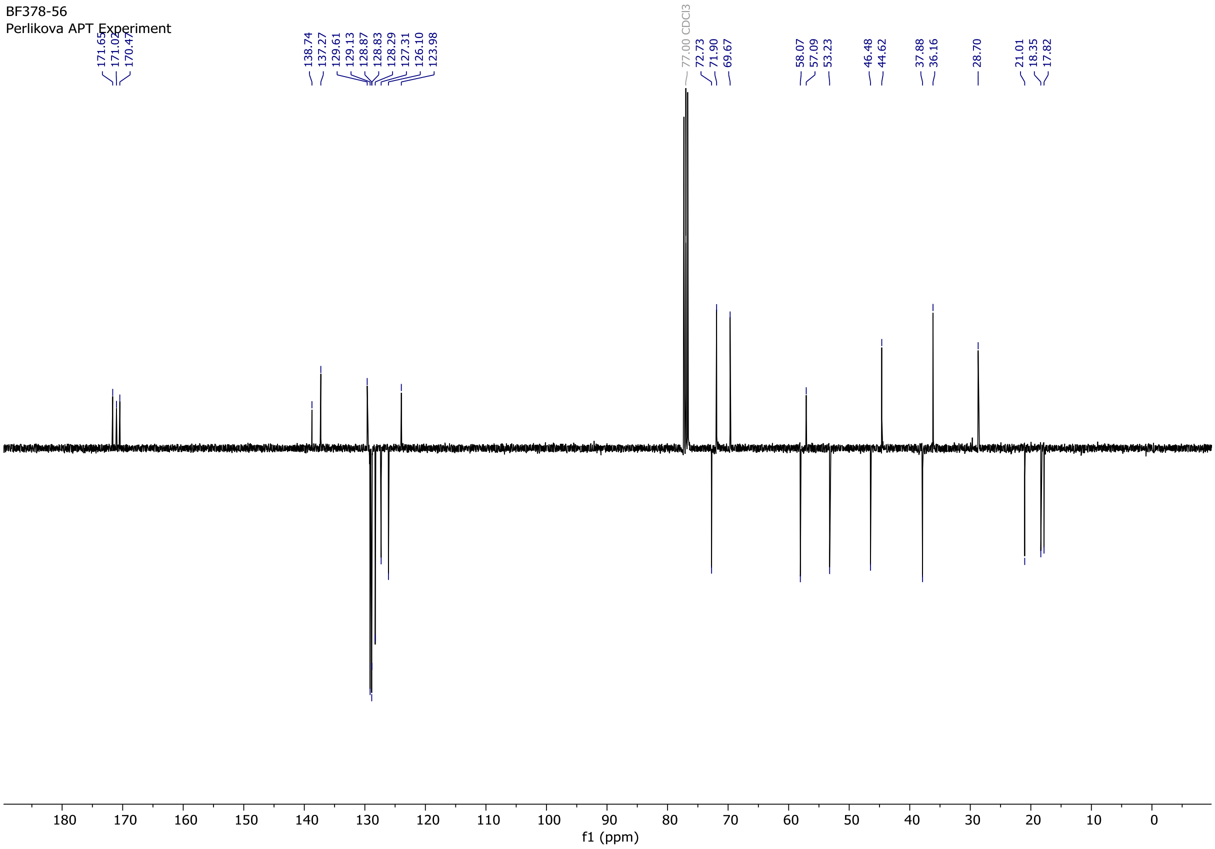

BF378-56
Perlikova APT Experiment
171.65
171.02
170.47
138.74
137.27
129.61
129.13
128.87
128.83
128.29
127.31
126.10
123.98
77.00 CDCl3
72.73
71.90
69.67
58.07
57.09
53.23
46.48
44.62
37.88
36.16
28.70
21.01
18.35
17.82
180
170
160
150
140
130
120
110
100
90
80
70
60
50
40
30
20
10
0
f1 (ppm)

29

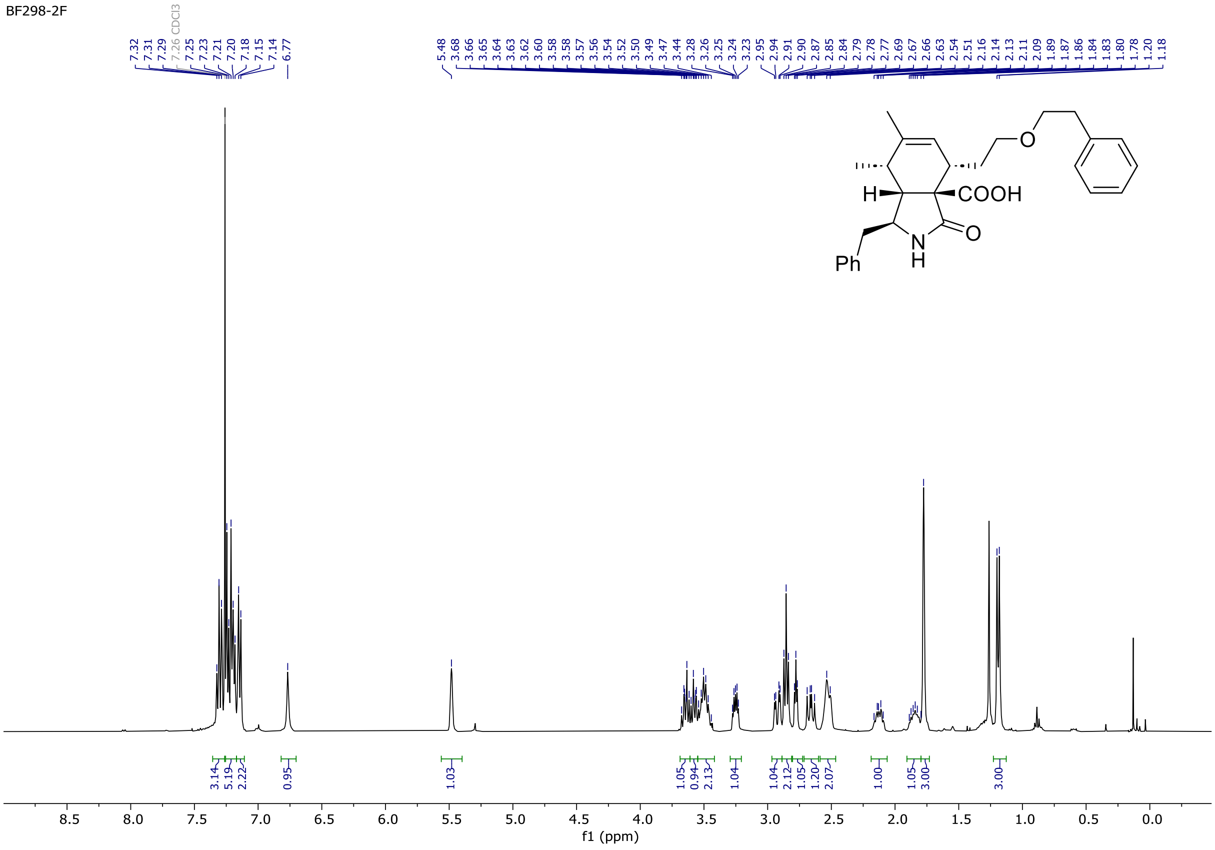
BF298-2F
7.32 7.31 7.29 7.26 CDCl3 7.25 7.23 7.21 7.20 7.18 7.15 7.14 6.77
5.48 3.68 3.66 3.65 3.64 3.63 3.62 3.60 3.58 3.58 3.57 3.56 3.54 3.52 3.50 3.49 3.47 3.44 3.28 3.26 3.25 3.24 3.23 2.95 2.94 2.91 2.90 2.87 2.85 2.84 2.79 2.78 2.77 2.69 2.67 2.66 2.63 2.54 2.51 2.16 2.14 2.13 2.11 2.09 1.89 1.87 1.86 1.84 1.83 1.80 1.78 1.20 1.18
COOH
H
O
N
H
Ph
3.14 5.19 2.22 0.95 1.03 1.05 0.94 2.13 1.04 1.04 2.12 1.05 1.20 2.07 1.00 1.05 3.00 3.00
f1 (ppm)

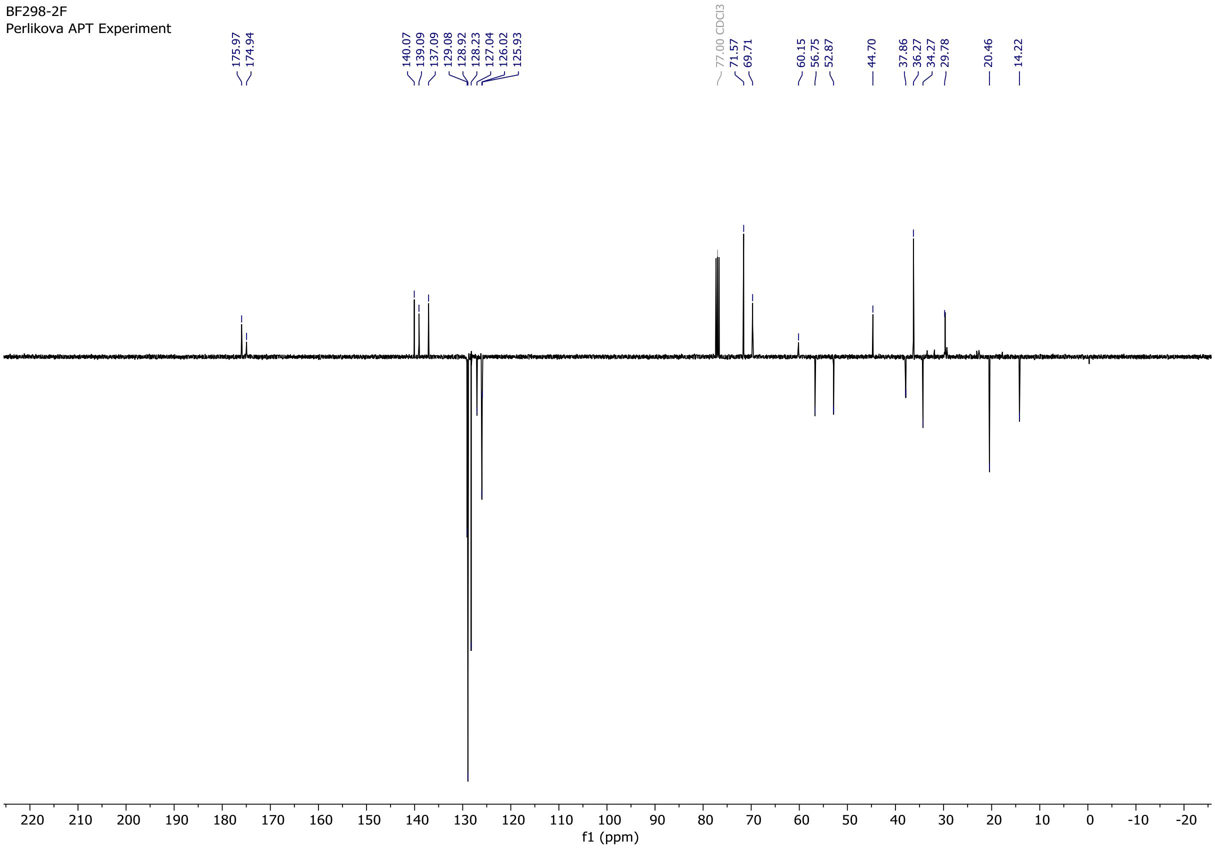
BF298-2F
Perlikova APT Experiment
175.97 174.94 140.07 139.09 137.09 129.08 128.92 128.23 127.04 126.02 125.93 77.00 CDCl3 71.57 69.71 60.15 56.75 52.87 44.70 37.86 36.27 34.27 29.78 20.46 14.22
f1 (ppm)

30

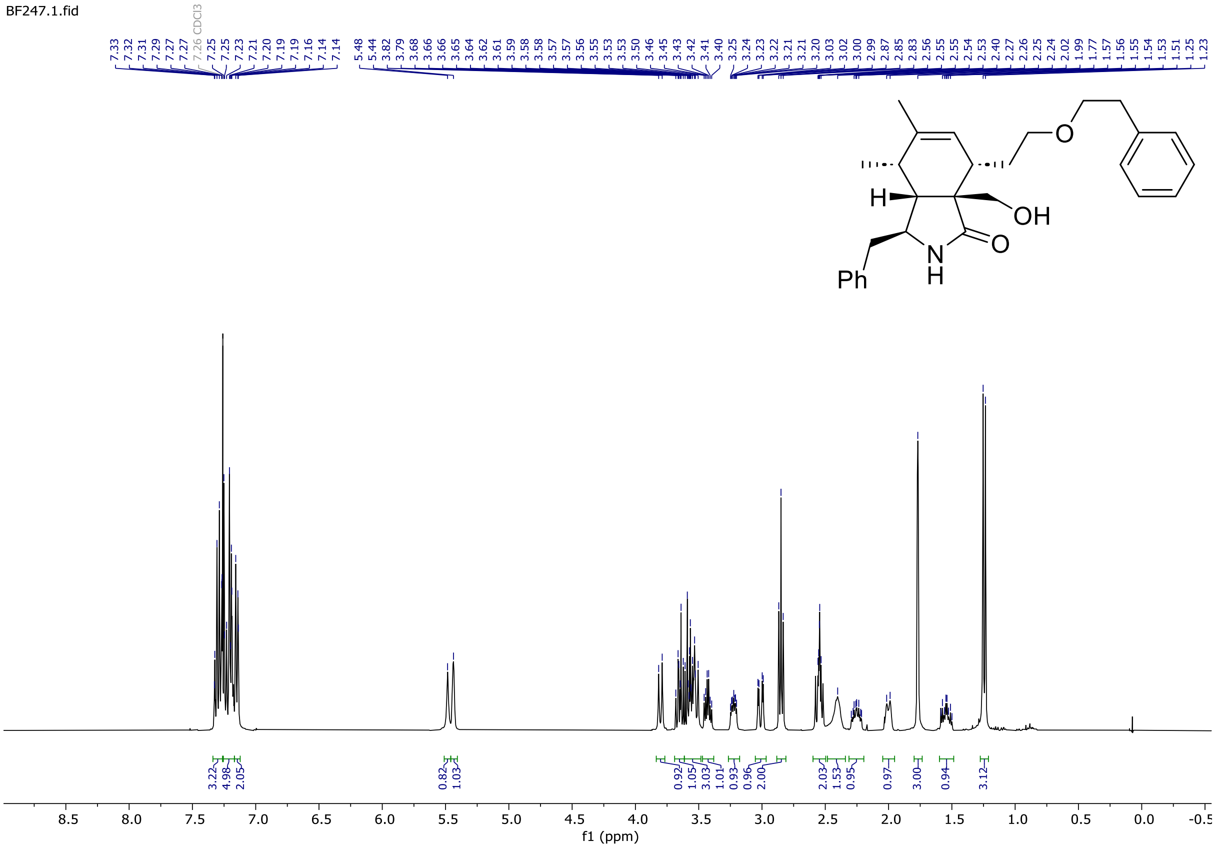
BF247.1.fid
f1 (ppm)
OH
O
N
H
Ph
H

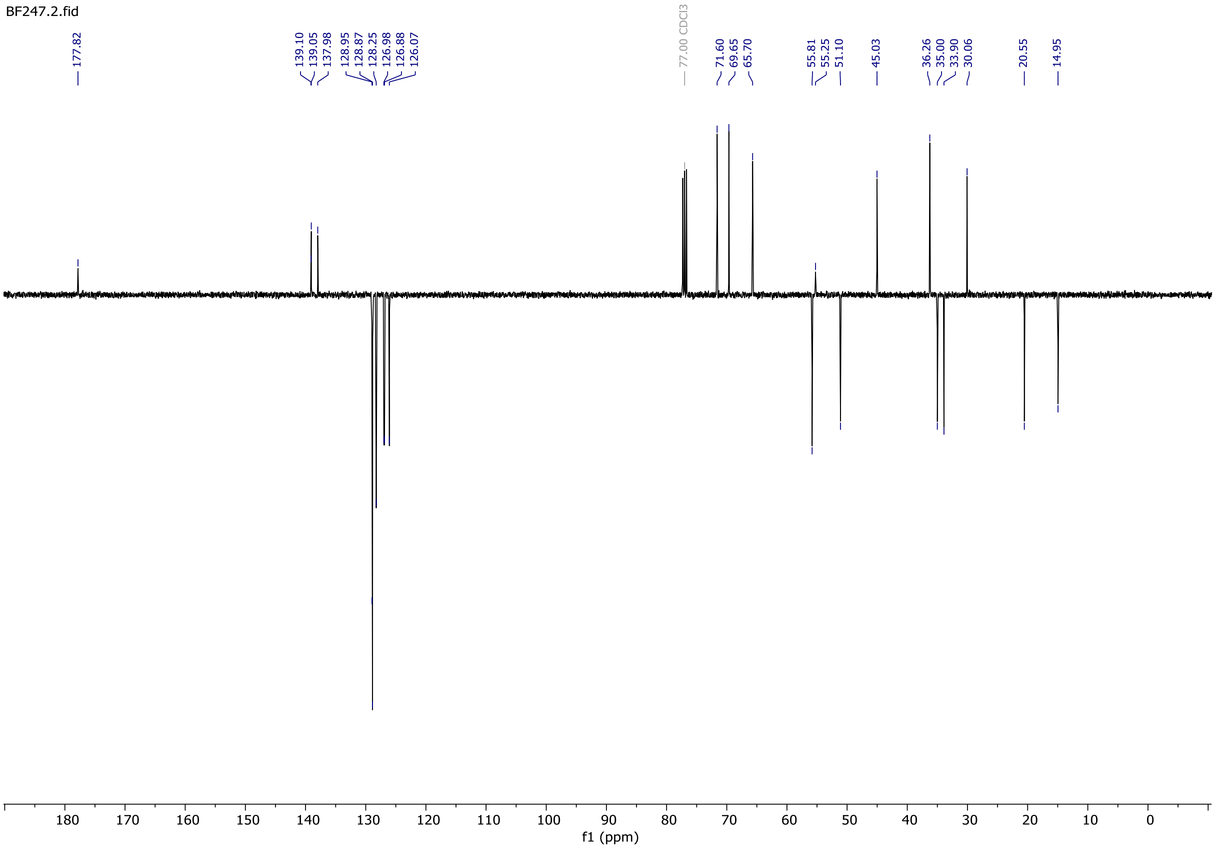
BF247.2.fid
177.82
77.00 CDCl3
f1 (ppm)
